# Modeling distinct pathogenesis of early- and late-onset preeclampsia via hierarchical representation learning of interactome

**DOI:** 10.1093/bib/bbag627

**Published:** 2026-11-13

**Authors:** Herdiantri Sufriyana, Yu-Wei Wu, Hua-Sheng Chiu, Pavel Sumazin, Emily Chia-Yu Su

**Affiliations:** Institute of Biomedical Informatics, College of Medicine, National Yang Ming Chiao Tung University, 155, Section 2, Linong Street, Beitou District, Taipei 112304, Taiwan; Graduate Institute of Biomedical Informatics, College of Medical Science and Technology, Taipei Medical University, 250, Wuxing Street, Xinyi District, Taipei 11031, Taiwan; Clinical Big Data Research Center, Taipei Medical University Hospital, 252, Wuxing Street, Xinyi District, Taipei 11031, Taiwan; Texas Children’s Cancer Center, Baylor College of Medicine, 6621, Fannin Street, Houston 77030, TX, United States; Texas Children’s Cancer Center, Baylor College of Medicine, 6621, Fannin Street, Houston 77030, TX, United States; Institute of Biomedical Informatics, College of Medicine, National Yang Ming Chiao Tung University, 155, Section 2, Linong Street, Beitou District, Taipei 112304, Taiwan; Graduate Institute of Biomedical Informatics, College of Medical Science and Technology, Taipei Medical University, 250, Wuxing Street, Xinyi District, Taipei 11031, Taiwan; Clinical Big Data Research Center, Taipei Medical University Hospital, 252, Wuxing Street, Xinyi District, Taipei 11031, Taiwan

**Keywords:** preeclampsia, menstruation, chorioamnionitis, HELLP syndrome, deep learning, large language model

## Abstract

Objectives: We developed and validated the pathogenesis models by hierarchical representation learning of interactomes connecting endometrial maturation, placentation, chorioamnionitis, and hemolysis, elevated liver enzyme, and low platelet (HELLP) syndrome. Methods: We utilized 19 gene sets from our previous work to infer interactomes to develop (*n* = 177) and validate (*n* = 352) explainable artificial intelligence models for each preeclampsia subtype using deep-insight visible neural network, gradient-weighted class activation mapping, and large language model. Results: The hierarchically learned representations identified novel genes for late-onset preeclampsia (LOPE), similar to chorioamnionitis (*ANKRD13A*, *SLA*), and HELLP syndrome (*PDE5A*). We also identified novel genes for early-onset preeclampsia (EOPE), similar to HELLP syndrome (*SWAP70*, *GREM2*, *GPR146*, *PIP5K1B*, *EZR*). Conclusions: Our pathogenesis models connected HELLP syndrome with both LOPE and EOPE. However, chorioamnionitis was only connected with LOPE.

## Introduction

The two-stage dysfunction theory is widely-accepted for explaining preeclampsia (PE) pathogenesis [1], which belongs to (Fig. 1a): pregnancy-induced hypertension (PIH) [2] and placental dysfunction-related diseases (PDDs), including fetal growth restriction (FGR) [3, 4]. PE may manifest as early- and late-onset PE (LOPE) [5–9], and may result in hemolysis, elevated liver enzyme, and low platelet (HELLP) syndrome, although it can occur independently of or precede PE [10]. However, this theory is typical for early-onset PE (EOPE) and shared with FGR (Fig. 1b).

**Figure 1:**
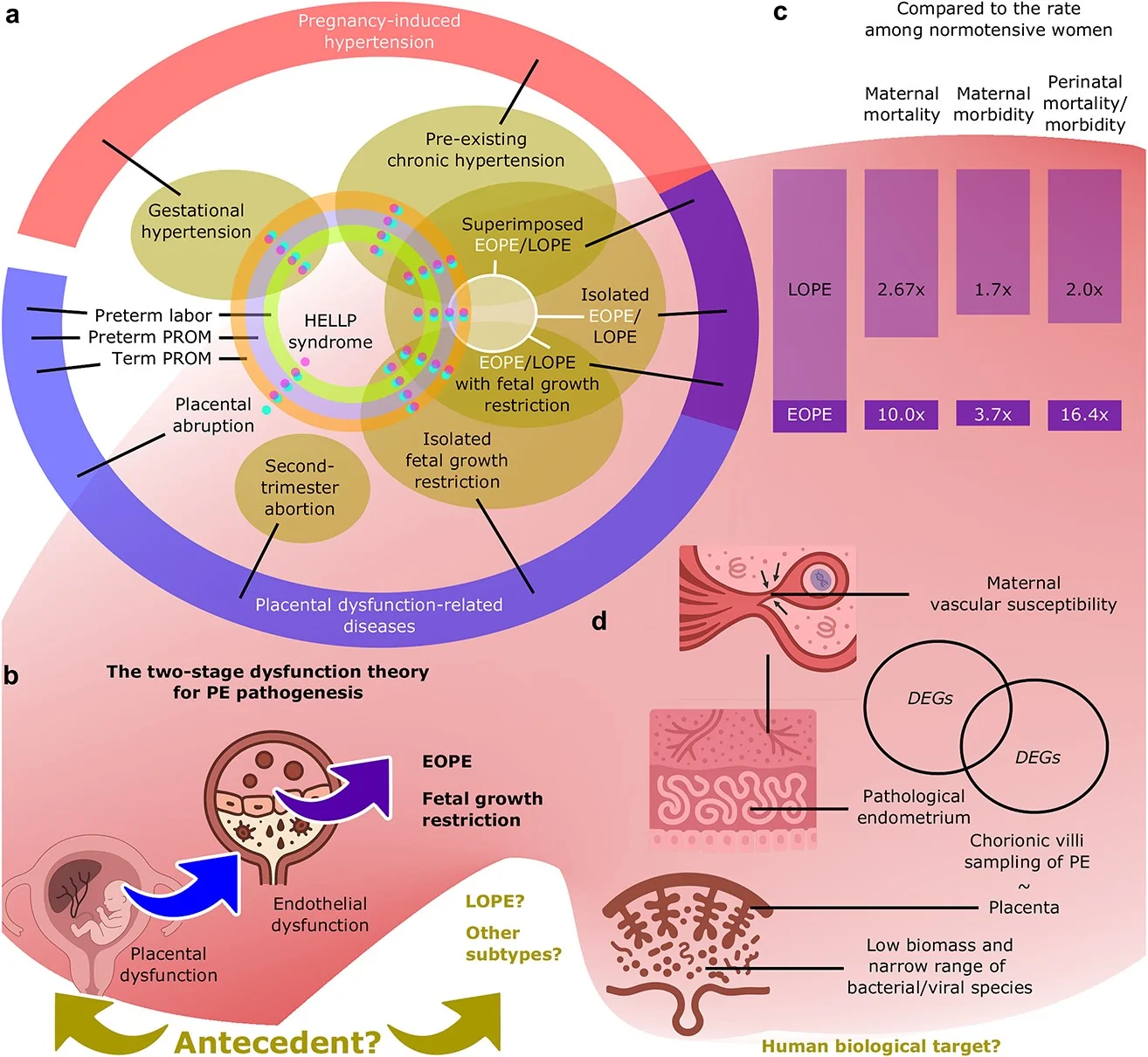
Summary of background knowledge in this study; (a) pregnancy conditions that are related to PE subtypes; (b) the two-stage dysfunction theory that is shared between EOPE and FGR; (c) impact representation of LOPE and EOPE; and (d) the potential antecedents of placental dysfunction in PE; red area covering panels a to d indicates what are already known; in a, HELLP syndrome and placental abruption are represented by small circles with magenta and turquoise colors, respectively; in c, the bars represent prevalence, while the numbers inside the bars indicate effect sizes [13] (see “Introduction”); DEG, differentially expressed gene; EOPE, early-onset PE; FGR, fetal growth restriction; HELLP, hemolysis, elevated liver enzyme, and low platelet; LOPE, late-onset PE; PE, preeclampsia; PROM, premature rupture of the membranes.

PE prevalence ranges from 2.7% to 8.2% worldwide [11, 12]. LOPE accounts for 87.74% of PE cases in the United States [13]. Although EOPE is more severe, LOPE leads to more significant impact due to greater number of cases (Fig. 1c). LOPE resulted in 2.67 and 1.7 times higher maternal mortality and morbidity, respectively, compared to those without PE [14]. Meanwhile, perinatal mortality and morbidity in LOPE were twice as high as those from normotensive pregnant women [13, 15]. Therefore, distinguishing both pathogeneses may help to reduce the adverse outcomes.

This PE pathogenesis theory proposes placental dysfunction that occurs before symptomatic, endothelial dysfunction [1]. The antecedents of placental dysfunction remain unknown (Fig. 1b). Several possibilities (Fig. 1d) have been revealed through associations between PE and endometrial maturation or decidualization [16–20], metagenomics profiling of the placenta [21–24], and maternal susceptibility to vascular dysfunction [25, 26]. However, no studies have identified human biological targets linked to the antecedents distinguishing LOPE from EOPE (Fig. 1d).

Previously, we identified transcriptomes differentiating LOPE and EOPE via differential expression or gene set enrichment analysis [27]. Yet, they do not model the gene–gene interactions to capture hierarchies and its network cannot facilitate validation. Meanwhile, common validation methods, e.g. quantitative polymerase chain reaction (qPCR), required a manageable number of gene candidates. To overcome these limitations, we employed predictive modeling via hierarchical representation learning, i.e. a deep insight visible neural network (DI-VNN) [28], in which representation learning preserves data [29], and it was hierarchically represented using gene-to-gene similarity values, their hierarchy, and gene expression [28]. We aimed to develop and validate pathogenesis models for EOPE and LOPE by hierarchical representation learning of their interactomes connecting endometrial maturation, decidualization, placentation, chorioamnionitis, and HELLP syndrome. We use the term “pathogenesis” to refer to the processes and factors associated with the development and maintenance of a disease, which may include downstream molecular changes, as distinguished from etiology, which concerns initiating causes [30].

## Methods

### Ethics approval and consent to participate

Ethical review was exempted by the Taipei Medical University Joint Institutional Review Board (TMU-JIRB number: N202106025). Consent to participate was obtained in the original studies that collected the data being used in this study.

### Study design

We utilized gene sets from our previous work [27] to infer interactomes for each PE subtype. An interactome was used to construct an explainable artificial intelligence (XAI) model for each PE-subtype pathogenesis model. We developed and externally validated these models using different messenger ribonucleic acid (mRNA) microarrays. They were retrieved from Gene Expression Omnibus (GEO) and ArrayExpress. Predictive modeling was applied to justify a pathogenesis model in this study (Supplementary Box S4). A sample was predicted to be negative or positive of a PE subtype (i.e. an outcome) by a gene set (i.e. predictors) in an XAI model.

The datasets consisted of mRNA expression data of total RNA extracts. Three datasets with the same microarray platform were utilized for the model development set (*n* = 177), while five datasets with diverse microarray platform were utilized for the model test set (*n* = 352). To handle cross-platform batch effects arising from the diverse microarray platforms, the test set was normalized by aligning each sample’s expression distribution to the average expression profile of controls in the development set using quantile normalization with a target distribution. Principal component analysis (PCA) confirmed that samples were not separated by experiment groups after normalization. Details on data preprocessing and gene set discovery were described in our previous work [27]. The test set was independent and challenging due to its diverse biological and technical characteristics, which facilitated a robust test for our pathogenesis models.

For the outcome definition, samples were positives if they were: (i) late-onset, severe PE; (ii) early-onset, severe PE; (iii) late-onset, mild-to-moderate PE with FGR; and (iv) late-onset, severe PE with FGR. Meanwhile, the samples were negatives if they were normotension without FGR. Initially, we determined outcome definitions by combining four axes related to PE: (a) PIH; (b) PDDs; (c) onset; and (d) severity. However, both the development and test sets were only sufficient for 4 of 28 potential combinations considering sample size estimation and gene set discovery. Details on outcome definition and sample size estimation were described in our previous work [27].

### Hierarchical representation learning

For predictors in the pathogenesis models (Supplementary Tables S1 and S2), we used gene sets which were discovered in our previous work [27]. A gene set was selected if it was a significant overlap between a pair of differentially expressed genes (DEGs) of a PE subtype and each of the associated factors. For the gene sets in this study, the associated factors were: (1) endometrial maturation; (2) placentation; (3) chorioamnionitis; and (4) HELLP syndrome. The DEGs of a PE subtype were identified by comparing normotension without FGR with each subtype. For endometrial maturation, the DEGs were identified by comparing proliferative- with early-, mid-, or late-secretory endometrium. Meanwhile, the DEGs of placentation were identified by comparing term with either first- or second-trimester placenta. We also identified the placental DEGs by comparing samples without and with either chorioamnionitis or HELLP syndrome at second and third trimesters. However, the gene set of HELLP syndrome at second trimester did not pass the selection criterion. Eventually, none of the samples from the test set was used to determine these gene sets. Hereafter, we shall not mention the control groups of the gene sets and the term “overlapped DEGs” for brevity.

Interactomes were identified for each gene set by knowledge- and data-driven ontology inference methods. The former applied pathway enrichment analysis (PEA) utilizing gene ontology (GO) database, while the latter applied clique-extracted ontology (CliXO) algorithm. Both resulted in directed acyclic graphs which would be used for constructing neural network architectures of the XAI models. Therefore, there were potentially 38 pathogenesis models from 19 gene sets and 2 ontology inference methods. However, no ontology might be inferred by an ontology inference method for a gene set. See technical details on ontology inference in Supplementary Box S1.

We developed and externally validated the XAI models by applying a DI-VNN [28]. The input representation of DI-VNN was a 3D feature map onto which we mapped gene sets. The coordinates were determined by *t*-moderated stochastic neighbor embedding (*t*-SNE) algorithm with Barnes–Hut approximation. *t*-SNE was selected for its emphasis on preserving local neighborhood structure in the gene–gene similarity space, which is critical for convolutional neural network processing where spatially proximal features are jointly analyzed by convolutional filters. Unlike PCA, which captures global linear variance, *t*-SNE preserves nonlinear co-expression relationships through optimization of Kullback–Leibler divergence with a heavy-tailed Student’s *t*-distribution that prevents crowding of dissimilar points. We conducted an ablation study comparing *t*-SNE with PCA and uniform manifold approximation and projection (UMAP) to evaluate the sensitivity of the models to alternative representations. This algorithm mapped genes according to the similarity scores which were also used for CliXO algorithm. Using each interactome, we subset a feature map hierarchically by the inferred ontologies. We also obtained a learned representation for each ontology. Both prediction and learned representation of a parent ontology also included the inputs and the learned representations of its child ontologies. Eventually, to train or fit the DI-VNNs, we followed the same hyperparameters as the previous study [28]. See technical details on gene mapping, feature map subsetting, and DI-VNN fitting in Supplementary Box S1.

### Statistical analysis

To evaluate the pathogenesis models, we used the test sets to compute the average values and 95% confidence intervals (CIs) of positive predictive value (PPV), true positive rate (TPR), negative predictive value (NPV), and true negative rate (TNR). The interval estimates were obtained by 30-time bootstrapping of each test set. A threshold of the predicted probabilities was chosen based on the development set if the threshold resulted in maximum difference of net benefits with those of the references. These were treat-all and treat-none scenarios in which all samples were respectively predicted as positive and negative. A pathogenesis model was valid if there was at least one ontology of which the lower bounds were finite and >0 for a pair of evaluation metrics using both development and test sets, i.e.: (i) PPV-TPR; or (ii) NPV-TNR. Respectively, the pathogenesis models were valid for representing positive or negative outcome. Furthermore, a pathogenesis model was valid if at least one ontology had an absolute difference of ≤25% between the averages using the development and test sets for the pair of evaluation metrics. We also evaluated explainability of the valid pathogenesis models using gradient-weighted class activation mapping (grad-CAM) method. A pathogenesis model was valid if at least one gene has a grad-CAM value >97.5th percentile of those in the same ontology. Furthermore, a pathogenesis model was valid if at least one ontology had a lower bound of area under the curve (AUC) of the receiver operating characteristic (AUC-ROC) >0.5 in both the development and test sets. When multiple ontologies shared identical eligible gene sets, we reported the one closest to the root in the ontology hierarchy. We computed AUC-ROC, AUC of the precision-recall curve (AUC-PRC), and root mean square error (RMSE) at the ontology level (Supplementary Tables S6, S8, S10, S12, S14, S16, and S18). Eventually, we used gene set artificial intelligence (GSAI), which was powered by a large language model (LLM) [31], to annotate the ontology function. We selected generative pretrained transformer-4 (GPT-4) as an LLM which was used for GSAI. GSAI synthesizes known functions of multiple genes into coherent biological narratives specific to their co-occurrence in an ontology, which is not directly available from traditional annotation databases (e.g. GO, Kyoto encyclopedia of genes and genomes [KEGG]) that list individual gene functions. Each GSAI annotation includes a confidence score [31]. All GSAI-generated annotations (Tables 1 and 2 and Supplementary Tables S3 and S4) were reviewed by domain experts for biological accuracy. We acknowledge that LLM-based annotations carry a risk of hallucination, and the annotations should be considered hypothesis-generating rather than definitive functional assignments.

**Table 1:** Discovery of gene set function by GSAI for late-onset, severe PE.

**Representation of**		**Ontology**	**GSAI**	
			**Query**	**Response summary** ^**a**^
Chorio-amnionitis	Third trimester	CliXO:86	*ANKRD13A*, *SLA*	The proteins in this system are primarily involved in the regulation of protein ubiquitination and immune response. *ANKRD13A* [D/D] contributes to the maintenance of protein homeostasis through its role in protein ubiquitination and degradation, while *SLA* [U/U] plays a crucial role in immune response regulation through its inhibitory effect on TCR signaling. These processes are essential for maintaining cellular function and immune system balance
HELLP syndrome	Third trimester	CliXO:70	*PDE5A*	The primary biological process performed by *PDE5A* [D/D] is the degradation of cGMP, a process that has significant implications for vascular and neuronal function. The protein’s role in this process is well-established, hence the high confidence score

^a^See Supplementary Table S3 for response details and each mention of gene name is modified by including up (U)- or down (D)-regulation status of respectively the associated factor and third-trimester placenta under late-onset, severe PE.

CliXO, clique-extracted ontology; GSAI, gene set artificial intelligence; HELLP, hemolysis, elevated liver enzyme, and low platelet; PE, preeclampsia.

**Table 2:** Discovery of gene set function by GSAI for early-onset, severe PE.

**Representation of**		**Ontology**	**GSAI**	
			**Query**	**Response summary** ^**a**^
HELLP syndrome	Third trimester	Root	ALKBH5, *SWAP70*, DUSP1, KLF4, *GREM2*, *GPR146*, *PIP5K1B*, *EZR*	The proteins in this system are involved in various aspects of cellular differentiation and signal transduction. They contribute to the regulation of gene expression (i.e. ALKBH5 [U/U]^b^), actin cytoskeleton organization (i.e. *SWAP70* [U/U] and *PIP5K1B* [D/D]^b^), MAP kinase signaling (i.e. DUSP1 [U/U]^b^), stem cell pluripotency (i.e. KLF4 [U/U]^b^), organogenesis (i.e. *GREM2* [U/U]^b^), and potentially other signaling pathways (i.e. *GPR146* [U/U] and *EZR* [U/U]^b^). Their interplay likely results in a complex network of regulatory mechanisms that control cellular differentiation and response to external signals

^a^See Supplementary Table S4 for response details and each mention of gene name is modified by including up (U)- or down (D)-regulation status of respectively the associated factor and third-trimester placenta under early-onset, severe PE.

^b^We redacted a part in parentheses to mention gene names, that were related to that part based on Supplementary Table S4, if they were not mentioned in the response summary by GSAI.

CliXO, clique-extracted ontology; GSAI, gene set artificial intelligence; HELLP, hemolysis, elevated liver enzyme, and low platelet; PE, preeclampsia.

## Results

### Pathogenesis models

Of 19 gene sets, we identified 9 interactomes (47%, *n* = 9/19) by PEA utilizing GO database (Fig. 2a) and 19 interactomes (100%, *n* = 19/19) by CliXO algorithm (Fig. 2b). All 28 interactomes resulted in 28 pathogenesis models. Only 10 (36%, *n* = 10/28), 7 (70%, *n* = 7/10), and 3 (43%, *n* = 3/7) of them fulfilled the criteria at ontology, gene, and gene levels, respectively. Eventually, three pathogenesis models were validated for 2 of 4 PE subtypes, i.e. late- and early-onset, severe PE without FGR. Hereafter, we only mention LOPE and EOPE for brevity. Both are severe and isolated (i.e. without FGR) in this study. The sample characteristics were already described in our previous study [27].

**Figure 2:**
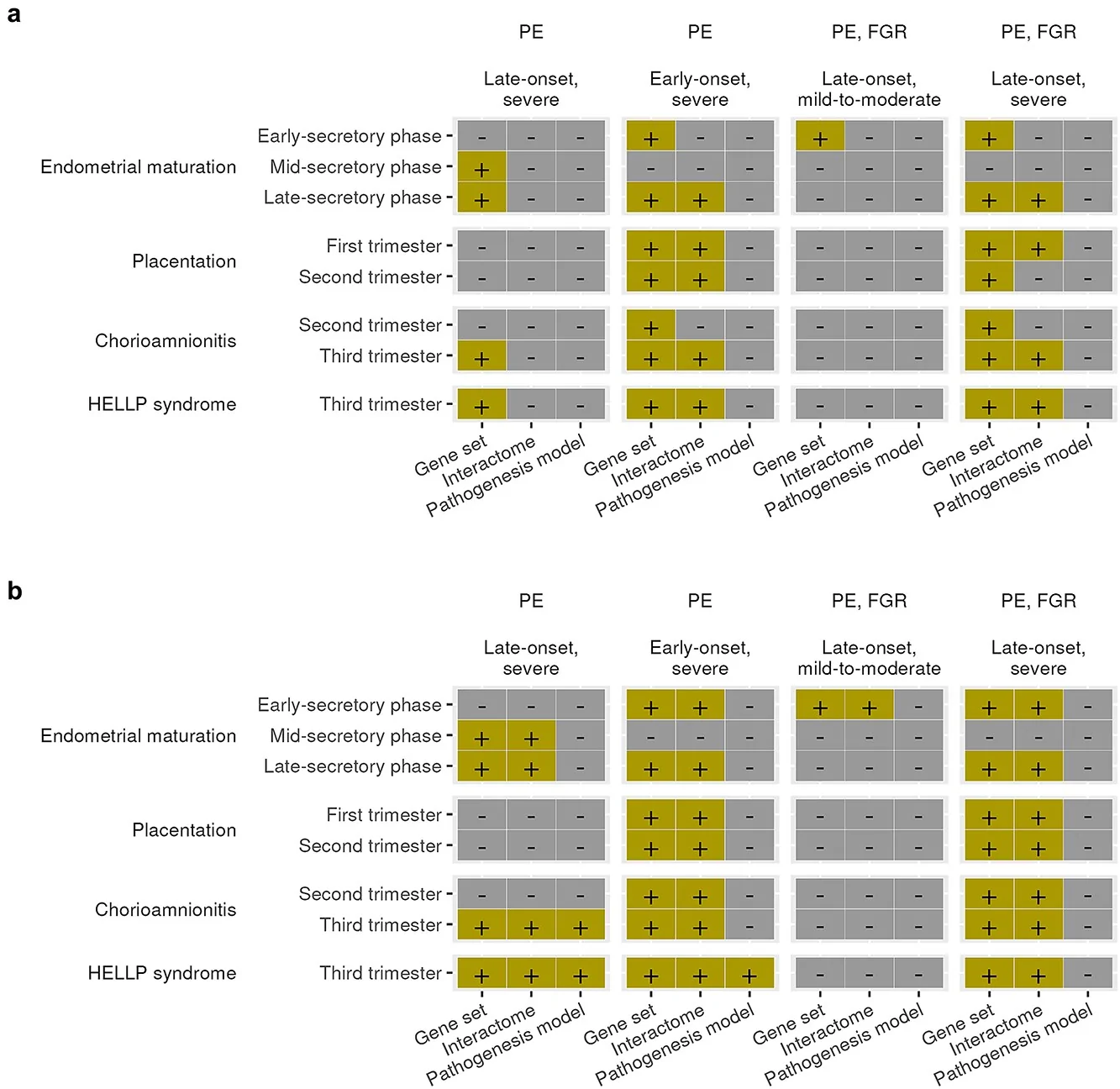
Availability of gene sets, interactomes, and pathogenesis models of a PE subtype and each of the associated factors; the interactomes were inferred by: (a) PEA utilizing GO database; and (b) CliXO algorithm; positive (+) and negative (−) symbols mean “available” and “not available,” respectively; colors are assigned to improve visualization by augmenting the symbols; CliXO, clique-extracted ontology; FGR, fetal growth restriction; GO, gene ontology; HELLP, hemolysis, elevated liver enzyme, and low platelet; PE, preeclampsia; PEA, pathway enrichment analysis.

All the pathogenesis models for LOPE fulfilled the criteria at ontology level for PPV and TPR; thus, they directly represented a positive outcome. For EOPE, the pathogenesis models fulfilled the criteria at ontology level for NPV and TNR; thus, they represented a negative outcome. If the prediction was negative, the outcome was truly normotension without FGR. However, the outcome might or might not be EOPE, if the prediction was positive. Other factors were also needed to sufficiently result in EOPE.

At the ontology level, the AUC-ROC ranged from 0.773 to 0.783. The probability thresholds selected from the development set ranged from 0.11 to 0.17 across models. Net benefit bar plots (Supplementary Figs S1, S2, S4, and S6–S9), threshold-specific confusion matrices (Supplementary Tables S5, S7, S9, S11, S13, S15, and S17), ROC curves (Supplementary Figs S3, S5, and S10), and AUC-ROC, AUC-PRC, and RMSE (Supplementary Tables S6, S8, S10, S12, S14, S16, and S18) are provided in the Supplementary Materials. Definitions of genes identified in the validated pathogenesis models are provided in Supplementary Table S19.

We conducted an ablation study comparing *t*-SNE with PCA and UMAP for the 3D feature map construction across all seven pathogenesis models (Supplementary Table S20). Each model was retrained with the alternative dimensionality reduction method and evaluated on the same external validation set used for the main study (*n* = 213–218). Across the seven models, *t*-SNE was the best performer by internal validation. Despite the stringent challenge of diverse microarray platforms in the external validation set, *t*-SNE maintained the highest mean AUC-ROC of 0.591 (95% CI 0.578–0.604). This was significantly above random chance (0.5). PCA achieved 0.497 (95% CI 0.467–0.527) and UMAP achieved 0.438 (95% CI 0.420–0.457). These results support the use of *t*-SNE for feature map construction, consistent with its emphasis on preserving local neighborhood structure in gene co-expression space.

### Hierarchical learning representation of late-onset, severe preeclampsia

Two pathogenesis models were valid for LOPE. The gene sets were overlaps between the DEGs of LOPE and these associated factors: (a) third-trimester placenta under chorioamnionitis (Fig. 3a); and (b) third-trimester placenta under HELLP syndrome (Fig. 3a). The functions of the eligible gene sets were annotated by GSAI as shown in Supplementary Table S3.

**Figure 3:**
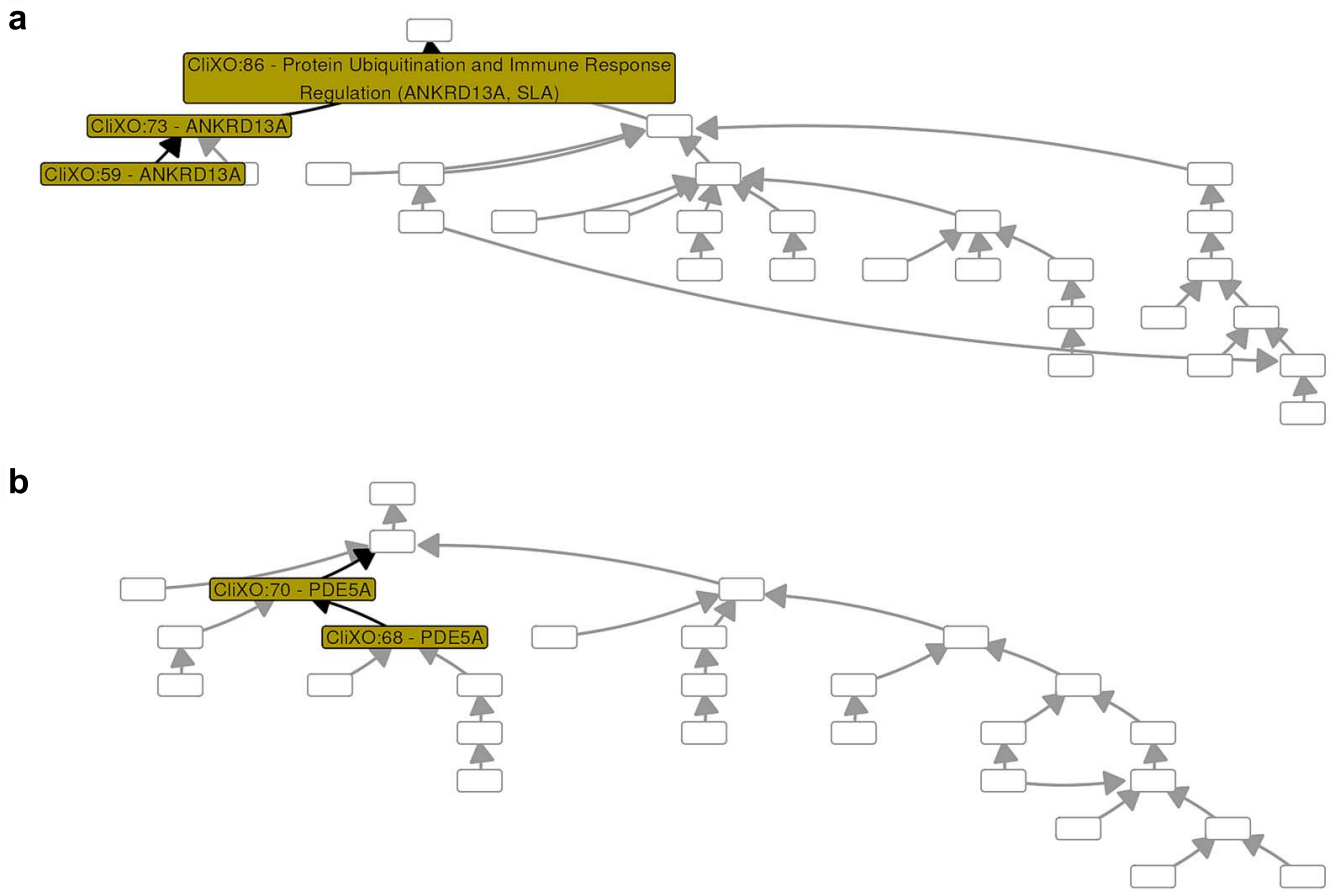
Hierarchical learning representation of late-onset, severe PE interactome; the representation was connected with (a) third-trimester placenta under chorioamnionitis; and (b) third-trimester placenta under HELLP syndrome; a node for an ontology is colored and labeled if the ontology was eligible and has at least one gene that was eligible; a label consists of ontology code and name with gene names (i.e. HGNC symbols); if there is only one gene, no ontology name is shown; to improve path visualization, an edge from a colored node is made bolder than that from uncolored node; CliXO, clique-extracted ontology; HELLP, hemolysis, elevated liver enzyme, and low platelet; HGNC, the human genome organization gene nomenclature; PE, preeclampsia.

In third-trimester placenta under chorioamnionitis (see GSAI annotation in Supplementary Box S2), *ANKRD13A*, and *SLA* were in the same ontology (CliXO:86) with an AUC-ROC of 0.783 (95% CI 0.745–0.821) (Fig. 3a). *ANKRD13A* was downregulated in third-trimester placenta under either chorioamnionitis or LOPE (Table 1), while *SLA* was upregulated in third-trimester placenta under either chorioamnionitis or LOPE (Table 1).

In third-trimester placenta under HELLP syndrome, *PDE5A* was downregulated (Table 1). The gene expression was regulated the same way for LOPE. *PDE5A* alone was predictive for LOPE in CliXO:70 with an AUC-ROC of 0.780 (95% CI 0.757–0.804) (Fig. 3a). See details on the GSAI annotation in Supplementary Box S2.

### Hierarchical learning representation of early-onset, severe preeclampsia

One pathogenesis model was valid for EOPE. The gene set was the overlap between DEGs of EOPE and third-trimester placenta under HELLP syndrome (Fig. 4). The function of the eligible gene set was annotated by GSAI as (Supplementary Table S4).

**Figure 4:**
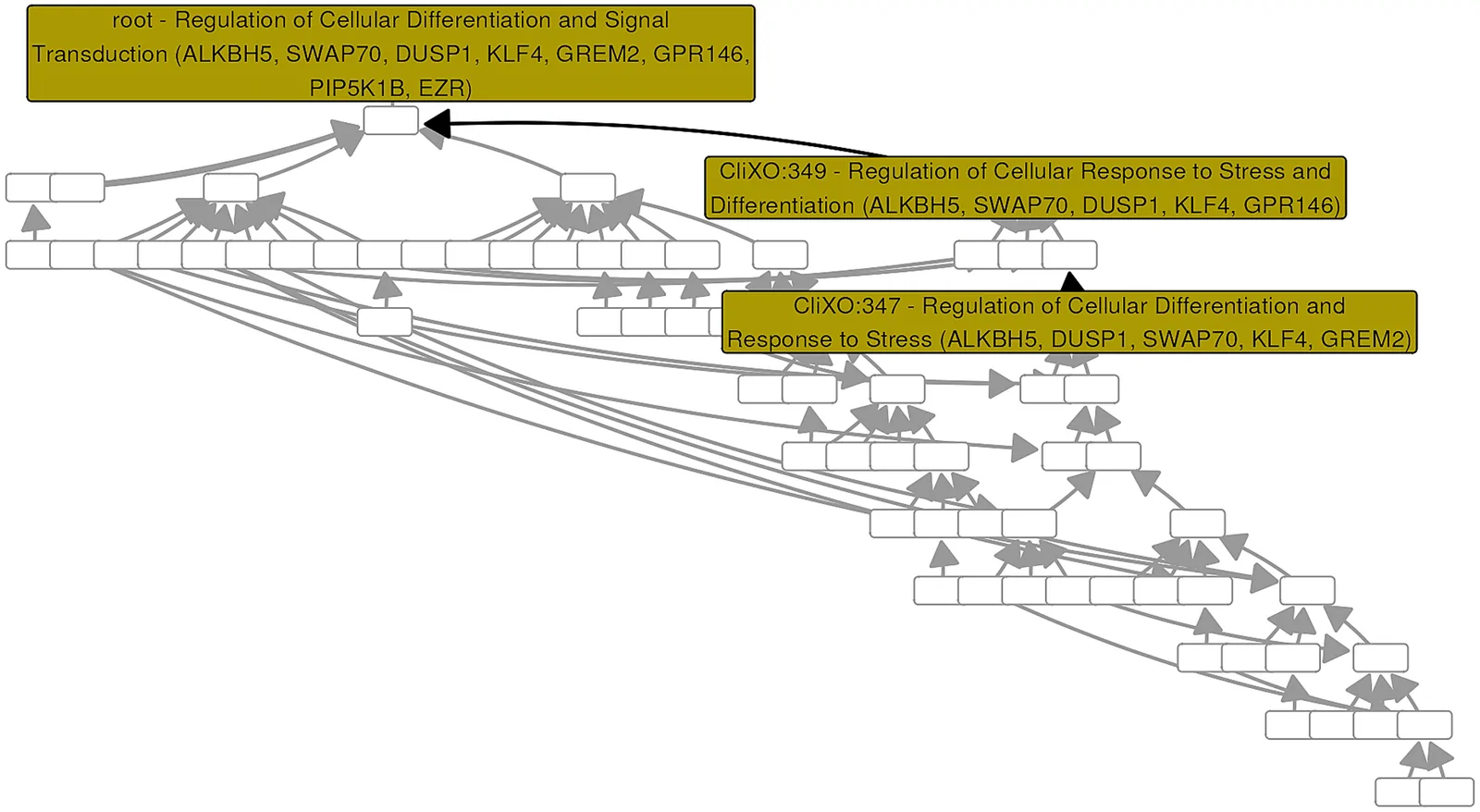
Hierarchical learning representation of early-onset, severe PE interactome; the representation was connected with third-trimester placenta under HELLP syndrome; a node for an ontology is colored and labeled if the ontology was eligible and has at least one gene that was eligible; a label consists of ontology code and name with gene names (i.e. HGNC symbols); if there is only one gene, no ontology name is shown; to improve path visualization, an edge from a colored node is made bolder than that from uncolored node; HELLP, hemolysis, elevated liver enzyme, and low platelet; HGNC, the human genome organization gene nomenclature; PE, preeclampsia.

In third-trimester placenta under HELLP syndrome, at least four genes were upregulated (Table 2). These genes were: (1) *ALKBH5*, (2) *SWAP70*, (3) *DUSP1*, and (4) *KLF4*. They were also regulated the same way for EOPE. To be predictive in the pathogenesis model (Fig. 4), all the four genes should be in the same ontology with either *GREM2* or *GPR146*, but not both, except there were *PIP5K1B* and *EZR*. The former was downregulated, while the others were upregulated, which might or might not result in EOPE. Nonetheless, the opposite regulations resulted in normotension without FGR. The root ontology had an AUC-ROC of 0.773 (95% CI 0.754–0.792). See the GSAI annotation in Supplementary Box S2.

## Discussion

Among the validated biological processes, novel genes were identified for both subtypes (Supplementary Table S19 and Supplementary Box S3). For LOPE, *ANKRD13A* and *SLA* were novel findings associated with chorioamnionitis, and *PDE5A* was associated with HELLP syndrome. Meanwhile, we also found novel genes for EOPE associated with HELLP syndrome (i.e. *SWAP70*, *GREM2*, *GPR146*, *PIP5K1B*, and *EZR*). Our findings that chorioamnionitis was connected only with LOPE highlight the importance of assessing competing risks during early trimesters, as early interventions targeting shared risk factors among competing conditions could enhance both predictive accuracy and patient outcomes. The observed relationship between PE and HELLP syndrome for both subtypes underscores the need for integrated, multi-system assessments in pregnancy care.

The DI-VNN method allows systematic identification of which genes contribute either LOPE or EOPE. The contribution of a gene was not only evaluated according to its expression value but also its correlation to other genes. These values were fitted to a specific PE subtype, enabling validation of the pathogenesis models using new data. Using these models, this study could differentiate LOPE from EOPE in the antecedents of placental dysfunction. Our models also identify other specific genes as specific contexts of the potential mechanism preceding placental dysfunction that distinguish EOPE and LOPE. Consistent with this network-based approach, recent studies have demonstrated the utility of graph-based representations for biomedical applications such as cell type annotation [32, 33], supporting the broader applicability of these methods in biomedicine. We note that our pathogenesis models identify factors associated with the development and maintenance of each PE subtype, which may include downstream molecular consequences rather than initiating causes. This is consistent with the distinction between pathogenesis and etiology [30], where pathogenetic factors do not necessarily precede disease onset but may perpetuate and maintain the condition. By narrowing gene candidates from high-dimensional transcriptomic data through hierarchical representation learning, our models are designed to enable targeted experimental validation that conventional transcriptomic and network analyses alone cannot achieve.

However, we are aware of several limitations in this study. First, mRNA expression findings in this study may represent pathogenetic factors—downstream consequences that maintain or worsen the condition—rather than etiological factors that initiate it. Yet, our pathogenesis models narrowed the gene candidates to a manageable number, which is now prioritized for targeted experimental validation. Future studies using qPCR, trophoblast assays, decidualization models, or organoid systems on these specific candidates would be needed to determine whether these factors are etiological or pathogenetic. Second, we could not validate the pathogenesis models for other PIH and PDD subtypes using the gene sets, which we identified previously [27] due to data unavailability. The validated pathogenesis models for LOPE and EOPE might indicate the common genes with those for gestational hypertension and isolated FGR. Nevertheless, we could differentiate LOPE from EOPE, which is important to gain insights on LOPE to expand early intervention strategies for PE. Third, we did not compare hierarchical DI-VNN with simpler predictive models or non-hierarchical neural networks. DI-VNN was chosen for its ability to preserve ontology hierarchy in the network architecture, enabling explainability through learned representations at each ontology level [28]. Fourth, our ablation study compared *t*-SNE with only two alternative dimensionality reduction methods (PCA and UMAP) for feature map construction (Supplementary Table S20). Nevertheless, these methods represent the three major paradigms: linear projection (PCA), local neighborhood preservation (*t*-SNE), and manifold approximation (UMAP), providing a principled comparison across fundamentally different approaches.

In conclusion, we have developed and validated pathogenesis models of LOPE and EOPE. The models connected HELLP syndrome with both LOPE and EOPE. However, chorioamnionitis was only connected with LOPE. The EOPE models only revealed protective roles against this condition. Future studies are needed to identify more factors that required for EOPE in addition to the loss of these protective mechanisms and to evaluate the therapeutic effect over the LOPE biological targets as proposed in this study.

**Key Points**

- Validated hierarchical representation learning on preeclampsia (PE) interactomes.
- Identified novel late-onset PE genes: *ANKRD13A*, *SLA*, and *PDE5A*.
- Hemolysis, elevated liver enzyme, and low platelet syndrome linked to both late- and early-onset PE.
- Chorioamnionitis was only connected with late-onset PE.

## Supplementary Material

supp_rev3_bbag627

## Data Availability

Data are available publicly in GEO and ArrayExpress, as described in our previous work [27] (https://github.com/herdiantrisufriyana/pec/blob/master/supplementary_material.pdf). We also publicly shared the source codes for data analysis (https://github.com/herdiantrisufriyana/pec) and model development (https://github.com/herdiantrisufriyana/pec_hrl).

## Abbreviations

AUC, area under the curve; AUC-PRC, AUC of the precision-recall curve; AUC-ROC, AUC of the receiver operating characteristic; EOPE, early-onset preeclampsia; cGMP, cyclic guanosine monophosphate; CI, confidence interval; CliXO, clique-extracted ontology; CVS, chorionic villi sampling; DAG, directed acyclic graph; DEG, differentially expressed gene; DI-VNN, deep insight visible neural network; EV, extracellular vesicle; FGR, fetal growth restriction; GEO, Gene Expression Omnibus; GO, gene ontology; GPT-4, generative pretrained transformer-4; grad-CAM, gradient-weighted class activation mapping; GSAI, gene set artificial intelligence; HELLP, hemolysis elevated liver enzyme and low platelet; HESC, human endometrial stromal cell; KEGG, Kyoto encyclopedia of genes and genomes; LOPE, late-onset preeclampsia; LLM, large language model; m6A, N6-methyladenosine; mRNA, messenger ribonucleic acid; NK, natural killer; NPV, negative predictive value; PE, preeclampsia; PCA, principal component analysis; PEA, pathway enrichment analysis; PDD, placental dysfunction-related disease; PIH, pregnancy-induced hypertension; PPV, positive predictive value; qPCR, quantitative polymerase chain reaction; TMU-JIRB, Taipei Medical University Joint Institutional Review Board; TNR, true negative rate; TPR, true positive rate; *t*-SNE, *t*-moderated stochastic neighbor embedding; UMAP, uniform manifold approximation and projection; XAI, explainable artificial intelligence.

## References

[1] Kornacki J, Olejniczak O, Sibiak R et al. Pathophysiology of pre-eclampsia-two theories of the development of the disease. *Int J Mol Sci* 2023;25:307. 10.3390/ijms25010307 PMC10779413 38203478

[2] Wu P, Green M, Myers JE. Hypertensive disorders of pregnancy. *BMJ* 2023;381:e071653. 10.1136/bmj-2022-071653 37391211

[3] Brosens I, Pijnenborg R, Vercruysse L et al. The “great obstetrical syndromes” are associated with disorders of deep placentation. *Am J Obstet Gynecol* 2011;204:193–201. 10.1016/j.ajog.2010.08.009 PMC3369813 21094932

[4] Hoffman MK . The great obstetrical syndromes and the placenta. *BJOG* 2023;130:8–15. 10.1111/1471-0528.17613 PMC10834844 37530495

[5] Rana S, Lemoine E, Granger JP et al. Preeclampsia: pathophysiology, challenges, and perspectives. *Circ Res* 2019;124:1094–112. 10.1161/CIRCRESAHA.118.313276 30920918

[6] Cruz JO, Conceição I, Tosatti JAG et al. Global DNA methylation in placental tissues from pregnant with preeclampsia: a systematic review and pathway analysis. *Placenta* 2020;101:97–107. 10.1016/j.placenta.2020.09.004 32942147

[7] Guo F, Zhang B, Yang H et al. Systemic transcriptome comparison between early- and late-onset pre-eclampsia shows distinct pathology and novel biomarkers. *Cell Prolif* 2021;54:e12968. 10.1111/cpr.12968 PMC7848957 33332660

[8] Chen H, Aneman I, Nikolic V et al. Maternal plasma proteome profiling of biomarkers and pathogenic mechanisms of early-onset and late-onset preeclampsia. *Sci Rep* 2022;12:19099. 10.1038/s41598-022-20658-x PMC9646706 36351970

[9] Huang Y, Sun Q, Zhou B et al. Lipidomic signatures in patients with early-onset and late-onset preeclampsia. *Metabolomics* 2024;20:65. 10.1007/s11306-024-02134-x PMC11180640 38879866

[10] Stojanovska V, Zenclussen AC. Innate and adaptive immune responses in HELLP syndrome. *Front Immunol* 2020;11:667. 10.3389/fimmu.2020.00667 PMC7174768 32351511

[11] Abalos E, Cuesta C, Grosso AL et al. Global and regional estimates of preeclampsia and eclampsia: a systematic review. *Eur J Obstet Gynecol Reprod Biol* 2013;170:1–7. 10.1016/j.ejogrb.2013.05.005 23746796

[12] Macedo TCC, Montagna E, Trevisan CM et al. Prevalence of preeclampsia and eclampsia in adolescent pregnancy: a systematic review and meta-analysis of 291,247 adolescents worldwide since 1969. *Eur J Obstet Gynecol Reprod Biol* 2020;248:177–86. 10.1016/j.ejogrb.2020.03.043 32283429

[13] Lisonkova S, Joseph KS. Incidence of preeclampsia: risk factors and outcomes associated with early- versus late-onset disease. *Am J Obstet Gynecol* 2013;209:544.e1–12. 10.1016/j.ajog.2013.08.019 23973398

[14] Lisonkova S, Sabr Y, Mayer C et al. Maternal morbidity associated with early-onset and late-onset preeclampsia. *Obstet Gynecol* 2014;124:771–81. 10.1097/AOG.0000000000000472 25198279

[15] Büyükeren M, Çelik HT, Örgül G et al. Neonatal outcomes of early- and late-onset preeclampsia. *Turk J Pediatr* 2020;62:812–9. 10.24953/turkjped.2020.05.013 33108084

[16] Rabaglino MB, Uiterweer EDP, Jeyabalan A et al. Bioinformatics approach reveals evidence for impaired endometrial maturation before and during early pregnancy in women who developed preeclampsia. *Hypertension* 2015;65:421–9. 10.1161/HYPERTENSIONAHA.114.04481 PMC4290371 25421975

[17] Conrad KP, Rabaglino MB, Uiterweer EDP. Emerging role for dysregulated decidualization in the genesis of preeclampsia. *Placenta* 2017;60:119–29. 10.1016/j.placenta.2017.06.005 PMC5718949 28693893

[18] Rabaglino MB, Conrad KP. Evidence for shared molecular pathways of dysregulated decidualization in preeclampsia and endometrial disorders revealed by microarray data integration. *FASEB J* 2019;33:11682–95. 10.1096/fj.201900662R PMC6902722 31356122

[19] Tong J, Niu Y, Chen ZJ et al. Comparison of the transcriptional profile in the decidua of early-onset and late-onset pre-eclampsia. *J Obstet Gynaecol Res* 2020;46:1055–66. 10.1111/jog.14257 32281216

[20] Garrido-Gómez T, Castillo-Marco N, Cordero T et al. Decidualization resistance in the origin of preeclampsia. *Am J Obstet Gynecol* 2022;226:S886–94. 10.1016/j.ajog.2020.09.039 33007270

[21] Pelzer E, Gomez-Arango LF, Barrett HL et al. Review: maternal health and the placental microbiome. *Placenta* 2017;54:30–7. 10.1016/j.placenta.2016.12.003 28034467

[22] Beckers KF, Sones JL. Maternal microbiome and the hypertensive disorder of pregnancy, preeclampsia. *Am J Physiol Heart Circ Physiol* 2020;318:H1–10. 10.1152/ajpheart.00469.2019 31626558

[23] Zakis DR, Paulissen E, Kornete L et al. The evidence for placental microbiome and its composition in healthy pregnancies: a systematic review. *J Reprod Immunol* 2022;149:103455. 10.1016/j.jri.2021.103455 34883392

[24] Yoffe L, Kuperman AA, Isakov O et al. Assessing the involvement of the placental microbiome and virome in preeclampsia using non coding RNA sequencing. *J Perinat Med* 2021;49:1071–83. 10.1515/jpm-2021-0006 34114389

[25] Kalafat E, Thilaganathan B. Cardiovascular origins of preeclampsia. *Curr Opin Obstet Gynecol* 2017;29:383–9. 10.1097/GCO.0000000000000419 28961633

[26] Tyrmi JS, Kaartokallio T, Lokki AI et al. Genetic risk factors associated with preeclampsia and hypertensive disorders of pregnancy. *JAMA Cardiol* 2023;8:674–83. 10.1001/jamacardio.2023.1312 PMC10248811 37285119

[27] Sufriyana H, Wu YW, Su EC. Low- and high-level information analyses of transcriptome connecting endometrial-decidua-placental origin of preeclampsia subtypes: a preliminary study. *Pac Symp Biocomput* 2024;29:549–63. 10.1142/9789811286421_0042 38160306

[28] Sufriyana H, Wu YW, Su EC. Human-guided deep learning with ante-hoc explainability by convolutional network from non-image data for pregnancy prognostication. *Neural Netw* 2023;162:99–116. 10.1016/j.neunet.2023.02.020 36898257

[29] Bengio Y, Courville A, Vincent P. Representation learning: a review and new perspectives. *IEEE Trans Pattern Anal Mach Intell* 2013;35:1798–828. 10.1109/TPAMI.2013.50 23787338

[30] Witthöft M , Etiology/pathogenesis, In: Gellman MD, Turner JR (eds.), Encyclopedia of Behavioral Medicine. New York, NY: Springer, 2013, 716–7. 10.1007/978-1-4419-1005-9_16

[31] Hu M, Alkhairy S, Lee I et al. Evaluation of large language models for discovery of gene set function. *Nat Methods* 2025;22:82–91. 10.1038/s41592-024-02525-x PMC11725441 39609565

[32] Huang YA, Li YC, You ZH et al. Consensus representation of multiple cell-cell graphs from gene signaling pathways for cell type annotation. *BMC Biol* 2025;23:23. 10.1186/s12915-025-02128-8 PMC11756145 39849579

[33] Li YC, You HR, Huang YA. scGraphDap: integrating functional state pseudo-labels and graph structure learning for robust cell type annotation in tumor microenvironments. *IEEE J Biomed Health Inform* 2026;30:3184–97. 10.1109/JBHI.2025.3607687 40928910

