# Supplementary material for: Modeling distinct pathogenesis of early- and late-onset preeclampsia via hierarchical representation learning of interactome
Source: Brief Bioinform. 2026 Nov 13;27(6):bbag627. doi: 10.1093/bib/bbag627 (PMC13761213; doi:10.1093/bib/bbag627)
Supplement: Supplementary file 1 [file supp_rev3_bbag627.docx]

Box S1. Technical details on ontology inference, gene mapping, feature map subset, and DI-VNN fitting.

**Ontology inference**

PEA utilizing GO database, we identified GO terms in two phases. First, we selected GO terms of which genes were enriched by DEGs of each of the associated factors. Second, among the selected GO terms for each of the associated factors, we identified those of which genes were enriched by each gene set in the pathogenesis models. Before any phases, GO terms were excluded if the evidence codes were IEA and IGI, respectively for limiting only human-curated terms^1^ and acyclic graphs^2^ for the neural network architectures. We conducted each enrichment analysis separately among GO terms with 15–200, 201­–500, 501–2000 and >2000 genes, to avoid irrelevant stringency in multiple testing correction due to gene abundance.^1^ All the significantly enriched child terms were connected to the next ancestors up to the root if the parent term was not significantly enriched. We constructed a DAG using GO with only significantly enriched terms for each pathogenesis model.

For CliXO algorithm, we inferred ontologies using each gene set in the pathogenesis models. This algorithm finds maximal cliques in a gene network by progressive thresholding of the similarity scores (i.e., Pearson correlation coefficients). We added a root ontology to be the parent of the inferred ontologies at the most ancestor positions. In addition, we aligned the inferred ontologies with GO using network-extracted ontology (NeXO) algorithm for assisting the ontology interpretation. We considered an ontology were novel if it was not significantly aligned to any GO terms.

**Gene mapping**

For example, if we have 468 genes, then there are 468 pixels on the feature map. Different gene sets had different feature maps. However, all samples used the same feature map for each gene set, while the gene expression values were different among samples. We transformed the expression values of each gene by 1-bit stochastic gradient descent into 1 and -1, if they were respectively ≥ and < the average, resembling up- and down-regulated gene expression at individual level. Zero values were assigned to any coordinates in a feature map, that were unoccupied by any genes.

**Feature map subset**

For example, if an ontology included 2 of 468 genes, then there are only 2 pixels on the 468-gene, feature map that was subset by that ontology. Specifically, we assigned zero values for the remaining 466 genes for any samples. Each subset predicted an outcome; thus, we had multiple versions of an outcome prediction as many as the number of the inferred ontologies for each pathogenesis model.

**DI-VNN fitting**

Briefly, a pathogenesis model was trained as a single network consisting multiple gene subsets based on the inferred ontologies. The predicted probabilities of the root and non-root ontologies contributed the losses with weights of 1 and 0.3.

Box S2. Details on GSAI annotation.

**Late-onset preeclampsia (LOPE)**

*Third-trimester placenta under chorioamnionitis*

SLA was annotated by GSAI as a negative regulator of T-cell receptor signaling, which in turns, prevent overactive immune responses (Table S3). For ANKRD13A, it was annotated by GSAI to be involved in ubiquitination which regulates protein degradation (Table S3).

*Third-trimester placenta under HELLP syndrome*

GSAI annotated PDE5A as playing a role in (Table S3): (1) regulation of vascular tone and (2) regulation of neuronal signaling. We considered the former was more relevant in this study based on the tissue context, i.e., placenta. Downregulation of *PDE5A* may resemble its inhibition by sildenafil, as annotated by GSAI, i.e., enhancing nitric oxide on its vasodilation effect, hence, decreasing blood pressure and improving blood flow.

**Early-onset preeclampsia (EOPE)**

*Third-trimester placenta under HELLP syndrome*

The downregulations of the four genes (Table S4) lead to: (1) less mRNA processing and export and adipogenesis by ALKBH5; (2) less activation of RAC1 by SWAP70, which regulates actin cytoskeleton dynamics; (3) less dephosphorylation of MAP kinase by DUSP1, involved in cell proliferation, differentiation, and response to stress; and (4) less transcription of genes by KLF4, which play roles in specific processes involved in cell cycle regulation, apoptosis, and epithelial mesenchymal transition. Meanwhile, the downregulations of *GREM2* and *GPR146* respectively lead to: (1) less antagonistic effect on bone morphogenetic protein involved in organogenesis; and (2) less G-protein coupled receptor with unknown exact function. Respectively, the up- and downregulations of *PIP5K1B* and *EZR* lead to: (1) more phosphatidylinositol 4,5-bisphosphate, a critical component of the inner cell membrane involved in signal transduction and cytoskeleton organization; and (2) less linkage of link the actin cytoskeleton to the plasma membrane, involved in cell adhesion, migration, and signal transduction.

Box S3. Comparison to previous studies and research implication.

**Comparison to previous studies**

The primary outcome of this study was to identify biologically meaningful targets upstream of placental dysfunction that distinguish LOPE from EOPE. Rather than stopping at conventional gene set discovery using off-the-shelf methods, we refined and structured the gene sets into hierarchical representations using both knowledge-based and data-driven ontologies. This approach enabled deeper and more systematic interpretation of the gene sets to uncover empirically-validated biological targets to each PE subtype for further investigation.

Opposite gene regulations between chorioamnionitis and LOPE were also identified by our pathogenesis models. Chorioamnionitis frequently causes preterm delivery from 10.7% (*n*=53/497) to 94.4% (*n*=17/18) among pregnant women who delivered at 33-36- and 21-24-weeks’ gestation.^3^ Meanwhile, PE incidence was >1 per 1000 ongoing pregnancies approximately starting from 34 weeks’ gestation.^4^ However, no placental samples under chorioamnionitis were found from pregnant women with PE when we identified the gene sets of interest in this study.^5^ Lacking of co-existence between chorioamnionitis and LOPE is required to whether competing risks between both conditions are merely due to clinical decision on pregnancy termination or pathophysiological due to opposite gene regulations.

For HELLP syndrome, our pathogenesis models could not differ this condition from either LOPE or EOPE. Most of HELLP syndrome cases reported hypertension which always present in PE, leaving 10-20% without hypertension.^6^ Alternatively, HELLP syndrome shared similar phenotype with atypical hemolytic uremic syndrome, a condition due to complement gene mutations.^7^ Similar genetic factors of mothers may lead to common effect on complement system and placenta between HELLP syndrome and PE. Investigation on these genetic factors and biomarkers in non-placental organs may help to differ PE from HELLP syndrome.

Furthermore, although our pathogenesis models are demonstrated in PE, the methodology is broadly applicable to other diseases. Unliked conventional approaches that only examine associations between individual disease-related genes and phenotypes, DI-VNN method incorporates gene-gene relationships when predicting outcomes. In addition, our predictive modeling offers a scalable way to validate and refine disease pathogenesis models beyond PE.

Box S4. Step-by-step workflow for this study.

**Step-by-step workflow**

Step 1.	Retrieve mRNA microarray datasets from GEO and ArrayExpress (accession numbers in ^5^, Table S2 of ⁵). Preprocess with quantile normalization.

Step 2. Identify overlapping DEGs between PE subtypes and associated factors (endometrial maturation, placentation, chorioamnionitis, HELLP syndrome) → 19 gene sets (Tables S1–S2). Details in ^5^.

Step 3.	Infer ontologies: PEA via GO (exclude IEA/IGI codes, enrichment in 4 gene-count bins) and CliXO (progressive thresholding of Pearson correlations). Details in Box S1. → 28 DAGs.

Step 4.	Generate 3D feature maps using t-SNE (Barnes-Hut, perplexity per ^8^) on gene-gene similarity matrix. Binarize expression (≥ mean → 1, < mean → −1).

Step 5.	Train DI-VNN per ^8^ hyperparameters. Root/non-root loss weights: 1/0.3. Validate on independent test set.

Step 6.	Apply cascading validation: C1 (PPV-TPR or NPV-TNR LB >0) → C2 (≤25% dev-test difference) → C3 (grad-CAM >97.5th percentile) → C4 (AUC-ROC LB >0.5 both sets).

Step 7.	Annotate surviving gene sets via GSAI (GPT-4, ^9^). Domain expert review.

Source codes:

https://github.com/herdiantrisufriyana/pec (Steps 1–2) and https://github.com/herdiantrisufriyana/pec_hrl (Steps 3–7).

Table S1. Predictors in the pathogenesis models.

| Representation of | | Number of genes | | | |
| --- | --- | --- | --- | --- | --- |
|  |  | PE | | PE with FGR | |
|  |  | Late-onset,  severe | Early-onset,  severe | Late-onset,  mild-to-moderate | Late-onset,  severe |
| Endometrial maturation | Early-secretory phase | 0 | 468 | 37 | 360 |
|  | Mid-secretory phase | 72 | 0 | 0 | 0 |
|  | Late-secretory phase | 58 | 405 | 0 | 304 |
| Placentation | First trimester | 0 | 353 | 0 | 280 |
|  | Second trimester | 0 | 44 | 0 | 41 |
| Chorioamnionitis | Second trimester | 0 | 24 | 0 | 20 |
|  | Third trimester | 58 | 426 | 0 | 375 |
| HELLP syndrome | Third trimester | 52 | 283 | 0 | 189 |

FGR, fetal growth restriction; HELLP, hemolysis, elevated liver enzyme, and low platelet; PE, preeclampsia.

Table S2. Predictors in the pathogenesis models.

| Subtype | | Representation of | | HGNC symbols |
| --- | --- | --- | --- | --- |
| PE | Early-onset, severe | Endometrial maturation | Early-secretory phase | FBLIM1, PALLD, INPP5F, PPT1, OAZ1, HEXB, PDGFC, OLFML2B, HMMR, SPON2, TOP2B, DCP2, SCARB1, UBE2Q2, HTRA1, LSM6, MOB3C, ILF2, CKS2, SLC23A2, XPO4, TEX2, GOLM1, TBX3, SLC29A3, ENG, SETX, KDM1A, TES, GNG7, QPCT, PM20D2, AMOTL2, FPGS, FSCN1, SLC7A2, TYRO3, SPON1, DDHD1, TSPAN33, MGAT5, CREB1, AGO1, CNPY4, CIB2, CXXC1, SYK, SPG21, NRSN2, PSAP, LRRC42, RABEP1, DDR2, RNF24, HJURP, CBX5, MID1, YWHAG, CDK14, TMEM30A, CYB561A3, KLHL3, PROCR, CNN3, IRF8, PIP5KL1, CCND3, SLCO2A1, PRRX1, MIIP, PFKM, SHANK2, HMGB3, ZNF462, GPX7, GPX3, COQ2, CARD16, SH3PXD2A, DROSHA, DNAJB6, CD47, STX6, LHFPL2, NRCAM, VCP, FRZB, CYFIP2, SESN3, PMAIP1, MELK, EZH2, GNG4, TNFRSF10D, VEGFA, FBN2, RAD51, MAP4K4, TRIP13, INTS7, DNMT3A, EFNB1, TRIB2, ALDH1B1, MICU3, LATS2, BASP1, FANCE, ODF2, RPL15, TRPC6, GRAMD1A, SLC26A6, TRIM25, SPRED1, MX1, KLHL42, OSMR, LMAN2L, LAMC2, SH2B3, SLC8A1, IL33, PKM, OVGP1, IGSF3, DLGAP4, SYDE1, DPYSL3, CDC42EP1, THOP1, TMEM216, ARHGAP28, TMEM106B, SH3BP5, PPP2R3A, MXRA5, RPS6KA6, APLN, LAMA4, MBTPS2, NCEH1, HNRNPA0, DIO2, RUSC2, LYN, PGRMC1, DONSON, CTSV, ETNK2, PANX1, PAWR, SLC6A8, GNG11, SUMO3, MTPN, CEBPG, ROBO4, RAB5B, FEM1B, CAPRIN1, ZMYND11, PUS7, TXNRD1, NUDT5, PAK2, ACTR2, PLBD1, RASSF8, GNB1, ZEB2, PTPRD, KIFC3, GRINA, MX2, TAF9B, TNS3, CORO1A, PLCB2, LEPROTL1, ATXN10, CDO1, RGS10, RCBTB2, HMOX1, MED13L, OPTN, LRRC32, ITGA5, PKD2, PTTG1IP, NUFIP2, IFIT1, TNFAIP8, NRIP1, PROM1, ZNF512B, UNC119, SIPA1L1, MUC15, MAD2L2, RNF20, RIN2, CDC42BPA, GPER1, PHTF2, RIOK1, RSRC1, SIK3, SUV39H1, RAB6B, RAB8B, CTBS, ARHGDIB, PARM1, FBLN7, SHMT2, RANBP6, PAPPA, HDAC1, SSR1, OAS3, CLOCK, RNF114, CDC42BPB, FOLR1, MLLT1, CPEB4, ARID3A, ZNF84, GRHL1, ST6GALNAC1, GLA, PJA1, LRCH1, RAPH1, AKAP13, SAMD8, NFKB1, ATP1A1, MYO5A, HPCAL1, MME, HEXIM1, SNX18, MAOB, MEF2A, DCXR, SERPINB9, ABAT, CITED2, LRRC1, SLC44A3, KLF4, CEBPD, BRCA1, FOSL2, TRA2B, SLIT3, CLIC5, GCLM, OPRK1, PLPP2, FSTL3, MAP3K8, CALM1, SPAG4, TIMP3, TRIML2, EXOC6, SLC39A8, FAM210A, TMEM141, HDHD3, PRKAB2, GPR146, EPHB3, MIOS, MMD, TM9SF3, ARHGAP26, PLIN2, RERG, ZFR, ZDHHC9, MYO9A, APOL4, ERP44, NEK11, RNASE1, NFIL3, DKK3, TRIP4, FAM120AOS, CLMN, FAM107B, WWC1, RBM47, NPC1, BHLHE40, DUSP1, S100A6, PTPN3, CDS1, SGMS2, TLCD1, RNF19A, EPS15, WDR11, PHKA2, TACC1, ABCA1, RHOBTB3, CRYAB, EDN1, EFR3A, LURAP1L, ARTN, GPD1L, PAPSS1, CDYL2, TMCC3, SH3RF1, SBF2, C9orf72, CNOT8, SORT1, OCEL1, WDFY2, GADD45G, SLC17A5, WDFY3, PPA1, PLCB1, TSPAN12, LRIG3, G3BP2, DISP1, BCAT2, KLF3, FBXO8, FLNB, PLPP3, NFIA, EZR, ZNF217, ANGPT1, KIAA0232, ERCC6, GULP1, ANXA1, JADE1, DUSP4, ZNF143, ERRFI1, CITED4, SPTY2D1, TSPAN13, NUP153, PLOD2, VSNL1, TMEM154, GSN, PTPRJ, SEMA3G, PPP2R1B, SACM1L, FRMD4A, LEP, TANK, PRKCH, ARHGAP5, BTD, CANT1, AMOT, CYP2J2, NHLRC2, NECTIN4, HSPA2, UBALD2, IPPK, EAF1, TPD52L1, PLEKHA7, AVPI1, DPYD, P4HA1, TRIM52, PDZD2, GDA, ADCY7, CDKN1B, PGBD2, PTBP3, FZD1, MATN2, APOL2, ARRDC3, LRRC41, COL1A2, PTPN12, ELL2, SLCO4A1, FLVCR2, SAV1, MXI1, TMEM268, SLC24A3, GPT2, OSBPL11, SNAPC1, CYB5R1, GGCX, PPP1CB, SLC31A2, RASEF, IQGAP2, PPP3CA, ACVRL1, CX3CR1, PREP, ARL6IP5, SRPK2, HMGCR, CRISPLD2, MRI1, TLK1, PPL, MAFF, PIP5K1B, DSP, GOT1, SLC2A1, SWAP70, CCSAP, SLC35B3, NEK7, ERLEC1, KRT19, ESRP1, TGFBR3, SCAPER, EPS8L2, DDIT4, TIGD2, RALGPS1, HILPDA, BCAR3, ANKRD37, MXD1, ADK, ICA1, GOLGA5, TLR4, TSPAN31, PIK3CB, ANKRD13A, HEY1, PRRG4, DLX5, ZBTB20, MT1F, SNAP23, WDSUB1, NT5E, AMD1, VEGFC, C1orf21 |
|  |  |  | Late-secretory phase | ABCA1, ANKRD13A, ZFAND5, PANX1, RASEF, LAPTM5, RNF11, ACKR2, TBXAS1, ETS2, ARRDC3, BHLHE40, MTMR6, ARHGEF12, STRN3, ELF4, RAPH1, ENG, EPS8L2, RGS16, GRHL1, MBD2, OXSR1, CTSA, NCEH1, VEGFA, AKAP13, TMEM154, LDLR, JUNB, YPEL2, CDYL2, TES, MAFF, CEBPD, GALE, SMIM3, SH3BP5, LYN, CSF2RB, TSC22D1, GULP1, CDKN1B, GADD45G, CORO2A, GPX3, NEK7, PRRG4, MINPP1, MXD1, SLC1A1, FAM43A, ZNF217, CHIC2, PPP2R5A, SLC2A1, PIK3CG, ZNF281, PIK3AP1, SLC38A6, TMEM176B, SNAPC1, SPTY2D1, HILPDA, IFIT1, STAB1, NR4A3, ZFYVE1, TIMP3, MAP3K8, CORO1A, SYNJ2, SLC38A1, RFK, PRKCH, RNF19A, ERP44, PPL, PGBD2, LATS2, HTRA1, LRRC8A, PAPPA, TREM1, KPNA1, TYROBP, MXRA5, NUCB2, LCP1, GBE1, PGK1, CPEB4, LURAP1L, EAF1, DUSP4, FBXO9, TSPAN12, RBM47, OPTN, ANKRD37, VDR, FAM210A, SLC25A4, MXI1, TMEM216, DUSP1, SLC7A2, TEX2, PIK3CB, PLIN2, LPL, SAMD8, ITGA5, TSPAN13, KIAA0513, IGSF3, ST6GAL1, SPAG4, SH2B3, ANXA1, PPA1, HEY1, TGFBR1, POLR2H, TANK, ILF2, STAT4, LYPD3, WDSUB1, KDM1A, PREP, CX3CR1, TPP1, RNASE1, GNG4, SGMS2, THRA, TMEM30A, DDIT4, SEMA4B, CASD1, PTPN12, FKBP4, DOCK8, KLF4, THOP1, UBR1, EPS8L1, BCAR3, SPI1, TP53BP2, SWAP70, FLI1, C9orf72, TSPAN14, SUMO3, PDZD2, CTBS, TMCC3, EDN1, ZNF143, FNDC3A, SH3PXD2A, TRAK2, ALDH1B1, DLX5, MYO5A, GRAMD1A, SLCO2B1, CIB2, RHOBTB3, DNAJB9, COL1A2, ARHGDIB, GLA, SARAF, NFIL3, HDAC1, MRI1, CYFIP2, MED13L, WDFY3, SMPDL3A, DCXR, EPS15, MEF2A, EFHD1, QPCT, HNMT, AMPD3, CRISPLD2, KIF17, CDK9, SLC9A9, C3AR1, FBXO8, LSM6, GNG7, GPER1, USP3, AMD1, PTBP3, COL17A1, GLE1, FOSL2, ELL2, LRRC32, ZNF512B, SLCO4A1, COQ2, COL6A1, PHKA2, CRYAB, CLIC5, HPGDS, SLC31A2, LIN7A, LY86, UBALD2, TNFAIP8, CTSF, COPZ2, TOP2B, CTSV, RNF24, SLC17A5, ARHGAP28, LRRC41, RCBTB1, PROM1, SERPINB9, SLC16A3, MAOB, SPON1, ASTE1, NPC1, DONSON, NARF, TNFAIP8L1, FAM172A, BRCA1, CHEK1, SBF2, ELMO1, TSPAN3, GRINA, KIAA0232, ASAP2, TMEM106B, ODF2, KLF3, XPO4, CDO1, LPXN, SULF2, MYO1F, DNMT3A, CALM1, MTPN, HLF, SH3BGRL, CLIC3, HSPA2, WFS1, NRIP1, SESN3, PIK3R1, TBC1D5, FGF13, RIN2, TYRO3, RBM26, TRIM25, HEXB, ARHGAP24, MAP4K4, CDK20, CYBB, OVGP1, TAF9B, OSMR, SMARCA2, DPYD, SACM1L, PLOD2, SNAP23, DNASE2, STK17A, CASP4, IGSF8, SUV39H1, CNPY4, TTLL5, ERO1A, OAS3, GOSR1, HMGB3, TRIM24, EDEM1, PPM1M, RAB8B, ST6GALNAC1, CCND1, MAD2L2, SLC39A8, FZD1, BAD, MOB3C, CLCN5, C4orf3, RDX, CSF3R, PCYOX1, PUS7, DDX17, FBN2, PPT1, PJA1, PEBP1, IL33, TKFC, CBX5, SRPK2, FBLN7, PALLD, CDC42BPA, CNN3, FAM107B, DDR2, DEGS1, GEMIN5, PODXL, FBLIM1, AMOTL2, LHFPL2, MAML2, PDGFC, DKK3, MID1, NIPAL3, PCCB, ATXN10, HJURP, SHANK2, TOR1B, PKM, BCAT2, CCDC113, UNC119, MME, MELK, RAD51, HMMR, MICU3, COX8A, INPP5F, PLBD1, MX1, CPD, ZNF462, GREM2, RNF20, TPD52L1, MTA1, PTPRJ, CKS2, TRIP13, PIP5K1B, LMAN2L, PLCB1, IGF1, FRMD4A, AZIN1, ZFP3, FOLR1, UBE2K, INTS7, CITED2, HNRNPA0, MATN2, GPX7, HMGN3, ZMYND11, PAM, HMGCR, MBNL2, EIF4A2, RPL15, CCDC115, NRCAM, PIGX, GSTK1, PIP5KL1, TRA2B, EPHB3, PFKM, ZNF84, PGRMC1, SLC23A2, STXBP6, CLOCK, TRIB2, DROSHA, CXXC1, NFIA |
|  |  | Placenta | First trimester | CX3CR1, SLIT3, CEBPD, MYO5A, SLC2A1, GNG11, DNAJC1, AKAP13, SCARB1, ZBTB20, PTPN12, IGF1, KIAA0232, CBX5, DYRK2, ETS2, MEF2A, GPER1, ZNF462, IFI44L, CYTL1, GSN, PARM1, NFKB1, RAB3B, SEMA3G, ST6GAL1, MCTP2, SYK, NFIA, TXNDC11, SMIM3, LRRC8A, JAK2, CDK14, MS4A4A, LAPTM5, JUNB, F8, RIN2, CLIC5, CARD16, PRKCE, KIF5B, CCDC59, TBXAS1, DDIT4, GNB1, EXOC6, CCND1, TMTC4, SAV1, NECTIN4, PPP2R5A, KMT2E, FZD1, CYBB, DPYD, ELMO1, SNAPC1, C3AR1, TSPAN33, HMGN3, MIDN, LTF, RIMKLA, TRIP10, CLOCK, TACC1, FLI1, MBTPS2, MXI1, RNASE1, MAML2, SYPL1, AMOTL2, DDR2, ACTN1, TDP1, MIIP, TANK, ARRDC3, YPEL2, GALNT18, TYROBP, DUSP1, ZNF281, LAMA4, EFHD1, EDEM1, SBF2, TEX2, THOP1, PPP3CA, TLR7, THRA, LRRC32, HSPA2, PIK3AP1, TSPAN14, ZFYVE1, MRI1, CHIC2, MLLT1, COL6A1, SLC9A9, MINPP1, DIO2, SULF2, ASTE1, PLCB1, RNF11, CMKLR1, ZNF331, PODXL, SDC4, WDSUB1, HMOX1, PDGFC, SMPD2, SLC26A6, PGRMC1, SLC6A8, GUCA2A, TLR4, BCL2L11, MXRA5, MAP3K9, BCORL1, HK1, RCBTB2, ASAP2, RALGPS1, ADCY7, TMCC3, ANGPT1, ARL6IP5, RASAL2, ARTN, RASEF, ANKDD1A, AVL9, SYDE1, MATN2, ILF2, GCLM, CTBS, GOLM1, PROM1, PM20D2, EBF3, SH2B3, ATP10D, IL33, ERRFI1, HNMT, DKK3, ELL2, GRINA, PDE5A, MAP4K4, SESN3, NMNAT2, BRAF, INSL4, NIPAL3, TRIB2, ARID3A, KDM1A, TMEM106B, VEGFA, CNNM4, WDR11, MT1F, CREBL2, PKM, CDO1, NUDT5, HEY1, PEBP1, SLC38A1, LHFPL2, NCEH1, SEMA4C, ZMYND11, PLOD2, TRPC6, FANCE, VEGFC, PMAIP1, SHANK2, PFKFB4, ACVRL1, SLC35B3, GALK1, GBP4, EDN1, NUCB2, DHX29, ADARB1, OLFML2B, CPOX, PAPSS1, GPR107, HMGCR, MIOS, ACSF2, DLX5, CCDC113, GAN, TMEM176B, FCN3, ARHGAP24, CDS1, KIFC3, XPO4, LRCH1, PCYOX1, INTS7, AZIN1, SPG21, APLN, WNK2, G3BP2, FGF13, SLCO2B1, LIMCH1, AGTR1, FBN2, SARAF, CNOT6L, TRIP13, YTHDC2, ZNF512B, TMEM141, TAPT1, SACM1L, INSIG1, RNF20, BRCA1, ERLIN2, CADM3, AMD1, GLA, PUS7, CKS2, ROBO4, AK2, PRKAB2, BTD, HMMR, PLBD1, LEPROTL1, WDFY2, FLVCR2, PHKA2, PDP2, DCXR, SLC38A6, SLC39A8, NPL, SLC11A1, CTSA, PTPRJ, C1orf21, MME, DUSP4, MXD1, KLHL3, RCAN3, APH1B, FOLR1, DUS2, SLC44A3, TMEM43, PIK3CB, ABLIM3, GRHL1, DAB2, LDLR, SIPA1L1, SLC24A3, MAT2A, MKNK1, SLC16A3, GRHL3, EPS8L1, HGF, TP53BP2, LSM1, MOB3C, NHLRC2, CDYL2, KIF2A, MAN1A2, PAPPA, NPEPPS, INPP5F, NEK7, TLCD1, RASSF8, TMEM268, CSNK2A2, CCSAP, KIF17, ZNF75A, KLHL42, PKD2, NIN, PPP2R1B, HSPA9, GGCX, CYP19A1, GPT2, SH3RF1, EZH2, TKFC, LURAP1L, CHEK1, SPON1, SCMH1, PLAG1, STX6, EPB41L5, DONSON, IDE, PDZD2, GEMIN5, PRKCH, RAD51, MAFF, GPD1L, TOP2B, PREP, ARHGAP26, FNDC3A, STAT5B, CLTC, GADD45G, ODF2, VCP, RHOBTB3, TMEM154, DOCK8, MELK, SMARCA2, TTI1, MTMR4, ETNK2, ARHGAP28, ESRP1, HJURP, UBE2H |
|  |  |  | Second trimester | GADD45G, KIF2A, STAT5B, HEY1, TMTC4, SLC35F2, ENG, C1orf21, LIMCH1, CLIC5, CLMN, TAPT1, APLN, SCARB1, HMGN3, GPER1, PAM, EPS8L1, MAFF, VSNL1, PAPPA2, PRKCH, NECTIN4, SLC7A2, SLC24A3, RASAL2, CCSAP, NEK7, PLOD2, PAPPA, CDK19, ABLIM3, MTMR4, HJURP, ARHGAP26, SYDE1, DUSP4, ACSF2, MAN1A2, EFHD1, PLCXD2, HMGCR, GOLM1, RAB3B |
|  |  | Chorioamnionitis | Second trimester | PROCR, CCDC113, FSTL3, RASEF, CNNM4, NEK11, GPT2, LIMCH1, LPL, LEP, CX3CR1, SH3PXD2A, INHA, DLX5, LRRC1, SLCO2A1, KRT19, QPCT, PIK3AP1, ENG, SYDE1, VDR, FCN3, ERRFI1 |
|  |  |  | Third trimester | C4orf3, EAF1, LEP, OLFML2B, SUV39H1, TSPAN33, INHA, SEMA4C, PIK3R1, COL17A1, FKBP8, RNASE1, ZMYND8, CD4, TBXAS1, MXI1, SERPINB9, NFIA, FSTL3, HILPDA, ANKRD37, GREM2, NRIP1, MID1, UBB, CDO1, ITGA5, SIPA1L1, SYK, FAM20B, PLCB2, TMEM45A, DIO2, ARRDC3, RFK, P4HA1, SEC23A, RNF24, PMEL, HPCAL1, ERRFI1, COL6A1, COL1A2, RAPH1, DNAJC1, SPON1, GBE1, KCTD12, BTD, GPT2, SLC11A2, TREM1, CEBPA, PDGFC, AMD1, MFAP5, CXXC1, BHLHE40, VCP, TRIM24, RASSF8, BCL2L11, DISP1, VDR, PPA1, KMT2E, ZC2HC1A, SLCO2A1, MS4A4A, CCDC127, SYNJ2, MAP3K8, ERO1A, SLC2A1, MGAT5, GALK1, DCP2, EBI3, PROM1, GDPD3, TMEM107, MAP4K4, RGS10, INPP5B, TSC22D1, GNB1, DPYSL3, PTBP3, TNFAIP8L1, AVL9, DSP, GSTK1, IPPK, BRCA1, KIAA0232, MKNK1, TMX1, APOL4, KIF2A, PAM, CYB5R1, PUS7, CNNM4, LPL, DOCK8, TNFAIP8, PFKM, TANK, HMMR, CTSZ, PAWR, CASP4, CHEK1, SDC4, NUFIP2, FBN2, ENG, HMGCR, INTS7, UBE2H, KLF4, SDF2L1, GPR146, CORO1A, F8, FSCN1, VEGFC, ARTN, FBLIM1, RAP1GAP, FAM107B, EDN1, ERP44, OSBPL11, UBR1, SLC35F2, GADD45G, PODXL, ALKBH5, SLIT3, GFOD2, AMOTL2, COQ2, PPP2R5A, TMEM43, ZNF175, CCND1, QPCT, PJA1, TACC1, TSPAN3, OCEL1, LAPTM5, CALM1, YPEL2, RIOK1, ELMO1, ABCA1, MAT2A, KLHL8, MATN2, LRRC32, RANBP2, SMPD2, GSN, NECTIN4, IFIT1, NARF, SHANK2, STXBP6, IFI44L, SHMT2, SLC11A1, ICA1, GBP4, CADM3, LTF, ARID3A, FOXJ2, PTPRD, TSPAN13, RBM22, TMEM141, SYNGR1, SPRED1, MX2, CLCN5, FBLN7, LURAP1L, PIK3CG, PALLD, SLCO4A1, ST6GAL1, PEBP1, ARHGAP28, ALDH1B1, STAT5B, BCAR3, LDLR, PAK2, SMPDL3A, NEK11, GPX7, TYROBP, ARMCX1, TBC1D9, TTLL5, CYTL1, PIK3CB, PKD2, TES, RBM47, SDF2, TDP1, EPS8L2, SLC30A1, CKS2, ILVBL, ASTE1, CCSAP, SIGLEC6, APLN, KLHL42, ILF2, OVGP1, PGBD2, FGF13, MTMR4, AMOT, ROBO4, SNX18, CNN3, SPAG4, WDFY2, SBF2, PRRG4, INSIG1, SARAF, HJURP, STAB1, GPD1L, SLC24A3, RAD51, KDM1A, PLAG1, LRRC42, LMAN2L, JUNB, SLC6A8, FLI1, ST6GALNAC1, GOT1, RBBP4, CDS1, LEPROTL1, PRRX1, MB21D2, PMAIP1, CHMP2B, ANXA1, GDA, PLPP3, SPTY2D1, PDZD2, ASAH1, COPZ2, TRIP13, ARHGDIB, TMEM30A, SMARCA2, MELK, IGSF3, ACVRL1, ADCY7, C9orf72, SSR1, CBX5, GPER1, SGMS2, ZBED5, SH3BGRL, CEBPG, FAP, TLCD1, SLC17A5, EPB41L5, FAM120AOS, ATXN10, ANXA4, SEC22C, KIF17, ZNF555, TNFRSF10D, CDC42BPA, RAB6B, ATAD1, BCL2, PIGX, STK24, DNMT3A, SEMA3G, SLC38A6, S100A6, RNF11, CX3CR1, ACTR2, ZEB2, VWCE, ACTN1, ZNF79, STAT4, RNF20, CLTC, SFXN3, GOLGA5, SLC39A8, PLIN2, ERLIN2, MRPS30, GNG11, NPL, RASEF, NPC1, SH3BP5, C3AR1, EZH2, XPO4, SCMH1, WFS1, MYO5A, ACVR2B, ZFAND5, GAN, LY86, PAPPA2, AKAP13, LRRC1, MIDN, FANCE, HDHD3, PRKCH, GNG7, ZNF143, SLC9A9, CALM2, CDK19, NRSN2, SLC7A2, EPS15, GRINA, CD28, LIMCH1, HPGDS, SPI1, CSF3R, GRAMD1A, CREB1, SLC28A1, MBD2, FOXJ3, EXOC6, PRKCD, RERG, CLIC3, VSNL1, CYBB, YTHDC2, MYO1F, HGF, MOB3A, HMGB3, RCBTB2, DLX5, CORO2A, WDR11, GRHL3, LYN, HSPA9, RPL15, MCTP2, TLR7, CMKLR1, IDE, DUSP1, SLC16A3, TRAK2, IGF1, SH3PXD2A, ANKRD13A, CCDC115, GRHL1, BAD, SAR1A, PIK3AP1, HTRA1, MAFF, RUSC2, MED13L, PROCR, RIMKLA, HNMT, MAML2, CARD16, PPP1R12C, HEY1, SLCO2B1, GUCA2A, EZR, CCDC113, EFHD1, MXD1, EFNB1, GALE, UBE2Q2, BCAT2, SYDE1, EPS8L1, KRT19, PPL, SCARB1, TRIP10, LRRC8A, ACKR2, CSNK2A2, MAP3K9, P2RY2 |
|  |  | HELLP syndrome | Third trimester | GPR146, SUV39H1, PIK3CB, CORO2A, BASP1, DLX5, SFXN3, SLC6A8, ZMYND8, SEMA4C, LRRC8A, CNNM4, HEY1, SEC23A, MIDN, EAF1, ERRFI1, PFKFB4, SLC2A1, VWCE, ANKRD37, ITGA5, GALK1, FANCE, MME, PPL, SWAP70, CYP2J2, MOB3A, SEMA4B, FBLIM1, ALKBH5, EFNB1, TSC22D1, KRT19, GUCA2A, C4orf3, SPAG4, SLC16A3, NECTIN4, TPP1, BCAT2, EPS8L2, GREM2, KLF4, DUSP1, DNAJC1, PIK3R1, SMIM3, GLA, NPEPPS, EPHB3, CSNK2A2, SIPA1L1, TOR1B, CLIC3, WFS1, CEBPA, PGRMC1, PLIN2, MYOF, TMEM45A, PLOD2, KLF3, EPS8L1, IGSF8, GRHL1, CXXC1, ENG, ARID3A, FLNB, TIMP3, PPP1R12C, AFMID, PIP5KL1, ELF4, FSTL3, INPP5B, BCORL1, APH1B, INHA, MAP3K9, SYDE1, PMEL, SHANK2, TREM1, IL36RN, HK1, FCN3, AMOT, QPCT, GSN, CCDC127, IPPK, ZNF331, NIM1K, GRHL3, HSPA9, GDPD3, P2RY2, ZDHHC9, MAML2, PIGX, ACKR2, WNK2, CDO1, TM9SF3, P4HA1, PREP, CYB5R1, SLC26A6, PRKAB2, MAP3K8, VEGFA, OVGP1, SLC31A2, TSPAN12, GCLM, PLCXD2, EFHD1, OCEL1, ZFAT, SCARB1, BHLHE40, FOSL2, SLC25A4, JUNB, LRRFIP2, HMOX1, KIF5B, SDC4, BAD, DPP7, WDFY2, FPGS, PRKCH, CLTC, CDK9, TMEM107, DONSON, ERO1A, NEK11, KIT, SPG21, GPT2, LDLR, HPCAL1, RPL15, TRIP10, ZC2HC1A, ABCA1, KMT2E, PHTF2, DIO2, PODXL, MBNL2, GORASP2, MXD1, STK24, CRISPLD2, NUFIP2, ZNF143, VEGFC, RSU1, IQGAP2, SUMO3, CREB1, FRZB, BCAR3, EBI3, NOD1, ASTE1, HMGB3, TXNRD1, SH3BP5, IGSF3, RASEF, MOB3C, TAF9B, PFKM, SH3PXD2A, PAWR, BTD, FKBP8, MTMR6, PIK3AP1, IGF1, PGBD2, MUC15, IDE, CCND1, MIIP, MAP4K4, PCCB, SH3RF1, FRMD4A, TLE6, CLDN16, PKD2, GGCX, MAT2A, NFIL3, ANXA1, NMNAT2, RNF20, ASAH1, CEBPD, C9orf72, GPX3, HSPA2, PRKAR1A, DYRK2, LYN, ARRDC3, MRS2, FEM1B, ERCC6, PDE5A, IL33, FBXO8, PJA1, SPRED1, PIP5K1B, APLN, GBE1, BTBD10, LEP, TSPAN3, ILVBL, CYB561A3, EXOC6, PAM, GALE, ZNF462, DDR2, STXBP6, PKM, HTRA1, LIMCH1, ARHGAP5, FAM43A, ADK, ATXN10, HPGDS, CALM1, ZFR, FAM20B, TRA2B, PAPPA2, CASD1, WDR11, RANBP6, SACM1L, CYP19A1, SLC23A2, UBE2Q2, HNMT, ZEB2, TRIM24, MXRA5, AZIN1, RBBP4, CCSAP, CD28, SPON1, PROCR, LRRC1, TLR7, UBB, KLHL42, ZNF555, CARD16, ZNF75A, AK2, NHLRC2, ZMYM4, VWA5A, PLPP3, MRPS30, FBN2, ZMYND11, CYBB, EZR |
| PE | Late-onset, severe | Endometrial maturation | Mid-secretory phase | ICA1, PLIN2, BMP2, DOCK8, GDA, NFIL3, RDX, CCDC127, CORO2A, BCAR3, SPAG4, FAM172A, ANKRD37, PKM, CLIP1, TUBB4B, AZIN1, ANKRD13A, PDE5A, IGF1, TUFT1, KRT19, INPP5F, EFNB1, SMPDL3A, PTPRD, NPR3, P2RY14, CTBS, SH3PXD2A, GRHL1, CDO1, LDLR, MYC, ASAH1, MRPL34, ZNF555, ELOVL5, APOLD1, FAM43A, TIAM1, BBS4, MCAT, DYNLL1, SCYL3, GPT2, SLCO2A1, SYDE1, HYAL2, TOR1B, PIK3R1, ERO1A, APPBP2, EDEM1, FAP, NECTIN4, DDIT4, NEDD4L, APLN, KLF3, GMFB, SLCO4A1, P4HA1, ERRFI1, TRIM68, ETS2, CEBPD, GULP1, RAPGEF3, FLNB, GPX3, DEGS1 |
|  |  |  | Late-secretory phase | MRPL34, DDIT4, ERO1A, KLF3, RDX, NFIL3, AZIN1, PKM, TUBB4B, SLCO4A1, VEGFA, BCAR3, SMPDL3A, CDO1, TUFT1, TMEM106B, CTBS, IGSF8, GULP1, GPX3, GBE1, EDEM1, SPON1, SPAG4, CCDC6, SLC25A4, PLIN2, NIPAL3, MYC, PREP, IGF1, INPP5F, FAM172A, DOCK8, MCAT, BMP2, PFKFB3, FAM43A, TOR1B, NFKBIA, ANKRD13A, CORO2A, SH3PXD2A, LDLR, BHLHE40, SLA, PIK3R1, TRIM68, ETS2, ANKRD37, GRHL1, P2RY14, TIAM1, DEGS1, ELOVL5, CLIP1, DYNLL1, CEBPD |
|  |  | Chorioamnionitis | Third trimester | CDO1, PLIN2, CCSAP, BCAR3, DOCK8, SPAG4, BHLHE40, NECTIN4, SIGLEC6, PTPRD, SFXN3, LDLR, CORO2A, CCDC6, TACC1, SELL, NEDD4L, SLCO4A1, SPON1, PFKFB3, GDA, APLN, IGF1, PIK3R1, SLC11A1, SLA, KRT19, SYDE1, ERRFI1, TUBB4B, SMPDL3A, GRHL1, PAK2, RBM22, CEBPA, ANKRD13A, GBE1, FAP, GPR146, HINT3, CCDC127, ZNF555, ICA1, CHMP2B, MYC, CA4, LEP, EFNB1, P4HA1, SH3PXD2A, ZMYND8, ASAH1, RAPGEF3, SLCO2A1, ZEB2, GPT2, ERO1A, ANKRD37 |
|  |  | HELLP syndrome | Third trimester | SFXN3, GBE1, ASAH1, KLF3, GPT2, LEP, SH3PXD2A, PKM, GPX3, FLNB, IGF1, CEBPD, ERRFI1, CCDC127, PDE5A, IGSF8, CDO1, CCSAP, SYDE1, ERO1A, SLC25A4, TOR1B, FCN3, BCAR3, NFIL3, LDLR, EFNB1, VEGFA, GRHL1, KRT19, ZEB2, GMFB, APLN, AZIN1, ZMYND8, FAM43A, NHLRC2, GPR146, NECTIN4, PIK3R1, SPON1, SPAG4, RAPGEF3, IQGAP2, BHLHE40, CEBPA, PREP, CORO2A, ANKRD37, ZNF555, P4HA1, PLIN2 |
| PE with FGR | Late-onset, mild-to-moderate | Endometrial maturation | Early-secretory phase | MID1, MT1F, TIMP1, ANKRD37, PKM, ACVR1, ISG15, IFIT1, TP53INP1, HILPDA, CITED2, TM9SF3, TMEM106B, SFRP1, FHL1, RPS6KA6, POGK, SGMS2, KRT17, TIPARP, TMSB10, PTPRE, PARP12, BPHL, ARL6IP5, STARD8, CLOCK, FAM107B, PPP1R14C, LAMC2, KRT19, CHI3L1, PTGFRN, CLIC5, TLR3, SPAG4, PAPSS1 |
| PE with FGR | Late-onset, severe | Endometrial maturation | Early-secretory phase | CDH11, PJA1, DESI2, IFITM3, MMD, PMP22, PKD2, LTBP1, AK4, CHMP2A, CHSY1, SEPHS1, SIK3, STX6, PMAIP1, HPCAL1, FBN2, RASSF8, FBLN5, MIIP, VIM, LEPROTL1, MEIS1, C4orf19, PDS5B, APBB2, FXYD5, SESN3, PROCR, RPS6KA5, ATP7B, QPCT, MTFMT, SOWAHC, SYDE1, HEY1, SSR1, NFKB1, DIO2, PROM1, SPON1, PDGFC, LSM6, CORO1C, RABEP1, GTDC1, RNF20, PALLD, PARVA, PRRX1, HMGB3, LYN, INHBB, APLN, RAB8B, FHL2, MME, PAWR, THOP1, ESYT2, ARID3A, SERPINE1, NCEH1, INPP5F, CEBPG, SHANK2, TBX3, SPAG4, CH25H, UBE2Q2, GYPC, TPM2, SLC6A8, MARCKS, CDYL2, LRRC1, PKP2, SH3BP5, OPN3, ZDHHC14, ING3, SYK, MGAT5, RCBTB2, KLHL3, PKNOX1, DSTYK, FSTL3, NFIA, PARM1, SNX18, ATP1A1, MYO5A, LAMA4, PPP2R5E, SEMA3G, EPHB3, CDO1, SLC36A1, OLFML2B, ZBTB2, CORO1A, ARHGDIB, NPR3, PRKAB2, SLC25A19, DAB2IP, PHTF2, WNT5A, TSC22D2, STX1A, TAF9B, MMP2, RIOK1, TNFRSF10B, CREB1, DDIT4, HECW2, RNF130, ZNF614, PHYHIPL, CYFIP2, BHLHE40, USP46, PPP2R3A, TNFRSF10D, NFIB, GULP1, CST3, EFNB1, GOT1, CALM1, MAD2L2, GIMAP4, CRY1, ZNF350, SPRY1, TNFAIP8, CPEB4, ABAT, NDUFB9, GRINA, CDC42BPA, COG2, PTPRD, ARSA, RNF19A, DLG3, ADM, CLDN3, STX12, MFGE8, RDH10, GORAB, GPR146, SPRED1, SEC61B, BASP1, RNF114, DUSP1, FAM107B, RHEB, AKAP13, DDA1, ABHD5, DDHD1, PKM, PTPRJ, TRIM25, VEGFA, EPB41L4B, SLC8B1, GRHL1, SUSD1, ARRDC3, SUV39H1, SLCO2A1, CLIC5, EZR, SH3RF1, KLF3, PXK, LRRC42, IQGAP2, IGSF3, PLEKHF2, DDR2, GPER1, DYNLL1, RNF24, SH3PXD2A, OPRK1, ANGPTL2, ZEB2, AASDHPPT, C1orf21, UBALD2, RARRES1, HTRA1, NEDD4L, GPX3, SLCO4A1, PREP, EVI5, MFSD1, NUFIP2, MID1, WDSUB1, CIB2, DSP, CALM3, BCAR3, SLIT3, ERCC6, CCND3, KITLG, NECTIN4, LRIG3, PTBP3, CYBRD1, REV3L, NRIP1, EFNA1, KRT19, FPGS, APOL4, SLC23A2, ACAA2, CALCOCO1, TACC2, DOCK5, TSPAN31, PHF19, MOB3C, DNMT3A, PGBD2, RERG, ARL6IP5, MATN2, GNAQ, FLNB, EAF1, UROS, TMCC3, MBIP, TBL1XR1, ARRDC4, NCAPD2, FAM120AOS, CITED2, DCP2, LEP, SLC44A3, RUNX1T1, LURAP1L, SNAPC1, NAXD, SNAI2, BPGM, DONSON, MORC3, FSCN1, NEK11, LRRC41, AHNAK, PFKFB3, BGN, S100A6, WDR11, TXNRD1, SQLE, SOCS2, RASEF, MXI1, IGFBP4, PLPP3, PDE9A, ENG, RNASE1, DUSP4, HNRNPA0, SLC17A5, BTD, CX3CR1, TM4SF1, ICA1, TXNIP, GCLM, FAM210A, CA2, MYO9A, ERRFI1, PIK3CB, ANGPT1, SERPINB9, IFT20, ZC3H6, CYB561A3, TIMP4, MAPRE2, TBC1D4, PRKCH, CA4, CYP2J2, PRRG4, TM9SF3, FOSL2, CTBS, CRYAB, ESRP1, LAMB1, COL1A2, CEP68, P4HA1, ACTG2, SLC2A1, MSH6, TLR4, SWAP70, ADAMTS5, EIF4G2, SCARB1, SGK1, PLOD2, HILPDA, VPS25, TGFBR3, DISP1, SERTAD2, PLIN2, DLX5, PAPPA, ADCY7, CNOT8, FRMD4A, NRBF2, RIN2, NFIL3, CMYA5, ERP44, FBXO28, MAP3K8, CCSAP, BCAT2, GNG11, SLC12A6, GPT2, PPP1CB, RAPH1, PDGFRA, TPD52L1, VEGFC, MPC1, TACC1, ANKRD37, MAOA, SEC11C, SLC30A2, PPL, BTAF1 |
|  |  |  | Late-secretory phase | VPS45, GSTK1, COL6A1, SERPINB9, PALMD, FXYD5, MATN2, CCDC113, PTPRJ, RDH10, SUV39H1, GBE1, KLF3, SARAF, CLCN5, GNAQ, PDGFRA, RUNX1T1, LSM6, TNFAIP8L1, DNMT3A, REV3L, ZFP3, DUSP1, SWAP70, DIAPH2, ACKR2, SHANK2, FAM107B, CITED2, C3AR1, TM4SF1, MORC3, RNF20, NVL, EFNA1, RASEF, COIL, INHBB, RGS16, RNF19A, LRRC41, ZNF75D, FGD6, SH3BP5, UBALD2, TRIM24, RIN2, TMEM176B, IVD, CD163, JMY, TBXAS1, ELL, ASAP2, CTBS, CASP4, UBR1, WWTR1, NCAPD2, TPM2, IGSF3, LYN, AMPD3, TMCC3, SMARCA2, NR4A3, DYNLL1, SQLE, IRF2BPL, PTBP3, CRY1, CYBRD1, NFIL3, TRIM25, DEGS1, SLC25A4, WDSUB1, PAM, CDC42BPA, STK17A, GRHL1, CLIC3, TNFAIP8, TAF9B, MFSD1, BTAF1, ABTB2, IGSF8, GRINA, SLC23A2, QPCT, DNAJB9, CDO1, UROS, DDA1, PAPPA, GORAB, CALM1, COL17A1, RNASE1, EPS8L1, PKNOX1, ELF4, RFK, TSC22D2, PROM1, ZFAND5, NCEH1, ZFP36L1, SLC25A19, SH3BGRL, NR2F1, MAML2, HMGN3, SLC17A5, SESN3, ANTXR2, PKM, DIP2B, NBAS, ARHGEF12, MME, BHLHE40, RARRES1, ERO1A, NRBF2, SLC16A3, MAD2L2, AHNAK, HTRA1, TIMMDC1, ASTE1, TSC22D1, EAF1, HNRNPA0, ELMO1, ST6GAL1, CYBB, INPP5F, HECW2, WFS1, CORO2A, CALD1, GPER1, FNDC3A, TMEM140, RNF24, FHL2, GULP1, KIF17, LTBP1, AZIN1, PHYHIPL, CIB2, C1QC, PLIN2, CYFIP2, NFIA, PPP2R5A, SPON1, VIM, DONSON, KAT14, PKP2, IGFBP4, SEC11C, FAM43A, SLC8B1, PDGFC, HPGDS, ESYT2, VEGFA, HEY1, EPHB3, MTMR6, CORO1C, RPS6KA5, COPZ2, C4orf3, ZNF614, SEPHS1, MAOA, ADM, TTLL5, ENG, SGCB, MMP2, DCN, TACSTD2, SOAT1, C1QB, UPP1, LAMB1, SH2D4A, FRMD4A, SYNJ2, SLC9A9, PIK3R1, CA2, DLX5, DDR2, IGFBP5, PJA1, MANBA, SLC2A1, LURAP1L, PGBD2, NDUFA4, SERPINE1, CDYL2, MSH6, PCM1, THOP1, CALM3, ABHD5, TPD52L1, SLCO2B1, G6PC3, FBLN5, GATA3, SOWAHC, BAD, SPAG4, IGF1, SLC12A6, MOCOS, MOB3C, NAXD, HILPDA, MREG, CRYAB, BCAT2, RDX, BCAR3, CX3CR1, TREM1, SERPINB2, NRIP1, DUSP4, SLC30A2, PALLD, ZDHHC14, GREM2, SH3PXD2A, PIGX, RAB8B, STAB1, CHSY1, PDS5B, DSTYK, IFITM3, CLIC5, SLCO4A1, ERLIN1, EFHD1, PMP22, MXI1, ARHGDIB, PPL, GPX3, HMGB3, FAM172A, FAM210A, NDUFB9, PRRG4, SEMA4B, GALNT10, MID1, COL1A2, ARRDC3, FBN2, AK4, GIMAP4, ANKRD37, AKAP13, ERP44, DDIT4, CFL2, RAPH1, PREP, LRRC8A, MYO5A, SNAPC1, PFKFB3, CORO1A, SLC1A1, BPGM, PIK3CB, BAZ1A, PIK3CG, SGK1, F3, PLOD2, TYROBP, MAP3K8, GLUL, FOSL2, PIK3AP1, ADAMTS5, PRKCH, LCP1, CPEB4, TBC1D15 |
|  |  | Placenta | First trimester | C1orf21, UPP1, AZIN1, ATP7B, CCDC113, DIP2B, MLLT10, ACSF2, ARL6IP5, SH2D4A, PIK3AP1, TLR4, SLC30A2, MME, CDO1, HIC2, PRKCA, MAN1A2, SLC36A1, CH25H, NRBF2, CCSAP, ANKDD1A, PFKFB4, TRMT6, USP46, WDSUB1, WEE1, SNAPC1, NPEPPS, TACSTD2, CTBS, TPM2, HECW2, GNG11, UROS, KLHL3, NCEH1, NEDD4L, BTAF1, TACC1, SLC44A3, SPON1, DDR2, STAT5B, THOP1, IFITM3, GRHL1, INSL4, ARRDC3, IGFBP4, SEMA3G, VEGFA, EPS8L1, GMFB, SOWAHC, NFIA, SLC11A1, GCLM, DOCK5, CYBB, TMEM140, MYO5A, DYRK2, KAT14, F8, ADCY7, SEMA4C, AKAP13, STX6, ANGPTL2, C3AR1, RCBTB2, PRKCE, SLC2A1, GRHL3, PLOD2, INPP5F, MAN1C1, PRKCH, RBM43, DLX5, MAP3K9, PREP, ZNF331, RASSF8, FNDC3A, FBN2, SLC35F6, DUS2, SQLE, TBC1D15, ZNF75A, SLCO2B1, SLC8B1, GNAQ, DDIT4, SH3RF1, CMKLR1, PKM, ACO1, INHBB, GATA3, SLIT3, BGN, RNF146, OLFML2B, TDP1, RNF130, RHEB, MSH6, ANGPT1, TIMP4, APLN, PROM1, NPL, PPP2R5E, KRT4, UBE2H, SLC6A8, PMAIP1, KIAA0040, MATN2, ESRP1, HK1, PDP2, UTP15, CMYA5, ARRDC4, CALM3, RDH10, MCTP2, SCARB1, NFIB, ABTB2, JAK2, LRRC8A, TTI1, CA8, ERRFI1, GALK1, BRAF, TSC22D2, NECTIN4, CA4, KIF2A, PDE9A, MTMR4, PARM1, CSNK2A2, ING3, EIF4G2, EPB41L4B, MKNK1, VEGFC, MFGE8, MIIP, PCM1, SLC16A3, LIG3, DUSP4, WDR11, TM4SF1, PKD2, SNAI2, AGTR1, INSIG1, SESN3, HMGN3, PPP2R5A, RLF, MREG, GSE1, LEPROTL1, NPR3, SOCS2, GRINA, RANBP10, AP5B1, PDGFC, COL6A1, CDH11, SYDE1, NGLY1, MMP2, RAB3B, RASEF, BTD, C1QB, KIF17, IRF2BPL, MXI1, IRX2, SARAF, IGFBP5, TSPAN5, KIF5B, TMCC3, YTHDC2, LURAP1L, SLC6A4, CLIC5, ASTE1, EFHD1, KCNK5, BCORL1, AASDHPPT, ANTXR2, NR2F1, RARRES1, MOB3C, TMTC4, RIN2, PALMD, LIMCH1, RIMKLA, AVL9, CHSY1, ELMO1, C4orf19, PAPPA, VIM, ST6GAL1, SMARCA2, DIO2, HEY1, ARID3A, ZFP36L1, PIK3CB, RNF20, PRKAB2, SHANK2, ASAP2, ACTG2, KITLG, PDE5A, LAMA4, NFKB1, CD200, ADARB1, LTF, CDYL2, GALNT18, DAB2, PLA2G5, IGF1, KMT2E, TMEM176B, TLR7, CADM3, PTPRJ, CPOX, GPT2, DONSON, SPRY1, WWTR1, SHE, GIMAP4, COIL, TBC1D4, SLC9A9, TYROBP, TRIP10, WNK2, GAN, SYK, RNASE1, C1QC, RRAS, TBXAS1, TXNIP, CLDN5, MAPRE2, RPS6KA5, MAML2, PFKFB3, GPER1, CX3CR1, DUSP1, WWC3 |
|  |  | Placenta | Second trimester | SYDE1, RAB3B, SCARB1, CDK19, DUSP4, NECTIN4, SLC35F2, PAPPA2, APLN, CLIC5, TMEM140, EFHD1, ENG, PLCXD2, PLOD2, MAN1A2, C1orf21, MAN1C1, SERPINE1, GPER1, LIMCH1, TIMP4, CD200, PAM, KIF2A, PAPPA, UROS, HEY1, CCSAP, ACSF2, SOWAHC, PRKCA, EPS8L1, STAT5B, SNAI2, CDH11, C4orf19, HMGN3, TMTC4, PRKCH, MTMR4 |
|  |  | Chorioamnionitis | Second trimester | ERRFI1, LIMCH1, CX3CR1, QPCT, LRRC1, RASEF, INHA, GPT2, DLX5, PROCR, FSTL3, PIK3AP1, NEK11, CCDC113, SH3PXD2A, SLCO2A1, LEP, KRT19, SYDE1, ENG |
|  |  |  | Third trimester | MAP3K9, SLC16A3, PPP1R12C, IRF2BPL, CLDN5, ACKR2, TSC22D1, FAM219A, DAB2IP, PGBD2, GBE1, CCDC113, C4orf3, GALK1, TSC22D2, BCAT2, RPS6KA5, PLEC, STX1A, EPS8L1, SLC35F2, SMAD2, UBR1, MAN1C1, RIOK1, CCDC47, UBE2Q2, GSE1, HIC2, RLF, ELL, RANBP10, MARCKS, PURB, FOXJ2, RIMKLA, BTD, DESI2, AK4, ARRDC3, GATA3, CA4, ZNF350, CLIC3, FGD6, RHEB, S100A6, NVL, SLC6A8, DIO2, PPP2R5E, PJA1, BAD, ARID3A, DSP, TNFRSF10B, CRKL, MOB3A, PDS5B, LEPROTL1, SCARB1, ZC3H6, TACC1, PPL, P4HA1, PIK3R1, GPR146, ANXA4, ASTE1, SLC28A1, PFKFB3, KIF17, CDK19, CLDN3, MAML2, TMEM45A, TRIP10, SARAF, LRRC8A, SHANK2, P2RY2, FBXO28, SEMA4C, ESYT2, PROCR, PCM1, MORC3, SP1, TRIM24, CSNK2A2, SYNJ2, ANKRD37, TNFRSF10D, IGSF3, LTBP1, PAM, ENG, ZNF175, BTAF1, MID1, GAN, SYN3, RARRES1, SSR1, STK24, CRY1, CAPN7, ERP44, SOWAHC, QPCT, PPP2R5A, SNX18, EVI5, CEBPG, CLCN5, CMYA5, SLC11A2, GPER1, SEMA3G, MSH6, PRRG4, RNF24, PAPPA2, SLC17A5, VIM, VEGFC, WDR11, FSTL3, HPCAL1, FBLN5, NRIP1, GYPC, SLCO2A1, SIGLEC6, F3, PMP22, STAB1, GREM2, COPZ2, ERRFI1, MRPS30, INHA, PIGX, ZC2HC1A, PKNOX1, CALD1, SERTAD2, PKP2, SUV39H1, RBM22, FBN2, TREM1, IFITM3, CD4, ENY2, PHF19, EFHD1, HEY1, APBB2, ZMYND8, SLC11A1, SYK, LTF, ZNF555, SPAG4, CORO1A, GRHL3, TACC2, PARVA, MAP3K8, LAMB1, ERLIN1, AVL9, MANBA, ALKBH5, CX3CR1, HILPDA, SH3BP5, C1QC, COL17A1, TNFAIP8L1, MTMR4, KIF2A, PDE9A, F8, CALM3, SLC30A2, FAM107B, FAM20B, ERO1A, EAF1, BCAR3, PAWR, SQLE, SLCO2B1, MKNK1, FOXJ3, SLC6A4, CD163, ATP7B, TPM2, SGK1, SNAI2, SYDE1, WWTR1, COL6A1, MXI1, TTLL5, NDUFA4, TNFAIP8, ADAMTS5, SDF2L1, ING3, ZBTB2, MMP2, RNF130, ELMO1, COL1A2, SH2D4A, HTRA1, PALLD, RERG, NR2F1, ANGPTL2, LEP, PIK3CB, MLLT10, GNG11, CFL2, STAT5B, ICA1, PLIN2, CCDC127, SOCS2, SFXN3, CDO1, COG3, PDGFRA, DCP2, LYN, PLPP3, SLC2A1, MGAT5, SERPINB9, SPON1, TSPAN5, SLC25A25, CASP4, NEK11, WWC3, GPT2, OLFML2B, CTSZ, APOL4, SLCO4A1, CYBRD1, DNMT3A, AASDHPPT, WFS1, FSCN1, SLIT3, TDP1, RCBTB2, KRT19, FAM120AOS, SH3PXD2A, DUSP1, PRKCH, HMGB3, APLN, PMAIP1, LRRC42, ANTXR2, RASSF8, CHMP2B, C3AR1, SEPHS1, CDH11, DLX5, CEBPA, ACTG2, OPN3, PRRX1, NECTIN4, GOT1, PTBP3, RNF20, USP46, EFNB1, DCN, TYROBP, LRRC1, GFOD2, RFK, BHLHE40, PIK3AP1, RDH10, UBE2H, CALM1, MTFMT, GRHL1, CD200, IGF1, SEC22C, YTHDC2, LIMCH1, EZR, CREB1, PTPN1, AKAP13, FAP, SH3BGRL, GSTK1, ADCY7, ARL2BP, SMARCA2, ZFAND5, PRKCA, RASEF, ARHGDIB, NAXD, VPS45, SAR1A, CORO2A, ARMCX1, SLC35F6, NDUFB4, ACO1, CCSAP, PHYHIPL, INSIG1, KMT2E, GRINA, RNASE1, RUNX1T1, CHMP2A, NFIA, PTPRD, PROM1, IVD, NUFIP2, GLUL, CALM2, SEC61B, PDGFC, CADM3, CDC42BPA, CD28, GTDC1, SGCB, ST6GAL1, UROS, CMKLR1, NDUFB9, KITLG, DISP1, ASAH1, TLR7, MCTP2, MYO5A, MATN2, LURAP1L, PXK, CYBB, NPL, PIK3CG, MEIS1, PKD2, TBXAS1, ZEB2, HPGDS, NEDD4L, RAPH1, SPRED1, SLC9A9 |
|  |  | HELLP syndrome | Third trimester | INHA, ENG, SH3BP5, LRRC1, GALK1, SH3PXD2A, KMT2E, FLNB, PRKAB2, PFKFB4, HTRA1, SFXN3, SYDE1, EPS8L1, HEY1, PKD2, TRIM24, MOB3A, NECTIN4, PDE5A, ING3, PAM, KLF3, GBE1, SUV39H1, ACKR2, PLCXD2, PIGX, FOSL2, PKM, TREM1, RASEF, STK24, ALKBH5, PLEC, CRKL, ELF4, TAF9B, GPX3, MIIP, ZNF331, ARID3A, EAF1, PAPPA2, PIK3R1, HK1, TRIP10, DONSON, BHLHE40, GREM2, NFIL3, PRKCH, MOB3C, ASTE1, SLC2A1, CEBPA, SLC16A3, PHTF2, BAD, PGBD2, CYB561A3, CYBB, LRRC8A, RPS6KA5, DDR2, VEGFA, DYRK2, FPGS, COIL, SH3RF1, BTD, GCLM, CCDC127, CSNK2A2, SHANK2, CALM1, DIO2, MAP3K8, HPGDS, TM9SF3, PAWR, GRHL3, GORAB, PIK3CB, PPP1R12C, PJA1, VPS45, BCAR3, FRMD4A, SLC25A4, ZC2HC1A, BASP1, SEMA4B, QPCT, PLOD2, APLN, PREP, ERO1A, TXNRD1, MYOF, SYN3, WFS1, IL36RN, CLIC3, ARRDC3, SEMA4C, ERCC6, FBN2, NOD1, WNK2, HMGB3, IQGAP2, P2RY2, SPAG4, ZNF75A, SLC6A8, SPON1, MME, SCARB1, SEPHS1, IGSF8, CREB1, NEK11, GMFB, KIT, EPHB3, FSTL3, CLDN16, SLC23A2, DLX5, IGF1, DUSP1, ZNF555, BCORL1, ERRFI1, RNF20, SWAP70, GPT2, C4orf3, VEGFC, AZIN1, BCAT2, MAML2, PROCR, P4HA1, MAP3K9, CORO2A, IGSF3, ANKRD37, SERPINB2, SPRED1, CCSAP, NPEPPS, EFHD1, FAM20B, UBE2Q2, DAB2IP, CYP2J2, AFMID, ZEB2, ASAH1, TMEM45A, TSC22D1, CD28, HPCAL1, ZMYND8, APBB2, IRF2BPL, FAM43A, GPR146, MRPS30, NUFIP2, MTMR6, CDO1, PLIN2, EFNB1, TLR7, LYN, PPL, KIF5B, LIMCH1, GATA3, PIK3AP1, KRT19, EZR, PLPP3, GRHL1, WDR11, LEP |

FGR, fetal growth restriction; HELLP, hemolysis, elevated liver enzyme, and low platelet; PE, preeclampsia.

Table S3. Discovery of gene set function by GSAI for late-onset, severe PE.

| Representation of | | Ontology | GSAI |  |
| --- | --- | --- | --- | --- |
|  |  |  | Query | Response* |
| Chorio-amnionitis | Third trimester | CliXO:86 | ANKRD13A, SLA | Process: Protein Ubiquitination and Immune Response Regulation (0.85)   1. ANKRD13A [D/D], also known as Ankyrin Repeat Domain 13A, is a protein that has been found to be involved in the process of ubiquitination, a post-translational modification that regulates protein degradation, cellular localization, and activity. ANKRD13A [D/D] has been shown to interact with ubiquitin and facilitate the transport of ubiquitinated proteins to the proteasome for degradation. This process is crucial for maintaining protein homeostasis within the cell. 2. SLA [U/U], Src-like-adaptor, is a protein that plays a significant role in immune response regulation. It acts as a negative regulator of T-cell receptor (TCR) signaling, which is a critical pathway in the immune response. By inhibiting TCR signaling, SLA [U/U] helps to prevent overactive immune responses and maintain immune system balance.   In summary, the proteins in this system are primarily involved in the regulation of protein ubiquitination and immune response. ANKRD13A [D/D] contributes to the maintenance of protein homeostasis through its role in protein ubiquitination and degradation, while SLA [U/U] plays a crucial role in immune response regulation through its inhibitory effect on TCR signaling. These processes are essential for maintaining cellular function and immune system balance. |
| HELLP syndrome | Third trimester | CliXO:70 | PDE5A | Process: Cyclic Guanosine Monophosphate (cGMP) Degradation (0.95)  PDE5A [D/D], or Phosphodiesterase 5A, is a key enzyme involved in the degradation of cyclic guanosine monophosphate (cGMP), a secondary messenger molecule that plays a crucial role in various physiological processes.  The primary function of PDE5A [D/D] is to hydrolyze cGMP to 5'-GMP, thereby terminating its signaling function. This protein is predominantly found in vascular smooth muscle cells, where it plays a critical role in regulating vascular tone and blood flow. By degrading cGMP, PDE5A [D/D] modulates the effects of nitric oxide (NO), a potent vasodilator that increases the concentration of cGMP in cells.  In the context of the cardiovascular system, PDE5A [D/D]'s activity is crucial for the regulation of blood pressure and cardiac function. Inhibition of PDE5A [D/D], as seen with drugs like sildenafil, can enhance the vasodilatory effects of NO, leading to decreased blood pressure and improved blood flow.  In the nervous system, PDE5A [D/D] also plays a role in the regulation of neuronal signaling. cGMP is involved in the modulation of synaptic plasticity and memory formation, and the activity of PDE5A [D/D] can influence these processes.  In summary, the primary biological process performed by PDE5A [D/D] is the degradation of cGMP, a process that has significant implications for vascular and neuronal function. The protein's role in this process is well-established, hence the high confidence score. |

*, confidence score is shown in parentheses after the process name and each mention of gene name is modified by including up (U)- or down (D)-regulation status of respectively the associated factor and third-trimester placenta under late-onset, severe PE; CliXO, clique-extracted ontology; GSAI, gene set artificial intelligence; HELLP, hemolysis, elevated liver enzyme, and low platelet; PE, preeclampsia.

Table S4. Discovery of gene set function by GSAI for early-onset, severe PE.

| Representation of | | Ontology | GSAI |  |
| --- | --- | --- | --- | --- |
|  |  |  | Query | Response* |
| HELLP syndrome | Third trimester | root | ALKBH5, SWAP70, DUSP1, KLF4, GREM2, GPR146, PIP5K1B, EZR | Process: Regulation of Cellular Differentiation and Signal Transduction (0.85)  1. ALKBH5 [U/U] is a demethylase that plays a crucial role in RNA metabolism and adipogenesis. It is involved in the regulation of mRNA processing and export, contributing to the overall process of gene expression.  2. SWAP70 [U/U] and PIP5K1B [D/D] are involved in the regulation of actin cytoskeleton organization. SWAP70 [U/U] is a signaling protein that mediates the activation of RAC1, a key regulator of actin cytoskeleton dynamics. PIP5K1B [D/D], on the other hand, is a kinase that generates phosphatidylinositol 4,5-bisphosphate, a critical component of the inner cell membrane that plays a role in signal transduction and cytoskeleton organization.  3. DUSP1 [U/U] is a dual specificity phosphatase that dephosphorylates MAP kinase, thereby playing a role in the MAP kinase signal transduction pathway. It is involved in the regulation of cellular processes such as proliferation, differentiation, and stress response.  4. KLF4 [U/U] is a transcription factor that plays a key role in maintaining stem cell pluripotency and promoting cellular reprogramming and differentiation. It is involved in various biological processes, including cell cycle regulation, apoptosis, and epithelial to mesenchymal transition.  5. GREM2 [U/U] is a BMP (bone morphogenetic protein) antagonist that plays a role in regulating organogenesis and differentiation. It is involved in various developmental processes, including limb bud formation, skeletal development, and neural tube closure.  6. GPR146 [U/U] is an orphan G-protein coupled receptor that is thought to be involved in signal transduction, although its exact function is not well understood.  7. EZR [U/U] is a member of the ezrin-radixin-moesin (ERM) family of proteins that link the actin cytoskeleton to the plasma membrane. It plays a role in various cellular processes, including cell adhesion, migration, and signal transduction.  In summary, the proteins in this system are involved in various aspects of cellular differentiation and signal transduction. They contribute to the regulation of gene expression, actin cytoskeleton organization, MAP kinase signaling, stem cell pluripotency, organogenesis, and potentially other signaling pathways. Their interplay likely results in a complex network of regulatory mechanisms that control cellular differentiation and response to external signals. |

*, confidence score is shown in parentheses after the process name and each mention of gene name is modified by including up (U)- or down (D)-regulation status of respectively the associated factor and third-trimester placenta under early-onset, severe PE; CliXO, clique-extracted ontology; GSAI, gene set artificial intelligence; HELLP, hemolysis, elevated liver enzyme, and low platelet; PE, preeclampsia.

Table S5. Threshold-specific confusion matrices for the late-onset, severe PE pathogenesis model connected with mid-secretory endometrium.

| Ontology | PPV/TPR (Dev) | PPV/TPR (Test) | NPV/TNR (Dev) | NPV/TNR (Test) | C1 | C2 | C3 | C4 | Reported |
| --- | --- | --- | --- | --- | --- | --- | --- | --- | --- |
| root (ICA1, PLIN2, BMP2, DOCK8, GDA, NFIL3, RDX, CCDC127, CORO2A, BCAR3, SPAG4, FAM172A, ANKRD37, PKM, CLIP1, TUBB4B, AZIN1, ANKRD13A, PDE5A, IGF1, TUFT1, KRT19, INPP5F, EFNB1, SMPDL3A, PTPRD, NPR3, P2RY14, CTBS, SH3PXD2A, GRHL1, CDO1, LDLR, MYC, ASAH1, MRPL34, ZNF555, ELOVL5, APOLD1, FAM43A, TIAM1, BBS4, MCAT, DYNLL1, SCYL3, GPT2, SLCO2A1, SYDE1, HYAL2, TOR1B, PIK3R1, ERO1A, APPBP2, EDEM1, FAP, NECTIN4, DDIT4, NEDD4L, APLN, KLF3, GMFB, SLCO4A1, P4HA1, ERRFI1, TRIM68, ETS2, CEBPD, GULP1, RAPGEF3, FLNB, GPX3, DEGS1) | 58.1% (53.4, 62.8) / 100.0% (100.0, 100.0) | 2.8% (2.3, 3.2) / 100.0% (100.0, 100.0) | 100.0% (100.0, 100.0) / 80.8% (77.5, 84.0) | — / 0.0% (0.0, 0.0) | ✓\| | \| |  |  |  |
| CliXO:111 (ICA1, PLIN2, BMP2, DOCK8, GDA, NFIL3, RDX, CCDC127, CORO2A, BCAR3, SPAG4, FAM172A, ANKRD37, PKM, CLIP1, TUBB4B, AZIN1, ANKRD13A, PDE5A, IGF1, TUFT1, KRT19, INPP5F, EFNB1, SMPDL3A, PTPRD, NPR3, P2RY14, CTBS, SH3PXD2A, GRHL1, CDO1, LDLR, MYC, ASAH1, MRPL34†, ZNF555, ELOVL5, APOLD1, FAM43A, TIAM1, BBS4, MCAT, DYNLL1†, SCYL3, GPT2, SLCO2A1, SYDE1, HYAL2, TOR1B, PIK3R1, ERO1A, APPBP2, EDEM1, FAP, NECTIN4, DDIT4, NEDD4L, APLN, KLF3, GMFB, SLCO4A1, P4HA1, ERRFI1, TRIM68, ETS2, CEBPD, GULP1, RAPGEF3, FLNB, GPX3, DEGS1) | 20.6% (18.2, 23.1) / 100.0% (100.0, 100.0) | 2.8% (2.3, 3.2) / 100.0% (100.0, 100.0) | — / 0.0% (0.0, 0.0) | — / 0.0% (0.0, 0.0) | ✓\| | ✓\| | ✓ |  | CliXO:111 |
| ├── CliXO:107 (BMP2, DOCK8, RDX, FAM172A, PKM, TUBB4B, AZIN1, PDE5A, IGF1, INPP5F, EFNB1, SMPDL3A, PTPRD, P2RY14, SH3PXD2A, CDO1, LDLR, ASAH1, MRPL34, ZNF555, ELOVL5, APOLD1, FAM43A, BBS4, MCAT, DYNLL1, SLCO2A1, SYDE1, HYAL2, FAP, DDIT4, APLN, P4HA1, ERRFI1, TRIM68) | 20.6% (18.2, 23.1) / 100.0% (100.0, 100.0) | 2.8% (2.3, 3.2) / 100.0% (100.0, 100.0) | — / 0.0% (0.0, 0.0) | — / 0.0% (0.0, 0.0) | ✓\| | ✓\| |  |  | CliXO:111 |
| │ ├── CliXO:098 (DOCK8, RDX, AZIN1, PDE5A, IGF1, SMPDL3A, PTPRD, ASAH1, FAM43A, FAP, DDIT4, P4HA1, ERRFI1) | 20.6% (18.2, 23.1) / 100.0% (100.0, 100.0) | 2.8% (2.3, 3.2) / 100.0% (100.0, 100.0) | — / 0.0% (0.0, 0.0) | — / 0.0% (0.0, 0.0) | ✓\| | ✓\| |  |  |  |
| │ │ └── CliXO:093 (DOCK8, RDX, AZIN1, PDE5A, IGF1, FAP, DDIT4, P4HA1, ERRFI1) | 20.6% (18.2, 23.1) / 100.0% (100.0, 100.0) | 2.8% (2.3, 3.2) / 100.0% (100.0, 100.0) | — / 0.0% (0.0, 0.0) | — / 0.0% (0.0, 0.0) | ✓\| | ✓\| |  |  |  |
| │ │ ├── CliXO:074 (RDX, AZIN1) | 20.6% (18.2, 23.1) / 100.0% (100.0, 100.0) | 2.8% (2.3, 3.2) / 100.0% (100.0, 100.0) | — / 0.0% (0.0, 0.0) | — / 0.0% (0.0, 0.0) | ✓\| | ✓\| |  |  |  |
| │ │ └── CliXO:082 (RDX, P4HA1) | 20.6% (18.2, 23.1) / 100.0% (100.0, 100.0) | 2.8% (2.3, 3.2) / 100.0% (100.0, 100.0) | — / 0.0% (0.0, 0.0) | — / 0.0% (0.0, 0.0) | ✓\| | ✓\| |  |  |  |
| │ └── CliXO:105 (BMP2, RDX, FAM172A, PKM, TUBB4B, AZIN1, PDE5A, IGF1, INPP5F, EFNB1, PTPRD, P2RY14, SH3PXD2A, CDO1, MRPL34, ELOVL5, APOLD1, FAM43A, BBS4, MCAT, DYNLL1, SLCO2A1, SYDE1, HYAL2, FAP, APLN, TRIM68) | 20.6% (18.2, 23.1) / 100.0% (100.0, 100.0) | 2.8% (2.3, 3.2) / 100.0% (100.0, 100.0) | — / 0.0% (0.0, 0.0) | — / 0.0% (0.0, 0.0) | ✓\| | ✓\| |  |  | CliXO:111 |
| │ ├── CliXO:088 (PDE5A, INPP5F, PTPRD, CDO1, APOLD1) | 20.6% (18.2, 23.1) / 100.0% (100.0, 100.0) | 2.8% (2.3, 3.2) / 100.0% (100.0, 100.0) | — / 0.0% (0.0, 0.0) | — / 0.0% (0.0, 0.0) | ✓\| | ✓\| |  |  |  |
| │ └── CliXO:104 (BMP2, RDX, FAM172A, PKM, TUBB4B, AZIN1, PDE5A, IGF1, INPP5F, EFNB1, PTPRD, P2RY14, SH3PXD2A, CDO1, MRPL34, ELOVL5, FAM43A, BBS4, MCAT, DYNLL1, SLCO2A1, SYDE1, HYAL2, FAP, APLN, TRIM68) | 20.6% (18.2, 23.1) / 100.0% (100.0, 100.0) | 2.8% (2.3, 3.2) / 100.0% (100.0, 100.0) | — / 0.0% (0.0, 0.0) | — / 0.0% (0.0, 0.0) | ✓\| | ✓\| |  |  |  |
| │ ├── CliXO:072 (SLCO2A1, TRIM68) | 20.6% (18.2, 23.1) / 100.0% (100.0, 100.0) | 2.8% (2.3, 3.2) / 100.0% (100.0, 100.0) | — / 0.0% (0.0, 0.0) | — / 0.0% (0.0, 0.0) | ✓\| | ✓\| |  |  |  |
| │ ├── CliXO:075 (RDX, TUBB4B) | 20.6% (18.2, 23.1) / 100.0% (100.0, 100.0) | 2.8% (2.3, 3.2) / 100.0% (100.0, 100.0) | — / 0.0% (0.0, 0.0) | — / 0.0% (0.0, 0.0) | ✓\| | ✓\| |  |  |  |
| │ ├── CliXO:087 (PKM, SH3PXD2A, ELOVL5, MCAT, DYNLL1) | 20.6% (18.2, 23.1) / 100.0% (100.0, 100.0) | 2.8% (2.3, 3.2) / 100.0% (100.0, 100.0) | — / 0.0% (0.0, 0.0) | — / 0.0% (0.0, 0.0) | ✓\| | ✓\| |  |  |  |
| │ │ └── CliXO:077 (PKM, MCAT) | 20.6% (18.2, 23.1) / 100.0% (100.0, 100.0) | 2.8% (2.3, 3.2) / 100.0% (100.0, 100.0) | — / 0.0% (0.0, 0.0) | — / 0.0% (0.0, 0.0) | ✓\| | ✓\| |  |  |  |
| │ └── CliXO:100 (BMP2, RDX, FAM172A, PKM, AZIN1, INPP5F, EFNB1, PTPRD, P2RY14, SH3PXD2A, CDO1, FAM43A, BBS4, MCAT, DYNLL1, SYDE1, HYAL2, FAP, TRIM68) | 20.6% (18.2, 23.1) / 100.0% (100.0, 100.0) | 2.8% (2.3, 3.2) / 100.0% (100.0, 100.0) | — / 0.0% (0.0, 0.0) | — / 0.0% (0.0, 0.0) | ✓\| | ✓\| |  |  |  |
| │ ├── CliXO:080 (PTPRD, BBS4) | 20.6% (18.2, 23.1) / 100.0% (100.0, 100.0) | 2.8% (2.3, 3.2) / 100.0% (100.0, 100.0) | — / 0.0% (0.0, 0.0) | — / 0.0% (0.0, 0.0) | ✓\| | ✓\| |  |  |  |
| │ ├── CliXO:086 (PTPRD, CDO1, FAM43A, FAP) | 20.6% (18.2, 23.1) / 100.0% (100.0, 100.0) | 2.8% (2.3, 3.2) / 100.0% (100.0, 100.0) | — / 0.0% (0.0, 0.0) | — / 0.0% (0.0, 0.0) | ✓\| | ✓\| |  |  |  |
| │ ├── CliXO:092 (RDX, AZIN1, INPP5F, MCAT, DYNLL1, HYAL2, TRIM68) | 20.6% (18.2, 23.1) / 100.0% (100.0, 100.0) | 2.8% (2.3, 3.2) / 100.0% (100.0, 100.0) | — / 0.0% (0.0, 0.0) | — / 0.0% (0.0, 0.0) | ✓\| | ✓\| |  |  |  |
| │ │ ├── CliXO:076 (INPP5F, DYNLL1) | 20.6% (18.2, 23.1) / 100.0% (100.0, 100.0) | 2.8% (2.3, 3.2) / 100.0% (100.0, 100.0) | — / 0.0% (0.0, 0.0) | — / 0.0% (0.0, 0.0) | ✓\| | ✓\| |  |  |  |
| │ │ └── CliXO:085 (RDX, AZIN1, DYNLL1, TRIM68) | 20.6% (18.2, 23.1) / 100.0% (100.0, 100.0) | 2.8% (2.3, 3.2) / 100.0% (100.0, 100.0) | — / 0.0% (0.0, 0.0) | — / 0.0% (0.0, 0.0) | ✓\| | ✓\| |  |  |  |
| │ └── CliXO:096 (BMP2, FAM172A, PKM, INPP5F, EFNB1, P2RY14, SH3PXD2A, CDO1, MCAT, DYNLL1, SYDE1) | 20.6% (18.2, 23.1) / 100.0% (100.0, 100.0) | 2.8% (2.3, 3.2) / 100.0% (100.0, 100.0) | — / 0.0% (0.0, 0.0) | — / 0.0% (0.0, 0.0) | ✓\| | ✓\| |  |  |  |
| │ └── CliXO:089 (BMP2, INPP5F, P2RY14, CDO1, DYNLL1, SYDE1) | 20.6% (18.2, 23.1) / 100.0% (100.0, 100.0) | 2.8% (2.3, 3.2) / 100.0% (100.0, 100.0) | — / 0.0% (0.0, 0.0) | — / 0.0% (0.0, 0.0) | ✓\| | ✓\| |  |  |  |
| └── CliXO:110 (ICA1, PLIN2, DOCK8, GDA, NFIL3, CCDC127, CORO2A, BCAR3, SPAG4, ANKRD37, CLIP1, ANKRD13A, TUFT1, KRT19, SMPDL3A, NPR3, CTBS, GRHL1, LDLR, MYC, ASAH1, ZNF555, TIAM1, SCYL3, GPT2, TOR1B, PIK3R1, ERO1A, APPBP2, EDEM1, NECTIN4, DDIT4, NEDD4L, KLF3, GMFB, SLCO4A1, P4HA1, ERRFI1, ETS2, CEBPD, GULP1, RAPGEF3, FLNB, GPX3, DEGS1) | 20.6% (18.2, 23.1) / 100.0% (100.0, 100.0) | 2.8% (2.3, 3.2) / 100.0% (100.0, 100.0) | — / 0.0% (0.0, 0.0) | — / 0.0% (0.0, 0.0) | ✓\| | ✓\| |  |  |  |
| ├── CliXO:101 (DOCK8, CCDC127, ANKRD13A, SMPDL3A, GRHL1, ASAH1, ZNF555, TIAM1, SCYL3, TOR1B, PIK3R1, EDEM1, NEDD4L, KLF3, GMFB, SLCO4A1, GULP1, RAPGEF3, FLNB, GPX3, DEGS1) | 20.6% (18.2, 23.1) / 100.0% (100.0, 100.0) | 2.8% (2.3, 3.2) / 100.0% (100.0, 100.0) | — / 0.0% (0.0, 0.0) | — / 0.0% (0.0, 0.0) | ✓\| | ✓\| |  |  |  |
| │ ├── CliXO:073 (GRHL1, NEDD4L) | 20.6% (18.2, 23.1) / 100.0% (100.0, 100.0) | 2.8% (2.3, 3.2) / 100.0% (100.0, 100.0) | — / 0.0% (0.0, 0.0) | — / 0.0% (0.0, 0.0) | ✓\| | ✓\| |  |  |  |
| │ ├── CliXO:078 (CCDC127, TIAM1) | 20.6% (18.2, 23.1) / 100.0% (100.0, 100.0) | 2.8% (2.3, 3.2) / 100.0% (100.0, 100.0) | — / 0.0% (0.0, 0.0) | — / 0.0% (0.0, 0.0) | ✓\| | ✓\| |  |  |  |
| │ ├── CliXO:079 (KLF3, DEGS1) | 20.6% (18.2, 23.1) / 100.0% (100.0, 100.0) | 2.8% (2.3, 3.2) / 100.0% (100.0, 100.0) | — / 0.0% (0.0, 0.0) | — / 0.0% (0.0, 0.0) | ✓\| | ✓\| |  |  |  |
| │ ├── CliXO:081 (SCYL3, GMFB) | 20.6% (18.2, 23.1) / 100.0% (100.0, 100.0) | 2.8% (2.3, 3.2) / 100.0% (100.0, 100.0) | — / 0.0% (0.0, 0.0) | — / 0.0% (0.0, 0.0) | ✓\| | ✓\| |  |  |  |
| │ └── CliXO:095 (DOCK8, CCDC127, SMPDL3A, ASAH1, ZNF555, PIK3R1, GMFB, RAPGEF3, FLNB, GPX3) | 20.6% (18.2, 23.1) / 100.0% (100.0, 100.0) | 2.8% (2.3, 3.2) / 100.0% (100.0, 100.0) | — / 0.0% (0.0, 0.0) | — / 0.0% (0.0, 0.0) | ✓\| | ✓\| |  |  |  |
| │ └── CliXO:090 (DOCK8, CCDC127, ASAH1, PIK3R1, RAPGEF3, GPX3) | 20.6% (18.2, 23.1) / 100.0% (100.0, 100.0) | 2.8% (2.3, 3.2) / 100.0% (100.0, 100.0) | — / 0.0% (0.0, 0.0) | — / 0.0% (0.0, 0.0) | ✓\| | ✓\| |  |  |  |
| │ └── CliXO:083 (PIK3R1, RAPGEF3, GPX3) | 20.6% (18.2, 23.1) / 100.0% (100.0, 100.0) | 2.8% (2.3, 3.2) / 100.0% (100.0, 100.0) | — / 0.0% (0.0, 0.0) | — / 0.0% (0.0, 0.0) | ✓\| | ✓\| |  |  |  |
| └── CliXO:109 (ICA1, PLIN2, DOCK8, GDA, NFIL3, CORO2A, BCAR3, SPAG4, ANKRD37, CLIP1, ANKRD13A, TUFT1, KRT19, SMPDL3A, NPR3, CTBS, GRHL1, LDLR, MYC, ASAH1, ZNF555, SCYL3, GPT2, TOR1B, PIK3R1, ERO1A, APPBP2, EDEM1, NECTIN4, DDIT4, NEDD4L, KLF3, GMFB, SLCO4A1, P4HA1, ERRFI1, ETS2, CEBPD, GULP1, RAPGEF3, FLNB, GPX3, DEGS1) | 20.6% (18.2, 23.1) / 100.0% (100.0, 100.0) | 2.8% (2.3, 3.2) / 100.0% (100.0, 100.0) | — / 0.0% (0.0, 0.0) | — / 0.0% (0.0, 0.0) | ✓\| | ✓\| |  |  |  |
| ├── CliXO:094 (PLIN2, GDA, ANKRD37, KRT19, NPR3, LDLR, GPT2, NECTIN4, DDIT4) | 20.6% (18.2, 23.1) / 100.0% (100.0, 100.0) | 2.8% (2.3, 3.2) / 100.0% (100.0, 100.0) | — / 0.0% (0.0, 0.0) | — / 0.0% (0.0, 0.0) | ✓\| | ✓\| |  |  |  |
| │ └── CliXO:084 (PLIN2, ANKRD37, NPR3) | 20.6% (18.2, 23.1) / 100.0% (100.0, 100.0) | 2.8% (2.3, 3.2) / 100.0% (100.0, 100.0) | — / 0.0% (0.0, 0.0) | — / 0.0% (0.0, 0.0) | ✓\| | ✓\| |  |  |  |
| └── CliXO:108 (ICA1, PLIN2, DOCK8, GDA, NFIL3, CORO2A, BCAR3, SPAG4, ANKRD37, CLIP1, ANKRD13A, TUFT1, KRT19, SMPDL3A, NPR3, CTBS, GRHL1, ASAH1, ZNF555, SCYL3, GPT2, TOR1B, PIK3R1, ERO1A, APPBP2, EDEM1, NEDD4L, KLF3, GMFB, SLCO4A1, P4HA1, ERRFI1, ETS2, CEBPD, GULP1, RAPGEF3, FLNB, GPX3, DEGS1) | 20.6% (18.2, 23.1) / 100.0% (100.0, 100.0) | 2.8% (2.3, 3.2) / 100.0% (100.0, 100.0) | — / 0.0% (0.0, 0.0) | — / 0.0% (0.0, 0.0) | ✓\| | ✓\| |  |  |  |
| ├── CliXO:099 (ICA1, DOCK8, CLIP1, ANKRD13A, SMPDL3A, CTBS, GRHL1, ASAH1, ZNF555, SCYL3, TOR1B, PIK3R1, APPBP2, NEDD4L, KLF3, GMFB, RAPGEF3, FLNB, GPX3) | 20.6% (18.2, 23.1) / 100.0% (100.0, 100.0) | 2.8% (2.3, 3.2) / 100.0% (100.0, 100.0) | — / 0.0% (0.0, 0.0) | — / 0.0% (0.0, 0.0) | ✓\| | ✓\| |  |  |  |
| │ └── CliXO:091 (DOCK8, SMPDL3A, ZNF555, PIK3R1, APPBP2, KLF3, GPX3) | 20.6% (18.2, 23.1) / 100.0% (100.0, 100.0) | 2.8% (2.3, 3.2) / 100.0% (100.0, 100.0) | — / 0.0% (0.0, 0.0) | — / 0.0% (0.0, 0.0) | ✓\| | ✓\| |  |  |  |
| └── CliXO:106 (ICA1, PLIN2, GDA, NFIL3, CORO2A, BCAR3, SPAG4, ANKRD37, CLIP1, ANKRD13A, TUFT1, KRT19, NPR3, CTBS, GRHL1, GPT2, PIK3R1, ERO1A, APPBP2, EDEM1, KLF3, GMFB, SLCO4A1, P4HA1, ERRFI1, ETS2, CEBPD, GULP1, RAPGEF3, FLNB, GPX3, DEGS1) | 20.6% (18.2, 23.1) / 100.0% (100.0, 100.0) | 2.8% (2.3, 3.2) / 100.0% (100.0, 100.0) | — / 0.0% (0.0, 0.0) | — / 0.0% (0.0, 0.0) | ✓\| | ✓\| |  |  |  |
| └── CliXO:103 (ICA1, PLIN2, GDA, NFIL3, CORO2A, BCAR3, CLIP1, ANKRD13A, TUFT1, KRT19, NPR3, GRHL1, GPT2, ERO1A, KLF3, GMFB, SLCO4A1, P4HA1, ERRFI1, ETS2, CEBPD, RAPGEF3, FLNB, GPX3, DEGS1) | 20.6% (18.2, 23.1) / 100.0% (100.0, 100.0) | 2.8% (2.3, 3.2) / 100.0% (100.0, 100.0) | — / 0.0% (0.0, 0.0) | — / 0.0% (0.0, 0.0) | ✓\| | ✓\| |  |  |  |
| └── CliXO:102 (ICA1, PLIN2, GDA, NFIL3, CORO2A, CLIP1, ANKRD13A, TUFT1, KRT19, NPR3, GRHL1, GPT2, ERO1A, SLCO4A1, P4HA1, ERRFI1, ETS2, CEBPD, FLNB, GPX3, DEGS1) | 20.6% (18.2, 23.1) / 100.0% (100.0, 100.0) | 2.8% (2.3, 3.2) / 100.0% (100.0, 100.0) | — / 0.0% (0.0, 0.0) | — / 0.0% (0.0, 0.0) | ✓\| | ✓\| |  |  |  |
| └── CliXO:097 (PLIN2, NFIL3, CORO2A, CLIP1, TUFT1, ERO1A, SLCO4A1, ETS2, CEBPD, FLNB, GPX3, DEGS1) | 20.6% (18.2, 23.1) / 100.0% (100.0, 100.0) | 2.8% (2.3, 3.2) / 100.0% (100.0, 100.0) | — / 0.0% (0.0, 0.0) | — / 0.0% (0.0, 0.0) | ✓\| | ✓\| |  |  |  |

The Reported column indicates the ontology from Tables 1–2 to which this row’s eligible genes belong. An ontology is reported if it passes all criteria (C1–C4) and its eligible gene set is not a strict subset of another passing ontology’s gene set. The column is empty when the ontology has no eligible genes or does not pass all criteria. ✓, the ontology passed the criterion; †, the gene had a grad-CAM value >97.5th percentile in the same ontology; |, separator between PPV/TPR and NPV/TNR results in C1 and C2 columns; AUC-ROC, area under the receiver operating characteristic curve; C1, lower bounds finite and >0; C2, absolute difference ≤25% between development and test sets; C3, at least 1 eligible gene; C4, lower bound of AUC-ROC >0.5 in both sets; CI, confidence interval; Dev, development set; grad-CAM, gradient-weighted class activation mapping; HELLP, hemolysis, elevated liver enzyme, and low platelet; NPV, negative predictive value; PE, preeclampsia; PPV, positive predictive value; TNR, true negative rate; TPR, true positive rate.

Table S6. AUC-ROC, AUC-PRC, and RMSE for the late-onset, severe PE pathogenesis model connected with mid-secretory endometrium.

| Ontology | AUC-ROC (Dev) | AUC-ROC (Test) | AUC-PRC (Dev) | AUC-PRC (Test) | RMSE (Dev) | RMSE (Test) | C1 | C2 | C3 | C4 | Reported |
| --- | --- | --- | --- | --- | --- | --- | --- | --- | --- | --- | --- |
| root (ICA1, PLIN2, BMP2, DOCK8, GDA, NFIL3, RDX, CCDC127, CORO2A, BCAR3, SPAG4, FAM172A, ANKRD37, PKM, CLIP1, TUBB4B, AZIN1, ANKRD13A, PDE5A, IGF1, TUFT1, KRT19, INPP5F, EFNB1, SMPDL3A, PTPRD, NPR3, P2RY14, CTBS, SH3PXD2A, GRHL1, CDO1, LDLR, MYC, ASAH1, MRPL34, ZNF555, ELOVL5, APOLD1, FAM43A, TIAM1, BBS4, MCAT, DYNLL1, SCYL3, GPT2, SLCO2A1, SYDE1, HYAL2, TOR1B, PIK3R1, ERO1A, APPBP2, EDEM1, FAP, NECTIN4, DDIT4, NEDD4L, APLN, KLF3, GMFB, SLCO4A1, P4HA1, ERRFI1, TRIM68, ETS2, CEBPD, GULP1, RAPGEF3, FLNB, GPX3, DEGS1) | 0.904 (0.888, 0.920) | 0.500 (0.500, 0.500) | 0.609 | 0.516 | 0.430 (±0.0080) | 0.371 (±0.0017) | ✓\| | \| |  |  |  |
| CliXO:111 (ICA1, PLIN2, BMP2, DOCK8, GDA, NFIL3, RDX, CCDC127, CORO2A, BCAR3, SPAG4, FAM172A, ANKRD37, PKM, CLIP1, TUBB4B, AZIN1, ANKRD13A, PDE5A, IGF1, TUFT1, KRT19, INPP5F, EFNB1, SMPDL3A, PTPRD, NPR3, P2RY14, CTBS, SH3PXD2A, GRHL1, CDO1, LDLR, MYC, ASAH1, MRPL34†, ZNF555, ELOVL5, APOLD1, FAM43A, TIAM1, BBS4, MCAT, DYNLL1†, SCYL3, GPT2, SLCO2A1, SYDE1, HYAL2, TOR1B, PIK3R1, ERO1A, APPBP2, EDEM1, FAP, NECTIN4, DDIT4, NEDD4L, APLN, KLF3, GMFB, SLCO4A1, P4HA1, ERRFI1, TRIM68, ETS2, CEBPD, GULP1, RAPGEF3, FLNB, GPX3, DEGS1) | 0.500 (0.500, 0.500) | 0.500 (0.500, 0.500) | 0.609 | 0.516 | 0.813 (±0.0125) | 0.899 (±0.0021) | ✓\| | ✓\| | ✓ |  | CliXO:111 |
| ├── CliXO:107 (BMP2, DOCK8, RDX, FAM172A, PKM, TUBB4B, AZIN1, PDE5A, IGF1, INPP5F, EFNB1, SMPDL3A, PTPRD, P2RY14, SH3PXD2A, CDO1, LDLR, ASAH1, MRPL34, ZNF555, ELOVL5, APOLD1, FAM43A, BBS4, MCAT, DYNLL1, SLCO2A1, SYDE1, HYAL2, FAP, DDIT4, APLN, P4HA1, ERRFI1, TRIM68) | 0.500 (0.500, 0.500) | 0.500 (0.500, 0.500) | 0.609 | 0.516 | 0.813 (±0.0125) | 0.899 (±0.0021) | ✓\| | ✓\| |  |  | CliXO:111 |
| │ ├── CliXO:098 (DOCK8, RDX, AZIN1, PDE5A, IGF1, SMPDL3A, PTPRD, ASAH1, FAM43A, FAP, DDIT4, P4HA1, ERRFI1) | 0.500 (0.500, 0.500) | 0.500 (0.500, 0.500) | 0.609 | 0.516 | 0.756 (±0.0112) | 0.835 (±0.0019) | ✓\| | ✓\| |  |  |  |
| │ │ └── CliXO:093 (DOCK8, RDX, AZIN1, PDE5A, IGF1, FAP, DDIT4, P4HA1, ERRFI1) | 0.500 (0.500, 0.500) | 0.500 (0.500, 0.500) | 0.609 | 0.516 | 0.756 (±0.0112) | 0.835 (±0.0019) | ✓\| | ✓\| |  |  |  |
| │ │ ├── CliXO:074 (RDX, AZIN1) | 0.500 (0.500, 0.500) | 0.500 (0.500, 0.500) | 0.609 | 0.516 | 0.756 (±0.0112) | 0.835 (±0.0019) | ✓\| | ✓\| |  |  |  |
| │ │ └── CliXO:082 (RDX, P4HA1) | 0.500 (0.500, 0.500) | 0.500 (0.500, 0.500) | 0.609 | 0.516 | 0.756 (±0.0112) | 0.835 (±0.0019) | ✓\| | ✓\| |  |  |  |
| │ └── CliXO:105 (BMP2, RDX, FAM172A, PKM, TUBB4B, AZIN1, PDE5A, IGF1, INPP5F, EFNB1, PTPRD, P2RY14, SH3PXD2A, CDO1, MRPL34, ELOVL5, APOLD1, FAM43A, BBS4, MCAT, DYNLL1, SLCO2A1, SYDE1, HYAL2, FAP, APLN, TRIM68) | 0.500 (0.500, 0.500) | 0.500 (0.500, 0.500) | 0.609 | 0.516 | 0.807 (±0.0123) | 0.892 (±0.0021) | ✓\| | ✓\| |  |  | CliXO:111 |
| │ ├── CliXO:088 (PDE5A, INPP5F, PTPRD, CDO1, APOLD1) | 0.500 (0.500, 0.500) | 0.500 (0.500, 0.500) | 0.609 | 0.516 | 0.756 (±0.0112) | 0.835 (±0.0019) | ✓\| | ✓\| |  |  |  |
| │ └── CliXO:104 (BMP2, RDX, FAM172A, PKM, TUBB4B, AZIN1, PDE5A, IGF1, INPP5F, EFNB1, PTPRD, P2RY14, SH3PXD2A, CDO1, MRPL34, ELOVL5, FAM43A, BBS4, MCAT, DYNLL1, SLCO2A1, SYDE1, HYAL2, FAP, APLN, TRIM68) | 0.500 (0.500, 0.500) | 0.500 (0.500, 0.500) | 0.609 | 0.516 | 0.448 (±0.0268) | 0.162 (±0.0139) | ✓\| | ✓\| |  |  |  |
| │ ├── CliXO:072 (SLCO2A1, TRIM68) | 0.500 (0.500, 0.500) | 0.500 (0.500, 0.500) | 0.609 | 0.516 | 0.756 (±0.0112) | 0.835 (±0.0019) | ✓\| | ✓\| |  |  |  |
| │ ├── CliXO:075 (RDX, TUBB4B) | 0.500 (0.500, 0.500) | 0.500 (0.500, 0.500) | 0.609 | 0.516 | 0.756 (±0.0112) | 0.835 (±0.0019) | ✓\| | ✓\| |  |  |  |
| │ ├── CliXO:087 (PKM, SH3PXD2A, ELOVL5, MCAT, DYNLL1) | 0.500 (0.500, 0.500) | 0.500 (0.500, 0.500) | 0.609 | 0.516 | 0.756 (±0.0112) | 0.835 (±0.0019) | ✓\| | ✓\| |  |  |  |
| │ │ └── CliXO:077 (PKM, MCAT) | 0.500 (0.500, 0.500) | 0.500 (0.500, 0.500) | 0.609 | 0.516 | 0.756 (±0.0112) | 0.835 (±0.0019) | ✓\| | ✓\| |  |  |  |
| │ └── CliXO:100 (BMP2, RDX, FAM172A, PKM, AZIN1, INPP5F, EFNB1, PTPRD, P2RY14, SH3PXD2A, CDO1, FAM43A, BBS4, MCAT, DYNLL1, SYDE1, HYAL2, FAP, TRIM68) | 0.500 (0.500, 0.500) | 0.500 (0.500, 0.500) | 0.609 | 0.516 | 0.756 (±0.0112) | 0.835 (±0.0019) | ✓\| | ✓\| |  |  |  |
| │ ├── CliXO:080 (PTPRD, BBS4) | 0.500 (0.500, 0.500) | 0.500 (0.500, 0.500) | 0.609 | 0.516 | 0.756 (±0.0112) | 0.835 (±0.0019) | ✓\| | ✓\| |  |  |  |
| │ ├── CliXO:086 (PTPRD, CDO1, FAM43A, FAP) | 0.500 (0.500, 0.500) | 0.500 (0.500, 0.500) | 0.609 | 0.516 | 0.756 (±0.0112) | 0.835 (±0.0019) | ✓\| | ✓\| |  |  |  |
| │ ├── CliXO:092 (RDX, AZIN1, INPP5F, MCAT, DYNLL1, HYAL2, TRIM68) | 0.500 (0.500, 0.500) | 0.500 (0.500, 0.500) | 0.609 | 0.516 | 0.756 (±0.0112) | 0.835 (±0.0019) | ✓\| | ✓\| |  |  |  |
| │ │ ├── CliXO:076 (INPP5F, DYNLL1) | 0.500 (0.500, 0.500) | 0.500 (0.500, 0.500) | 0.609 | 0.516 | 0.756 (±0.0112) | 0.835 (±0.0019) | ✓\| | ✓\| |  |  |  |
| │ │ └── CliXO:085 (RDX, AZIN1, DYNLL1, TRIM68) | 0.500 (0.500, 0.500) | 0.500 (0.500, 0.500) | 0.609 | 0.516 | 0.756 (±0.0112) | 0.835 (±0.0019) | ✓\| | ✓\| |  |  |  |
| │ │ ├── CliXO:074 (RDX, AZIN1) | 0.500 (0.500, 0.500) | 0.500 (0.500, 0.500) | 0.609 | 0.516 | 0.756 (±0.0112) | 0.835 (±0.0019) | ✓\| | ✓\| |  |  |  |
| │ └── CliXO:096 (BMP2, FAM172A, PKM, INPP5F, EFNB1, P2RY14, SH3PXD2A, CDO1, MCAT, DYNLL1, SYDE1) | 0.500 (0.500, 0.500) | 0.500 (0.500, 0.500) | 0.609 | 0.516 | 0.756 (±0.0112) | 0.835 (±0.0019) | ✓\| | ✓\| |  |  |  |
| │ │ └── CliXO:077 (PKM, MCAT) | 0.500 (0.500, 0.500) | 0.500 (0.500, 0.500) | 0.609 | 0.516 | 0.756 (±0.0112) | 0.835 (±0.0019) | ✓\| | ✓\| |  |  |  |
| │ └── CliXO:089 (BMP2, INPP5F, P2RY14, CDO1, DYNLL1, SYDE1) | 0.500 (0.500, 0.500) | 0.500 (0.500, 0.500) | 0.609 | 0.516 | 0.756 (±0.0112) | 0.835 (±0.0019) | ✓\| | ✓\| |  |  |  |
| │ │ ├── CliXO:076 (INPP5F, DYNLL1) | 0.500 (0.500, 0.500) | 0.500 (0.500, 0.500) | 0.609 | 0.516 | 0.756 (±0.0112) | 0.835 (±0.0019) | ✓\| | ✓\| |  |  |  |
| └── CliXO:110 (ICA1, PLIN2, DOCK8, GDA, NFIL3, CCDC127, CORO2A, BCAR3, SPAG4, ANKRD37, CLIP1, ANKRD13A, TUFT1, KRT19, SMPDL3A, NPR3, CTBS, GRHL1, LDLR, MYC, ASAH1, ZNF555, TIAM1, SCYL3, GPT2, TOR1B, PIK3R1, ERO1A, APPBP2, EDEM1, NECTIN4, DDIT4, NEDD4L, KLF3, GMFB, SLCO4A1, P4HA1, ERRFI1, ETS2, CEBPD, GULP1, RAPGEF3, FLNB, GPX3, DEGS1) | 0.500 (0.500, 0.500) | 0.500 (0.500, 0.500) | 0.609 | 0.516 | 0.756 (±0.0112) | 0.835 (±0.0019) | ✓\| | ✓\| |  |  |  |
| ├── CliXO:101 (DOCK8, CCDC127, ANKRD13A, SMPDL3A, GRHL1, ASAH1, ZNF555, TIAM1, SCYL3, TOR1B, PIK3R1, EDEM1, NEDD4L, KLF3, GMFB, SLCO4A1, GULP1, RAPGEF3, FLNB, GPX3, DEGS1) | 0.500 (0.500, 0.500) | 0.500 (0.500, 0.500) | 0.609 | 0.516 | 0.756 (±0.0112) | 0.835 (±0.0019) | ✓\| | ✓\| |  |  |  |
| │ ├── CliXO:073 (GRHL1, NEDD4L) | 0.500 (0.500, 0.500) | 0.500 (0.500, 0.500) | 0.609 | 0.516 | 0.756 (±0.0112) | 0.835 (±0.0019) | ✓\| | ✓\| |  |  |  |
| │ ├── CliXO:078 (CCDC127, TIAM1) | 0.500 (0.500, 0.500) | 0.500 (0.500, 0.500) | 0.609 | 0.516 | 0.756 (±0.0112) | 0.835 (±0.0019) | ✓\| | ✓\| |  |  |  |
| │ ├── CliXO:079 (KLF3, DEGS1) | 0.500 (0.500, 0.500) | 0.500 (0.500, 0.500) | 0.609 | 0.516 | 0.756 (±0.0112) | 0.835 (±0.0019) | ✓\| | ✓\| |  |  |  |
| │ ├── CliXO:081 (SCYL3, GMFB) | 0.500 (0.500, 0.500) | 0.500 (0.500, 0.500) | 0.609 | 0.516 | 0.756 (±0.0112) | 0.835 (±0.0019) | ✓\| | ✓\| |  |  |  |
| │ └── CliXO:095 (DOCK8, CCDC127, SMPDL3A, ASAH1, ZNF555, PIK3R1, GMFB, RAPGEF3, FLNB, GPX3) | 0.500 (0.500, 0.500) | 0.500 (0.500, 0.500) | 0.609 | 0.516 | 0.756 (±0.0112) | 0.835 (±0.0019) | ✓\| | ✓\| |  |  |  |
| │ └── CliXO:090 (DOCK8, CCDC127, ASAH1, PIK3R1, RAPGEF3, GPX3) | 0.500 (0.500, 0.500) | 0.500 (0.500, 0.500) | 0.609 | 0.516 | 0.756 (±0.0112) | 0.835 (±0.0019) | ✓\| | ✓\| |  |  |  |
| │ └── CliXO:083 (PIK3R1, RAPGEF3, GPX3) | 0.500 (0.500, 0.500) | 0.500 (0.500, 0.500) | 0.609 | 0.516 | 0.756 (±0.0112) | 0.835 (±0.0019) | ✓\| | ✓\| |  |  |  |
| └── CliXO:109 (ICA1, PLIN2, DOCK8, GDA, NFIL3, CORO2A, BCAR3, SPAG4, ANKRD37, CLIP1, ANKRD13A, TUFT1, KRT19, SMPDL3A, NPR3, CTBS, GRHL1, LDLR, MYC, ASAH1, ZNF555, SCYL3, GPT2, TOR1B, PIK3R1, ERO1A, APPBP2, EDEM1, NECTIN4, DDIT4, NEDD4L, KLF3, GMFB, SLCO4A1, P4HA1, ERRFI1, ETS2, CEBPD, GULP1, RAPGEF3, FLNB, GPX3, DEGS1) | 0.500 (0.500, 0.500) | 0.500 (0.500, 0.500) | 0.609 | 0.516 | 0.756 (±0.0112) | 0.835 (±0.0019) | ✓\| | ✓\| |  |  |  |
| ├── CliXO:094 (PLIN2, GDA, ANKRD37, KRT19, NPR3, LDLR, GPT2, NECTIN4, DDIT4) | 0.500 (0.500, 0.500) | 0.500 (0.500, 0.500) | 0.609 | 0.516 | 0.756 (±0.0112) | 0.835 (±0.0019) | ✓\| | ✓\| |  |  |  |
| │ └── CliXO:084 (PLIN2, ANKRD37, NPR3) | 0.500 (0.500, 0.500) | 0.500 (0.500, 0.500) | 0.609 | 0.516 | 0.756 (±0.0112) | 0.835 (±0.0019) | ✓\| | ✓\| |  |  |  |
| └── CliXO:108 (ICA1, PLIN2, DOCK8, GDA, NFIL3, CORO2A, BCAR3, SPAG4, ANKRD37, CLIP1, ANKRD13A, TUFT1, KRT19, SMPDL3A, NPR3, CTBS, GRHL1, ASAH1, ZNF555, SCYL3, GPT2, TOR1B, PIK3R1, ERO1A, APPBP2, EDEM1, NEDD4L, KLF3, GMFB, SLCO4A1, P4HA1, ERRFI1, ETS2, CEBPD, GULP1, RAPGEF3, FLNB, GPX3, DEGS1) | 0.500 (0.500, 0.500) | 0.500 (0.500, 0.500) | 0.609 | 0.516 | 0.756 (±0.0112) | 0.835 (±0.0019) | ✓\| | ✓\| |  |  |  |
| ├── CliXO:099 (ICA1, DOCK8, CLIP1, ANKRD13A, SMPDL3A, CTBS, GRHL1, ASAH1, ZNF555, SCYL3, TOR1B, PIK3R1, APPBP2, NEDD4L, KLF3, GMFB, RAPGEF3, FLNB, GPX3) | 0.500 (0.500, 0.500) | 0.500 (0.500, 0.500) | 0.609 | 0.516 | 0.756 (±0.0112) | 0.835 (±0.0019) | ✓\| | ✓\| |  |  |  |
| │ ├── CliXO:073 (GRHL1, NEDD4L) | 0.500 (0.500, 0.500) | 0.500 (0.500, 0.500) | 0.609 | 0.516 | 0.756 (±0.0112) | 0.835 (±0.0019) | ✓\| | ✓\| |  |  |  |
| │ ├── CliXO:081 (SCYL3, GMFB) | 0.500 (0.500, 0.500) | 0.500 (0.500, 0.500) | 0.609 | 0.516 | 0.756 (±0.0112) | 0.835 (±0.0019) | ✓\| | ✓\| |  |  |  |
| │ └── CliXO:083 (PIK3R1, RAPGEF3, GPX3) | 0.500 (0.500, 0.500) | 0.500 (0.500, 0.500) | 0.609 | 0.516 | 0.756 (±0.0112) | 0.835 (±0.0019) | ✓\| | ✓\| |  |  |  |
| │ └── CliXO:091 (DOCK8, SMPDL3A, ZNF555, PIK3R1, APPBP2, KLF3, GPX3) | 0.500 (0.500, 0.500) | 0.500 (0.500, 0.500) | 0.609 | 0.516 | 0.756 (±0.0112) | 0.835 (±0.0019) | ✓\| | ✓\| |  |  |  |
| └── CliXO:106 (ICA1, PLIN2, GDA, NFIL3, CORO2A, BCAR3, SPAG4, ANKRD37, CLIP1, ANKRD13A, TUFT1, KRT19, NPR3, CTBS, GRHL1, GPT2, PIK3R1, ERO1A, APPBP2, EDEM1, KLF3, GMFB, SLCO4A1, P4HA1, ERRFI1, ETS2, CEBPD, GULP1, RAPGEF3, FLNB, GPX3, DEGS1) | 0.500 (0.500, 0.500) | 0.500 (0.500, 0.500) | 0.609 | 0.516 | 0.756 (±0.0112) | 0.835 (±0.0019) | ✓\| | ✓\| |  |  |  |
| │ └── CliXO:083 (PIK3R1, RAPGEF3, GPX3) | 0.500 (0.500, 0.500) | 0.500 (0.500, 0.500) | 0.609 | 0.516 | 0.756 (±0.0112) | 0.835 (±0.0019) | ✓\| | ✓\| |  |  |  |
| │ └── CliXO:084 (PLIN2, ANKRD37, NPR3) | 0.500 (0.500, 0.500) | 0.500 (0.500, 0.500) | 0.609 | 0.516 | 0.756 (±0.0112) | 0.835 (±0.0019) | ✓\| | ✓\| |  |  |  |
| └── CliXO:103 (ICA1, PLIN2, GDA, NFIL3, CORO2A, BCAR3, CLIP1, ANKRD13A, TUFT1, KRT19, NPR3, GRHL1, GPT2, ERO1A, KLF3, GMFB, SLCO4A1, P4HA1, ERRFI1, ETS2, CEBPD, RAPGEF3, FLNB, GPX3, DEGS1) | 0.500 (0.500, 0.500) | 0.500 (0.500, 0.500) | 0.609 | 0.516 | 0.756 (±0.0112) | 0.835 (±0.0019) | ✓\| | ✓\| |  |  |  |
| │ ├── CliXO:079 (KLF3, DEGS1) | 0.500 (0.500, 0.500) | 0.500 (0.500, 0.500) | 0.609 | 0.516 | 0.756 (±0.0112) | 0.835 (±0.0019) | ✓\| | ✓\| |  |  |  |
| └── CliXO:102 (ICA1, PLIN2, GDA, NFIL3, CORO2A, CLIP1, ANKRD13A, TUFT1, KRT19, NPR3, GRHL1, GPT2, ERO1A, SLCO4A1, P4HA1, ERRFI1, ETS2, CEBPD, FLNB, GPX3, DEGS1) | 0.500 (0.500, 0.500) | 0.500 (0.500, 0.500) | 0.609 | 0.516 | 0.756 (±0.0112) | 0.835 (±0.0019) | ✓\| | ✓\| |  |  |  |
| └── CliXO:097 (PLIN2, NFIL3, CORO2A, CLIP1, TUFT1, ERO1A, SLCO4A1, ETS2, CEBPD, FLNB, GPX3, DEGS1) | 0.500 (0.500, 0.500) | 0.500 (0.500, 0.500) | 0.609 | 0.516 | 0.756 (±0.0112) | 0.835 (±0.0019) | ✓\| | ✓\| |  |  |  |

The Reported column indicates the ontology from Tables 1–2 to which this row’s eligible genes belong. An ontology is reported if it passes all criteria (C1–C4) and its eligible gene set is not a strict subset of another passing ontology’s gene set. The column is empty when the ontology has no eligible genes or does not pass all criteria. ✓, the ontology passed the criterion; †, the gene had a grad-CAM value >97.5th percentile in the same ontology; |, separator between PPV/TPR and NPV/TNR results in C1 and C2 columns; AUC-PRC, area under the precision-recall curve; AUC-ROC, area under the receiver operating characteristic curve; C1, lower bounds finite and >0; C2, absolute difference ≤25% between development and test sets; C3, at least 1 eligible gene; C4, lower bound of AUC-ROC >0.5 in both sets; CI, confidence interval; Dev, development set; grad-CAM, gradient-weighted class activation mapping; HELLP, hemolysis, elevated liver enzyme, and low platelet; PE, preeclampsia; RMSE, root mean square error.

Table S7. Threshold-specific confusion matrices for the late-onset, severe PE pathogenesis model connected with third-trimester placenta under chorioamnionitis.

| Ontology | PPV/TPR (Dev) | PPV/TPR (Test) | NPV/TNR (Dev) | NPV/TNR (Test) | C1 | C2 | C3 | C4 | Reported |
| --- | --- | --- | --- | --- | --- | --- | --- | --- | --- |
| root (CDO1, PLIN2, CCSAP, BCAR3, DOCK8, SPAG4, BHLHE40†, NECTIN4, SIGLEC6, PTPRD, SFXN3, LDLR, CORO2A, CCDC6, TACC1, SELL, NEDD4L, SLCO4A1, SPON1, PFKFB3, GDA, APLN, IGF1, PIK3R1, SLC11A1, SLA, KRT19, SYDE1, ERRFI1, TUBB4B, SMPDL3A, GRHL1, PAK2, RBM22, CEBPA, ANKRD13A†, GBE1, FAP, GPR146, HINT3, CCDC127, ZNF555, ICA1, CHMP2B, MYC, CA4, LEP, EFNB1, P4HA1, SH3PXD2A, ZMYND8, ASAH1, RAPGEF3, SLCO2A1, ZEB2, GPT2, ERO1A, ANKRD37) | 22.9% (20.2, 25.6) / 100.0% (100.0, 100.0) | 3.0% (2.6, 3.4) / 100.0% (100.0, 100.0) | — / 0.0% (0.0, 0.0) | — / 0.0% (0.0, 0.0) | ✓\| | ✓\| | ✓ |  | root |
| CliXO:86 (CDO1, PLIN2, CCSAP, BCAR3, DOCK8, SPAG4, BHLHE40, NECTIN4, SIGLEC6, PTPRD, SFXN3, LDLR, CORO2A, CCDC6, TACC1, SELL, NEDD4L, SLCO4A1, SPON1, PFKFB3, GDA, APLN, IGF1, PIK3R1, SLC11A1, SLA†, KRT19, SYDE1, ERRFI1, TUBB4B, SMPDL3A, GRHL1, PAK2, RBM22, CEBPA, ANKRD13A†, GBE1, FAP, GPR146, HINT3, CCDC127, ZNF555, ICA1, CHMP2B, MYC, CA4, LEP, EFNB1, P4HA1, SH3PXD2A, ZMYND8, ASAH1, RAPGEF3, SLCO2A1, ZEB2, GPT2, ERO1A, ANKRD37) | 22.9% (20.2, 25.6) / 100.0% (100.0, 100.0) | 3.0% (2.6, 3.4) / 100.0% (100.0, 100.0) | — / 0.0% (0.0, 0.0) | — / 0.0% (0.0, 0.0) | ✓\| | ✓\| | ✓ | ✓ | CliXO:86 |
| ├── CliXO:73 (DOCK8, GDA, ANKRD13A, HINT3, CHMP2B) | 22.9% (20.2, 25.6) / 100.0% (100.0, 100.0) | 3.0% (2.6, 3.4) / 100.0% (100.0, 100.0) | — / 0.0% (0.0, 0.0) | — / 0.0% (0.0, 0.0) | ✓\| | ✓\| |  | ✓ | CliXO:86 |
| │ ├── CliXO:59 (GDA, ANKRD13A) | 22.9% (20.2, 25.6) / 100.0% (100.0, 100.0) | 3.0% (2.6, 3.4) / 100.0% (100.0, 100.0) | — / 0.0% (0.0, 0.0) | — / 0.0% (0.0, 0.0) | ✓\| | ✓\| |  | ✓ | CliXO:86 |
| │ └── CliXO:64 (HINT3, CHMP2B) | 22.9% (20.2, 25.6) / 100.0% (100.0, 100.0) | 3.0% (2.6, 3.4) / 100.0% (100.0, 100.0) | — / 0.0% (0.0, 0.0) | — / 0.0% (0.0, 0.0) | ✓\| | ✓\| |  |  |  |
| └── CliXO:85 (CDO1, PLIN2, CCSAP, BCAR3, DOCK8, SPAG4, BHLHE40, NECTIN4, SIGLEC6, PTPRD, SFXN3, LDLR, CORO2A, CCDC6, TACC1, SELL, NEDD4L, SLCO4A1, SPON1, PFKFB3, APLN, IGF1, PIK3R1, SLC11A1, SLA, KRT19, SYDE1, ERRFI1, TUBB4B, SMPDL3A, GRHL1, PAK2, RBM22, CEBPA, GBE1, FAP, GPR146, CCDC127, ZNF555, ICA1, MYC, CA4, LEP, EFNB1, P4HA1, SH3PXD2A, ZMYND8, ASAH1, RAPGEF3, SLCO2A1, ZEB2, GPT2, ERO1A, ANKRD37) | 100.0% (100.0, 100.0) / 70.3% (65.3, 75.4) | 3.0% (2.6, 3.4) / 100.0% (100.0, 100.0) | 92.0% (90.5, 93.5) / 100.0% (100.0, 100.0) | — / 0.0% (0.0, 0.0) | ✓\| | \| |  |  |  |
| ├── CliXO:74 (TACC1, SYDE1, EFNB1, SH3PXD2A, ZMYND8, RAPGEF3) | 22.9% (20.2, 25.6) / 100.0% (100.0, 100.0) | 3.0% (2.6, 3.4) / 100.0% (100.0, 100.0) | — / 0.0% (0.0, 0.0) | — / 0.0% (0.0, 0.0) | ✓\| | ✓\| |  |  |  |
| ├── CliXO:75 (BHLHE40, PFKFB3, SLC11A1, GPR146, P4HA1, ZMYND8, ERO1A) | 22.9% (20.2, 25.6) / 100.0% (100.0, 100.0) | 3.0% (2.6, 3.4) / 100.0% (100.0, 100.0) | — / 0.0% (0.0, 0.0) | — / 0.0% (0.0, 0.0) | ✓\| | ✓\| |  |  |  |
| │ └── CliXO:58 (P4HA1, ERO1A) | 22.9% (20.2, 25.6) / 100.0% (100.0, 100.0) | 3.0% (2.6, 3.4) / 100.0% (100.0, 100.0) | — / 0.0% (0.0, 0.0) | — / 0.0% (0.0, 0.0) | ✓\| | ✓\| |  |  |  |
| ├── CliXO:81 (DOCK8, PTPRD, LDLR, CCDC6, SELL, NEDD4L, SPON1, PFKFB3, APLN, IGF1, SLC11A1, SLA, ERRFI1, TUBB4B, SMPDL3A, PAK2, FAP, ZNF555, ICA1, MYC, ASAH1, ZEB2) | 26.9% (23.6, 30.3) / 88.2% (84.6, 91.7) | 3.0% (2.6, 3.4) / 100.0% (100.0, 100.0) | 87.8% (84.1, 91.6) / 28.9% (25.6, 32.3) | — / 0.0% (0.0, 0.0) | ✓\| | ✓\| |  |  | CliXO:86 |
| │ ├── CliXO:61 (SPON1, TUBB4B) | 22.9% (20.2, 25.6) / 100.0% (100.0, 100.0) | 3.0% (2.6, 3.4) / 100.0% (100.0, 100.0) | — / 0.0% (0.0, 0.0) | — / 0.0% (0.0, 0.0) | ✓\| | ✓\| |  |  |  |
| │ ├── CliXO:66 (LDLR, TUBB4B, MYC) | 22.9% (20.2, 25.6) / 100.0% (100.0, 100.0) | 3.0% (2.6, 3.4) / 100.0% (100.0, 100.0) | — / 0.0% (0.0, 0.0) | — / 0.0% (0.0, 0.0) | ✓\| | ✓\| |  |  |  |
| │ ├── CliXO:70 (CCDC6, SMPDL3A, PAK2, ASAH1) | 22.9% (20.2, 25.6) / 100.0% (100.0, 100.0) | 3.0% (2.6, 3.4) / 100.0% (100.0, 100.0) | — / 0.0% (0.0, 0.0) | — / 0.0% (0.0, 0.0) | ✓\| | ✓\| |  |  |  |
| │ │ └── CliXO:60 (CCDC6, PAK2) | 22.9% (20.2, 25.6) / 100.0% (100.0, 100.0) | 3.0% (2.6, 3.4) / 100.0% (100.0, 100.0) | — / 0.0% (0.0, 0.0) | — / 0.0% (0.0, 0.0) | ✓\| | ✓\| |  |  |  |
| │ ├── CliXO:71 (SLC11A1, SLA, FAP, MYC) | 22.9% (20.2, 25.6) / 100.0% (100.0, 100.0) | 3.0% (2.6, 3.4) / 100.0% (100.0, 100.0) | — / 0.0% (0.0, 0.0) | — / 0.0% (0.0, 0.0) | ✓\| | ✓\| |  |  | CliXO:86 |
| │ │ └── CliXO:62 (SLA, MYC) | 22.9% (20.2, 25.6) / 100.0% (100.0, 100.0) | 3.0% (2.6, 3.4) / 100.0% (100.0, 100.0) | — / 0.0% (0.0, 0.0) | — / 0.0% (0.0, 0.0) | ✓\| | ✓\| |  |  | CliXO:86 |
| │ └── CliXO:78 (DOCK8, PTPRD, NEDD4L, SPON1, APLN, IGF1†, PAK2, FAP, ZNF555, ICA1, ASAH1, ZEB2) | 22.9% (20.2, 25.6) / 100.0% (100.0, 100.0) | 3.0% (2.6, 3.4) / 100.0% (100.0, 100.0) | — / 0.0% (0.0, 0.0) | — / 0.0% (0.0, 0.0) | ✓\| | ✓\| | ✓ |  | CliXO:78 |
| │ ├── CliXO:63 (ZNF555, ZEB2) | 22.9% (20.2, 25.6) / 100.0% (100.0, 100.0) | 3.0% (2.6, 3.4) / 100.0% (100.0, 100.0) | — / 0.0% (0.0, 0.0) | — / 0.0% (0.0, 0.0) | ✓\| | ✓\| |  |  |  |
| │ ├── CliXO:68 (NEDD4L, PAK2, ASAH1) | 22.9% (20.2, 25.6) / 100.0% (100.0, 100.0) | 3.0% (2.6, 3.4) / 100.0% (100.0, 100.0) | — / 0.0% (0.0, 0.0) | — / 0.0% (0.0, 0.0) | ✓\| | ✓\| |  |  |  |
| │ └── CliXO:77 (DOCK8, PTPRD, SPON1, APLN, IGF1, PAK2, ICA1, ASAH1, ZEB2) | 22.9% (20.2, 25.6) / 100.0% (100.0, 100.0) | 3.0% (2.6, 3.4) / 100.0% (100.0, 100.0) | — / 0.0% (0.0, 0.0) | — / 0.0% (0.0, 0.0) | ✓\| | ✓\| |  |  | CliXO:78 |
| │ └── CliXO:76 (DOCK8, PTPRD, SPON1, APLN, IGF1, PAK2, ASAH1, ZEB2) | 22.9% (20.2, 25.6) / 100.0% (100.0, 100.0) | 3.0% (2.6, 3.4) / 100.0% (100.0, 100.0) | — / 0.0% (0.0, 0.0) | — / 0.0% (0.0, 0.0) | ✓\| | ✓\| |  |  | CliXO:78 |
| │ └── CliXO:69 (PTPRD, SPON1, IGF1, ZEB2) | 22.9% (20.2, 25.6) / 100.0% (100.0, 100.0) | 3.0% (2.6, 3.4) / 100.0% (100.0, 100.0) | — / 0.0% (0.0, 0.0) | — / 0.0% (0.0, 0.0) | ✓\| | ✓\| |  |  | CliXO:78 |
| └── CliXO:84 (CDO1, PLIN2, CCSAP, BCAR3, SPAG4, BHLHE40, NECTIN4, SIGLEC6, SFXN3, CORO2A, TACC1, SLCO4A1, PIK3R1, KRT19, SYDE1, ERRFI1, GRHL1, CEBPA, GBE1, GPR146, CCDC127, CA4, LEP, EFNB1, P4HA1, SH3PXD2A, ZMYND8, SLCO2A1, GPT2, ERO1A, ANKRD37) | 22.9% (20.2, 25.6) / 100.0% (100.0, 100.0) | 3.0% (2.6, 3.4) / 100.0% (100.0, 100.0) | — / 0.0% (0.0, 0.0) | — / 0.0% (0.0, 0.0) | ✓\| | ✓\| |  |  | root |
| └── CliXO:83 (CDO1, PLIN2, CCSAP, BCAR3, SPAG4, BHLHE40, NECTIN4, SIGLEC6, SFXN3, CORO2A, TACC1, SLCO4A1, PIK3R1, KRT19, SYDE1, ERRFI1, GRHL1, CEBPA, GBE1, GPR146, CCDC127, LEP, EFNB1, P4HA1, SH3PXD2A, ZMYND8, SLCO2A1, GPT2, ERO1A, ANKRD37) | 22.9% (20.2, 25.6) / 100.0% (100.0, 100.0) | 3.0% (2.6, 3.4) / 100.0% (100.0, 100.0) | — / 0.0% (0.0, 0.0) | — / 0.0% (0.0, 0.0) | ✓\| | ✓\| |  |  | root |
| └── CliXO:82 (CDO1, PLIN2, CCSAP, BCAR3, SPAG4, BHLHE40, NECTIN4, SIGLEC6, SFXN3, CORO2A, KRT19, SYDE1, ERRFI1, GRHL1, GPR146, LEP, EFNB1, P4HA1, SH3PXD2A, ZMYND8, SLCO2A1, GPT2, ERO1A, ANKRD37) | 22.9% (20.2, 25.6) / 100.0% (100.0, 100.0) | 3.0% (2.6, 3.4) / 100.0% (100.0, 100.0) | — / 0.0% (0.0, 0.0) | — / 0.0% (0.0, 0.0) | ✓\| | ✓\| |  |  | root |
| ├── CliXO:72 (KRT19, LEP, SH3PXD2A, SLCO2A1) | 22.9% (20.2, 25.6) / 100.0% (100.0, 100.0) | 3.0% (2.6, 3.4) / 100.0% (100.0, 100.0) | — / 0.0% (0.0, 0.0) | — / 0.0% (0.0, 0.0) | ✓\| | ✓\| |  |  |  |
| └── CliXO:80 (PLIN2, CCSAP, SPAG4, BHLHE40, NECTIN4, SIGLEC6, SFXN3, CORO2A, KRT19, SYDE1, ERRFI1, GRHL1, GPR146, LEP, EFNB1, P4HA1, SH3PXD2A, GPT2, ERO1A, ANKRD37) | 22.9% (20.2, 25.6) / 100.0% (100.0, 100.0) | 3.0% (2.6, 3.4) / 100.0% (100.0, 100.0) | — / 0.0% (0.0, 0.0) | — / 0.0% (0.0, 0.0) | ✓\| | ✓\| |  |  | root |
| ├── CliXO:65 (BHLHE40, ANKRD37) | 22.9% (20.2, 25.6) / 100.0% (100.0, 100.0) | 3.0% (2.6, 3.4) / 100.0% (100.0, 100.0) | — / 0.0% (0.0, 0.0) | — / 0.0% (0.0, 0.0) | ✓\| | ✓\| |  |  | root |
| └── CliXO:79 (PLIN2, CCSAP, SPAG4, BHLHE40, NECTIN4, SFXN3, CORO2A, KRT19, SYDE1, GRHL1, GPR146, LEP, P4HA1, SH3PXD2A, GPT2, ERO1A) | 22.9% (20.2, 25.6) / 100.0% (100.0, 100.0) | 3.0% (2.6, 3.4) / 100.0% (100.0, 100.0) | — / 0.0% (0.0, 0.0) | — / 0.0% (0.0, 0.0) | ✓\| | ✓\| |  |  |  |
| └── CliXO:67 (NECTIN4, SFXN3, ERO1A) | 22.9% (20.2, 25.6) / 100.0% (100.0, 100.0) | 3.0% (2.6, 3.4) / 100.0% (100.0, 100.0) | — / 0.0% (0.0, 0.0) | — / 0.0% (0.0, 0.0) | ✓\| | ✓\| |  |  |  |

The Reported column indicates the ontology from Tables 1–2 to which this row’s eligible genes belong. An ontology is reported if it passes all criteria (C1–C4) and its eligible gene set is not a strict subset of another passing ontology’s gene set. The column is empty when the ontology has no eligible genes or does not pass all criteria. ✓, the ontology passed the criterion; †, the gene had a grad-CAM value >97.5th percentile in the same ontology; |, separator between PPV/TPR and NPV/TNR results in C1 and C2 columns; AUC-ROC, area under the receiver operating characteristic curve; C1, lower bounds finite and >0; C2, absolute difference ≤25% between development and test sets; C3, at least 1 eligible gene; C4, lower bound of AUC-ROC >0.5 in both sets; CI, confidence interval; Dev, development set; grad-CAM, gradient-weighted class activation mapping; HELLP, hemolysis, elevated liver enzyme, and low platelet; NPV, negative predictive value; PE, preeclampsia; PPV, positive predictive value; TNR, true negative rate; TPR, true positive rate.

Table S8. AUC-ROC, AUC-PRC, and RMSE for the late-onset, severe PE pathogenesis model connected with third-trimester placenta under chorioamnionitis.

| Ontology | AUC-ROC (Dev) | AUC-ROC (Test) | AUC-PRC (Dev) | AUC-PRC (Test) | RMSE (Dev) | RMSE (Test) | C1 | C2 | C3 | C4 | Reported |
| --- | --- | --- | --- | --- | --- | --- | --- | --- | --- | --- | --- |
| root (CDO1, PLIN2, CCSAP, BCAR3, DOCK8, SPAG4, BHLHE40†, NECTIN4, SIGLEC6, PTPRD, SFXN3, LDLR, CORO2A, CCDC6, TACC1, SELL, NEDD4L, SLCO4A1, SPON1, PFKFB3, GDA, APLN, IGF1, PIK3R1, SLC11A1, SLA, KRT19, SYDE1, ERRFI1, TUBB4B, SMPDL3A, GRHL1, PAK2, RBM22, CEBPA, ANKRD13A†, GBE1, FAP, GPR146, HINT3, CCDC127, ZNF555, ICA1, CHMP2B, MYC, CA4, LEP, EFNB1, P4HA1, SH3PXD2A, ZMYND8, ASAH1, RAPGEF3, SLCO2A1, ZEB2, GPT2, ERO1A, ANKRD37) | 0.199 (0.168, 0.231) | 0.275 (0.267, 0.283) | 0.088 | 0.016 | 0.669 (±0.0104) | 0.533 (±0.0070) | ✓\| | ✓\| | ✓ |  | root |
| CliXO:86 (CDO1, PLIN2, CCSAP, BCAR3, DOCK8, SPAG4, BHLHE40, NECTIN4, SIGLEC6, PTPRD, SFXN3, LDLR, CORO2A, CCDC6, TACC1, SELL, NEDD4L, SLCO4A1, SPON1, PFKFB3, GDA, APLN, IGF1, PIK3R1, SLC11A1, SLA†, KRT19, SYDE1, ERRFI1, TUBB4B, SMPDL3A, GRHL1, PAK2, RBM22, CEBPA, ANKRD13A†, GBE1, FAP, GPR146, HINT3, CCDC127, ZNF555, ICA1, CHMP2B, MYC, CA4, LEP, EFNB1, P4HA1, SH3PXD2A, ZMYND8, ASAH1, RAPGEF3, SLCO2A1, ZEB2, GPT2, ERO1A, ANKRD37) | 0.783 (0.745, 0.821) | 0.725 (0.717, 0.733) | 0.318 | 0.516 | 0.518 (±0.0039) | 0.555 (±0.0010) | ✓\| | ✓\| | ✓ | ✓ | CliXO:86 |
| ├── CliXO:73 (DOCK8, GDA, ANKRD13A, HINT3, CHMP2B) | 0.810 (0.782, 0.839) | 0.725 (0.717, 0.733) | 0.675 | 0.516 | 0.545 (±0.0051) | 0.598 (±0.0011) | ✓\| | ✓\| |  | ✓ | CliXO:86 |
| │ ├── CliXO:59 (GDA, ANKRD13A) | 0.810 (0.782, 0.839) | 0.725 (0.717, 0.733) | 0.476 | 0.516 | 0.566 (±0.0085) | 0.666 (±0.0025) | ✓\| | ✓\| |  | ✓ | CliXO:86 |
| │ └── CliXO:64 (HINT3, CHMP2B) | 0.500 (0.500, 0.500) | 0.500 (0.500, 0.500) | 0.609 | 0.516 | 0.875 (±0.0154) | 0.982 (±0.0020) | ✓\| | ✓\| |  |  |  |
| └── CliXO:85 (CDO1, PLIN2, CCSAP, BCAR3, DOCK8, SPAG4, BHLHE40, NECTIN4, SIGLEC6, PTPRD, SFXN3, LDLR, CORO2A, CCDC6, TACC1, SELL, NEDD4L, SLCO4A1, SPON1, PFKFB3, APLN, IGF1, PIK3R1, SLC11A1, SLA, KRT19, SYDE1, ERRFI1, TUBB4B, SMPDL3A, GRHL1, PAK2, RBM22, CEBPA, GBE1, FAP, GPR146, CCDC127, ZNF555, ICA1, MYC, CA4, LEP, EFNB1, P4HA1, SH3PXD2A, ZMYND8, ASAH1, RAPGEF3, SLCO2A1, ZEB2, GPT2, ERO1A, ANKRD37) | 0.693 (0.656, 0.730) | 0.500 (0.500, 0.500) | 0.376 | 0.516 | 0.535 (±0.0084) | 0.649 (±0.0010) | ✓\| | \| |  |  |  |
| ├── CliXO:74 (TACC1, SYDE1, EFNB1, SH3PXD2A, ZMYND8, RAPGEF3) | 0.500 (0.500, 0.500) | 0.500 (0.500, 0.500) | 0.609 | 0.516 | 0.875 (±0.0154) | 0.982 (±0.0020) | ✓\| | ✓\| |  |  |  |
| ├── CliXO:75 (BHLHE40, PFKFB3, SLC11A1, GPR146, P4HA1, ZMYND8, ERO1A) | 0.208 (0.181, 0.234) | 0.500 (0.500, 0.500) | 0.088 | 0.516 | 0.679 (±0.0102) | 0.214 (±0.0060) | ✓\| | ✓\| |  |  |  |
| │ └── CliXO:58 (P4HA1, ERO1A) | 0.500 (0.500, 0.500) | 0.500 (0.500, 0.500) | 0.609 | 0.516 | 0.875 (±0.0154) | 0.982 (±0.0020) | ✓\| | ✓\| |  |  |  |
| ├── CliXO:81 (DOCK8, PTPRD, LDLR, CCDC6, SELL, NEDD4L, SPON1, PFKFB3, APLN, IGF1, SLC11A1, SLA, ERRFI1, TUBB4B, SMPDL3A, PAK2, FAP, ZNF555, ICA1, MYC, ASAH1, ZEB2) | 0.563 (0.525, 0.602) | 0.500 (0.500, 0.500) | 0.102 | 0.516 | 0.526 (±0.0139) | 0.623 (±0.0008) | ✓\| | ✓\| |  |  | CliXO:86 |
| │ ├── CliXO:61 (SPON1, TUBB4B) | 0.500 (0.500, 0.500) | 0.500 (0.500, 0.500) | 0.609 | 0.516 | 0.875 (±0.0154) | 0.982 (±0.0020) | ✓\| | ✓\| |  |  |  |
| │ ├── CliXO:66 (LDLR, TUBB4B, MYC) | 0.500 (0.500, 0.500) | 0.500 (0.500, 0.500) | 0.609 | 0.516 | 0.875 (±0.0154) | 0.982 (±0.0020) | ✓\| | ✓\| |  |  |  |
| │ ├── CliXO:70 (CCDC6, SMPDL3A, PAK2, ASAH1) | 0.500 (0.500, 0.500) | 0.500 (0.500, 0.500) | 0.609 | 0.516 | 0.875 (±0.0154) | 0.982 (±0.0020) | ✓\| | ✓\| |  |  |  |
| │ │ └── CliXO:60 (CCDC6, PAK2) | 0.500 (0.500, 0.500) | 0.500 (0.500, 0.500) | 0.609 | 0.516 | 0.875 (±0.0154) | 0.982 (±0.0020) | ✓\| | ✓\| |  |  |  |
| │ ├── CliXO:71 (SLC11A1, SLA, FAP, MYC) | 0.856 (0.829, 0.883) | 0.500 (0.500, 0.500) | 0.504 | 0.516 | 0.748 (±0.0129) | 0.865 (±0.0017) | ✓\| | ✓\| |  |  | CliXO:86 |
| │ │ └── CliXO:62 (SLA, MYC) | 0.500 (0.500, 0.500) | 0.500 (0.500, 0.500) | 0.609 | 0.516 | 0.752 (±0.0127) | 0.842 (±0.0017) | ✓\| | ✓\| |  |  | CliXO:86 |
| │ └── CliXO:78 (DOCK8, PTPRD, NEDD4L, SPON1, APLN, IGF1†, PAK2, FAP, ZNF555, ICA1, ASAH1, ZEB2) | 0.217 (0.184, 0.250) | 0.500 (0.500, 0.500) | 0.088 | 0.516 | 0.616 (±0.0115) | 0.206 (±0.0066) | ✓\| | ✓\| | ✓ |  | CliXO:78 |
| │ ├── CliXO:63 (ZNF555, ZEB2) | 0.500 (0.500, 0.500) | 0.500 (0.500, 0.500) | 0.609 | 0.516 | 0.875 (±0.0154) | 0.982 (±0.0020) | ✓\| | ✓\| |  |  |  |
| │ ├── CliXO:68 (NEDD4L, PAK2, ASAH1) | 0.500 (0.500, 0.500) | 0.500 (0.500, 0.500) | 0.609 | 0.516 | 0.875 (±0.0154) | 0.982 (±0.0020) | ✓\| | ✓\| |  |  |  |
| │ └── CliXO:77 (DOCK8, PTPRD, SPON1, APLN, IGF1, PAK2, ICA1, ASAH1, ZEB2) | 0.217 (0.184, 0.250) | 0.500 (0.500, 0.500) | 0.088 | 0.516 | 0.575 (±0.0068) | 0.361 (±0.0016) | ✓\| | ✓\| |  |  | CliXO:78 |
| │ └── CliXO:76 (DOCK8, PTPRD, SPON1, APLN, IGF1, PAK2, ASAH1, ZEB2) | 0.783 (0.750, 0.816) | 0.500 (0.500, 0.500) | 0.675 | 0.516 | 0.579 (±0.0061) | 0.630 (±0.0009) | ✓\| | ✓\| |  |  | CliXO:78 |
| │ └── CliXO:69 (PTPRD, SPON1, IGF1, ZEB2) | 0.783 (0.750, 0.816) | 0.500 (0.500, 0.500) | 0.675 | 0.516 | 0.681 (±0.0107) | 0.784 (±0.0015) | ✓\| | ✓\| |  |  | CliXO:78 |
| └── CliXO:84 (CDO1, PLIN2, CCSAP, BCAR3, SPAG4, BHLHE40, NECTIN4, SIGLEC6, SFXN3, CORO2A, TACC1, SLCO4A1, PIK3R1, KRT19, SYDE1, ERRFI1, GRHL1, CEBPA, GBE1, GPR146, CCDC127, CA4, LEP, EFNB1, P4HA1, SH3PXD2A, ZMYND8, SLCO2A1, GPT2, ERO1A, ANKRD37) | 0.208 (0.181, 0.234) | 0.500 (0.500, 0.500) | 0.088 | 0.516 | 0.664 (±0.0125) | 0.176 (±0.0090) | ✓\| | ✓\| |  |  | root |
| └── CliXO:83 (CDO1, PLIN2, CCSAP, BCAR3, SPAG4, BHLHE40, NECTIN4, SIGLEC6, SFXN3, CORO2A, TACC1, SLCO4A1, PIK3R1, KRT19, SYDE1, ERRFI1, GRHL1, CEBPA, GBE1, GPR146, CCDC127, LEP, EFNB1, P4HA1, SH3PXD2A, ZMYND8, SLCO2A1, GPT2, ERO1A, ANKRD37) | 0.208 (0.181, 0.234) | 0.500 (0.500, 0.500) | 0.088 | 0.516 | 0.659 (±0.0121) | 0.180 (±0.0085) | ✓\| | ✓\| |  |  | root |
| └── CliXO:82 (CDO1, PLIN2, CCSAP, BCAR3, SPAG4, BHLHE40, NECTIN4, SIGLEC6, SFXN3, CORO2A, KRT19, SYDE1, ERRFI1, GRHL1, GPR146, LEP, EFNB1, P4HA1, SH3PXD2A, ZMYND8, SLCO2A1, GPT2, ERO1A, ANKRD37) | 0.208 (0.181, 0.234) | 0.500 (0.500, 0.500) | 0.088 | 0.516 | 0.629 (±0.0081) | 0.284 (±0.0033) | ✓\| | ✓\| |  |  | root |
| ├── CliXO:72 (KRT19, LEP, SH3PXD2A, SLCO2A1) | 0.500 (0.500, 0.500) | 0.500 (0.500, 0.500) | 0.609 | 0.516 | 0.875 (±0.0154) | 0.982 (±0.0020) | ✓\| | ✓\| |  |  |  |
| └── CliXO:80 (PLIN2, CCSAP, SPAG4, BHLHE40, NECTIN4, SIGLEC6, SFXN3, CORO2A, KRT19, SYDE1, ERRFI1, GRHL1, GPR146, LEP, EFNB1, P4HA1, SH3PXD2A, GPT2, ERO1A, ANKRD37) | 0.208 (0.181, 0.234) | 0.500 (0.500, 0.500) | 0.088 | 0.516 | 0.626 (±0.0079) | 0.292 (±0.0031) | ✓\| | ✓\| |  |  | root |
| ├── CliXO:65 (BHLHE40, ANKRD37) | 0.792 (0.766, 0.819) | 0.500 (0.500, 0.500) | 0.694 | 0.516 | 0.698 (±0.0114) | 0.809 (±0.0016) | ✓\| | ✓\| |  |  | root |
| └── CliXO:79 (PLIN2, CCSAP, SPAG4, BHLHE40, NECTIN4, SFXN3, CORO2A, KRT19, SYDE1, GRHL1, GPR146, LEP, P4HA1, SH3PXD2A, GPT2, ERO1A) | 0.208 (0.181, 0.234) | 0.500 (0.500, 0.500) | 0.088 | 0.516 | 0.679 (±0.0102) | 0.214 (±0.0060) | ✓\| | ✓\| |  |  |  |
| │ └── CliXO:58 (P4HA1, ERO1A) | 0.500 (0.500, 0.500) | 0.500 (0.500, 0.500) | 0.609 | 0.516 | 0.875 (±0.0154) | 0.982 (±0.0020) | ✓\| | ✓\| |  |  |  |
| └── CliXO:67 (NECTIN4, SFXN3, ERO1A) | 0.500 (0.500, 0.500) | 0.500 (0.500, 0.500) | 0.609 | 0.516 | 0.875 (±0.0154) | 0.982 (±0.0020) | ✓\| | ✓\| |  |  |  |

The Reported column indicates the ontology from Tables 1–2 to which this row’s eligible genes belong. An ontology is reported if it passes all criteria (C1–C4) and its eligible gene set is not a strict subset of another passing ontology’s gene set. The column is empty when the ontology has no eligible genes or does not pass all criteria. ✓, the ontology passed the criterion; †, the gene had a grad-CAM value >97.5th percentile in the same ontology; |, separator between PPV/TPR and NPV/TNR results in C1 and C2 columns; AUC-PRC, area under the precision-recall curve; AUC-ROC, area under the receiver operating characteristic curve; C1, lower bounds finite and >0; C2, absolute difference ≤25% between development and test sets; C3, at least 1 eligible gene; C4, lower bound of AUC-ROC >0.5 in both sets; CI, confidence interval; Dev, development set; grad-CAM, gradient-weighted class activation mapping; HELLP, hemolysis, elevated liver enzyme, and low platelet; PE, preeclampsia; RMSE, root mean square error.

Table S9. Threshold-specific confusion matrices for the late-onset, severe PE pathogenesis model connected with third-trimester placenta under HELLP syndrome.

| Ontology | PPV/TPR (Dev) | PPV/TPR (Test) | NPV/TNR (Dev) | NPV/TNR (Test) | C1 | C2 | C3 | C4 | Reported |
| --- | --- | --- | --- | --- | --- | --- | --- | --- | --- |
| root (SFXN3, GBE1, ASAH1, KLF3, GPT2, LEP, SH3PXD2A, PKM, GPX3, FLNB, IGF1, CEBPD, ERRFI1, CCDC127, PDE5A, IGSF8, CDO1, CCSAP, SYDE1, ERO1A, SLC25A4, TOR1B, FCN3, BCAR3, NFIL3, LDLR, EFNB1, VEGFA, GRHL1, KRT19, ZEB2, GMFB, APLN, AZIN1, ZMYND8, FAM43A, NHLRC2, GPR146, NECTIN4, PIK3R1, SPON1, SPAG4, RAPGEF3, IQGAP2, BHLHE40, CEBPA, PREP, CORO2A, ANKRD37, ZNF555, P4HA1, PLIN2) | 34.9% (30.5, 39.2) / 86.1% (81.6, 90.7) | 3.4% (3.0, 3.9) / 100.0% (100.0, 100.0) | 93.5% (91.4, 95.5) / 52.9% (48.4, 57.3) | — / 0.5% (0.4, 0.6) | ✓\| | \| |  | ✓ |  |
| CliXO:78 (SFXN3, GBE1, ASAH1, KLF3, GPT2, LEP, SH3PXD2A, PKM, GPX3, FLNB, IGF1, CEBPD, ERRFI1, CCDC127, PDE5A, IGSF8, CDO1, CCSAP, SYDE1, ERO1A, SLC25A4, TOR1B, FCN3, BCAR3, NFIL3, LDLR, EFNB1, VEGFA, GRHL1, KRT19, ZEB2, GMFB, APLN, AZIN1, ZMYND8, FAM43A, NHLRC2, GPR146, NECTIN4, PIK3R1, SPON1, SPAG4, RAPGEF3, IQGAP2, BHLHE40, CEBPA, PREP, CORO2A, ANKRD37, ZNF555, P4HA1, PLIN2) | 59.7% (54.9, 64.6) / 86.1% (81.6, 90.7) | 3.4% (3.0, 3.9) / 100.0% (100.0, 100.0) | 95.6% (94.1, 97.1) / 83.5% (81.1, 85.8) | — / 0.5% (0.4, 0.6) | ✓\| | \| |  |  |  |
| ├── CliXO:53 (LDLR, SPON1) | 22.3% (19.8, 24.8) / 100.0% (100.0, 100.0) | 3.4% (2.9, 3.9) / 100.0% (100.0, 100.0) | — / 0.0% (0.0, 0.0) | — / 0.0% (0.0, 0.0) | ✓\| | ✓\| |  |  |  |
| ├── CliXO:70 (ASAH1, IGF1, PDE5A†, ZEB2, GMFB, APLN, AZIN1, NHLRC2, SPON1, IQGAP2, ZNF555) | 22.3% (19.8, 24.8) / 100.0% (100.0, 100.0) | 3.4% (2.9, 3.9) / 100.0% (100.0, 100.0) | — / 0.0% (0.0, 0.0) | — / 0.0% (0.0, 0.0) | ✓\| | ✓\| | ✓ | ✓ | CliXO:70 |
| │ ├── CliXO:65 (GMFB, AZIN1, NHLRC2, IQGAP2) | 22.3% (19.8, 24.8) / 100.0% (100.0, 100.0) | 3.4% (2.9, 3.9) / 100.0% (100.0, 100.0) | — / 0.0% (0.0, 0.0) | — / 0.0% (0.0, 0.0) | ✓\| | ✓\| |  |  |  |
| │ │ └── CliXO:55 (GMFB, AZIN1) | 22.3% (19.8, 24.8) / 100.0% (100.0, 100.0) | 3.4% (2.9, 3.9) / 100.0% (100.0, 100.0) | — / 0.0% (0.0, 0.0) | — / 0.0% (0.0, 0.0) | ✓\| | ✓\| |  |  |  |
| │ └── CliXO:68 (ASAH1, IGF1, PDE5A, ZEB2, APLN, AZIN1, NHLRC2, SPON1, IQGAP2, ZNF555) | 22.3% (19.8, 24.8) / 100.0% (100.0, 100.0) | 3.4% (2.9, 3.9) / 100.0% (100.0, 100.0) | — / 0.0% (0.0, 0.0) | — / 0.0% (0.0, 0.0) | ✓\| | ✓\| |  | ✓ | CliXO:70 |
| │ ├── CliXO:63 (PDE5A, AZIN1, NHLRC2, IQGAP2) | 40.2% (35.5, 45.0) / 100.0% (100.0, 100.0) | 3.4% (3.0, 3.9) / 100.0% (100.0, 100.0) | 100.0% (100.0, 100.0) / 56.1% (51.4, 60.8) | — / 0.9% (0.7, 1.1) | ✓\| | \| |  | ✓ | CliXO:70 |
| │ └── CliXO:67 (IGF1, PDE5A, ZEB2, SPON1, ZNF555) | 22.3% (19.8, 24.8) / 100.0% (100.0, 100.0) | 3.4% (2.9, 3.9) / 100.0% (100.0, 100.0) | — / 0.0% (0.0, 0.0) | — / 0.0% (0.0, 0.0) | ✓\| | ✓\| |  | ✓ |  |
| │ └── CliXO:62 (IGF1, ZEB2, SPON1) | 22.3% (19.8, 24.8) / 100.0% (100.0, 100.0) | 3.4% (2.9, 3.9) / 100.0% (100.0, 100.0) | — / 0.0% (0.0, 0.0) | — / 0.0% (0.0, 0.0) | ✓\| | ✓\| |  |  |  |
| │ └── CliXO:58 (IGF1, ZEB2) | 22.3% (19.8, 24.8) / 100.0% (100.0, 100.0) | 3.4% (2.9, 3.9) / 100.0% (100.0, 100.0) | — / 0.0% (0.0, 0.0) | — / 0.0% (0.0, 0.0) | ✓\| | ✓\| |  |  |  |
| └── CliXO:77 (SFXN3, GBE1, KLF3, GPT2, LEP, SH3PXD2A, PKM, GPX3, FLNB, CEBPD, ERRFI1, CCDC127, IGSF8, CDO1, CCSAP, SYDE1, ERO1A, SLC25A4, TOR1B, FCN3, BCAR3, NFIL3, LDLR, EFNB1, VEGFA, GRHL1, KRT19, ZMYND8, FAM43A, GPR146, NECTIN4, PIK3R1, SPAG4, RAPGEF3, BHLHE40, CEBPA, PREP, CORO2A, ANKRD37, P4HA1, PLIN2) | 54.5% (49.2, 59.8) / 86.1% (81.6, 90.7) | 3.4% (2.9, 3.9) / 100.0% (100.0, 100.0) | 95.4% (93.9, 97.0) / 79.7% (77.3, 82.1) | — / 0.0% (0.0, 0.0) | ✓\| | \| |  |  | CliXO:71 |
| ├── CliXO:54 (GPX3, FCN3) | 22.3% (19.8, 24.8) / 100.0% (100.0, 100.0) | 3.4% (2.9, 3.9) / 100.0% (100.0, 100.0) | — / 0.0% (0.0, 0.0) | — / 0.0% (0.0, 0.0) | ✓\| | ✓\| |  |  |  |
| ├── CliXO:71 (GPT2, FLNB, CEBPD, ERRFI1, IGSF8, SLC25A4, TOR1B, LDLR, VEGFA, FAM43A†, CEBPA, PREP) | 22.3% (19.8, 24.8) / 100.0% (100.0, 100.0) | 3.4% (2.9, 3.9) / 100.0% (100.0, 100.0) | — / 0.0% (0.0, 0.0) | — / 0.0% (0.0, 0.0) | ✓\| | ✓\| | ✓ |  | CliXO:71 |
| │ └── CliXO:69 (GPT2, FLNB, CEBPD, ERRFI1, IGSF8, SLC25A4, TOR1B, LDLR, FAM43A, PREP) | 22.3% (19.8, 24.8) / 100.0% (100.0, 100.0) | 3.4% (2.9, 3.9) / 100.0% (100.0, 100.0) | — / 0.0% (0.0, 0.0) | — / 0.0% (0.0, 0.0) | ✓\| | ✓\| |  |  |  |
| │ └── CliXO:56 (LDLR, PREP) | 22.3% (19.8, 24.8) / 100.0% (100.0, 100.0) | 3.4% (2.9, 3.9) / 100.0% (100.0, 100.0) | — / 0.0% (0.0, 0.0) | — / 0.0% (0.0, 0.0) | ✓\| | ✓\| |  |  |  |
| └── CliXO:76 (SFXN3, GBE1, KLF3, GPT2, LEP, SH3PXD2A, PKM, GPX3, FLNB, CEBPD, ERRFI1, CCDC127, IGSF8, CDO1, CCSAP, SYDE1, ERO1A, TOR1B, BCAR3, NFIL3, EFNB1, VEGFA, GRHL1, KRT19, ZMYND8, FAM43A, GPR146, NECTIN4, PIK3R1, SPAG4, RAPGEF3, BHLHE40, CEBPA, PREP, CORO2A, ANKRD37, P4HA1, PLIN2) | 22.3% (19.8, 24.8) / 100.0% (100.0, 100.0) | 3.4% (2.9, 3.9) / 100.0% (100.0, 100.0) | — / 0.0% (0.0, 0.0) | — / 0.0% (0.0, 0.0) | ✓\| | ✓\| |  |  | CliXO:71 |
| ├── CliXO:66 (GPX3, CEBPD, VEGFA, GPR146, P4HA1) | 22.3% (19.8, 24.8) / 100.0% (100.0, 100.0) | 3.4% (2.9, 3.9) / 100.0% (100.0, 100.0) | — / 0.0% (0.0, 0.0) | — / 0.0% (0.0, 0.0) | ✓\| | ✓\| |  |  |  |
| │ └── CliXO:57 (GPX3, VEGFA) | 22.3% (19.8, 24.8) / 100.0% (100.0, 100.0) | 3.4% (2.9, 3.9) / 100.0% (100.0, 100.0) | — / 0.0% (0.0, 0.0) | — / 0.0% (0.0, 0.0) | ✓\| | ✓\| |  |  |  |
| └── CliXO:75 (SFXN3, GBE1, KLF3, GPT2, LEP, SH3PXD2A, PKM, FLNB, ERRFI1, CCDC127, CDO1, CCSAP, SYDE1, ERO1A, BCAR3, NFIL3, EFNB1, GRHL1, KRT19, ZMYND8, FAM43A, GPR146, NECTIN4, PIK3R1, SPAG4, BHLHE40, CEBPA, PREP, CORO2A, ANKRD37, P4HA1, PLIN2) | 22.3% (19.8, 24.8) / 100.0% (100.0, 100.0) | 3.4% (2.9, 3.9) / 100.0% (100.0, 100.0) | — / 0.0% (0.0, 0.0) | — / 0.0% (0.0, 0.0) | ✓\| | ✓\| |  |  |  |
| ├── CliXO:60 (NFIL3, BHLHE40, ANKRD37) | 22.3% (19.8, 24.8) / 100.0% (100.0, 100.0) | 3.4% (2.9, 3.9) / 100.0% (100.0, 100.0) | — / 0.0% (0.0, 0.0) | — / 0.0% (0.0, 0.0) | ✓\| | ✓\| |  |  |  |
| │ └── CliXO:59 (BHLHE40, ANKRD37) | 22.3% (19.8, 24.8) / 100.0% (100.0, 100.0) | 3.4% (2.9, 3.9) / 100.0% (100.0, 100.0) | — / 0.0% (0.0, 0.0) | — / 0.0% (0.0, 0.0) | ✓\| | ✓\| |  |  |  |
| └── CliXO:74 (SFXN3, GBE1, KLF3, GPT2, LEP, SH3PXD2A, PKM, FLNB, ERRFI1, CDO1, CCSAP, SYDE1, ERO1A, BCAR3, EFNB1, GRHL1, KRT19, ZMYND8, GPR146, NECTIN4, PIK3R1, SPAG4, BHLHE40, CORO2A, ANKRD37, P4HA1, PLIN2) | 22.3% (19.8, 24.8) / 100.0% (100.0, 100.0) | 3.4% (2.9, 3.9) / 100.0% (100.0, 100.0) | — / 0.0% (0.0, 0.0) | — / 0.0% (0.0, 0.0) | ✓\| | ✓\| |  |  |  |
| └── CliXO:73 (SFXN3, KLF3, GPT2, LEP, SH3PXD2A, PKM, FLNB, ERRFI1, CCSAP, SYDE1, ERO1A, BCAR3, EFNB1, GRHL1, KRT19, GPR146, NECTIN4, SPAG4, BHLHE40, CORO2A, ANKRD37, P4HA1, PLIN2) | 22.3% (19.8, 24.8) / 100.0% (100.0, 100.0) | 3.4% (2.9, 3.9) / 100.0% (100.0, 100.0) | — / 0.0% (0.0, 0.0) | — / 0.0% (0.0, 0.0) | ✓\| | ✓\| |  |  |  |
| ├── CliXO:64 (SFXN3, KLF3, NECTIN4, BHLHE40) | 22.3% (19.8, 24.8) / 100.0% (100.0, 100.0) | 3.4% (2.9, 3.9) / 100.0% (100.0, 100.0) | — / 0.0% (0.0, 0.0) | — / 0.0% (0.0, 0.0) | ✓\| | ✓\| |  |  |  |
| └── CliXO:72 (SFXN3, GPT2, LEP, SH3PXD2A, FLNB, CCSAP, SYDE1, ERO1A, KRT19, GPR146, NECTIN4, SPAG4, BHLHE40, CORO2A, P4HA1, PLIN2) | 22.3% (19.8, 24.8) / 100.0% (100.0, 100.0) | 3.4% (2.9, 3.9) / 100.0% (100.0, 100.0) | — / 0.0% (0.0, 0.0) | — / 0.0% (0.0, 0.0) | ✓\| | ✓\| |  |  |  |
| ├── CliXO:52 (ERO1A, P4HA1) | 22.3% (19.8, 24.8) / 100.0% (100.0, 100.0) | 3.4% (2.9, 3.9) / 100.0% (100.0, 100.0) | — / 0.0% (0.0, 0.0) | — / 0.0% (0.0, 0.0) | ✓\| | ✓\| |  |  |  |
| └── CliXO:61 (SFXN3, ERO1A, NECTIN4) | 22.3% (19.8, 24.8) / 100.0% (100.0, 100.0) | 3.4% (2.9, 3.9) / 100.0% (100.0, 100.0) | — / 0.0% (0.0, 0.0) | — / 0.0% (0.0, 0.0) | ✓\| | ✓\| |  |  |  |

The Reported column indicates the ontology from Tables 1–2 to which this row’s eligible genes belong. An ontology is reported if it passes all criteria (C1–C4) and its eligible gene set is not a strict subset of another passing ontology’s gene set. The column is empty when the ontology has no eligible genes or does not pass all criteria. ✓, the ontology passed the criterion; †, the gene had a grad-CAM value >97.5th percentile in the same ontology; |, separator between PPV/TPR and NPV/TNR results in C1 and C2 columns; AUC-ROC, area under the receiver operating characteristic curve; C1, lower bounds finite and >0; C2, absolute difference ≤25% between development and test sets; C3, at least 1 eligible gene; C4, lower bound of AUC-ROC >0.5 in both sets; CI, confidence interval; Dev, development set; grad-CAM, gradient-weighted class activation mapping; HELLP, hemolysis, elevated liver enzyme, and low platelet; NPV, negative predictive value; PE, preeclampsia; PPV, positive predictive value; TNR, true negative rate; TPR, true positive rate.

Table S10. AUC-ROC, AUC-PRC, and RMSE for the late-onset, severe PE pathogenesis model connected with third-trimester placenta under HELLP syndrome.

| Ontology | AUC-ROC (Dev) | AUC-ROC (Test) | AUC-PRC (Dev) | AUC-PRC (Test) | RMSE (Dev) | RMSE (Test) | C1 | C2 | C3 | C4 | Reported |
| --- | --- | --- | --- | --- | --- | --- | --- | --- | --- | --- | --- |
| root (SFXN3, GBE1, ASAH1, KLF3, GPT2, LEP, SH3PXD2A, PKM, GPX3, FLNB, IGF1, CEBPD, ERRFI1, CCDC127, PDE5A, IGSF8, CDO1, CCSAP, SYDE1, ERO1A, SLC25A4, TOR1B, FCN3, BCAR3, NFIL3, LDLR, EFNB1, VEGFA, GRHL1, KRT19, ZEB2, GMFB, APLN, AZIN1, ZMYND8, FAM43A, NHLRC2, GPR146, NECTIN4, PIK3R1, SPON1, SPAG4, RAPGEF3, IQGAP2, BHLHE40, CEBPA, PREP, CORO2A, ANKRD37, ZNF555, P4HA1, PLIN2) | 0.695 (0.668, 0.722) | 0.502 (0.502, 0.503) | 0.611 | 0.516 | 0.419 (±0.0126) | 0.321 (±0.0030) | ✓\| | \| |  | ✓ |  |
| CliXO:78 (SFXN3, GBE1, ASAH1, KLF3, GPT2, LEP, SH3PXD2A, PKM, GPX3, FLNB, IGF1, CEBPD, ERRFI1, CCDC127, PDE5A, IGSF8, CDO1, CCSAP, SYDE1, ERO1A, SLC25A4, TOR1B, FCN3, BCAR3, NFIL3, LDLR, EFNB1, VEGFA, GRHL1, KRT19, ZEB2, GMFB, APLN, AZIN1, ZMYND8, FAM43A, NHLRC2, GPR146, NECTIN4, PIK3R1, SPON1, SPAG4, RAPGEF3, IQGAP2, BHLHE40, CEBPA, PREP, CORO2A, ANKRD37, ZNF555, P4HA1, PLIN2) | 0.776 (0.744, 0.808) | 0.452 (0.429, 0.475) | 0.103 | 0.431 | 0.441 (±0.0055) | 0.526 (±0.0006) | ✓\| | \| |  |  |  |
| ├── CliXO:53 (LDLR, SPON1) | 0.500 (0.500, 0.500) | 0.500 (0.500, 0.500) | 0.609 | 0.516 | 0.466 (±0.0275) | 0.181 (±0.0136) | ✓\| | ✓\| |  |  |  |
| ├── CliXO:70 (ASAH1, IGF1, PDE5A†, ZEB2, GMFB, APLN, AZIN1, NHLRC2, SPON1, IQGAP2, ZNF555) | 0.780 (0.757, 0.804) | 0.505 (0.504, 0.506) | 0.609 | 0.516 | 0.492 (±0.0137) | 0.674 (±0.0013) | ✓\| | ✓\| | ✓ | ✓ | CliXO:70 |
| │ ├── CliXO:65 (GMFB, AZIN1, NHLRC2, IQGAP2) | 0.500 (0.500, 0.500) | 0.500 (0.500, 0.500) | 0.609 | 0.516 | 0.466 (±0.0275) | 0.181 (±0.0136) | ✓\| | ✓\| |  |  |  |
| │ │ └── CliXO:55 (GMFB, AZIN1) | 0.500 (0.500, 0.500) | 0.500 (0.500, 0.500) | 0.609 | 0.516 | 0.466 (±0.0275) | 0.181 (±0.0136) | ✓\| | ✓\| |  |  |  |
| │ └── CliXO:68 (ASAH1, IGF1, PDE5A, ZEB2, APLN, AZIN1, NHLRC2, SPON1, IQGAP2, ZNF555) | 0.780 (0.757, 0.804) | 0.505 (0.504, 0.506) | 0.609 | 0.516 | 0.496 (±0.0140) | 0.681 (±0.0013) | ✓\| | ✓\| |  | ✓ | CliXO:70 |
| │ ├── CliXO:63 (PDE5A, AZIN1, NHLRC2, IQGAP2) | 0.780 (0.757, 0.804) | 0.505 (0.504, 0.506) | 0.609 | 0.516 | 0.493 (±0.0151) | 0.690 (±0.0013) | ✓\| | \| |  | ✓ | CliXO:70 |
| │ └── CliXO:67 (IGF1, PDE5A, ZEB2, SPON1, ZNF555) | 0.780 (0.757, 0.804) | 0.505 (0.504, 0.506) | 0.609 | 0.516 | 0.851 (±0.0140) | 0.954 (±0.0023) | ✓\| | ✓\| |  | ✓ |  |
| │ └── CliXO:62 (IGF1, ZEB2, SPON1) | 0.500 (0.500, 0.500) | 0.500 (0.500, 0.500) | 0.609 | 0.516 | 0.466 (±0.0275) | 0.181 (±0.0136) | ✓\| | ✓\| |  |  |  |
| │ └── CliXO:58 (IGF1, ZEB2) | 0.500 (0.500, 0.500) | 0.500 (0.500, 0.500) | 0.609 | 0.516 | 0.466 (±0.0275) | 0.181 (±0.0136) | ✓\| | ✓\| |  |  |  |
| └── CliXO:77 (SFXN3, GBE1, KLF3, GPT2, LEP, SH3PXD2A, PKM, GPX3, FLNB, CEBPD, ERRFI1, CCDC127, IGSF8, CDO1, CCSAP, SYDE1, ERO1A, SLC25A4, TOR1B, FCN3, BCAR3, NFIL3, LDLR, EFNB1, VEGFA, GRHL1, KRT19, ZMYND8, FAM43A, GPR146, NECTIN4, PIK3R1, SPAG4, RAPGEF3, BHLHE40, CEBPA, PREP, CORO2A, ANKRD37, P4HA1, PLIN2) | 0.721 (0.677, 0.765) | 0.450 (0.426, 0.473) | 0.103 | 0.431 | 0.412 (±0.0136) | 0.452 (±0.0010) | ✓\| | \| |  |  | CliXO:71 |
| ├── CliXO:54 (GPX3, FCN3) | 0.500 (0.500, 0.500) | 0.500 (0.500, 0.500) | 0.609 | 0.516 | 0.466 (±0.0275) | 0.181 (±0.0136) | ✓\| | ✓\| |  |  |  |
| ├── CliXO:71 (GPT2, FLNB, CEBPD, ERRFI1, IGSF8, SLC25A4, TOR1B, LDLR, VEGFA, FAM43A†, CEBPA, PREP) | 0.189 (0.161, 0.217) | 0.450 (0.426, 0.473) | 0.088 | 0.431 | 0.454 (±0.0262) | 0.182 (±0.0117) | ✓\| | ✓\| | ✓ |  | CliXO:71 |
| │ └── CliXO:69 (GPT2, FLNB, CEBPD, ERRFI1, IGSF8, SLC25A4, TOR1B, LDLR, FAM43A, PREP) | 0.189 (0.161, 0.217) | 0.450 (0.426, 0.473) | 0.190 | 0.445 | 0.786 (±0.0125) | 0.880 (±0.0021) | ✓\| | ✓\| |  |  |  |
| │ └── CliXO:56 (LDLR, PREP) | 0.500 (0.500, 0.500) | 0.500 (0.500, 0.500) | 0.609 | 0.516 | 0.466 (±0.0275) | 0.181 (±0.0136) | ✓\| | ✓\| |  |  |  |
| └── CliXO:76 (SFXN3, GBE1, KLF3, GPT2, LEP, SH3PXD2A, PKM, GPX3, FLNB, CEBPD, ERRFI1, CCDC127, IGSF8, CDO1, CCSAP, SYDE1, ERO1A, TOR1B, BCAR3, NFIL3, EFNB1, VEGFA, GRHL1, KRT19, ZMYND8, FAM43A, GPR146, NECTIN4, PIK3R1, SPAG4, RAPGEF3, BHLHE40, CEBPA, PREP, CORO2A, ANKRD37, P4HA1, PLIN2) | 0.514 (0.478, 0.551) | 0.450 (0.426, 0.473) | 0.103 | 0.431 | 0.420 (±0.0174) | 0.416 (±0.0016) | ✓\| | ✓\| |  |  | CliXO:71 |
| ├── CliXO:66 (GPX3, CEBPD, VEGFA, GPR146, P4HA1) | 0.500 (0.500, 0.500) | 0.500 (0.500, 0.500) | 0.609 | 0.516 | 0.466 (±0.0275) | 0.181 (±0.0136) | ✓\| | ✓\| |  |  |  |
| │ └── CliXO:57 (GPX3, VEGFA) | 0.500 (0.500, 0.500) | 0.500 (0.500, 0.500) | 0.609 | 0.516 | 0.466 (±0.0275) | 0.181 (±0.0136) | ✓\| | ✓\| |  |  |  |
| └── CliXO:75 (SFXN3, GBE1, KLF3, GPT2, LEP, SH3PXD2A, PKM, FLNB, ERRFI1, CCDC127, CDO1, CCSAP, SYDE1, ERO1A, BCAR3, NFIL3, EFNB1, GRHL1, KRT19, ZMYND8, FAM43A, GPR146, NECTIN4, PIK3R1, SPAG4, BHLHE40, CEBPA, PREP, CORO2A, ANKRD37, P4HA1, PLIN2) | 0.511 (0.474, 0.547) | 0.550 (0.527, 0.574) | 0.377 | 0.080 | 0.691 (±0.0103) | 0.731 (±0.0016) | ✓\| | ✓\| |  |  |  |
| ├── CliXO:60 (NFIL3, BHLHE40, ANKRD37) | 0.500 (0.500, 0.500) | 0.500 (0.500, 0.500) | 0.609 | 0.516 | 0.466 (±0.0275) | 0.181 (±0.0136) | ✓\| | ✓\| |  |  |  |
| │ └── CliXO:59 (BHLHE40, ANKRD37) | 0.500 (0.500, 0.500) | 0.500 (0.500, 0.500) | 0.609 | 0.516 | 0.466 (±0.0275) | 0.181 (±0.0136) | ✓\| | ✓\| |  |  |  |
| └── CliXO:74 (SFXN3, GBE1, KLF3, GPT2, LEP, SH3PXD2A, PKM, FLNB, ERRFI1, CDO1, CCSAP, SYDE1, ERO1A, BCAR3, EFNB1, GRHL1, KRT19, ZMYND8, GPR146, NECTIN4, PIK3R1, SPAG4, BHLHE40, CORO2A, ANKRD37, P4HA1, PLIN2) | 0.500 (0.500, 0.500) | 0.500 (0.500, 0.500) | 0.609 | 0.516 | 0.466 (±0.0275) | 0.181 (±0.0136) | ✓\| | ✓\| |  |  |  |
| └── CliXO:73 (SFXN3, KLF3, GPT2, LEP, SH3PXD2A, PKM, FLNB, ERRFI1, CCSAP, SYDE1, ERO1A, BCAR3, EFNB1, GRHL1, KRT19, GPR146, NECTIN4, SPAG4, BHLHE40, CORO2A, ANKRD37, P4HA1, PLIN2) | 0.500 (0.500, 0.500) | 0.500 (0.500, 0.500) | 0.609 | 0.516 | 0.466 (±0.0275) | 0.181 (±0.0136) | ✓\| | ✓\| |  |  |  |
| │ └── CliXO:59 (BHLHE40, ANKRD37) | 0.500 (0.500, 0.500) | 0.500 (0.500, 0.500) | 0.609 | 0.516 | 0.466 (±0.0275) | 0.181 (±0.0136) | ✓\| | ✓\| |  |  |  |
| ├── CliXO:64 (SFXN3, KLF3, NECTIN4, BHLHE40) | 0.500 (0.500, 0.500) | 0.500 (0.500, 0.500) | 0.609 | 0.516 | 0.466 (±0.0275) | 0.181 (±0.0136) | ✓\| | ✓\| |  |  |  |
| └── CliXO:72 (SFXN3, GPT2, LEP, SH3PXD2A, FLNB, CCSAP, SYDE1, ERO1A, KRT19, GPR146, NECTIN4, SPAG4, BHLHE40, CORO2A, P4HA1, PLIN2) | 0.500 (0.500, 0.500) | 0.500 (0.500, 0.500) | 0.609 | 0.516 | 0.466 (±0.0275) | 0.181 (±0.0136) | ✓\| | ✓\| |  |  |  |
| ├── CliXO:52 (ERO1A, P4HA1) | 0.500 (0.500, 0.500) | 0.500 (0.500, 0.500) | 0.609 | 0.516 | 0.466 (±0.0275) | 0.181 (±0.0136) | ✓\| | ✓\| |  |  |  |
| └── CliXO:61 (SFXN3, ERO1A, NECTIN4) | 0.500 (0.500, 0.500) | 0.500 (0.500, 0.500) | 0.609 | 0.516 | 0.466 (±0.0275) | 0.181 (±0.0136) | ✓\| | ✓\| |  |  |  |

The Reported column indicates the ontology from Tables 1–2 to which this row’s eligible genes belong. An ontology is reported if it passes all criteria (C1–C4) and its eligible gene set is not a strict subset of another passing ontology’s gene set. The column is empty when the ontology has no eligible genes or does not pass all criteria. ✓, the ontology passed the criterion; †, the gene had a grad-CAM value >97.5th percentile in the same ontology; |, separator between PPV/TPR and NPV/TNR results in C1 and C2 columns; AUC-PRC, area under the precision-recall curve; AUC-ROC, area under the receiver operating characteristic curve; C1, lower bounds finite and >0; C2, absolute difference ≤25% between development and test sets; C3, at least 1 eligible gene; C4, lower bound of AUC-ROC >0.5 in both sets; CI, confidence interval; Dev, development set; grad-CAM, gradient-weighted class activation mapping; HELLP, hemolysis, elevated liver enzyme, and low platelet; PE, preeclampsia; RMSE, root mean square error.

Table S11. Threshold-specific confusion matrices for the early-onset, severe PE pathogenesis model connected with late-secretory endometrium.

| Ontology | PPV/TPR (Dev) | PPV/TPR (Test) | NPV/TNR (Dev) | NPV/TNR (Test) | C1 | C2 | C3 | C4 | Reported |
| --- | --- | --- | --- | --- | --- | --- | --- | --- | --- |
| root (ABCA1, ANKRD13A, ZFAND5, PANX1, RASEF, LAPTM5, RNF11, ACKR2, TBXAS1, ETS2, ARRDC3, BHLHE40, MTMR6, ARHGEF12, STRN3, ELF4, RAPH1, ENG, EPS8L2, RGS16, GRHL1, MBD2, OXSR1, CTSA, NCEH1, VEGFA, AKAP13, TMEM154, LDLR, JUNB, YPEL2, CDYL2, TES, MAFF, CEBPD, GALE, SMIM3, SH3BP5, LYN, CSF2RB, TSC22D1, GULP1, CDKN1B, GADD45G, CORO2A, GPX3, NEK7, PRRG4, MINPP1, MXD1, SLC1A1, FAM43A, ZNF217, CHIC2, PPP2R5A, SLC2A1, PIK3CG, ZNF281, PIK3AP1, SLC38A6, TMEM176B, SNAPC1, SPTY2D1, HILPDA, IFIT1, STAB1, NR4A3, ZFYVE1, TIMP3, MAP3K8, CORO1A, SYNJ2, SLC38A1, RFK, PRKCH, RNF19A, ERP44, PPL, PGBD2, LATS2, HTRA1, LRRC8A, PAPPA, TREM1, KPNA1, TYROBP, MXRA5, NUCB2, LCP1, GBE1, PGK1, CPEB4, LURAP1L, EAF1, DUSP4, FBXO9, TSPAN12, RBM47, OPTN, ANKRD37, VDR, FAM210A, SLC25A4, MXI1, TMEM216, DUSP1, SLC7A2, TEX2, PIK3CB, PLIN2, LPL, SAMD8, ITGA5, TSPAN13, KIAA0513, IGSF3, ST6GAL1, SPAG4, SH2B3, ANXA1, PPA1, HEY1, TGFBR1, POLR2H, TANK, ILF2, STAT4, LYPD3, WDSUB1, KDM1A, PREP, CX3CR1, TPP1, RNASE1, GNG4, SGMS2, THRA, TMEM30A, DDIT4, SEMA4B, CASD1, PTPN12, FKBP4, DOCK8, KLF4, THOP1, UBR1, EPS8L1, BCAR3, SPI1, TP53BP2, SWAP70, FLI1, C9orf72, TSPAN14, SUMO3, PDZD2, CTBS, TMCC3, EDN1, ZNF143, FNDC3A, SH3PXD2A, TRAK2, ALDH1B1, DLX5, MYO5A, GRAMD1A, SLCO2B1, CIB2, RHOBTB3, DNAJB9, COL1A2, ARHGDIB, GLA, SARAF, NFIL3, HDAC1, MRI1, CYFIP2, MED13L, WDFY3, SMPDL3A, DCXR, EPS15, MEF2A, EFHD1, QPCT, HNMT, AMPD3, CRISPLD2, KIF17, CDK9, SLC9A9, C3AR1, FBXO8, LSM6, GNG7, GPER1, USP3, AMD1, PTBP3, COL17A1, GLE1, FOSL2, ELL2, LRRC32, ZNF512B, SLCO4A1, COQ2, COL6A1, PHKA2, CRYAB, CLIC5, HPGDS, SLC31A2, LIN7A, LY86, UBALD2, TNFAIP8, CTSF, COPZ2, TOP2B, CTSV, RNF24, SLC17A5, ARHGAP28, LRRC41, RCBTB1, PROM1, SERPINB9, SLC16A3, MAOB, SPON1, ASTE1, NPC1, DONSON, NARF, TNFAIP8L1, FAM172A, BRCA1, CHEK1, SBF2, ELMO1, TSPAN3, GRINA, KIAA0232, ASAP2, TMEM106B, ODF2, KLF3, XPO4, CDO1, LPXN, SULF2, MYO1F, DNMT3A, CALM1, MTPN, HLF, SH3BGRL, CLIC3, HSPA2, WFS1, NRIP1, SESN3, PIK3R1, TBC1D5, FGF13, RIN2, TYRO3, RBM26, TRIM25, HEXB, ARHGAP24, MAP4K4, CDK20, CYBB, OVGP1, TAF9B, OSMR, SMARCA2, DPYD, SACM1L, PLOD2, SNAP23, DNASE2, STK17A, CASP4, IGSF8, SUV39H1, CNPY4, TTLL5, ERO1A, OAS3, GOSR1, HMGB3, TRIM24, EDEM1, PPM1M, RAB8B, ST6GALNAC1, CCND1, MAD2L2, SLC39A8, FZD1, BAD, MOB3C, CLCN5, C4orf3, RDX, CSF3R, PCYOX1, PUS7, DDX17, FBN2, PPT1, PJA1, PEBP1, IL33, TKFC, CBX5, SRPK2, FBLN7, PALLD, CDC42BPA, CNN3, FAM107B, DDR2, DEGS1, GEMIN5, PODXL, FBLIM1, AMOTL2, LHFPL2, MAML2, PDGFC, DKK3, MID1, NIPAL3, PCCB, ATXN10, HJURP, SHANK2, TOR1B, PKM, BCAT2, CCDC113, UNC119, MME, MELK, RAD51, HMMR, MICU3, COX8A, INPP5F, PLBD1, MX1, CPD, ZNF462, GREM2, RNF20, TPD52L1, MTA1, PTPRJ, CKS2, TRIP13, PIP5K1B, LMAN2L, PLCB1, IGF1, FRMD4A, AZIN1, ZFP3, FOLR1, UBE2K, INTS7, CITED2, HNRNPA0, MATN2, GPX7, HMGN3, ZMYND11, PAM, HMGCR, MBNL2, EIF4A2, RPL15, CCDC115, NRCAM, PIGX, GSTK1, PIP5KL1, TRA2B, EPHB3, PFKM, ZNF84, PGRMC1, SLC23A2, STXBP6, CLOCK, TRIB2, DROSHA, CXXC1, NFIA) | 43.1% (40.7, 45.4) / 100.0% (100.0, 100.0) | 0.8% (0.7, 1.0) / — | — / 0.0% (0.0, 0.0) | — / 0.0% (0.0, 0.0) | \| | \| |  |  |  |
| CliXO:454 (ABCA1, ANKRD13A, ZFAND5, PANX1, RASEF, LAPTM5, RNF11, ACKR2, TBXAS1, ETS2, ARRDC3, BHLHE40, MTMR6, ARHGEF12, STRN3, ELF4, RAPH1, ENG, EPS8L2, RGS16, GRHL1, MBD2, OXSR1, CTSA, NCEH1, VEGFA, AKAP13, TMEM154, LDLR, JUNB, YPEL2, CDYL2, TES, MAFF, CEBPD, GALE, SMIM3, SH3BP5, LYN, CSF2RB, TSC22D1, GULP1, CDKN1B, GADD45G, CORO2A, GPX3, NEK7, PRRG4, MINPP1, MXD1, SLC1A1, FAM43A, ZNF217, CHIC2, PPP2R5A, SLC2A1, PIK3CG, ZNF281, PIK3AP1, SLC38A6, TMEM176B, SNAPC1, SPTY2D1, HILPDA, IFIT1, STAB1, NR4A3, ZFYVE1, TIMP3, MAP3K8, CORO1A, SYNJ2, SLC38A1, RFK, PRKCH, RNF19A, ERP44, PPL, PGBD2, LATS2, HTRA1, LRRC8A, PAPPA, TREM1, KPNA1, TYROBP, MXRA5, NUCB2, LCP1, GBE1, PGK1, CPEB4, LURAP1L, EAF1, DUSP4, FBXO9, TSPAN12, RBM47, OPTN, ANKRD37, VDR, FAM210A, SLC25A4, MXI1, TMEM216, DUSP1, SLC7A2, TEX2, PIK3CB, PLIN2, LPL, SAMD8, ITGA5, TSPAN13, KIAA0513, IGSF3, ST6GAL1, SPAG4, SH2B3, ANXA1, PPA1, HEY1, TGFBR1, POLR2H, TANK, ILF2, STAT4, LYPD3, WDSUB1, KDM1A, PREP, CX3CR1, TPP1, RNASE1, GNG4, SGMS2, THRA, TMEM30A, DDIT4, SEMA4B, CASD1, PTPN12, FKBP4, DOCK8, KLF4, THOP1, UBR1, EPS8L1, BCAR3, SPI1, TP53BP2, SWAP70, FLI1, C9orf72, TSPAN14, SUMO3, PDZD2, CTBS, TMCC3, EDN1, ZNF143, FNDC3A, SH3PXD2A, TRAK2, ALDH1B1, DLX5, MYO5A, GRAMD1A, SLCO2B1, CIB2, RHOBTB3, DNAJB9, COL1A2, ARHGDIB, GLA, SARAF, NFIL3, HDAC1, MRI1, CYFIP2, MED13L, WDFY3, SMPDL3A, DCXR, EPS15, MEF2A, EFHD1, QPCT, HNMT, AMPD3, CRISPLD2, KIF17, CDK9, SLC9A9, C3AR1, FBXO8, LSM6, GNG7, GPER1, USP3, AMD1, PTBP3, COL17A1, GLE1, FOSL2, ELL2, LRRC32, ZNF512B, SLCO4A1, COQ2, COL6A1, PHKA2, CRYAB, CLIC5, HPGDS, SLC31A2, LIN7A, LY86, UBALD2, TNFAIP8, CTSF, COPZ2, TOP2B, CTSV, RNF24, SLC17A5, ARHGAP28, LRRC41, RCBTB1, PROM1, SERPINB9, SLC16A3, MAOB, SPON1, ASTE1, NPC1, DONSON, NARF, TNFAIP8L1, FAM172A, BRCA1, CHEK1, SBF2, ELMO1, TSPAN3, GRINA, KIAA0232, ASAP2, TMEM106B, ODF2, KLF3, XPO4, CDO1, LPXN, SULF2, MYO1F, DNMT3A, CALM1, MTPN, HLF, SH3BGRL, CLIC3, HSPA2, WFS1, NRIP1, SESN3, PIK3R1, TBC1D5, FGF13, RIN2, TYRO3, RBM26, TRIM25, HEXB, ARHGAP24, MAP4K4, CDK20, CYBB, OVGP1, TAF9B, OSMR, SMARCA2, DPYD, SACM1L, PLOD2, SNAP23, DNASE2, STK17A, CASP4, IGSF8, SUV39H1, CNPY4, TTLL5, ERO1A, OAS3, GOSR1, HMGB3, TRIM24, EDEM1, PPM1M, RAB8B, ST6GALNAC1, CCND1, MAD2L2, SLC39A8, FZD1, BAD, MOB3C, CLCN5, C4orf3, RDX, CSF3R, PCYOX1, PUS7, DDX17, FBN2, PPT1, PJA1, PEBP1, IL33, TKFC, CBX5, SRPK2, FBLN7, PALLD, CDC42BPA, CNN3, FAM107B, DDR2, DEGS1, GEMIN5, PODXL, FBLIM1, AMOTL2, LHFPL2, MAML2, PDGFC, DKK3, MID1, NIPAL3, PCCB, ATXN10, HJURP, SHANK2, TOR1B, PKM, BCAT2, CCDC113, UNC119, MME, MELK, RAD51, HMMR, MICU3, COX8A, INPP5F, PLBD1, MX1, CPD, ZNF462, GREM2, RNF20, TPD52L1, MTA1, PTPRJ, CKS2, TRIP13, PIP5K1B, LMAN2L, PLCB1, IGF1, FRMD4A, AZIN1, ZFP3, FOLR1, UBE2K, INTS7, CITED2, HNRNPA0, MATN2, GPX7, HMGN3, ZMYND11, PAM, HMGCR, MBNL2, EIF4A2, RPL15, CCDC115, NRCAM, PIGX, GSTK1, PIP5KL1, TRA2B, EPHB3, PFKM, ZNF84, PGRMC1, SLC23A2, STXBP6, CLOCK, TRIB2, DROSHA, CXXC1, NFIA) | 43.1% (40.7, 45.4) / 100.0% (100.0, 100.0) | 0.8% (0.7, 1.0) / — | — / 0.0% (0.0, 0.0) | — / 0.0% (0.0, 0.0) | \| | \| |  |  |  |
| ├── CliXO:416 (KPNA1, NUCB2, ST6GALNAC1) | 72.0% (68.4, 75.7) / 76.7% (73.8, 79.6) | 0.8% (0.7, 1.0) / — | 81.7% (79.8, 83.5) / 77.3% (74.3, 80.4) | — / 0.0% (0.0, 0.0) | \| | \| |  |  |  |
| ├── CliXO:436 (CSF2RB, FAM43A, SLC38A6, PGK1, VDR, ANXA1, HEY1, LYPD3, CASD1, QPCT, COQ2, ARHGAP28, SPON1, BRCA1, CHEK1, TMEM106B, CDO1, HLF, SH3BGRL, CLIC3, WFS1, SESN3, PIK3R1, FGF13, RBM26, HEXB, SMARCA2, IGSF8, CNPY4, CCND1, MAD2L2, FZD1, C4orf3, PCYOX1, PJA1, IL33, CNN3, DDR2, FBLIM1, AMOTL2, LHFPL2, PDGFC, PKM, MME, MICU3, INPP5F) | 72.0% (68.4, 75.7) / 76.7% (73.8, 79.6) | 0.8% (0.7, 1.0) / — | 81.7% (79.8, 83.5) / 77.3% (74.3, 80.4) | — / 0.0% (0.0, 0.0) | \| | \| |  |  |  |
| │ ├── CliXO:410 (HEY1, LYPD3) | 72.0% (68.4, 75.7) / 76.7% (73.8, 79.6) | 0.8% (0.7, 1.0) / — | 81.7% (79.8, 83.5) / 77.3% (74.3, 80.4) | — / 0.0% (0.0, 0.0) | \| | \| |  |  |  |
| │ ├── CliXO:411 (ARHGAP28, SESN3) | 72.0% (68.4, 75.7) / 76.7% (73.8, 79.6) | 0.8% (0.7, 1.0) / — | 81.7% (79.8, 83.5) / 77.3% (74.3, 80.4) | — / 0.0% (0.0, 0.0) | \| | \| |  |  |  |
| │ ├── CliXO:415 (COQ2, SPON1, CHEK1) | 72.0% (68.4, 75.7) / 76.7% (73.8, 79.6) | 0.8% (0.7, 1.0) / — | 81.7% (79.8, 83.5) / 77.3% (74.3, 80.4) | — / 0.0% (0.0, 0.0) | \| | \| |  |  |  |
| │ ├── CliXO:420 (CSF2RB, FAM43A, SLC38A6, CASD1, QPCT, SH3BGRL, FGF13, IL33, CNN3, MICU3) | 72.0% (68.4, 75.7) / 76.7% (73.8, 79.6) | 0.8% (0.7, 1.0) / — | 81.7% (79.8, 83.5) / 77.3% (74.3, 80.4) | — / 0.0% (0.0, 0.0) | \| | \| |  |  |  |
| │ │ ├── CliXO:406 (SH3BGRL, FGF13) | 72.0% (68.4, 75.7) / 76.7% (73.8, 79.6) | 0.8% (0.7, 1.0) / — | 81.7% (79.8, 83.5) / 77.3% (74.3, 80.4) | — / 0.0% (0.0, 0.0) | \| | \| |  |  |  |
| │ │ └── CliXO:407 (FAM43A, QPCT) | 72.0% (68.4, 75.7) / 76.7% (73.8, 79.6) | 0.8% (0.7, 1.0) / — | 81.7% (79.8, 83.5) / 77.3% (74.3, 80.4) | — / 0.0% (0.0, 0.0) | \| | \| |  |  |  |
| │ └── CliXO:430 (FAM43A, SLC38A6, HEY1, CASD1, QPCT, SPON1, CHEK1, TMEM106B, SH3BGRL, CLIC3, SESN3, PIK3R1, FGF13, RBM26, SMARCA2, IGSF8, CCND1, MAD2L2, C4orf3, PJA1, IL33, CNN3, DDR2, FBLIM1, LHFPL2, PDGFC, PKM, MME, MICU3) | 72.0% (68.4, 75.7) / 76.7% (73.8, 79.6) | 0.8% (0.7, 1.0) / — | 81.7% (79.8, 83.5) / 77.3% (74.3, 80.4) | — / 0.0% (0.0, 0.0) | \| | \| |  |  |  |
| │ └── CliXO:413 (LHFPL2, PKM) | 72.0% (68.4, 75.7) / 76.7% (73.8, 79.6) | 0.8% (0.7, 1.0) / — | 81.7% (79.8, 83.5) / 77.3% (74.3, 80.4) | — / 0.0% (0.0, 0.0) | \| | \| |  |  |  |
| ├── CliXO:442 (PANX1, RNF11, BHLHE40, MTMR6, STRN3, NCEH1, VEGFA, JUNB, SMIM3, CSF2RB, TSC22D1, GULP1, CDKN1B, MINPP1, MXD1, FAM43A, ZNF281, SLC38A6, TMEM176B, SNAPC1, SPTY2D1, HILPDA, NR4A3, MAP3K8, SYNJ2, PPL, LATS2, TREM1, PGK1, DUSP4, ANKRD37, VDR, DUSP1, ITGA5, TSPAN13, KIAA0513, SPAG4, SH2B3, ANXA1, HEY1, LYPD3, WDSUB1, SGMS2, TMEM30A, DDIT4, SEMA4B, CASD1, EPS8L1, BCAR3, TP53BP2, TMCC3, MYO5A, RHOBTB3, WDFY3, MEF2A, QPCT, HNMT, SLCO4A1, HPGDS, RNF24, ARHGAP28, RCBTB1, MAOB, SPON1, CHEK1, ELMO1, TMEM106B, LPXN, SH3BGRL, CLIC3, SESN3, PIK3R1, FGF13, RBM26, SACM1L, MAD2L2, IL33, CNN3, FBLIM1, LHFPL2, MAML2, PDGFC, PKM, MME, MICU3) | 72.0% (68.4, 75.7) / 76.7% (73.8, 79.6) | 0.8% (0.7, 1.0) / — | 81.7% (79.8, 83.5) / 77.3% (74.3, 80.4) | — / 0.0% (0.0, 0.0) | \| | \| |  |  |  |
| │ └── CliXO:441 (RNF11, BHLHE40, MTMR6, STRN3, NCEH1, VEGFA, JUNB, SMIM3, CSF2RB, TSC22D1, GULP1, CDKN1B, MINPP1, MXD1, FAM43A, ZNF281, SLC38A6, TMEM176B, SNAPC1, HILPDA, NR4A3, SYNJ2, PPL, LATS2, TREM1, PGK1, DUSP4, ANKRD37, VDR, DUSP1, ITGA5, SPAG4, SH2B3, ANXA1, HEY1, LYPD3, WDSUB1, SGMS2, SEMA4B, CASD1, BCAR3, TP53BP2, TMCC3, MYO5A, WDFY3, MEF2A, QPCT, HNMT, SLCO4A1, HPGDS, ARHGAP28, RCBTB1, MAOB, SPON1, CHEK1, ELMO1, TMEM106B, LPXN, SH3BGRL, CLIC3, SESN3, PIK3R1, FGF13, RBM26, SACM1L, MAD2L2, IL33, CNN3, FBLIM1, LHFPL2, MAML2, PDGFC, PKM, MME, MICU3) | 72.0% (68.4, 75.7) / 76.7% (73.8, 79.6) | 0.8% (0.7, 1.0) / — | 81.7% (79.8, 83.5) / 77.3% (74.3, 80.4) | — / 0.0% (0.0, 0.0) | \| | \| |  |  |  |
| │ ├── CliXO:417 (WDFY3, HNMT, ARHGAP28, RCBTB1, ELMO1, LPXN) | 72.0% (68.4, 75.7) / 76.7% (73.8, 79.6) | 0.8% (0.7, 1.0) / — | 81.7% (79.8, 83.5) / 77.3% (74.3, 80.4) | — / 0.0% (0.0, 0.0) | \| | \| |  |  |  |
| │ │ └── CliXO:405 (ARHGAP28, RCBTB1) | 72.0% (68.4, 75.7) / 76.7% (73.8, 79.6) | 0.8% (0.7, 1.0) / — | 81.7% (79.8, 83.5) / 77.3% (74.3, 80.4) | — / 0.0% (0.0, 0.0) | \| | \| |  |  |  |
| │ ├── CliXO:418 (PGK1, SPAG4, HEY1, LYPD3, BCAR3, SLCO4A1) | 72.0% (68.4, 75.7) / 76.7% (73.8, 79.6) | 0.8% (0.7, 1.0) / — | 81.7% (79.8, 83.5) / 77.3% (74.3, 80.4) | — / 0.0% (0.0, 0.0) | \| | \| |  |  |  |
| │ └── CliXO:437 (RNF11, MTMR6, STRN3, JUNB, SMIM3, CSF2RB, TSC22D1, GULP1, CDKN1B, MINPP1, MXD1, FAM43A, ZNF281, SLC38A6, HILPDA, NR4A3, SYNJ2, TREM1, PGK1, DUSP4, ANKRD37, VDR, ITGA5, SPAG4, ANXA1, HEY1, LYPD3, WDSUB1, CASD1, BCAR3, QPCT, HNMT, ARHGAP28, RCBTB1, SPON1, CHEK1, TMEM106B, SH3BGRL, CLIC3, SESN3, PIK3R1, FGF13, RBM26, MAD2L2, IL33, CNN3, FBLIM1, LHFPL2, PDGFC, PKM, MME, MICU3) | 72.0% (68.4, 75.7) / 76.7% (73.8, 79.6) | 0.8% (0.7, 1.0) / — | 81.7% (79.8, 83.5) / 77.3% (74.3, 80.4) | — / 0.0% (0.0, 0.0) | \| | \| |  |  |  |
| │ └── CliXO:432 (RNF11, MTMR6, STRN3, SMIM3, CSF2RB, TSC22D1, GULP1, MXD1, FAM43A, SLC38A6, HILPDA, SYNJ2, TREM1, PGK1, DUSP4, VDR, ITGA5, ANXA1, HEY1, LYPD3, WDSUB1, CASD1, QPCT, ARHGAP28, SH3BGRL, SESN3, PIK3R1, FGF13, IL33, CNN3, FBLIM1, PKM, MME, MICU3) | 72.0% (68.4, 75.7) / 76.7% (73.8, 79.6) | 0.8% (0.7, 1.0) / — | 81.7% (79.8, 83.5) / 77.3% (74.3, 80.4) | — / 0.0% (0.0, 0.0) | \| | \| |  |  |  |
| │ ├── CliXO:409 (WDSUB1, MICU3) | 72.0% (68.4, 75.7) / 76.7% (73.8, 79.6) | 0.8% (0.7, 1.0) / — | 81.7% (79.8, 83.5) / 77.3% (74.3, 80.4) | — / 0.0% (0.0, 0.0) | \| | \| |  |  |  |
| │ ├── CliXO:414 (ITGA5, ANXA1) | 72.0% (68.4, 75.7) / 76.7% (73.8, 79.6) | 0.8% (0.7, 1.0) / — | 81.7% (79.8, 83.5) / 77.3% (74.3, 80.4) | — / 0.0% (0.0, 0.0) | \| | \| |  |  |  |
| │ └── CliXO:425 (STRN3, SMIM3, CSF2RB, FAM43A, SLC38A6, HILPDA, CASD1, QPCT, ARHGAP28, SH3BGRL, SESN3, FGF13, IL33, CNN3, MME, MICU3) | 72.0% (68.4, 75.7) / 76.7% (73.8, 79.6) | 0.8% (0.7, 1.0) / — | 81.7% (79.8, 83.5) / 77.3% (74.3, 80.4) | — / 0.0% (0.0, 0.0) | \| | \| |  |  |  |
| ├── CliXO:448 (IFIT1, MXRA5, FBXO9, SLC25A4, TMEM216, PPA1, POLR2H, ILF2, KDM1A, THRA, CASD1, FKBP4, THOP1, SUMO3, SH3PXD2A, ALDH1B1, DLX5, CIB2, COL1A2, GLA, HDAC1, MRI1, DCXR, QPCT, KIF17, LSM6, GNG7, GPER1, AMD1, GLE1, ZNF512B, COQ2, COL6A1, PHKA2, CLIC5, LIN7A, CTSF, COPZ2, TOP2B, CTSV, LRRC41, SPON1, ASTE1, DONSON, TNFAIP8L1, FAM172A, BRCA1, CHEK1, TSPAN3, GRINA, KIAA0232, TMEM106B, ODF2, XPO4, CDO1, SULF2, DNMT3A, CALM1, MTPN, HLF, SH3BGRL, CLIC3, HSPA2, WFS1, NRIP1, SESN3, PIK3R1, TBC1D5, FGF13, TYRO3, RBM26, TRIM25, HEXB, CDK20, OVGP1, TAF9B, SMARCA2, DNASE2, IGSF8, SUV39H1, CNPY4, TTLL5, OAS3, GOSR1, HMGB3, TRIM24, PPM1M, ST6GALNAC1, CCND1, MAD2L2, SLC39A8, FZD1, BAD, MOB3C, CLCN5, C4orf3, RDX, PCYOX1, PUS7, DDX17, FBN2, PPT1, PJA1, PEBP1, IL33, TKFC, CBX5, SRPK2, FBLN7, PALLD, CDC42BPA, CNN3, DDR2, GEMIN5, PODXL, FBLIM1, AMOTL2, LHFPL2, PDGFC, DKK3, MID1, NIPAL3, PCCB, ATXN10, HJURP, SHANK2, PKM, BCAT2, CCDC113, UNC119, MME, MELK, RAD51, HMMR, COX8A, INPP5F, PLBD1, MX1, ZNF462, GREM2, RNF20, TPD52L1, MTA1, CKS2, TRIP13, PIP5K1B, LMAN2L, PLCB1, IGF1, AZIN1, ZFP3, FOLR1, UBE2K, INTS7, HNRNPA0, MATN2, GPX7, HMGN3, ZMYND11, PAM, HMGCR, MBNL2, EIF4A2, RPL15, CCDC115, NRCAM, PIGX, GSTK1, PIP5KL1, TRA2B, EPHB3, PFKM, ZNF84, PGRMC1, SLC23A2, STXBP6, CLOCK, TRIB2, DROSHA, CXXC1, NFIA) | 43.1% (40.7, 45.4) / 100.0% (100.0, 100.0) | 0.8% (0.7, 1.0) / — | — / 0.0% (0.0, 0.0) | — / 0.0% (0.0, 0.0) | \| | \| |  |  |  |
| │ ├── CliXO:435 (POLR2H, ILF2, CASD1, SUMO3, SH3PXD2A, ALDH1B1, QPCT, GPER1, AMD1, TOP2B, CTSV, FAM172A, TSPAN3, NRIP1, FGF13, HEXB, CNPY4, CCND1, MAD2L2, FZD1, C4orf3, PUS7, FBN2, PJA1, IL33, PALLD, CNN3, DDR2, FBLIM1, AMOTL2, LHFPL2, PDGFC, DKK3, MID1, PKM, MME, INPP5F, PLBD1, PIP5K1B, INTS7, SLC23A2) | 72.0% (68.4, 75.7) / 76.7% (73.8, 79.6) | 0.8% (0.7, 1.0) / — | 81.7% (79.8, 83.5) / 77.3% (74.3, 80.4) | — / 0.0% (0.0, 0.0) | \| | \| |  |  |  |
| │ │ └── CliXO:427 (QPCT, AMD1, TOP2B, FAM172A, NRIP1, HEXB, CNPY4, CCND1, MAD2L2, FZD1, C4orf3, PJA1, IL33, PALLD, CNN3, DDR2, FBLIM1, AMOTL2, LHFPL2, PDGFC, MME, INPP5F) | 72.0% (68.4, 75.7) / 76.7% (73.8, 79.6) | 0.8% (0.7, 1.0) / — | 81.7% (79.8, 83.5) / 77.3% (74.3, 80.4) | — / 0.0% (0.0, 0.0) | \| | \| |  |  |  |
| │ │ └── CliXO:424 (QPCT, NRIP1, HEXB, CCND1, MAD2L2, FZD1, C4orf3, PJA1, IL33, CNN3, DDR2, FBLIM1, AMOTL2, PDGFC, MME) | 72.0% (68.4, 75.7) / 76.7% (73.8, 79.6) | 0.8% (0.7, 1.0) / — | 81.7% (79.8, 83.5) / 77.3% (74.3, 80.4) | — / 0.0% (0.0, 0.0) | \| | \| |  |  |  |
| │ ├── CliXO:443 (IFIT1, CASD1, DCXR, ZNF512B, COQ2, CTSF, SPON1, BRCA1, CHEK1, TSPAN3, TMEM106B, CDO1, SULF2, HLF, SH3BGRL, CLIC3, HSPA2, WFS1, SESN3, PIK3R1, TBC1D5, FGF13, TYRO3, RBM26, TAF9B, SMARCA2, HMGB3, TRIM24, CLCN5, RDX, PCYOX1, PUS7, DDX17, PPT1, PEBP1, TKFC, SRPK2, FBLN7, PALLD, GEMIN5, AMOTL2, MID1, ATXN10, HJURP, SHANK2, CCDC113, MELK, RAD51, HMMR, INPP5F, PLBD1, MX1, ZNF462, GREM2, RNF20, TPD52L1, MTA1, CKS2, TRIP13, PIP5K1B, PLCB1, IGF1, AZIN1, ZFP3, UBE2K, INTS7, HNRNPA0, MATN2, GPX7, HMGN3, PAM, MBNL2, EIF4A2, RPL15, CCDC115, NRCAM, TRA2B, ZNF84, PGRMC1, SLC23A2, STXBP6, CLOCK, TRIB2, DROSHA, NFIA) | 43.1% (40.7, 45.4) / 100.0% (100.0, 100.0) | 0.8% (0.7, 1.0) / — | — / 0.0% (0.0, 0.0) | — / 0.0% (0.0, 0.0) | \| | \| |  |  |  |
| │ │ └── CliXO:438 (CASD1, DCXR, COQ2, SPON1, BRCA1, CHEK1, TMEM106B, CDO1, SULF2, HLF, SH3BGRL, CLIC3, HSPA2, WFS1, SESN3, PIK3R1, FGF13, TYRO3, RBM26, TAF9B, SMARCA2, TRIM24, CLCN5, PCYOX1, DDX17, GEMIN5, HJURP, CCDC113, MELK, HMMR, PLBD1, MX1, ZNF462, RNF20, MTA1, CKS2, PIP5K1B, PLCB1, AZIN1, ZFP3, UBE2K, INTS7, HNRNPA0, MATN2, GPX7, HMGN3, PAM, MBNL2, EIF4A2, ZNF84, SLC23A2, STXBP6, TRIB2, DROSHA) | 72.0% (68.4, 75.7) / 76.7% (73.8, 79.6) | 0.8% (0.7, 1.0) / — | 81.7% (79.8, 83.5) / 77.3% (74.3, 80.4) | — / 0.0% (0.0, 0.0) | \| | \| |  |  |  |
| │ │ └── CliXO:423 (CASD1, TMEM106B, SH3BGRL, WFS1, SESN3, PIK3R1, FGF13, TYRO3, RBM26, SMARCA2, TRIM24, PCYOX1, PIP5K1B, ZFP3, PAM) | 72.0% (68.4, 75.7) / 76.7% (73.8, 79.6) | 0.8% (0.7, 1.0) / — | 81.7% (79.8, 83.5) / 77.3% (74.3, 80.4) | — / 0.0% (0.0, 0.0) | \| | \| |  |  |  |
| │ │ └── CliXO:422 (CASD1, TMEM106B, SH3BGRL, WFS1, SESN3, PIK3R1, TYRO3, RBM26, SMARCA2, TRIM24) | 72.0% (68.4, 75.7) / 76.7% (73.8, 79.6) | 0.8% (0.7, 1.0) / — | 81.7% (79.8, 83.5) / 77.3% (74.3, 80.4) | — / 0.0% (0.0, 0.0) | \| | \| |  |  |  |
| │ └── CliXO:447 (IFIT1, MXRA5, FBXO9, SLC25A4, TMEM216, PPA1, POLR2H, ILF2, KDM1A, THRA, CASD1, FKBP4, THOP1, SUMO3, SH3PXD2A, ALDH1B1, DLX5, CIB2, COL1A2, GLA, HDAC1, MRI1, DCXR, KIF17, LSM6, GNG7, GPER1, AMD1, GLE1, ZNF512B, COQ2, COL6A1, PHKA2, CLIC5, LIN7A, CTSF, COPZ2, TOP2B, CTSV, LRRC41, SPON1, ASTE1, DONSON, TNFAIP8L1, FAM172A, BRCA1, CHEK1, TSPAN3, GRINA, KIAA0232, TMEM106B, ODF2, XPO4, CDO1, SULF2, DNMT3A, CALM1, MTPN, HLF, SH3BGRL, CLIC3, HSPA2, WFS1, NRIP1, PIK3R1, TBC1D5, FGF13, TYRO3, RBM26, TRIM25, HEXB, CDK20, OVGP1, TAF9B, SMARCA2, DNASE2, IGSF8, SUV39H1, CNPY4, TTLL5, OAS3, GOSR1, HMGB3, TRIM24, PPM1M, ST6GALNAC1, CCND1, MAD2L2, SLC39A8, FZD1, BAD, MOB3C, CLCN5, C4orf3, RDX, PCYOX1, PUS7, DDX17, FBN2, PPT1, PJA1, PEBP1, TKFC, CBX5, SRPK2, FBLN7, PALLD, CDC42BPA, DDR2, GEMIN5, PODXL, FBLIM1, AMOTL2, LHFPL2, PDGFC, DKK3, MID1, NIPAL3, PCCB, ATXN10, HJURP, SHANK2, PKM, BCAT2, CCDC113, UNC119, MME, MELK, RAD51, HMMR, COX8A, INPP5F, PLBD1, MX1, ZNF462, GREM2, RNF20, TPD52L1, MTA1, CKS2, TRIP13, PIP5K1B, LMAN2L, PLCB1, IGF1, AZIN1, ZFP3, FOLR1, UBE2K, INTS7, HNRNPA0, MATN2, GPX7, HMGN3, ZMYND11, PAM, HMGCR, MBNL2, EIF4A2, RPL15, CCDC115, NRCAM, PIGX, GSTK1, PIP5KL1, TRA2B, EPHB3, PFKM, ZNF84, PGRMC1, SLC23A2, STXBP6, CLOCK, TRIB2, DROSHA, CXXC1, NFIA) | 43.1% (40.7, 45.4) / 100.0% (100.0, 100.0) | 0.8% (0.7, 1.0) / — | — / 0.0% (0.0, 0.0) | — / 0.0% (0.0, 0.0) | \| | \| |  |  |  |
| │ ├── CliXO:434 (GLA, GLE1, LIN7A, LRRC41, TNFAIP8L1, KIAA0232, ODF2, DNMT3A, MTPN, HSPA2, CDK20, OVGP1, DNASE2, SUV39H1, TTLL5, OAS3, HMGB3, PPM1M, ST6GALNAC1, SLC39A8, MOB3C, DDX17, PEBP1, TKFC, SRPK2, CDC42BPA, PODXL, NIPAL3, PCCB, HJURP, RAD51, HMMR, MX1, TRIP13, LMAN2L, PLCB1, ZMYND11, HMGCR, RPL15, GSTK1) | 72.0% (68.4, 75.7) / 76.7% (73.8, 79.6) | 0.8% (0.7, 1.0) / — | 81.7% (79.8, 83.5) / 77.3% (74.3, 80.4) | — / 0.0% (0.0, 0.0) | \| | \| |  |  |  |
| │ │ └── CliXO:412 (PODXL, NIPAL3) | 72.0% (68.4, 75.7) / 76.7% (73.8, 79.6) | 0.8% (0.7, 1.0) / — | 81.7% (79.8, 83.5) / 77.3% (74.3, 80.4) | — / 0.0% (0.0, 0.0) | \| | \| |  |  |  |
| │ ├── CliXO:439 (MXRA5, FBXO9, POLR2H, ILF2, KDM1A, THOP1, SUMO3, SH3PXD2A, CIB2, COL1A2, HDAC1, KIF17, GNG7, GPER1, AMD1, COL6A1, COPZ2, TOP2B, CTSV, FAM172A, GRINA, KIAA0232, XPO4, HEXB, IGSF8, CNPY4, GOSR1, CCND1, MAD2L2, FZD1, BAD, CLCN5, C4orf3, PUS7, FBN2, PPT1, PJA1, CBX5, FBLN7, PALLD, DDR2, GEMIN5, FBLIM1, AMOTL2, LHFPL2, PDGFC, DKK3, MID1, PKM, UNC119, MME, COX8A, INPP5F, IGF1, INTS7, GPX7, MBNL2, NRCAM, PIP5KL1, TRA2B, EPHB3, PFKM, PGRMC1, SLC23A2, CLOCK, CXXC1, NFIA) | 43.1% (40.7, 45.4) / 100.0% (100.0, 100.0) | 0.8% (0.7, 1.0) / — | — / 0.0% (0.0, 0.0) | — / 0.0% (0.0, 0.0) | \| | \| |  |  |  |
| │ └── CliXO:446 (IFIT1, MXRA5, FBXO9, SLC25A4, TMEM216, PPA1, POLR2H, ILF2, KDM1A, THRA, CASD1, FKBP4, THOP1, SUMO3, SH3PXD2A, ALDH1B1, DLX5, CIB2, COL1A2, GLA, HDAC1, MRI1, DCXR, KIF17, LSM6, GNG7, GPER1, AMD1, GLE1, ZNF512B, COQ2, COL6A1, PHKA2, CLIC5, LIN7A, CTSF, COPZ2, TOP2B, CTSV, LRRC41, SPON1, ASTE1, DONSON, TNFAIP8L1, FAM172A, BRCA1, CHEK1, TSPAN3, GRINA, KIAA0232, TMEM106B, ODF2, XPO4, CDO1, SULF2, DNMT3A, CALM1, MTPN, HLF, SH3BGRL, CLIC3, HSPA2, WFS1, NRIP1, PIK3R1, TBC1D5, FGF13, TYRO3, RBM26, TRIM25, HEXB, CDK20, OVGP1, TAF9B, SMARCA2, DNASE2, IGSF8, SUV39H1, CNPY4, TTLL5, OAS3, GOSR1, HMGB3, TRIM24, PPM1M, ST6GALNAC1, CCND1, SLC39A8, MOB3C, CLCN5, C4orf3, RDX, PCYOX1, PUS7, DDX17, FBN2, PPT1, PJA1, PEBP1, TKFC, CBX5, SRPK2, FBLN7, PALLD, CDC42BPA, DDR2, GEMIN5, AMOTL2, LHFPL2, PDGFC, DKK3, MID1, NIPAL3, PCCB, ATXN10, HJURP, SHANK2, BCAT2, CCDC113, UNC119, MELK, RAD51, HMMR, COX8A, INPP5F, PLBD1, MX1, ZNF462, GREM2, RNF20, TPD52L1, MTA1, CKS2, TRIP13, PIP5K1B, LMAN2L, PLCB1, IGF1, AZIN1, ZFP3, FOLR1, UBE2K, INTS7, HNRNPA0, MATN2, GPX7, HMGN3, ZMYND11, PAM, HMGCR, MBNL2, EIF4A2, RPL15, CCDC115, NRCAM, PIGX, GSTK1, PIP5KL1, TRA2B, EPHB3, PFKM, ZNF84, PGRMC1, SLC23A2, STXBP6, CLOCK, TRIB2, DROSHA, CXXC1, NFIA) | 43.1% (40.7, 45.4) / 100.0% (100.0, 100.0) | 0.8% (0.7, 1.0) / — | — / 0.0% (0.0, 0.0) | — / 0.0% (0.0, 0.0) | \| | \| |  |  |  |
| │ ├── CliXO:419 (CTSF, OVGP1, SLC39A8, RDX, CDC42BPA, NIPAL3, HMGCR, RPL15, GSTK1) | 72.0% (68.4, 75.7) / 76.7% (73.8, 79.6) | 0.8% (0.7, 1.0) / — | 81.7% (79.8, 83.5) / 77.3% (74.3, 80.4) | — / 0.0% (0.0, 0.0) | \| | \| |  |  |  |
| │ ├── CliXO:421 (FBXO9, SLC25A4, KIF17, LIN7A, LRRC41, GRINA, DNASE2, IGSF8, UNC119, PIP5KL1) | 72.0% (68.4, 75.7) / 76.7% (73.8, 79.6) | 0.8% (0.7, 1.0) / — | 81.7% (79.8, 83.5) / 77.3% (74.3, 80.4) | — / 0.0% (0.0, 0.0) | \| | \| |  |  |  |
| │ └── CliXO:445 (IFIT1, MXRA5, FBXO9, SLC25A4, TMEM216, PPA1, POLR2H, ILF2, KDM1A, FKBP4, THOP1, SUMO3, SH3PXD2A, ALDH1B1, CIB2, COL1A2, GLA, HDAC1, MRI1, DCXR, KIF17, LSM6, GNG7, GPER1, AMD1, GLE1, ZNF512B, COL6A1, PHKA2, CLIC5, LIN7A, CTSF, COPZ2, TOP2B, CTSV, LRRC41, ASTE1, DONSON, TNFAIP8L1, FAM172A, TSPAN3, GRINA, KIAA0232, ODF2, XPO4, CDO1, SULF2, DNMT3A, CALM1, MTPN, HSPA2, WFS1, TBC1D5, TYRO3, TRIM25, HEXB, CDK20, OVGP1, TAF9B, DNASE2, SUV39H1, CNPY4, TTLL5, OAS3, GOSR1, HMGB3, TRIM24, PPM1M, CCND1, SLC39A8, MOB3C, CLCN5, C4orf3, RDX, PUS7, DDX17, FBN2, PPT1, PJA1, PEBP1, TKFC, CBX5, SRPK2, FBLN7, PALLD, CDC42BPA, DDR2, GEMIN5, AMOTL2, LHFPL2, PDGFC, DKK3, MID1, PCCB, ATXN10, HJURP, SHANK2, BCAT2, CCDC113, UNC119, MELK, RAD51, HMMR, COX8A, INPP5F, PLBD1, MX1, ZNF462, GREM2, RNF20, TPD52L1, MTA1, CKS2, TRIP13, PIP5K1B, LMAN2L, PLCB1, IGF1, AZIN1, ZFP3, FOLR1, UBE2K, INTS7, HNRNPA0, MATN2, GPX7, HMGN3, ZMYND11, PAM, HMGCR, MBNL2, EIF4A2, RPL15, CCDC115, NRCAM, PIGX, GSTK1, PIP5KL1, TRA2B, EPHB3, PFKM, ZNF84, PGRMC1, SLC23A2, STXBP6, CLOCK, TRIB2, DROSHA, CXXC1, NFIA) | 43.1% (40.7, 45.4) / 100.0% (100.0, 100.0) | 0.8% (0.7, 1.0) / — | — / 0.0% (0.0, 0.0) | — / 0.0% (0.0, 0.0) | \| | \| |  |  |  |
| └── CliXO:453 (ABCA1, ANKRD13A, ZFAND5, PANX1, RASEF, LAPTM5, RNF11, ACKR2, TBXAS1, ETS2, ARRDC3, BHLHE40, MTMR6, ARHGEF12, STRN3, ELF4, RAPH1, ENG, EPS8L2, RGS16, GRHL1, MBD2, OXSR1, CTSA, NCEH1, VEGFA, AKAP13, TMEM154, LDLR, JUNB, YPEL2, CDYL2, TES, MAFF, CEBPD, GALE, SMIM3, SH3BP5, LYN, CSF2RB, TSC22D1, GULP1, CDKN1B, GADD45G, CORO2A, GPX3, NEK7, PRRG4, MINPP1, MXD1, SLC1A1, FAM43A, ZNF217, CHIC2, PPP2R5A, SLC2A1, PIK3CG, ZNF281, PIK3AP1, SLC38A6, TMEM176B, SNAPC1, SPTY2D1, HILPDA, STAB1, NR4A3, ZFYVE1, TIMP3, MAP3K8, CORO1A, SYNJ2, SLC38A1, RFK, PRKCH, RNF19A, ERP44, PPL, PGBD2, LATS2, HTRA1, LRRC8A, PAPPA, TREM1, KPNA1, TYROBP, NUCB2, LCP1, GBE1, PGK1, CPEB4, LURAP1L, EAF1, DUSP4, TSPAN12, RBM47, OPTN, ANKRD37, VDR, FAM210A, MXI1, DUSP1, SLC7A2, TEX2, PIK3CB, PLIN2, LPL, SAMD8, ITGA5, TSPAN13, KIAA0513, IGSF3, ST6GAL1, SPAG4, SH2B3, ANXA1, HEY1, TGFBR1, TANK, STAT4, LYPD3, WDSUB1, PREP, CX3CR1, TPP1, RNASE1, GNG4, SGMS2, TMEM30A, DDIT4, SEMA4B, PTPN12, DOCK8, KLF4, UBR1, EPS8L1, BCAR3, SPI1, TP53BP2, SWAP70, FLI1, C9orf72, TSPAN14, PDZD2, CTBS, TMCC3, EDN1, ZNF143, FNDC3A, TRAK2, MYO5A, GRAMD1A, SLCO2B1, RHOBTB3, DNAJB9, ARHGDIB, SARAF, NFIL3, CYFIP2, MED13L, WDFY3, SMPDL3A, EPS15, MEF2A, EFHD1, HNMT, AMPD3, CRISPLD2, CDK9, SLC9A9, C3AR1, FBXO8, USP3, PTBP3, COL17A1, FOSL2, ELL2, LRRC32, SLCO4A1, CRYAB, HPGDS, SLC31A2, LY86, UBALD2, TNFAIP8, RNF24, SLC17A5, ARHGAP28, RCBTB1, PROM1, SERPINB9, SLC16A3, MAOB, NPC1, NARF, SBF2, ELMO1, ASAP2, KLF3, LPXN, MYO1F, RIN2, ARHGAP24, MAP4K4, CYBB, OSMR, DPYD, SACM1L, PLOD2, SNAP23, STK17A, CASP4, ERO1A, EDEM1, RAB8B, CSF3R, FAM107B, DEGS1, MAML2, TOR1B, MICU3, CPD, PTPRJ, FRMD4A, CITED2) | 43.1% (40.7, 45.4) / 100.0% (100.0, 100.0) | 0.8% (0.7, 1.0) / — | — / 0.0% (0.0, 0.0) | — / 0.0% (0.0, 0.0) | \| | \| |  |  |  |
| ├── CliXO:428 (ANKRD13A, RASEF, ACKR2, TBXAS1, RGS16, OXSR1, GALE, CORO2A, PPP2R5A, ZFYVE1, RBM47, OPTN, IGSF3, TANK, SPI1, TSPAN14, TRAK2, AMPD3, UBALD2, SLC17A5, ASAP2, ERO1A, CSF3R, CPD) | 72.0% (68.4, 75.7) / 76.7% (73.8, 79.6) | 0.8% (0.7, 1.0) / — | 81.7% (79.8, 83.5) / 77.3% (74.3, 80.4) | — / 0.0% (0.0, 0.0) | \| | \| |  |  |  |
| │ └── CliXO:408 (RBM47, IGSF3) | 72.0% (68.4, 75.7) / 76.7% (73.8, 79.6) | 0.8% (0.7, 1.0) / — | 81.7% (79.8, 83.5) / 77.3% (74.3, 80.4) | — / 0.0% (0.0, 0.0) | \| | \| |  |  |  |
| ├── CliXO:433 (VEGFA, ZNF281, SLC38A6, SNAPC1, HILPDA, SYNJ2, PPL, TREM1, PGK1, DUSP4, ANKRD37, VDR, ITGA5, TSPAN13, SPAG4, ANXA1, HEY1, LYPD3, SGMS2, SEMA4B, BCAR3, TP53BP2, MYO5A, AMPD3, USP3, COL17A1, SLCO4A1, SLC31A2, RNF24, SLC17A5, SLC16A3, NPC1, MYO1F, ARHGAP24, OSMR, PLOD2, ERO1A) | 72.0% (68.4, 75.7) / 76.7% (73.8, 79.6) | 0.8% (0.7, 1.0) / — | 81.7% (79.8, 83.5) / 77.3% (74.3, 80.4) | — / 0.0% (0.0, 0.0) | \| | \| |  |  |  |
| └── CliXO:452 (ABCA1, ANKRD13A, ZFAND5, PANX1, RASEF, LAPTM5, RNF11, ACKR2, TBXAS1, ETS2, ARRDC3, BHLHE40, MTMR6, ARHGEF12, STRN3, ELF4, RAPH1, ENG, EPS8L2, RGS16, GRHL1, MBD2, OXSR1, CTSA, NCEH1, VEGFA, AKAP13, TMEM154, LDLR, JUNB, YPEL2, CDYL2, TES, MAFF, CEBPD, GALE, SMIM3, SH3BP5, LYN, CSF2RB, TSC22D1, GULP1, CDKN1B, GADD45G, CORO2A, GPX3, NEK7, PRRG4, MINPP1, MXD1, SLC1A1, FAM43A, ZNF217, CHIC2, PPP2R5A, SLC2A1, PIK3CG, ZNF281, PIK3AP1, SLC38A6, TMEM176B, SNAPC1, SPTY2D1, HILPDA, STAB1, NR4A3, ZFYVE1, TIMP3, MAP3K8, CORO1A, SYNJ2, SLC38A1, RFK, PRKCH, RNF19A, ERP44, PPL, PGBD2, LATS2, HTRA1, LRRC8A, PAPPA, TREM1, KPNA1, TYROBP, NUCB2, LCP1, GBE1, PGK1, CPEB4, LURAP1L, EAF1, DUSP4, TSPAN12, OPTN, ANKRD37, VDR, FAM210A, MXI1, DUSP1, SLC7A2, TEX2, PIK3CB, PLIN2, LPL, SAMD8, ITGA5, TSPAN13, KIAA0513, IGSF3, ST6GAL1, SPAG4, SH2B3, ANXA1, TGFBR1, TANK, STAT4, LYPD3, WDSUB1, PREP, CX3CR1, TPP1, RNASE1, GNG4, SGMS2, TMEM30A, DDIT4, SEMA4B, PTPN12, DOCK8, KLF4, UBR1, EPS8L1, BCAR3, SPI1, TP53BP2, SWAP70, FLI1, C9orf72, TSPAN14, PDZD2, CTBS, TMCC3, EDN1, ZNF143, FNDC3A, TRAK2, MYO5A, GRAMD1A, SLCO2B1, RHOBTB3, DNAJB9, ARHGDIB, SARAF, NFIL3, CYFIP2, MED13L, WDFY3, SMPDL3A, EPS15, MEF2A, EFHD1, HNMT, AMPD3, CRISPLD2, CDK9, SLC9A9, C3AR1, FBXO8, USP3, PTBP3, COL17A1, FOSL2, ELL2, LRRC32, SLCO4A1, CRYAB, HPGDS, SLC31A2, LY86, UBALD2, TNFAIP8, RNF24, SLC17A5, ARHGAP28, RCBTB1, PROM1, SERPINB9, SLC16A3, MAOB, NPC1, NARF, SBF2, ELMO1, ASAP2, KLF3, LPXN, MYO1F, RIN2, ARHGAP24, MAP4K4, CYBB, OSMR, DPYD, SACM1L, PLOD2, SNAP23, STK17A, CASP4, ERO1A, EDEM1, RAB8B, CSF3R, FAM107B, DEGS1, MAML2, TOR1B, MICU3, CPD, PTPRJ, FRMD4A, CITED2) | 72.0% (68.4, 75.7) / 76.7% (73.8, 79.6) | 0.8% (0.7, 1.0) / — | 81.7% (79.8, 83.5) / 77.3% (74.3, 80.4) | — / 0.0% (0.0, 0.0) | \| | \| |  |  |  |
| ├── CliXO:444 (PANX1, RNF11, ARRDC3, BHLHE40, MTMR6, STRN3, NCEH1, VEGFA, JUNB, MAFF, SMIM3, CSF2RB, TSC22D1, GULP1, CDKN1B, NEK7, MINPP1, MXD1, FAM43A, ZNF217, SLC2A1, ZNF281, SLC38A6, TMEM176B, SNAPC1, SPTY2D1, HILPDA, NR4A3, MAP3K8, SYNJ2, RFK, PPL, LATS2, HTRA1, TREM1, LCP1, PGK1, EAF1, DUSP4, ANKRD37, VDR, MXI1, DUSP1, ITGA5, TSPAN13, SPAG4, SH2B3, ANXA1, STAT4, LYPD3, WDSUB1, PREP, GNG4, SGMS2, TMEM30A, DDIT4, SEMA4B, KLF4, UBR1, EPS8L1, BCAR3, TP53BP2, C9orf72, FNDC3A, MYO5A, RHOBTB3, DNAJB9, ARHGDIB, SARAF, CYFIP2, MED13L, WDFY3, MEF2A, EFHD1, HNMT, AMPD3, CRISPLD2, SLC9A9, USP3, COL17A1, ELL2, LRRC32, SLCO4A1, HPGDS, SLC31A2, TNFAIP8, RNF24, SLC17A5, ARHGAP28, RCBTB1, SERPINB9, SLC16A3, MAOB, NPC1, NARF, ELMO1, LPXN, MYO1F, ARHGAP24, MAP4K4, OSMR, SACM1L, PLOD2, STK17A, CASP4, ERO1A, MICU3) | 72.0% (68.4, 75.7) / 76.7% (73.8, 79.6) | 0.8% (0.7, 1.0) / — | 81.7% (79.8, 83.5) / 77.3% (74.3, 80.4) | — / 0.0% (0.0, 0.0) | \| | \| |  |  |  |
| └── CliXO:451 (ABCA1, ANKRD13A, ZFAND5, PANX1, RASEF, LAPTM5, RNF11, ACKR2, TBXAS1, ETS2, ARRDC3, BHLHE40, MTMR6, ARHGEF12, STRN3, ELF4, RAPH1, ENG, EPS8L2, RGS16, GRHL1, MBD2, OXSR1, CTSA, NCEH1, VEGFA, AKAP13, TMEM154, LDLR, JUNB, YPEL2, CDYL2, TES, MAFF, CEBPD, GALE, SMIM3, SH3BP5, LYN, CSF2RB, TSC22D1, GULP1, CDKN1B, GADD45G, CORO2A, GPX3, NEK7, PRRG4, MINPP1, MXD1, SLC1A1, ZNF217, CHIC2, PPP2R5A, SLC2A1, PIK3CG, ZNF281, PIK3AP1, TMEM176B, SNAPC1, SPTY2D1, HILPDA, STAB1, NR4A3, ZFYVE1, TIMP3, MAP3K8, CORO1A, SYNJ2, SLC38A1, RFK, PRKCH, RNF19A, ERP44, PPL, PGBD2, LATS2, HTRA1, LRRC8A, PAPPA, TREM1, KPNA1, TYROBP, NUCB2, LCP1, GBE1, PGK1, CPEB4, LURAP1L, EAF1, DUSP4, TSPAN12, OPTN, ANKRD37, VDR, FAM210A, MXI1, DUSP1, SLC7A2, TEX2, PIK3CB, PLIN2, LPL, SAMD8, ITGA5, TSPAN13, KIAA0513, IGSF3, ST6GAL1, SPAG4, SH2B3, TGFBR1, TANK, STAT4, WDSUB1, PREP, CX3CR1, TPP1, RNASE1, GNG4, SGMS2, TMEM30A, DDIT4, SEMA4B, PTPN12, DOCK8, KLF4, UBR1, EPS8L1, BCAR3, SPI1, TP53BP2, SWAP70, FLI1, C9orf72, TSPAN14, PDZD2, CTBS, TMCC3, EDN1, ZNF143, FNDC3A, TRAK2, MYO5A, GRAMD1A, SLCO2B1, RHOBTB3, DNAJB9, ARHGDIB, SARAF, NFIL3, CYFIP2, MED13L, WDFY3, SMPDL3A, EPS15, MEF2A, EFHD1, HNMT, AMPD3, CRISPLD2, CDK9, SLC9A9, C3AR1, FBXO8, USP3, PTBP3, COL17A1, FOSL2, ELL2, LRRC32, SLCO4A1, CRYAB, HPGDS, SLC31A2, LY86, UBALD2, TNFAIP8, RNF24, SLC17A5, ARHGAP28, RCBTB1, PROM1, SERPINB9, SLC16A3, MAOB, NPC1, NARF, SBF2, ELMO1, ASAP2, KLF3, LPXN, MYO1F, RIN2, ARHGAP24, MAP4K4, CYBB, OSMR, DPYD, SACM1L, PLOD2, SNAP23, STK17A, CASP4, ERO1A, EDEM1, RAB8B, CSF3R, FAM107B, DEGS1, MAML2, TOR1B, CPD, PTPRJ, FRMD4A, CITED2) | 72.0% (68.4, 75.7) / 76.7% (73.8, 79.6) | 0.8% (0.7, 1.0) / — | 81.7% (79.8, 83.5) / 77.3% (74.3, 80.4) | — / 0.0% (0.0, 0.0) | \| | \| |  |  |  |
| ├── CliXO:440 (ABCA1, ZFAND5, PANX1, LAPTM5, ACKR2, ETS2, ARRDC3, ARHGEF12, RGS16, GRHL1, OXSR1, NCEH1, AKAP13, YPEL2, TES, MAFF, SH3BP5, LYN, TSC22D1, GADD45G, MXD1, ZNF217, CHIC2, SLC2A1, PIK3AP1, SPTY2D1, MAP3K8, PRKCH, RNF19A, LATS2, HTRA1, LRRC8A, EAF1, MXI1, SLC7A2, TEX2, PLIN2, LPL, SAMD8, TSPAN13, ST6GAL1, TGFBR1, STAT4, TPP1, GNG4, DDIT4, SEMA4B, PTPN12, SWAP70, FLI1, FNDC3A, GRAMD1A, SARAF, NFIL3, EPS15, MEF2A, PTBP3, FOSL2, ELL2, LRRC32, CRYAB, UBALD2, RNF24, PROM1, NARF, ASAP2, RIN2, CYBB, CASP4, RAB8B, FAM107B, MAML2, TOR1B, FRMD4A) | 72.0% (68.4, 75.7) / 76.7% (73.8, 79.6) | 0.8% (0.7, 1.0) / — | 81.7% (79.8, 83.5) / 77.3% (74.3, 80.4) | — / 0.0% (0.0, 0.0) | \| | \| |  |  |  |
| │ └── CliXO:431 (ABCA1, ZFAND5, PANX1, ETS2, ARHGEF12, NCEH1, TES, SH3BP5, TSC22D1, SLC2A1, MAP3K8, RNF19A, HTRA1, LRRC8A, EAF1, MXI1, PLIN2, LPL, ST6GAL1, FLI1, GRAMD1A, SARAF, EPS15, PTBP3, ELL2, PROM1, ASAP2, RAB8B, FAM107B, MAML2, TOR1B) | 72.0% (68.4, 75.7) / 76.7% (73.8, 79.6) | 0.8% (0.7, 1.0) / — | 81.7% (79.8, 83.5) / 77.3% (74.3, 80.4) | — / 0.0% (0.0, 0.0) | \| | \| |  |  | CliXO:449 |
| └── CliXO:450 (ABCA1, ANKRD13A, ZFAND5, PANX1, RASEF, LAPTM5, RNF11, ACKR2, TBXAS1, ETS2, ARRDC3, BHLHE40, MTMR6, ARHGEF12, STRN3, ELF4, RAPH1, ENG, EPS8L2, RGS16, GRHL1, MBD2, OXSR1, CTSA, NCEH1, VEGFA, AKAP13, TMEM154, LDLR, JUNB, YPEL2, CDYL2, TES, MAFF, CEBPD, GALE, SMIM3, SH3BP5, LYN, CSF2RB, TSC22D1, GULP1, CDKN1B, GADD45G, CORO2A, GPX3, NEK7, PRRG4, MINPP1, MXD1, SLC1A1, ZNF217, CHIC2, PPP2R5A, SLC2A1, PIK3CG, ZNF281, PIK3AP1, TMEM176B, SNAPC1, SPTY2D1, HILPDA, STAB1, NR4A3, ZFYVE1, TIMP3, MAP3K8, CORO1A, SYNJ2, SLC38A1, RFK, PRKCH, RNF19A, ERP44, PPL, PGBD2, LATS2, HTRA1, LRRC8A, TREM1, TYROBP, LCP1, GBE1, PGK1, CPEB4, LURAP1L, EAF1, DUSP4, TSPAN12, OPTN, ANKRD37, VDR, FAM210A, MXI1, DUSP1, SLC7A2, TEX2, PIK3CB, PLIN2, SAMD8, ITGA5, TSPAN13, IGSF3, ST6GAL1, SPAG4, SH2B3, TGFBR1, TANK, STAT4, WDSUB1, PREP, CX3CR1, TPP1, GNG4, SGMS2, TMEM30A, DDIT4, SEMA4B, PTPN12, DOCK8, KLF4, UBR1, EPS8L1, BCAR3, SPI1, TP53BP2, SWAP70, FLI1, C9orf72, TSPAN14, PDZD2, CTBS, TMCC3, EDN1, ZNF143, FNDC3A, TRAK2, MYO5A, GRAMD1A, SLCO2B1, RHOBTB3, DNAJB9, ARHGDIB, SARAF, NFIL3, CYFIP2, MED13L, SMPDL3A, EPS15, MEF2A, EFHD1, AMPD3, CRISPLD2, CDK9, SLC9A9, C3AR1, FBXO8, USP3, PTBP3, COL17A1, FOSL2, ELL2, LRRC32, SLCO4A1, CRYAB, HPGDS, SLC31A2, LY86, UBALD2, TNFAIP8, RNF24, SLC17A5, RCBTB1, PROM1, SERPINB9, SLC16A3, MAOB, NPC1, NARF, SBF2, ELMO1, ASAP2, KLF3, LPXN, MYO1F, RIN2, ARHGAP24, MAP4K4, CYBB, OSMR, DPYD, SACM1L, PLOD2, SNAP23, STK17A, CASP4, ERO1A, EDEM1, RAB8B, CSF3R, FAM107B, DEGS1, MAML2, TOR1B, CPD, PTPRJ, FRMD4A, CITED2) | 66.1% (63.2, 69.0) / 83.9% (81.2, 86.6) | 0.8% (0.7, 1.0) / — | 84.9% (82.4, 87.4) / 67.6% (65.0, 70.2) | — / 0.0% (0.0, 0.0) | \| | \| |  |  |  |
| ├── CliXO:426 (CORO1A, STAT4, PREP, GNG4, RHOBTB3, ARHGDIB, SARAF, CYFIP2, MEF2A, EFHD1, SLC9A9, HPGDS, LY86, RCBTB1, MAOB, ELMO1, LPXN) | 72.0% (68.4, 75.7) / 76.7% (73.8, 79.6) | 0.8% (0.7, 1.0) / — | 81.7% (79.8, 83.5) / 77.3% (74.3, 80.4) | — / 0.0% (0.0, 0.0) | \| | \| |  |  |  |
| ├── CliXO:429 (ANKRD13A, RASEF, TBXAS1, RAPH1, ENG, RGS16, GRHL1, CTSA, OPTN, IGSF3, EPS8L1, SPI1, TSPAN14, AMPD3, PTBP3, COL17A1, SLC31A2, UBALD2, RNF24, SLC17A5, SLC16A3, ASAP2, MAP4K4, PLOD2, CASP4, ERO1A, CPD, PTPRJ) | 72.0% (68.4, 75.7) / 76.7% (73.8, 79.6) | 0.8% (0.7, 1.0) / — | 81.7% (79.8, 83.5) / 77.3% (74.3, 80.4) | — / 0.0% (0.0, 0.0) | \| | \| |  |  | CliXO:449 |
| └── CliXO:449 (ABCA1, ANKRD13A, ZFAND5, PANX1, RASEF, LAPTM5, RNF11, ACKR2, TBXAS1, ETS2, ARRDC3, BHLHE40, MTMR6, ARHGEF12, STRN3, ELF4, RAPH1, ENG, EPS8L2, RGS16, GRHL1, MBD2, OXSR1, CTSA, NCEH1, VEGFA, AKAP13, TMEM154, LDLR†, JUNB, YPEL2, CDYL2, TES, MAFF, CEBPD, GALE, SMIM3, SH3BP5, LYN, CSF2RB, TSC22D1, GULP1, CDKN1B, GADD45G, CORO2A, GPX3, NEK7, PRRG4, MINPP1, MXD1, SLC1A1, ZNF217, CHIC2, SLC2A1, PIK3CG, ZNF281, PIK3AP1, TMEM176B, SNAPC1, SPTY2D1, HILPDA, STAB1, NR4A3, TIMP3, MAP3K8, CORO1A, SYNJ2, SLC38A1, RFK, PRKCH, RNF19A, ERP44, PPL†, LATS2, HTRA1, LRRC8A, TREM1, TYROBP, LCP1, GBE1, PGK1, CPEB4, EAF1, DUSP4, TSPAN12, OPTN, ANKRD37, VDR, FAM210A, MXI1, DUSP1, SLC7A2, TEX2, PIK3CB, PLIN2, SAMD8, TSPAN13, ST6GAL1, SPAG4, SH2B3, TGFBR1, STAT4, WDSUB1, PREP, CX3CR1, TPP1, GNG4, SGMS2, TMEM30A, DDIT4, SEMA4B, PTPN12, DOCK8, KLF4, UBR1, EPS8L1, BCAR3, SPI1, TP53BP2, SWAP70, FLI1, C9orf72, TSPAN14, PDZD2, CTBS, TMCC3, EDN1, ZNF143, FNDC3A, MYO5A, GRAMD1A, SLCO2B1, RHOBTB3, DNAJB9, ARHGDIB, SARAF, NFIL3, CYFIP2, MED13L, SMPDL3A, EPS15, MEF2A, EFHD1, AMPD3, CRISPLD2, CDK9, SLC9A9, C3AR1, FBXO8, USP3, PTBP3†, COL17A1, FOSL2, ELL2, LRRC32†, SLCO4A1, CRYAB, HPGDS, SLC31A2, LY86, UBALD2, TNFAIP8, RNF24, SLC17A5, PROM1, SERPINB9, MAOB, NPC1, NARF, SBF2, ELMO1, ASAP2, KLF3, LPXN, MYO1F, RIN2, ARHGAP24, MAP4K4, CYBB, OSMR, DPYD, SACM1L, PLOD2, SNAP23†, STK17A, CASP4, ERO1A, EDEM1, RAB8B, CSF3R, FAM107B, DEGS1, MAML2, TOR1B, CPD, PTPRJ, FRMD4A, CITED2) | 46.2% (43.8, 48.6) / 93.8% (92.1, 95.4) | 0.9% (0.7, 1.1) / — | 77.1% (70.2, 83.9) / 17.2% (14.5, 19.9) | 100.0% (100.0, 100.0) / 6.5% (5.9, 7.2) | \|✓ | \|✓ | ✓ |  | CliXO:449 |

The Reported column indicates the ontology from Tables 1–2 to which this row’s eligible genes belong. An ontology is reported if it passes all criteria (C1–C4) and its eligible gene set is not a strict subset of another passing ontology’s gene set. The column is empty when the ontology has no eligible genes or does not pass all criteria. ✓, the ontology passed the criterion; †, the gene had a grad-CAM value >97.5th percentile in the same ontology; |, separator between PPV/TPR and NPV/TNR results in C1 and C2 columns; AUC-ROC, area under the receiver operating characteristic curve; C1, lower bounds finite and >0; C2, absolute difference ≤25% between development and test sets; C3, at least 1 eligible gene; C4, lower bound of AUC-ROC >0.5 in both sets; CI, confidence interval; Dev, development set; grad-CAM, gradient-weighted class activation mapping; HELLP, hemolysis, elevated liver enzyme, and low platelet; NPV, negative predictive value; PE, preeclampsia; PPV, positive predictive value; TNR, true negative rate; TPR, true positive rate.

Table S12. AUC-ROC, AUC-PRC, and RMSE for the early-onset, severe PE pathogenesis model connected with late-secretory endometrium.

| Ontology | AUC-ROC (Dev) | AUC-ROC (Test) | AUC-PRC (Dev) | AUC-PRC (Test) | RMSE (Dev) | RMSE (Test) | C1 | C2 | C3 | C4 | Reported |
| --- | --- | --- | --- | --- | --- | --- | --- | --- | --- | --- | --- |
| root (ABCA1, ANKRD13A, ZFAND5, PANX1, RASEF, LAPTM5, RNF11, ACKR2, TBXAS1, ETS2, ARRDC3, BHLHE40, MTMR6, ARHGEF12, STRN3, ELF4, RAPH1, ENG, EPS8L2, RGS16, GRHL1, MBD2, OXSR1, CTSA, NCEH1, VEGFA, AKAP13, TMEM154, LDLR, JUNB, YPEL2, CDYL2, TES, MAFF, CEBPD, GALE, SMIM3, SH3BP5, LYN, CSF2RB, TSC22D1, GULP1, CDKN1B, GADD45G, CORO2A, GPX3, NEK7, PRRG4, MINPP1, MXD1, SLC1A1, FAM43A, ZNF217, CHIC2, PPP2R5A, SLC2A1, PIK3CG, ZNF281, PIK3AP1, SLC38A6, TMEM176B, SNAPC1, SPTY2D1, HILPDA, IFIT1, STAB1, NR4A3, ZFYVE1, TIMP3, MAP3K8, CORO1A, SYNJ2, SLC38A1, RFK, PRKCH, RNF19A, ERP44, PPL, PGBD2, LATS2, HTRA1, LRRC8A, PAPPA, TREM1, KPNA1, TYROBP, MXRA5, NUCB2, LCP1, GBE1, PGK1, CPEB4, LURAP1L, EAF1, DUSP4, FBXO9, TSPAN12, RBM47, OPTN, ANKRD37, VDR, FAM210A, SLC25A4, MXI1, TMEM216, DUSP1, SLC7A2, TEX2, PIK3CB, PLIN2, LPL, SAMD8, ITGA5, TSPAN13, KIAA0513, IGSF3, ST6GAL1, SPAG4, SH2B3, ANXA1, PPA1, HEY1, TGFBR1, POLR2H, TANK, ILF2, STAT4, LYPD3, WDSUB1, KDM1A, PREP, CX3CR1, TPP1, RNASE1, GNG4, SGMS2, THRA, TMEM30A, DDIT4, SEMA4B, CASD1, PTPN12, FKBP4, DOCK8, KLF4, THOP1, UBR1, EPS8L1, BCAR3, SPI1, TP53BP2, SWAP70, FLI1, C9orf72, TSPAN14, SUMO3, PDZD2, CTBS, TMCC3, EDN1, ZNF143, FNDC3A, SH3PXD2A, TRAK2, ALDH1B1, DLX5, MYO5A, GRAMD1A, SLCO2B1, CIB2, RHOBTB3, DNAJB9, COL1A2, ARHGDIB, GLA, SARAF, NFIL3, HDAC1, MRI1, CYFIP2, MED13L, WDFY3, SMPDL3A, DCXR, EPS15, MEF2A, EFHD1, QPCT, HNMT, AMPD3, CRISPLD2, KIF17, CDK9, SLC9A9, C3AR1, FBXO8, LSM6, GNG7, GPER1, USP3, AMD1, PTBP3, COL17A1, GLE1, FOSL2, ELL2, LRRC32, ZNF512B, SLCO4A1, COQ2, COL6A1, PHKA2, CRYAB, CLIC5, HPGDS, SLC31A2, LIN7A, LY86, UBALD2, TNFAIP8, CTSF, COPZ2, TOP2B, CTSV, RNF24, SLC17A5, ARHGAP28, LRRC41, RCBTB1, PROM1, SERPINB9, SLC16A3, MAOB, SPON1, ASTE1, NPC1, DONSON, NARF, TNFAIP8L1, FAM172A, BRCA1, CHEK1, SBF2, ELMO1, TSPAN3, GRINA, KIAA0232, ASAP2, TMEM106B, ODF2, KLF3, XPO4, CDO1, LPXN, SULF2, MYO1F, DNMT3A, CALM1, MTPN, HLF, SH3BGRL, CLIC3, HSPA2, WFS1, NRIP1, SESN3, PIK3R1, TBC1D5, FGF13, RIN2, TYRO3, RBM26, TRIM25, HEXB, ARHGAP24, MAP4K4, CDK20, CYBB, OVGP1, TAF9B, OSMR, SMARCA2, DPYD, SACM1L, PLOD2, SNAP23, DNASE2, STK17A, CASP4, IGSF8, SUV39H1, CNPY4, TTLL5, ERO1A, OAS3, GOSR1, HMGB3, TRIM24, EDEM1, PPM1M, RAB8B, ST6GALNAC1, CCND1, MAD2L2, SLC39A8, FZD1, BAD, MOB3C, CLCN5, C4orf3, RDX, CSF3R, PCYOX1, PUS7, DDX17, FBN2, PPT1, PJA1, PEBP1, IL33, TKFC, CBX5, SRPK2, FBLN7, PALLD, CDC42BPA, CNN3, FAM107B, DDR2, DEGS1, GEMIN5, PODXL, FBLIM1, AMOTL2, LHFPL2, MAML2, PDGFC, DKK3, MID1, NIPAL3, PCCB, ATXN10, HJURP, SHANK2, TOR1B, PKM, BCAT2, CCDC113, UNC119, MME, MELK, RAD51, HMMR, MICU3, COX8A, INPP5F, PLBD1, MX1, CPD, ZNF462, GREM2, RNF20, TPD52L1, MTA1, PTPRJ, CKS2, TRIP13, PIP5K1B, LMAN2L, PLCB1, IGF1, FRMD4A, AZIN1, ZFP3, FOLR1, UBE2K, INTS7, CITED2, HNRNPA0, MATN2, GPX7, HMGN3, ZMYND11, PAM, HMGCR, MBNL2, EIF4A2, RPL15, CCDC115, NRCAM, PIGX, GSTK1, PIP5KL1, TRA2B, EPHB3, PFKM, ZNF84, PGRMC1, SLC23A2, STXBP6, CLOCK, TRIB2, DROSHA, CXXC1, NFIA) | 0.791 (0.771, 0.811) | 0.457 (0.376, 0.537) | 0.666 | 0.257 | 0.590 (±0.0122) | 0.867 (±0.0007) | \| | \| |  |  |  |
| CliXO:454 (ABCA1, ANKRD13A, ZFAND5, PANX1, RASEF, LAPTM5, RNF11, ACKR2, TBXAS1, ETS2, ARRDC3, BHLHE40, MTMR6, ARHGEF12, STRN3, ELF4, RAPH1, ENG, EPS8L2, RGS16, GRHL1, MBD2, OXSR1, CTSA, NCEH1, VEGFA, AKAP13, TMEM154, LDLR, JUNB, YPEL2, CDYL2, TES, MAFF, CEBPD, GALE, SMIM3, SH3BP5, LYN, CSF2RB, TSC22D1, GULP1, CDKN1B, GADD45G, CORO2A, GPX3, NEK7, PRRG4, MINPP1, MXD1, SLC1A1, FAM43A, ZNF217, CHIC2, PPP2R5A, SLC2A1, PIK3CG, ZNF281, PIK3AP1, SLC38A6, TMEM176B, SNAPC1, SPTY2D1, HILPDA, IFIT1, STAB1, NR4A3, ZFYVE1, TIMP3, MAP3K8, CORO1A, SYNJ2, SLC38A1, RFK, PRKCH, RNF19A, ERP44, PPL, PGBD2, LATS2, HTRA1, LRRC8A, PAPPA, TREM1, KPNA1, TYROBP, MXRA5, NUCB2, LCP1, GBE1, PGK1, CPEB4, LURAP1L, EAF1, DUSP4, FBXO9, TSPAN12, RBM47, OPTN, ANKRD37, VDR, FAM210A, SLC25A4, MXI1, TMEM216, DUSP1, SLC7A2, TEX2, PIK3CB, PLIN2, LPL, SAMD8, ITGA5, TSPAN13, KIAA0513, IGSF3, ST6GAL1, SPAG4, SH2B3, ANXA1, PPA1, HEY1, TGFBR1, POLR2H, TANK, ILF2, STAT4, LYPD3, WDSUB1, KDM1A, PREP, CX3CR1, TPP1, RNASE1, GNG4, SGMS2, THRA, TMEM30A, DDIT4, SEMA4B, CASD1, PTPN12, FKBP4, DOCK8, KLF4, THOP1, UBR1, EPS8L1, BCAR3, SPI1, TP53BP2, SWAP70, FLI1, C9orf72, TSPAN14, SUMO3, PDZD2, CTBS, TMCC3, EDN1, ZNF143, FNDC3A, SH3PXD2A, TRAK2, ALDH1B1, DLX5, MYO5A, GRAMD1A, SLCO2B1, CIB2, RHOBTB3, DNAJB9, COL1A2, ARHGDIB, GLA, SARAF, NFIL3, HDAC1, MRI1, CYFIP2, MED13L, WDFY3, SMPDL3A, DCXR, EPS15, MEF2A, EFHD1, QPCT, HNMT, AMPD3, CRISPLD2, KIF17, CDK9, SLC9A9, C3AR1, FBXO8, LSM6, GNG7, GPER1, USP3, AMD1, PTBP3, COL17A1, GLE1, FOSL2, ELL2, LRRC32, ZNF512B, SLCO4A1, COQ2, COL6A1, PHKA2, CRYAB, CLIC5, HPGDS, SLC31A2, LIN7A, LY86, UBALD2, TNFAIP8, CTSF, COPZ2, TOP2B, CTSV, RNF24, SLC17A5, ARHGAP28, LRRC41, RCBTB1, PROM1, SERPINB9, SLC16A3, MAOB, SPON1, ASTE1, NPC1, DONSON, NARF, TNFAIP8L1, FAM172A, BRCA1, CHEK1, SBF2, ELMO1, TSPAN3, GRINA, KIAA0232, ASAP2, TMEM106B, ODF2, KLF3, XPO4, CDO1, LPXN, SULF2, MYO1F, DNMT3A, CALM1, MTPN, HLF, SH3BGRL, CLIC3, HSPA2, WFS1, NRIP1, SESN3, PIK3R1, TBC1D5, FGF13, RIN2, TYRO3, RBM26, TRIM25, HEXB, ARHGAP24, MAP4K4, CDK20, CYBB, OVGP1, TAF9B, OSMR, SMARCA2, DPYD, SACM1L, PLOD2, SNAP23, DNASE2, STK17A, CASP4, IGSF8, SUV39H1, CNPY4, TTLL5, ERO1A, OAS3, GOSR1, HMGB3, TRIM24, EDEM1, PPM1M, RAB8B, ST6GALNAC1, CCND1, MAD2L2, SLC39A8, FZD1, BAD, MOB3C, CLCN5, C4orf3, RDX, CSF3R, PCYOX1, PUS7, DDX17, FBN2, PPT1, PJA1, PEBP1, IL33, TKFC, CBX5, SRPK2, FBLN7, PALLD, CDC42BPA, CNN3, FAM107B, DDR2, DEGS1, GEMIN5, PODXL, FBLIM1, AMOTL2, LHFPL2, MAML2, PDGFC, DKK3, MID1, NIPAL3, PCCB, ATXN10, HJURP, SHANK2, TOR1B, PKM, BCAT2, CCDC113, UNC119, MME, MELK, RAD51, HMMR, MICU3, COX8A, INPP5F, PLBD1, MX1, CPD, ZNF462, GREM2, RNF20, TPD52L1, MTA1, PTPRJ, CKS2, TRIP13, PIP5K1B, LMAN2L, PLCB1, IGF1, FRMD4A, AZIN1, ZFP3, FOLR1, UBE2K, INTS7, CITED2, HNRNPA0, MATN2, GPX7, HMGN3, ZMYND11, PAM, HMGCR, MBNL2, EIF4A2, RPL15, CCDC115, NRCAM, PIGX, GSTK1, PIP5KL1, TRA2B, EPHB3, PFKM, ZNF84, PGRMC1, SLC23A2, STXBP6, CLOCK, TRIB2, DROSHA, CXXC1, NFIA) | 0.275 (0.252, 0.298) | 0.450 (0.395, 0.505) | 0.201 | 0.504 | 0.570 (±0.0040) | 0.466 (±0.0001) | \| | \| |  |  |  |
| ├── CliXO:416 (KPNA1, NUCB2, ST6GALNAC1) | 0.770 (0.750, 0.791) | 0.450 (0.395, 0.505) | 0.535 | 0.504 | 0.438 (±0.0176) | 0.824 (±0.0006) | \| | \| |  |  |  |
| ├── CliXO:436 (CSF2RB, FAM43A, SLC38A6, PGK1, VDR, ANXA1, HEY1, LYPD3, CASD1, QPCT, COQ2, ARHGAP28, SPON1, BRCA1, CHEK1, TMEM106B, CDO1, HLF, SH3BGRL, CLIC3, WFS1, SESN3, PIK3R1, FGF13, RBM26, HEXB, SMARCA2, IGSF8, CNPY4, CCND1, MAD2L2, FZD1, C4orf3, PCYOX1, PJA1, IL33, CNN3, DDR2, FBLIM1, AMOTL2, LHFPL2, PDGFC, PKM, MME, MICU3, INPP5F) | 0.770 (0.750, 0.791) | 0.450 (0.395, 0.505) | 0.535 | 0.504 | 0.446 (±0.0189) | 0.873 (±0.0007) | \| | \| |  |  |  |
| │ ├── CliXO:410 (HEY1, LYPD3) | 0.770 (0.750, 0.791) | 0.450 (0.395, 0.505) | 0.535 | 0.504 | 0.436 (±0.0171) | 0.804 (±0.0006) | \| | \| |  |  |  |
| │ ├── CliXO:411 (ARHGAP28, SESN3) | 0.770 (0.750, 0.791) | 0.450 (0.395, 0.505) | 0.535 | 0.504 | 0.436 (±0.0171) | 0.804 (±0.0006) | \| | \| |  |  |  |
| │ ├── CliXO:415 (COQ2, SPON1, CHEK1) | 0.770 (0.750, 0.791) | 0.450 (0.395, 0.505) | 0.535 | 0.504 | 0.438 (±0.0176) | 0.825 (±0.0006) | \| | \| |  |  |  |
| │ ├── CliXO:420 (CSF2RB, FAM43A, SLC38A6, CASD1, QPCT, SH3BGRL, FGF13, IL33, CNN3, MICU3) | 0.770 (0.750, 0.791) | 0.450 (0.395, 0.505) | 0.535 | 0.504 | 0.441 (±0.0181) | 0.842 (±0.0007) | \| | \| |  |  |  |
| │ │ ├── CliXO:406 (SH3BGRL, FGF13) | 0.770 (0.750, 0.791) | 0.450 (0.395, 0.505) | 0.535 | 0.504 | 0.436 (±0.0170) | 0.802 (±0.0006) | \| | \| |  |  |  |
| │ │ └── CliXO:407 (FAM43A, QPCT) | 0.770 (0.750, 0.791) | 0.450 (0.395, 0.505) | 0.535 | 0.504 | 0.436 (±0.0171) | 0.804 (±0.0006) | \| | \| |  |  |  |
| │ └── CliXO:430 (FAM43A, SLC38A6, HEY1, CASD1, QPCT, SPON1, CHEK1, TMEM106B, SH3BGRL, CLIC3, SESN3, PIK3R1, FGF13, RBM26, SMARCA2, IGSF8, CCND1, MAD2L2, C4orf3, PJA1, IL33, CNN3, DDR2, FBLIM1, LHFPL2, PDGFC, PKM, MME, MICU3) | 0.770 (0.750, 0.791) | 0.450 (0.395, 0.505) | 0.535 | 0.504 | 0.442 (±0.0184) | 0.851 (±0.0007) | \| | \| |  |  |  |
| │ │ ├── CliXO:406 (SH3BGRL, FGF13) | 0.770 (0.750, 0.791) | 0.450 (0.395, 0.505) | 0.535 | 0.504 | 0.436 (±0.0170) | 0.802 (±0.0006) | \| | \| |  |  |  |
| │ │ └── CliXO:407 (FAM43A, QPCT) | 0.770 (0.750, 0.791) | 0.450 (0.395, 0.505) | 0.535 | 0.504 | 0.436 (±0.0171) | 0.804 (±0.0006) | \| | \| |  |  |  |
| │ └── CliXO:413 (LHFPL2, PKM) | 0.770 (0.750, 0.791) | 0.450 (0.395, 0.505) | 0.535 | 0.504 | 0.436 (±0.0171) | 0.804 (±0.0006) | \| | \| |  |  |  |
| ├── CliXO:442 (PANX1, RNF11, BHLHE40, MTMR6, STRN3, NCEH1, VEGFA, JUNB, SMIM3, CSF2RB, TSC22D1, GULP1, CDKN1B, MINPP1, MXD1, FAM43A, ZNF281, SLC38A6, TMEM176B, SNAPC1, SPTY2D1, HILPDA, NR4A3, MAP3K8, SYNJ2, PPL, LATS2, TREM1, PGK1, DUSP4, ANKRD37, VDR, DUSP1, ITGA5, TSPAN13, KIAA0513, SPAG4, SH2B3, ANXA1, HEY1, LYPD3, WDSUB1, SGMS2, TMEM30A, DDIT4, SEMA4B, CASD1, EPS8L1, BCAR3, TP53BP2, TMCC3, MYO5A, RHOBTB3, WDFY3, MEF2A, QPCT, HNMT, SLCO4A1, HPGDS, RNF24, ARHGAP28, RCBTB1, MAOB, SPON1, CHEK1, ELMO1, TMEM106B, LPXN, SH3BGRL, CLIC3, SESN3, PIK3R1, FGF13, RBM26, SACM1L, MAD2L2, IL33, CNN3, FBLIM1, LHFPL2, MAML2, PDGFC, PKM, MME, MICU3) | 0.770 (0.750, 0.791) | 0.450 (0.395, 0.505) | 0.535 | 0.504 | 0.440 (±0.0177) | 0.825 (±0.0006) | \| | \| |  |  |  |
| │ └── CliXO:441 (RNF11, BHLHE40, MTMR6, STRN3, NCEH1, VEGFA, JUNB, SMIM3, CSF2RB, TSC22D1, GULP1, CDKN1B, MINPP1, MXD1, FAM43A, ZNF281, SLC38A6, TMEM176B, SNAPC1, HILPDA, NR4A3, SYNJ2, PPL, LATS2, TREM1, PGK1, DUSP4, ANKRD37, VDR, DUSP1, ITGA5, SPAG4, SH2B3, ANXA1, HEY1, LYPD3, WDSUB1, SGMS2, SEMA4B, CASD1, BCAR3, TP53BP2, TMCC3, MYO5A, WDFY3, MEF2A, QPCT, HNMT, SLCO4A1, HPGDS, ARHGAP28, RCBTB1, MAOB, SPON1, CHEK1, ELMO1, TMEM106B, LPXN, SH3BGRL, CLIC3, SESN3, PIK3R1, FGF13, RBM26, SACM1L, MAD2L2, IL33, CNN3, FBLIM1, LHFPL2, MAML2, PDGFC, PKM, MME, MICU3) | 0.770 (0.750, 0.791) | 0.450 (0.395, 0.505) | 0.535 | 0.504 | 0.440 (±0.0176) | 0.823 (±0.0006) | \| | \| |  |  |  |
| │ ├── CliXO:417 (WDFY3, HNMT, ARHGAP28, RCBTB1, ELMO1, LPXN) | 0.770 (0.750, 0.791) | 0.450 (0.395, 0.505) | 0.535 | 0.504 | 0.440 (±0.0180) | 0.839 (±0.0007) | \| | \| |  |  |  |
| │ │ └── CliXO:405 (ARHGAP28, RCBTB1) | 0.770 (0.750, 0.791) | 0.450 (0.395, 0.505) | 0.535 | 0.504 | 0.436 (±0.0171) | 0.804 (±0.0006) | \| | \| |  |  |  |
| │ ├── CliXO:418 (PGK1, SPAG4, HEY1, LYPD3, BCAR3, SLCO4A1) | 0.770 (0.750, 0.791) | 0.450 (0.395, 0.505) | 0.535 | 0.504 | 0.440 (±0.0180) | 0.839 (±0.0007) | \| | \| |  |  |  |
| │ ├── CliXO:410 (HEY1, LYPD3) | 0.770 (0.750, 0.791) | 0.450 (0.395, 0.505) | 0.535 | 0.504 | 0.436 (±0.0171) | 0.804 (±0.0006) | \| | \| |  |  |  |
| │ └── CliXO:437 (RNF11, MTMR6, STRN3, JUNB, SMIM3, CSF2RB, TSC22D1, GULP1, CDKN1B, MINPP1, MXD1, FAM43A, ZNF281, SLC38A6, HILPDA, NR4A3, SYNJ2, TREM1, PGK1, DUSP4, ANKRD37, VDR, ITGA5, SPAG4, ANXA1, HEY1, LYPD3, WDSUB1, CASD1, BCAR3, QPCT, HNMT, ARHGAP28, RCBTB1, SPON1, CHEK1, TMEM106B, SH3BGRL, CLIC3, SESN3, PIK3R1, FGF13, RBM26, MAD2L2, IL33, CNN3, FBLIM1, LHFPL2, PDGFC, PKM, MME, MICU3) | 0.770 (0.750, 0.791) | 0.450 (0.395, 0.505) | 0.535 | 0.504 | 0.446 (±0.0189) | 0.870 (±0.0007) | \| | \| |  |  |  |
| │ │ └── CliXO:405 (ARHGAP28, RCBTB1) | 0.770 (0.750, 0.791) | 0.450 (0.395, 0.505) | 0.535 | 0.504 | 0.436 (±0.0171) | 0.804 (±0.0006) | \| | \| |  |  |  |
| │ └── CliXO:413 (LHFPL2, PKM) | 0.770 (0.750, 0.791) | 0.450 (0.395, 0.505) | 0.535 | 0.504 | 0.436 (±0.0171) | 0.804 (±0.0006) | \| | \| |  |  |  |
| │ └── CliXO:432 (RNF11, MTMR6, STRN3, SMIM3, CSF2RB, TSC22D1, GULP1, MXD1, FAM43A, SLC38A6, HILPDA, SYNJ2, TREM1, PGK1, DUSP4, VDR, ITGA5, ANXA1, HEY1, LYPD3, WDSUB1, CASD1, QPCT, ARHGAP28, SH3BGRL, SESN3, PIK3R1, FGF13, IL33, CNN3, FBLIM1, PKM, MME, MICU3) | 0.770 (0.750, 0.791) | 0.450 (0.395, 0.505) | 0.535 | 0.504 | 0.446 (±0.0189) | 0.873 (±0.0007) | \| | \| |  |  |  |
| │ ├── CliXO:409 (WDSUB1, MICU3) | 0.770 (0.750, 0.791) | 0.450 (0.395, 0.505) | 0.535 | 0.504 | 0.436 (±0.0171) | 0.804 (±0.0006) | \| | \| |  |  |  |
| │ ├── CliXO:410 (HEY1, LYPD3) | 0.770 (0.750, 0.791) | 0.450 (0.395, 0.505) | 0.535 | 0.504 | 0.436 (±0.0171) | 0.804 (±0.0006) | \| | \| |  |  |  |
| │ ├── CliXO:414 (ITGA5, ANXA1) | 0.770 (0.750, 0.791) | 0.450 (0.395, 0.505) | 0.535 | 0.504 | 0.436 (±0.0171) | 0.804 (±0.0006) | \| | \| |  |  |  |
| │ └── CliXO:425 (STRN3, SMIM3, CSF2RB, FAM43A, SLC38A6, HILPDA, CASD1, QPCT, ARHGAP28, SH3BGRL, SESN3, FGF13, IL33, CNN3, MME, MICU3) | 0.770 (0.750, 0.791) | 0.450 (0.395, 0.505) | 0.535 | 0.504 | 0.443 (±0.0185) | 0.858 (±0.0007) | \| | \| |  |  |  |
| │ ├── CliXO:411 (ARHGAP28, SESN3) | 0.770 (0.750, 0.791) | 0.450 (0.395, 0.505) | 0.535 | 0.504 | 0.436 (±0.0171) | 0.804 (±0.0006) | \| | \| |  |  |  |
| │ ├── CliXO:420 (CSF2RB, FAM43A, SLC38A6, CASD1, QPCT, SH3BGRL, FGF13, IL33, CNN3, MICU3) | 0.770 (0.750, 0.791) | 0.450 (0.395, 0.505) | 0.535 | 0.504 | 0.441 (±0.0181) | 0.842 (±0.0007) | \| | \| |  |  |  |
| │ │ ├── CliXO:406 (SH3BGRL, FGF13) | 0.770 (0.750, 0.791) | 0.450 (0.395, 0.505) | 0.535 | 0.504 | 0.436 (±0.0170) | 0.802 (±0.0006) | \| | \| |  |  |  |
| │ │ └── CliXO:407 (FAM43A, QPCT) | 0.770 (0.750, 0.791) | 0.450 (0.395, 0.505) | 0.535 | 0.504 | 0.436 (±0.0171) | 0.804 (±0.0006) | \| | \| |  |  |  |
| ├── CliXO:448 (IFIT1, MXRA5, FBXO9, SLC25A4, TMEM216, PPA1, POLR2H, ILF2, KDM1A, THRA, CASD1, FKBP4, THOP1, SUMO3, SH3PXD2A, ALDH1B1, DLX5, CIB2, COL1A2, GLA, HDAC1, MRI1, DCXR, QPCT, KIF17, LSM6, GNG7, GPER1, AMD1, GLE1, ZNF512B, COQ2, COL6A1, PHKA2, CLIC5, LIN7A, CTSF, COPZ2, TOP2B, CTSV, LRRC41, SPON1, ASTE1, DONSON, TNFAIP8L1, FAM172A, BRCA1, CHEK1, TSPAN3, GRINA, KIAA0232, TMEM106B, ODF2, XPO4, CDO1, SULF2, DNMT3A, CALM1, MTPN, HLF, SH3BGRL, CLIC3, HSPA2, WFS1, NRIP1, SESN3, PIK3R1, TBC1D5, FGF13, TYRO3, RBM26, TRIM25, HEXB, CDK20, OVGP1, TAF9B, SMARCA2, DNASE2, IGSF8, SUV39H1, CNPY4, TTLL5, OAS3, GOSR1, HMGB3, TRIM24, PPM1M, ST6GALNAC1, CCND1, MAD2L2, SLC39A8, FZD1, BAD, MOB3C, CLCN5, C4orf3, RDX, PCYOX1, PUS7, DDX17, FBN2, PPT1, PJA1, PEBP1, IL33, TKFC, CBX5, SRPK2, FBLN7, PALLD, CDC42BPA, CNN3, DDR2, GEMIN5, PODXL, FBLIM1, AMOTL2, LHFPL2, PDGFC, DKK3, MID1, NIPAL3, PCCB, ATXN10, HJURP, SHANK2, PKM, BCAT2, CCDC113, UNC119, MME, MELK, RAD51, HMMR, COX8A, INPP5F, PLBD1, MX1, ZNF462, GREM2, RNF20, TPD52L1, MTA1, CKS2, TRIP13, PIP5K1B, LMAN2L, PLCB1, IGF1, AZIN1, ZFP3, FOLR1, UBE2K, INTS7, HNRNPA0, MATN2, GPX7, HMGN3, ZMYND11, PAM, HMGCR, MBNL2, EIF4A2, RPL15, CCDC115, NRCAM, PIGX, GSTK1, PIP5KL1, TRA2B, EPHB3, PFKM, ZNF84, PGRMC1, SLC23A2, STXBP6, CLOCK, TRIB2, DROSHA, CXXC1, NFIA) | 0.230 (0.209, 0.250) | 0.450 (0.395, 0.505) | 0.185 | 0.504 | 0.551 (±0.0084) | 0.273 (±0.0014) | \| | \| |  |  |  |
| │ ├── CliXO:435 (POLR2H, ILF2, CASD1, SUMO3, SH3PXD2A, ALDH1B1, QPCT, GPER1, AMD1, TOP2B, CTSV, FAM172A, TSPAN3, NRIP1, FGF13, HEXB, CNPY4, CCND1, MAD2L2, FZD1, C4orf3, PUS7, FBN2, PJA1, IL33, PALLD, CNN3, DDR2, FBLIM1, AMOTL2, LHFPL2, PDGFC, DKK3, MID1, PKM, MME, INPP5F, PLBD1, PIP5K1B, INTS7, SLC23A2) | 0.770 (0.750, 0.791) | 0.450 (0.395, 0.505) | 0.535 | 0.504 | 0.447 (±0.0190) | 0.875 (±0.0007) | \| | \| |  |  |  |
| │ └── CliXO:413 (LHFPL2, PKM) | 0.770 (0.750, 0.791) | 0.450 (0.395, 0.505) | 0.535 | 0.504 | 0.436 (±0.0171) | 0.804 (±0.0006) | \| | \| |  |  |  |
| │ │ └── CliXO:427 (QPCT, AMD1, TOP2B, FAM172A, NRIP1, HEXB, CNPY4, CCND1, MAD2L2, FZD1, C4orf3, PJA1, IL33, PALLD, CNN3, DDR2, FBLIM1, AMOTL2, LHFPL2, PDGFC, MME, INPP5F) | 0.770 (0.750, 0.791) | 0.450 (0.395, 0.505) | 0.535 | 0.504 | 0.447 (±0.0190) | 0.876 (±0.0007) | \| | \| |  |  |  |
| │ │ └── CliXO:424 (QPCT, NRIP1, HEXB, CCND1, MAD2L2, FZD1, C4orf3, PJA1, IL33, CNN3, DDR2, FBLIM1, AMOTL2, PDGFC, MME) | 0.770 (0.750, 0.791) | 0.450 (0.395, 0.505) | 0.535 | 0.504 | 0.443 (±0.0186) | 0.861 (±0.0007) | \| | \| |  |  |  |
| │ ├── CliXO:443 (IFIT1, CASD1, DCXR, ZNF512B, COQ2, CTSF, SPON1, BRCA1, CHEK1, TSPAN3, TMEM106B, CDO1, SULF2, HLF, SH3BGRL, CLIC3, HSPA2, WFS1, SESN3, PIK3R1, TBC1D5, FGF13, TYRO3, RBM26, TAF9B, SMARCA2, HMGB3, TRIM24, CLCN5, RDX, PCYOX1, PUS7, DDX17, PPT1, PEBP1, TKFC, SRPK2, FBLN7, PALLD, GEMIN5, AMOTL2, MID1, ATXN10, HJURP, SHANK2, CCDC113, MELK, RAD51, HMMR, INPP5F, PLBD1, MX1, ZNF462, GREM2, RNF20, TPD52L1, MTA1, CKS2, TRIP13, PIP5K1B, PLCB1, IGF1, AZIN1, ZFP3, UBE2K, INTS7, HNRNPA0, MATN2, GPX7, HMGN3, PAM, MBNL2, EIF4A2, RPL15, CCDC115, NRCAM, TRA2B, ZNF84, PGRMC1, SLC23A2, STXBP6, CLOCK, TRIB2, DROSHA, NFIA) | 0.230 (0.209, 0.250) | 0.450 (0.395, 0.505) | 0.185 | 0.504 | 0.640 (±0.0073) | 0.456 (±0.0002) | \| | \| |  |  |  |
| │ │ └── CliXO:438 (CASD1, DCXR, COQ2, SPON1, BRCA1, CHEK1, TMEM106B, CDO1, SULF2, HLF, SH3BGRL, CLIC3, HSPA2, WFS1, SESN3, PIK3R1, FGF13, TYRO3, RBM26, TAF9B, SMARCA2, TRIM24, CLCN5, PCYOX1, DDX17, GEMIN5, HJURP, CCDC113, MELK, HMMR, PLBD1, MX1, ZNF462, RNF20, MTA1, CKS2, PIP5K1B, PLCB1, AZIN1, ZFP3, UBE2K, INTS7, HNRNPA0, MATN2, GPX7, HMGN3, PAM, MBNL2, EIF4A2, ZNF84, SLC23A2, STXBP6, TRIB2, DROSHA) | 0.770 (0.750, 0.791) | 0.450 (0.395, 0.505) | 0.535 | 0.504 | 0.446 (±0.0189) | 0.872 (±0.0007) | \| | \| |  |  |  |
| │ ├── CliXO:415 (COQ2, SPON1, CHEK1) | 0.770 (0.750, 0.791) | 0.450 (0.395, 0.505) | 0.535 | 0.504 | 0.438 (±0.0176) | 0.825 (±0.0006) | \| | \| |  |  |  |
| │ │ └── CliXO:423 (CASD1, TMEM106B, SH3BGRL, WFS1, SESN3, PIK3R1, FGF13, TYRO3, RBM26, SMARCA2, TRIM24, PCYOX1, PIP5K1B, ZFP3, PAM) | 0.770 (0.750, 0.791) | 0.450 (0.395, 0.505) | 0.535 | 0.504 | 0.444 (±0.0186) | 0.862 (±0.0007) | \| | \| |  |  |  |
| │ │ ├── CliXO:406 (SH3BGRL, FGF13) | 0.770 (0.750, 0.791) | 0.450 (0.395, 0.505) | 0.535 | 0.504 | 0.436 (±0.0170) | 0.802 (±0.0006) | \| | \| |  |  |  |
| │ │ └── CliXO:422 (CASD1, TMEM106B, SH3BGRL, WFS1, SESN3, PIK3R1, TYRO3, RBM26, SMARCA2, TRIM24) | 0.770 (0.750, 0.791) | 0.450 (0.395, 0.505) | 0.535 | 0.504 | 0.441 (±0.0183) | 0.849 (±0.0007) | \| | \| |  |  |  |
| │ └── CliXO:447 (IFIT1, MXRA5, FBXO9, SLC25A4, TMEM216, PPA1, POLR2H, ILF2, KDM1A, THRA, CASD1, FKBP4, THOP1, SUMO3, SH3PXD2A, ALDH1B1, DLX5, CIB2, COL1A2, GLA, HDAC1, MRI1, DCXR, KIF17, LSM6, GNG7, GPER1, AMD1, GLE1, ZNF512B, COQ2, COL6A1, PHKA2, CLIC5, LIN7A, CTSF, COPZ2, TOP2B, CTSV, LRRC41, SPON1, ASTE1, DONSON, TNFAIP8L1, FAM172A, BRCA1, CHEK1, TSPAN3, GRINA, KIAA0232, TMEM106B, ODF2, XPO4, CDO1, SULF2, DNMT3A, CALM1, MTPN, HLF, SH3BGRL, CLIC3, HSPA2, WFS1, NRIP1, PIK3R1, TBC1D5, FGF13, TYRO3, RBM26, TRIM25, HEXB, CDK20, OVGP1, TAF9B, SMARCA2, DNASE2, IGSF8, SUV39H1, CNPY4, TTLL5, OAS3, GOSR1, HMGB3, TRIM24, PPM1M, ST6GALNAC1, CCND1, MAD2L2, SLC39A8, FZD1, BAD, MOB3C, CLCN5, C4orf3, RDX, PCYOX1, PUS7, DDX17, FBN2, PPT1, PJA1, PEBP1, TKFC, CBX5, SRPK2, FBLN7, PALLD, CDC42BPA, DDR2, GEMIN5, PODXL, FBLIM1, AMOTL2, LHFPL2, PDGFC, DKK3, MID1, NIPAL3, PCCB, ATXN10, HJURP, SHANK2, PKM, BCAT2, CCDC113, UNC119, MME, MELK, RAD51, HMMR, COX8A, INPP5F, PLBD1, MX1, ZNF462, GREM2, RNF20, TPD52L1, MTA1, CKS2, TRIP13, PIP5K1B, LMAN2L, PLCB1, IGF1, AZIN1, ZFP3, FOLR1, UBE2K, INTS7, HNRNPA0, MATN2, GPX7, HMGN3, ZMYND11, PAM, HMGCR, MBNL2, EIF4A2, RPL15, CCDC115, NRCAM, PIGX, GSTK1, PIP5KL1, TRA2B, EPHB3, PFKM, ZNF84, PGRMC1, SLC23A2, STXBP6, CLOCK, TRIB2, DROSHA, CXXC1, NFIA) | 0.770 (0.750, 0.791) | 0.450 (0.395, 0.505) | 0.535 | 0.504 | 0.664 (±0.0145) | 0.992 (±0.0008) | \| | \| |  |  |  |
| │ ├── CliXO:434 (GLA, GLE1, LIN7A, LRRC41, TNFAIP8L1, KIAA0232, ODF2, DNMT3A, MTPN, HSPA2, CDK20, OVGP1, DNASE2, SUV39H1, TTLL5, OAS3, HMGB3, PPM1M, ST6GALNAC1, SLC39A8, MOB3C, DDX17, PEBP1, TKFC, SRPK2, CDC42BPA, PODXL, NIPAL3, PCCB, HJURP, RAD51, HMMR, MX1, TRIP13, LMAN2L, PLCB1, ZMYND11, HMGCR, RPL15, GSTK1) | 0.770 (0.750, 0.791) | 0.450 (0.395, 0.505) | 0.535 | 0.504 | 0.440 (±0.0180) | 0.839 (±0.0007) | \| | \| |  |  |  |
| │ │ └── CliXO:412 (PODXL, NIPAL3) | 0.770 (0.750, 0.791) | 0.450 (0.395, 0.505) | 0.535 | 0.504 | 0.436 (±0.0171) | 0.804 (±0.0006) | \| | \| |  |  |  |
| │ ├── CliXO:439 (MXRA5, FBXO9, POLR2H, ILF2, KDM1A, THOP1, SUMO3, SH3PXD2A, CIB2, COL1A2, HDAC1, KIF17, GNG7, GPER1, AMD1, COL6A1, COPZ2, TOP2B, CTSV, FAM172A, GRINA, KIAA0232, XPO4, HEXB, IGSF8, CNPY4, GOSR1, CCND1, MAD2L2, FZD1, BAD, CLCN5, C4orf3, PUS7, FBN2, PPT1, PJA1, CBX5, FBLN7, PALLD, DDR2, GEMIN5, FBLIM1, AMOTL2, LHFPL2, PDGFC, DKK3, MID1, PKM, UNC119, MME, COX8A, INPP5F, IGF1, INTS7, GPX7, MBNL2, NRCAM, PIP5KL1, TRA2B, EPHB3, PFKM, PGRMC1, SLC23A2, CLOCK, CXXC1, NFIA) | 0.230 (0.209, 0.250) | 0.450 (0.395, 0.505) | 0.185 | 0.504 | 0.641 (±0.0074) | 0.458 (±0.0002) | \| | \| |  |  |  |
| │ └── CliXO:413 (LHFPL2, PKM) | 0.770 (0.750, 0.791) | 0.450 (0.395, 0.505) | 0.535 | 0.504 | 0.436 (±0.0171) | 0.804 (±0.0006) | \| | \| |  |  |  |
| │ └── CliXO:446 (IFIT1, MXRA5, FBXO9, SLC25A4, TMEM216, PPA1, POLR2H, ILF2, KDM1A, THRA, CASD1, FKBP4, THOP1, SUMO3, SH3PXD2A, ALDH1B1, DLX5, CIB2, COL1A2, GLA, HDAC1, MRI1, DCXR, KIF17, LSM6, GNG7, GPER1, AMD1, GLE1, ZNF512B, COQ2, COL6A1, PHKA2, CLIC5, LIN7A, CTSF, COPZ2, TOP2B, CTSV, LRRC41, SPON1, ASTE1, DONSON, TNFAIP8L1, FAM172A, BRCA1, CHEK1, TSPAN3, GRINA, KIAA0232, TMEM106B, ODF2, XPO4, CDO1, SULF2, DNMT3A, CALM1, MTPN, HLF, SH3BGRL, CLIC3, HSPA2, WFS1, NRIP1, PIK3R1, TBC1D5, FGF13, TYRO3, RBM26, TRIM25, HEXB, CDK20, OVGP1, TAF9B, SMARCA2, DNASE2, IGSF8, SUV39H1, CNPY4, TTLL5, OAS3, GOSR1, HMGB3, TRIM24, PPM1M, ST6GALNAC1, CCND1, SLC39A8, MOB3C, CLCN5, C4orf3, RDX, PCYOX1, PUS7, DDX17, FBN2, PPT1, PJA1, PEBP1, TKFC, CBX5, SRPK2, FBLN7, PALLD, CDC42BPA, DDR2, GEMIN5, AMOTL2, LHFPL2, PDGFC, DKK3, MID1, NIPAL3, PCCB, ATXN10, HJURP, SHANK2, BCAT2, CCDC113, UNC119, MELK, RAD51, HMMR, COX8A, INPP5F, PLBD1, MX1, ZNF462, GREM2, RNF20, TPD52L1, MTA1, CKS2, TRIP13, PIP5K1B, LMAN2L, PLCB1, IGF1, AZIN1, ZFP3, FOLR1, UBE2K, INTS7, HNRNPA0, MATN2, GPX7, HMGN3, ZMYND11, PAM, HMGCR, MBNL2, EIF4A2, RPL15, CCDC115, NRCAM, PIGX, GSTK1, PIP5KL1, TRA2B, EPHB3, PFKM, ZNF84, PGRMC1, SLC23A2, STXBP6, CLOCK, TRIB2, DROSHA, CXXC1, NFIA) | 0.230 (0.209, 0.250) | 0.450 (0.395, 0.505) | 0.185 | 0.504 | 0.729 (±0.0105) | 0.409 (±0.0004) | \| | \| |  |  |  |
| │ │ ├── CliXO:406 (SH3BGRL, FGF13) | 0.770 (0.750, 0.791) | 0.450 (0.395, 0.505) | 0.535 | 0.504 | 0.436 (±0.0170) | 0.802 (±0.0006) | \| | \| |  |  |  |
| │ ├── CliXO:415 (COQ2, SPON1, CHEK1) | 0.770 (0.750, 0.791) | 0.450 (0.395, 0.505) | 0.535 | 0.504 | 0.438 (±0.0176) | 0.825 (±0.0006) | \| | \| |  |  |  |
| │ ├── CliXO:419 (CTSF, OVGP1, SLC39A8, RDX, CDC42BPA, NIPAL3, HMGCR, RPL15, GSTK1) | 0.770 (0.750, 0.791) | 0.450 (0.395, 0.505) | 0.535 | 0.504 | 0.441 (±0.0182) | 0.846 (±0.0007) | \| | \| |  |  |  |
| │ ├── CliXO:421 (FBXO9, SLC25A4, KIF17, LIN7A, LRRC41, GRINA, DNASE2, IGSF8, UNC119, PIP5KL1) | 0.770 (0.750, 0.791) | 0.450 (0.395, 0.505) | 0.535 | 0.504 | 0.441 (±0.0183) | 0.849 (±0.0007) | \| | \| |  |  |  |
| │ └── CliXO:445 (IFIT1, MXRA5, FBXO9, SLC25A4, TMEM216, PPA1, POLR2H, ILF2, KDM1A, FKBP4, THOP1, SUMO3, SH3PXD2A, ALDH1B1, CIB2, COL1A2, GLA, HDAC1, MRI1, DCXR, KIF17, LSM6, GNG7, GPER1, AMD1, GLE1, ZNF512B, COL6A1, PHKA2, CLIC5, LIN7A, CTSF, COPZ2, TOP2B, CTSV, LRRC41, ASTE1, DONSON, TNFAIP8L1, FAM172A, TSPAN3, GRINA, KIAA0232, ODF2, XPO4, CDO1, SULF2, DNMT3A, CALM1, MTPN, HSPA2, WFS1, TBC1D5, TYRO3, TRIM25, HEXB, CDK20, OVGP1, TAF9B, DNASE2, SUV39H1, CNPY4, TTLL5, OAS3, GOSR1, HMGB3, TRIM24, PPM1M, CCND1, SLC39A8, MOB3C, CLCN5, C4orf3, RDX, PUS7, DDX17, FBN2, PPT1, PJA1, PEBP1, TKFC, CBX5, SRPK2, FBLN7, PALLD, CDC42BPA, DDR2, GEMIN5, AMOTL2, LHFPL2, PDGFC, DKK3, MID1, PCCB, ATXN10, HJURP, SHANK2, BCAT2, CCDC113, UNC119, MELK, RAD51, HMMR, COX8A, INPP5F, PLBD1, MX1, ZNF462, GREM2, RNF20, TPD52L1, MTA1, CKS2, TRIP13, PIP5K1B, LMAN2L, PLCB1, IGF1, AZIN1, ZFP3, FOLR1, UBE2K, INTS7, HNRNPA0, MATN2, GPX7, HMGN3, ZMYND11, PAM, HMGCR, MBNL2, EIF4A2, RPL15, CCDC115, NRCAM, PIGX, GSTK1, PIP5KL1, TRA2B, EPHB3, PFKM, ZNF84, PGRMC1, SLC23A2, STXBP6, CLOCK, TRIB2, DROSHA, CXXC1, NFIA) | 0.230 (0.209, 0.250) | 0.450 (0.395, 0.505) | 0.185 | 0.504 | 0.593 (±0.0151) | 0.127 (±0.0052) | \| | \| |  |  |  |
| └── CliXO:453 (ABCA1, ANKRD13A, ZFAND5, PANX1, RASEF, LAPTM5, RNF11, ACKR2, TBXAS1, ETS2, ARRDC3, BHLHE40, MTMR6, ARHGEF12, STRN3, ELF4, RAPH1, ENG, EPS8L2, RGS16, GRHL1, MBD2, OXSR1, CTSA, NCEH1, VEGFA, AKAP13, TMEM154, LDLR, JUNB, YPEL2, CDYL2, TES, MAFF, CEBPD, GALE, SMIM3, SH3BP5, LYN, CSF2RB, TSC22D1, GULP1, CDKN1B, GADD45G, CORO2A, GPX3, NEK7, PRRG4, MINPP1, MXD1, SLC1A1, FAM43A, ZNF217, CHIC2, PPP2R5A, SLC2A1, PIK3CG, ZNF281, PIK3AP1, SLC38A6, TMEM176B, SNAPC1, SPTY2D1, HILPDA, STAB1, NR4A3, ZFYVE1, TIMP3, MAP3K8, CORO1A, SYNJ2, SLC38A1, RFK, PRKCH, RNF19A, ERP44, PPL, PGBD2, LATS2, HTRA1, LRRC8A, PAPPA, TREM1, KPNA1, TYROBP, NUCB2, LCP1, GBE1, PGK1, CPEB4, LURAP1L, EAF1, DUSP4, TSPAN12, RBM47, OPTN, ANKRD37, VDR, FAM210A, MXI1, DUSP1, SLC7A2, TEX2, PIK3CB, PLIN2, LPL, SAMD8, ITGA5, TSPAN13, KIAA0513, IGSF3, ST6GAL1, SPAG4, SH2B3, ANXA1, HEY1, TGFBR1, TANK, STAT4, LYPD3, WDSUB1, PREP, CX3CR1, TPP1, RNASE1, GNG4, SGMS2, TMEM30A, DDIT4, SEMA4B, PTPN12, DOCK8, KLF4, UBR1, EPS8L1, BCAR3, SPI1, TP53BP2, SWAP70, FLI1, C9orf72, TSPAN14, PDZD2, CTBS, TMCC3, EDN1, ZNF143, FNDC3A, TRAK2, MYO5A, GRAMD1A, SLCO2B1, RHOBTB3, DNAJB9, ARHGDIB, SARAF, NFIL3, CYFIP2, MED13L, WDFY3, SMPDL3A, EPS15, MEF2A, EFHD1, HNMT, AMPD3, CRISPLD2, CDK9, SLC9A9, C3AR1, FBXO8, USP3, PTBP3, COL17A1, FOSL2, ELL2, LRRC32, SLCO4A1, CRYAB, HPGDS, SLC31A2, LY86, UBALD2, TNFAIP8, RNF24, SLC17A5, ARHGAP28, RCBTB1, PROM1, SERPINB9, SLC16A3, MAOB, NPC1, NARF, SBF2, ELMO1, ASAP2, KLF3, LPXN, MYO1F, RIN2, ARHGAP24, MAP4K4, CYBB, OSMR, DPYD, SACM1L, PLOD2, SNAP23, STK17A, CASP4, ERO1A, EDEM1, RAB8B, CSF3R, FAM107B, DEGS1, MAML2, TOR1B, MICU3, CPD, PTPRJ, FRMD4A, CITED2) | 0.184 (0.163, 0.205) | 0.450 (0.395, 0.505) | 0.096 | 0.504 | 0.586 (±0.0117) | 0.182 (±0.0030) | \| | \| |  |  |  |
| ├── CliXO:428 (ANKRD13A, RASEF, ACKR2, TBXAS1, RGS16, OXSR1, GALE, CORO2A, PPP2R5A, ZFYVE1, RBM47, OPTN, IGSF3, TANK, SPI1, TSPAN14, TRAK2, AMPD3, UBALD2, SLC17A5, ASAP2, ERO1A, CSF3R, CPD) | 0.770 (0.750, 0.791) | 0.450 (0.395, 0.505) | 0.535 | 0.504 | 0.440 (±0.0180) | 0.839 (±0.0007) | \| | \| |  |  |  |
| │ └── CliXO:408 (RBM47, IGSF3) | 0.770 (0.750, 0.791) | 0.450 (0.395, 0.505) | 0.535 | 0.504 | 0.436 (±0.0171) | 0.804 (±0.0006) | \| | \| |  |  |  |
| ├── CliXO:433 (VEGFA, ZNF281, SLC38A6, SNAPC1, HILPDA, SYNJ2, PPL, TREM1, PGK1, DUSP4, ANKRD37, VDR, ITGA5, TSPAN13, SPAG4, ANXA1, HEY1, LYPD3, SGMS2, SEMA4B, BCAR3, TP53BP2, MYO5A, AMPD3, USP3, COL17A1, SLCO4A1, SLC31A2, RNF24, SLC17A5, SLC16A3, NPC1, MYO1F, ARHGAP24, OSMR, PLOD2, ERO1A) | 0.770 (0.750, 0.791) | 0.450 (0.395, 0.505) | 0.535 | 0.504 | 0.437 (±0.0170) | 0.801 (±0.0006) | \| | \| |  |  |  |
| │ ├── CliXO:414 (ITGA5, ANXA1) | 0.770 (0.750, 0.791) | 0.450 (0.395, 0.505) | 0.535 | 0.504 | 0.436 (±0.0171) | 0.804 (±0.0006) | \| | \| |  |  |  |
| │ ├── CliXO:418 (PGK1, SPAG4, HEY1, LYPD3, BCAR3, SLCO4A1) | 0.770 (0.750, 0.791) | 0.450 (0.395, 0.505) | 0.535 | 0.504 | 0.440 (±0.0180) | 0.839 (±0.0007) | \| | \| |  |  |  |
| │ ├── CliXO:410 (HEY1, LYPD3) | 0.770 (0.750, 0.791) | 0.450 (0.395, 0.505) | 0.535 | 0.504 | 0.436 (±0.0171) | 0.804 (±0.0006) | \| | \| |  |  |  |
| └── CliXO:452 (ABCA1, ANKRD13A, ZFAND5, PANX1, RASEF, LAPTM5, RNF11, ACKR2, TBXAS1, ETS2, ARRDC3, BHLHE40, MTMR6, ARHGEF12, STRN3, ELF4, RAPH1, ENG, EPS8L2, RGS16, GRHL1, MBD2, OXSR1, CTSA, NCEH1, VEGFA, AKAP13, TMEM154, LDLR, JUNB, YPEL2, CDYL2, TES, MAFF, CEBPD, GALE, SMIM3, SH3BP5, LYN, CSF2RB, TSC22D1, GULP1, CDKN1B, GADD45G, CORO2A, GPX3, NEK7, PRRG4, MINPP1, MXD1, SLC1A1, FAM43A, ZNF217, CHIC2, PPP2R5A, SLC2A1, PIK3CG, ZNF281, PIK3AP1, SLC38A6, TMEM176B, SNAPC1, SPTY2D1, HILPDA, STAB1, NR4A3, ZFYVE1, TIMP3, MAP3K8, CORO1A, SYNJ2, SLC38A1, RFK, PRKCH, RNF19A, ERP44, PPL, PGBD2, LATS2, HTRA1, LRRC8A, PAPPA, TREM1, KPNA1, TYROBP, NUCB2, LCP1, GBE1, PGK1, CPEB4, LURAP1L, EAF1, DUSP4, TSPAN12, OPTN, ANKRD37, VDR, FAM210A, MXI1, DUSP1, SLC7A2, TEX2, PIK3CB, PLIN2, LPL, SAMD8, ITGA5, TSPAN13, KIAA0513, IGSF3, ST6GAL1, SPAG4, SH2B3, ANXA1, TGFBR1, TANK, STAT4, LYPD3, WDSUB1, PREP, CX3CR1, TPP1, RNASE1, GNG4, SGMS2, TMEM30A, DDIT4, SEMA4B, PTPN12, DOCK8, KLF4, UBR1, EPS8L1, BCAR3, SPI1, TP53BP2, SWAP70, FLI1, C9orf72, TSPAN14, PDZD2, CTBS, TMCC3, EDN1, ZNF143, FNDC3A, TRAK2, MYO5A, GRAMD1A, SLCO2B1, RHOBTB3, DNAJB9, ARHGDIB, SARAF, NFIL3, CYFIP2, MED13L, WDFY3, SMPDL3A, EPS15, MEF2A, EFHD1, HNMT, AMPD3, CRISPLD2, CDK9, SLC9A9, C3AR1, FBXO8, USP3, PTBP3, COL17A1, FOSL2, ELL2, LRRC32, SLCO4A1, CRYAB, HPGDS, SLC31A2, LY86, UBALD2, TNFAIP8, RNF24, SLC17A5, ARHGAP28, RCBTB1, PROM1, SERPINB9, SLC16A3, MAOB, NPC1, NARF, SBF2, ELMO1, ASAP2, KLF3, LPXN, MYO1F, RIN2, ARHGAP24, MAP4K4, CYBB, OSMR, DPYD, SACM1L, PLOD2, SNAP23, STK17A, CASP4, ERO1A, EDEM1, RAB8B, CSF3R, FAM107B, DEGS1, MAML2, TOR1B, MICU3, CPD, PTPRJ, FRMD4A, CITED2) | 0.816 (0.795, 0.837) | 0.450 (0.395, 0.505) | 0.547 | 0.504 | 0.420 (±0.0118) | 0.751 (±0.0006) | \| | \| |  |  |  |
| ├── CliXO:444 (PANX1, RNF11, ARRDC3, BHLHE40, MTMR6, STRN3, NCEH1, VEGFA, JUNB, MAFF, SMIM3, CSF2RB, TSC22D1, GULP1, CDKN1B, NEK7, MINPP1, MXD1, FAM43A, ZNF217, SLC2A1, ZNF281, SLC38A6, TMEM176B, SNAPC1, SPTY2D1, HILPDA, NR4A3, MAP3K8, SYNJ2, RFK, PPL, LATS2, HTRA1, TREM1, LCP1, PGK1, EAF1, DUSP4, ANKRD37, VDR, MXI1, DUSP1, ITGA5, TSPAN13, SPAG4, SH2B3, ANXA1, STAT4, LYPD3, WDSUB1, PREP, GNG4, SGMS2, TMEM30A, DDIT4, SEMA4B, KLF4, UBR1, EPS8L1, BCAR3, TP53BP2, C9orf72, FNDC3A, MYO5A, RHOBTB3, DNAJB9, ARHGDIB, SARAF, CYFIP2, MED13L, WDFY3, MEF2A, EFHD1, HNMT, AMPD3, CRISPLD2, SLC9A9, USP3, COL17A1, ELL2, LRRC32, SLCO4A1, HPGDS, SLC31A2, TNFAIP8, RNF24, SLC17A5, ARHGAP28, RCBTB1, SERPINB9, SLC16A3, MAOB, NPC1, NARF, ELMO1, LPXN, MYO1F, ARHGAP24, MAP4K4, OSMR, SACM1L, PLOD2, STK17A, CASP4, ERO1A, MICU3) | 0.770 (0.750, 0.791) | 0.450 (0.395, 0.505) | 0.535 | 0.504 | 0.534 (±0.0126) | 0.273 (±0.0014) | \| | \| |  |  |  |
| │ ├── CliXO:409 (WDSUB1, MICU3) | 0.770 (0.750, 0.791) | 0.450 (0.395, 0.505) | 0.535 | 0.504 | 0.436 (±0.0171) | 0.804 (±0.0006) | \| | \| |  |  |  |
| │ ├── CliXO:414 (ITGA5, ANXA1) | 0.770 (0.750, 0.791) | 0.450 (0.395, 0.505) | 0.535 | 0.504 | 0.436 (±0.0171) | 0.804 (±0.0006) | \| | \| |  |  |  |
| │ ├── CliXO:417 (WDFY3, HNMT, ARHGAP28, RCBTB1, ELMO1, LPXN) | 0.770 (0.750, 0.791) | 0.450 (0.395, 0.505) | 0.535 | 0.504 | 0.440 (±0.0180) | 0.839 (±0.0007) | \| | \| |  |  |  |
| │ │ └── CliXO:405 (ARHGAP28, RCBTB1) | 0.770 (0.750, 0.791) | 0.450 (0.395, 0.505) | 0.535 | 0.504 | 0.436 (±0.0171) | 0.804 (±0.0006) | \| | \| |  |  |  |
| └── CliXO:451 (ABCA1, ANKRD13A, ZFAND5, PANX1, RASEF, LAPTM5, RNF11, ACKR2, TBXAS1, ETS2, ARRDC3, BHLHE40, MTMR6, ARHGEF12, STRN3, ELF4, RAPH1, ENG, EPS8L2, RGS16, GRHL1, MBD2, OXSR1, CTSA, NCEH1, VEGFA, AKAP13, TMEM154, LDLR, JUNB, YPEL2, CDYL2, TES, MAFF, CEBPD, GALE, SMIM3, SH3BP5, LYN, CSF2RB, TSC22D1, GULP1, CDKN1B, GADD45G, CORO2A, GPX3, NEK7, PRRG4, MINPP1, MXD1, SLC1A1, ZNF217, CHIC2, PPP2R5A, SLC2A1, PIK3CG, ZNF281, PIK3AP1, TMEM176B, SNAPC1, SPTY2D1, HILPDA, STAB1, NR4A3, ZFYVE1, TIMP3, MAP3K8, CORO1A, SYNJ2, SLC38A1, RFK, PRKCH, RNF19A, ERP44, PPL, PGBD2, LATS2, HTRA1, LRRC8A, PAPPA, TREM1, KPNA1, TYROBP, NUCB2, LCP1, GBE1, PGK1, CPEB4, LURAP1L, EAF1, DUSP4, TSPAN12, OPTN, ANKRD37, VDR, FAM210A, MXI1, DUSP1, SLC7A2, TEX2, PIK3CB, PLIN2, LPL, SAMD8, ITGA5, TSPAN13, KIAA0513, IGSF3, ST6GAL1, SPAG4, SH2B3, TGFBR1, TANK, STAT4, WDSUB1, PREP, CX3CR1, TPP1, RNASE1, GNG4, SGMS2, TMEM30A, DDIT4, SEMA4B, PTPN12, DOCK8, KLF4, UBR1, EPS8L1, BCAR3, SPI1, TP53BP2, SWAP70, FLI1, C9orf72, TSPAN14, PDZD2, CTBS, TMCC3, EDN1, ZNF143, FNDC3A, TRAK2, MYO5A, GRAMD1A, SLCO2B1, RHOBTB3, DNAJB9, ARHGDIB, SARAF, NFIL3, CYFIP2, MED13L, WDFY3, SMPDL3A, EPS15, MEF2A, EFHD1, HNMT, AMPD3, CRISPLD2, CDK9, SLC9A9, C3AR1, FBXO8, USP3, PTBP3, COL17A1, FOSL2, ELL2, LRRC32, SLCO4A1, CRYAB, HPGDS, SLC31A2, LY86, UBALD2, TNFAIP8, RNF24, SLC17A5, ARHGAP28, RCBTB1, PROM1, SERPINB9, SLC16A3, MAOB, NPC1, NARF, SBF2, ELMO1, ASAP2, KLF3, LPXN, MYO1F, RIN2, ARHGAP24, MAP4K4, CYBB, OSMR, DPYD, SACM1L, PLOD2, SNAP23, STK17A, CASP4, ERO1A, EDEM1, RAB8B, CSF3R, FAM107B, DEGS1, MAML2, TOR1B, CPD, PTPRJ, FRMD4A, CITED2) | 0.755 (0.735, 0.776) | 0.450 (0.395, 0.505) | 0.518 | 0.504 | 0.424 (±0.0152) | 0.822 (±0.0006) | \| | \| |  |  |  |
| │ ├── CliXO:417 (WDFY3, HNMT, ARHGAP28, RCBTB1, ELMO1, LPXN) | 0.770 (0.750, 0.791) | 0.450 (0.395, 0.505) | 0.535 | 0.504 | 0.440 (±0.0180) | 0.839 (±0.0007) | \| | \| |  |  |  |
| │ │ └── CliXO:405 (ARHGAP28, RCBTB1) | 0.770 (0.750, 0.791) | 0.450 (0.395, 0.505) | 0.535 | 0.504 | 0.436 (±0.0171) | 0.804 (±0.0006) | \| | \| |  |  |  |
| ├── CliXO:440 (ABCA1, ZFAND5, PANX1, LAPTM5, ACKR2, ETS2, ARRDC3, ARHGEF12, RGS16, GRHL1, OXSR1, NCEH1, AKAP13, YPEL2, TES, MAFF, SH3BP5, LYN, TSC22D1, GADD45G, MXD1, ZNF217, CHIC2, SLC2A1, PIK3AP1, SPTY2D1, MAP3K8, PRKCH, RNF19A, LATS2, HTRA1, LRRC8A, EAF1, MXI1, SLC7A2, TEX2, PLIN2, LPL, SAMD8, TSPAN13, ST6GAL1, TGFBR1, STAT4, TPP1, GNG4, DDIT4, SEMA4B, PTPN12, SWAP70, FLI1, FNDC3A, GRAMD1A, SARAF, NFIL3, EPS15, MEF2A, PTBP3, FOSL2, ELL2, LRRC32, CRYAB, UBALD2, RNF24, PROM1, NARF, ASAP2, RIN2, CYBB, CASP4, RAB8B, FAM107B, MAML2, TOR1B, FRMD4A) | 0.681 (0.652, 0.710) | 0.450 (0.395, 0.505) | 0.249 | 0.504 | 0.438 (±0.0164) | 0.797 (±0.0006) | \| | \| |  |  |  |
| │ └── CliXO:431 (ABCA1, ZFAND5, PANX1, ETS2, ARHGEF12, NCEH1, TES, SH3BP5, TSC22D1, SLC2A1, MAP3K8, RNF19A, HTRA1, LRRC8A, EAF1, MXI1, PLIN2, LPL, ST6GAL1, FLI1, GRAMD1A, SARAF, EPS15, PTBP3, ELL2, PROM1, ASAP2, RAB8B, FAM107B, MAML2, TOR1B) | 0.762 (0.739, 0.785) | 0.450 (0.395, 0.505) | 0.377 | 0.504 | 0.431 (±0.0157) | 0.817 (±0.0006) | \| | \| |  |  | CliXO:449 |
| └── CliXO:450 (ABCA1, ANKRD13A, ZFAND5, PANX1, RASEF, LAPTM5, RNF11, ACKR2, TBXAS1, ETS2, ARRDC3, BHLHE40, MTMR6, ARHGEF12, STRN3, ELF4, RAPH1, ENG, EPS8L2, RGS16, GRHL1, MBD2, OXSR1, CTSA, NCEH1, VEGFA, AKAP13, TMEM154, LDLR, JUNB, YPEL2, CDYL2, TES, MAFF, CEBPD, GALE, SMIM3, SH3BP5, LYN, CSF2RB, TSC22D1, GULP1, CDKN1B, GADD45G, CORO2A, GPX3, NEK7, PRRG4, MINPP1, MXD1, SLC1A1, ZNF217, CHIC2, PPP2R5A, SLC2A1, PIK3CG, ZNF281, PIK3AP1, TMEM176B, SNAPC1, SPTY2D1, HILPDA, STAB1, NR4A3, ZFYVE1, TIMP3, MAP3K8, CORO1A, SYNJ2, SLC38A1, RFK, PRKCH, RNF19A, ERP44, PPL, PGBD2, LATS2, HTRA1, LRRC8A, TREM1, TYROBP, LCP1, GBE1, PGK1, CPEB4, LURAP1L, EAF1, DUSP4, TSPAN12, OPTN, ANKRD37, VDR, FAM210A, MXI1, DUSP1, SLC7A2, TEX2, PIK3CB, PLIN2, SAMD8, ITGA5, TSPAN13, IGSF3, ST6GAL1, SPAG4, SH2B3, TGFBR1, TANK, STAT4, WDSUB1, PREP, CX3CR1, TPP1, GNG4, SGMS2, TMEM30A, DDIT4, SEMA4B, PTPN12, DOCK8, KLF4, UBR1, EPS8L1, BCAR3, SPI1, TP53BP2, SWAP70, FLI1, C9orf72, TSPAN14, PDZD2, CTBS, TMCC3, EDN1, ZNF143, FNDC3A, TRAK2, MYO5A, GRAMD1A, SLCO2B1, RHOBTB3, DNAJB9, ARHGDIB, SARAF, NFIL3, CYFIP2, MED13L, SMPDL3A, EPS15, MEF2A, EFHD1, AMPD3, CRISPLD2, CDK9, SLC9A9, C3AR1, FBXO8, USP3, PTBP3, COL17A1, FOSL2, ELL2, LRRC32, SLCO4A1, CRYAB, HPGDS, SLC31A2, LY86, UBALD2, TNFAIP8, RNF24, SLC17A5, RCBTB1, PROM1, SERPINB9, SLC16A3, MAOB, NPC1, NARF, SBF2, ELMO1, ASAP2, KLF3, LPXN, MYO1F, RIN2, ARHGAP24, MAP4K4, CYBB, OSMR, DPYD, SACM1L, PLOD2, SNAP23, STK17A, CASP4, ERO1A, EDEM1, RAB8B, CSF3R, FAM107B, DEGS1, MAML2, TOR1B, CPD, PTPRJ, FRMD4A, CITED2) | 0.751 (0.724, 0.778) | 0.446 (0.392, 0.500) | 0.622 | 0.004 | 0.500 (±0.0079) | 0.347 (±0.0008) | \| | \| |  |  |  |
| ├── CliXO:426 (CORO1A, STAT4, PREP, GNG4, RHOBTB3, ARHGDIB, SARAF, CYFIP2, MEF2A, EFHD1, SLC9A9, HPGDS, LY86, RCBTB1, MAOB, ELMO1, LPXN) | 0.770 (0.750, 0.791) | 0.450 (0.395, 0.505) | 0.535 | 0.504 | 0.444 (±0.0187) | 0.864 (±0.0007) | \| | \| |  |  |  |
| ├── CliXO:429 (ANKRD13A, RASEF, TBXAS1, RAPH1, ENG, RGS16, GRHL1, CTSA, OPTN, IGSF3, EPS8L1, SPI1, TSPAN14, AMPD3, PTBP3, COL17A1, SLC31A2, UBALD2, RNF24, SLC17A5, SLC16A3, ASAP2, MAP4K4, PLOD2, CASP4, ERO1A, CPD, PTPRJ) | 0.725 (0.702, 0.748) | 0.450 (0.395, 0.505) | 0.422 | 0.504 | 0.431 (±0.0155) | 0.816 (±0.0006) | \| | \| |  |  | CliXO:449 |
| └── CliXO:449 (ABCA1, ANKRD13A, ZFAND5, PANX1, RASEF, LAPTM5, RNF11, ACKR2, TBXAS1, ETS2, ARRDC3, BHLHE40, MTMR6, ARHGEF12, STRN3, ELF4, RAPH1, ENG, EPS8L2, RGS16, GRHL1, MBD2, OXSR1, CTSA, NCEH1, VEGFA, AKAP13, TMEM154, LDLR†, JUNB, YPEL2, CDYL2, TES, MAFF, CEBPD, GALE, SMIM3, SH3BP5, LYN, CSF2RB, TSC22D1, GULP1, CDKN1B, GADD45G, CORO2A, GPX3, NEK7, PRRG4, MINPP1, MXD1, SLC1A1, ZNF217, CHIC2, SLC2A1, PIK3CG, ZNF281, PIK3AP1, TMEM176B, SNAPC1, SPTY2D1, HILPDA, STAB1, NR4A3, TIMP3, MAP3K8, CORO1A, SYNJ2, SLC38A1, RFK, PRKCH, RNF19A, ERP44, PPL†, LATS2, HTRA1, LRRC8A, TREM1, TYROBP, LCP1, GBE1, PGK1, CPEB4, EAF1, DUSP4, TSPAN12, OPTN, ANKRD37, VDR, FAM210A, MXI1, DUSP1, SLC7A2, TEX2, PIK3CB, PLIN2, SAMD8, TSPAN13, ST6GAL1, SPAG4, SH2B3, TGFBR1, STAT4, WDSUB1, PREP, CX3CR1, TPP1, GNG4, SGMS2, TMEM30A, DDIT4, SEMA4B, PTPN12, DOCK8, KLF4, UBR1, EPS8L1, BCAR3, SPI1, TP53BP2, SWAP70, FLI1, C9orf72, TSPAN14, PDZD2, CTBS, TMCC3, EDN1, ZNF143, FNDC3A, MYO5A, GRAMD1A, SLCO2B1, RHOBTB3, DNAJB9, ARHGDIB, SARAF, NFIL3, CYFIP2, MED13L, SMPDL3A, EPS15, MEF2A, EFHD1, AMPD3, CRISPLD2, CDK9, SLC9A9, C3AR1, FBXO8, USP3, PTBP3†, COL17A1, FOSL2, ELL2, LRRC32†, SLCO4A1, CRYAB, HPGDS, SLC31A2, LY86, UBALD2, TNFAIP8, RNF24, SLC17A5, PROM1, SERPINB9, MAOB, NPC1, NARF, SBF2, ELMO1, ASAP2, KLF3, LPXN, MYO1F, RIN2, ARHGAP24, MAP4K4, CYBB, OSMR, DPYD, SACM1L, PLOD2, SNAP23†, STK17A, CASP4, ERO1A, EDEM1, RAB8B, CSF3R, FAM107B, DEGS1, MAML2, TOR1B, CPD, PTPRJ, FRMD4A, CITED2) | 0.291 (0.266, 0.315) | 0.480 (0.422, 0.538) | 0.131 | 0.504 | 0.596 (±0.0049) | 0.367 (±0.0006) | \|✓ | \|✓ | ✓ |  | CliXO:449 |

The Reported column indicates the ontology from Tables 1–2 to which this row’s eligible genes belong. An ontology is reported if it passes all criteria (C1–C4) and its eligible gene set is not a strict subset of another passing ontology’s gene set. The column is empty when the ontology has no eligible genes or does not pass all criteria. ✓, the ontology passed the criterion; †, the gene had a grad-CAM value >97.5th percentile in the same ontology; |, separator between PPV/TPR and NPV/TNR results in C1 and C2 columns; AUC-PRC, area under the precision-recall curve; AUC-ROC, area under the receiver operating characteristic curve; C1, lower bounds finite and >0; C2, absolute difference ≤25% between development and test sets; C3, at least 1 eligible gene; C4, lower bound of AUC-ROC >0.5 in both sets; CI, confidence interval; Dev, development set; grad-CAM, gradient-weighted class activation mapping; HELLP, hemolysis, elevated liver enzyme, and low platelet; PE, preeclampsia; RMSE, root mean square error.

Table S13. Threshold-specific confusion matrices for the early-onset, severe PE pathogenesis model connected with first-trimester placenta.

| Ontology | PPV/TPR (Dev) | PPV/TPR (Test) | NPV/TNR (Dev) | NPV/TNR (Test) | C1 | C2 | C3 | C4 | Reported |
| --- | --- | --- | --- | --- | --- | --- | --- | --- | --- |
| root (CX3CR1, SLIT3, CEBPD, MYO5A, SLC2A1, GNG11, DNAJC1, AKAP13, SCARB1, ZBTB20, PTPN12, IGF1, KIAA0232, CBX5, DYRK2, ETS2, MEF2A, GPER1, ZNF462, IFI44L, CYTL1, GSN, PARM1, NFKB1, RAB3B, SEMA3G, ST6GAL1, MCTP2, SYK, NFIA, TXNDC11, SMIM3, LRRC8A, JAK2, CDK14, MS4A4A, LAPTM5, JUNB, F8, RIN2, CLIC5, CARD16, PRKCE, KIF5B, CCDC59, TBXAS1, DDIT4, GNB1, EXOC6, CCND1, TMTC4, SAV1, NECTIN4, PPP2R5A, KMT2E, FZD1, CYBB, DPYD, ELMO1, SNAPC1, C3AR1, TSPAN33, HMGN3, MIDN, LTF, RIMKLA, TRIP10, CLOCK, TACC1, FLI1, MBTPS2, MXI1, RNASE1, MAML2, SYPL1, AMOTL2, DDR2, ACTN1, TDP1, MIIP, TANK, ARRDC3, YPEL2, GALNT18, TYROBP, DUSP1, ZNF281, LAMA4, EFHD1, EDEM1, SBF2, TEX2, THOP1, PPP3CA, TLR7, THRA, LRRC32, HSPA2, PIK3AP1, TSPAN14, ZFYVE1, MRI1, CHIC2, MLLT1, COL6A1, SLC9A9, MINPP1, DIO2, SULF2, ASTE1, PLCB1, RNF11, CMKLR1, ZNF331, PODXL, SDC4, WDSUB1, HMOX1, PDGFC, SMPD2, SLC26A6, PGRMC1, SLC6A8, GUCA2A, TLR4, BCL2L11, MXRA5, MAP3K9, BCORL1, HK1, RCBTB2, ASAP2, RALGPS1, ADCY7, TMCC3, ANGPT1, ARL6IP5, RASAL2, ARTN, RASEF, ANKDD1A, AVL9, SYDE1, MATN2, ILF2, GCLM, CTBS, GOLM1, PROM1, PM20D2, EBF3, SH2B3, ATP10D, IL33, ERRFI1, HNMT, DKK3, ELL2, GRINA, PDE5A, MAP4K4, SESN3, NMNAT2, BRAF, INSL4, NIPAL3, TRIB2, ARID3A, KDM1A, TMEM106B, VEGFA, CNNM4, WDR11, MT1F, CREBL2, PKM, CDO1, NUDT5, HEY1, PEBP1, SLC38A1, LHFPL2, NCEH1, SEMA4C, ZMYND11, PLOD2, TRPC6, FANCE, VEGFC, PMAIP1, SHANK2, PFKFB4, ACVRL1, SLC35B3, GALK1, GBP4, EDN1, NUCB2, DHX29, ADARB1, OLFML2B, CPOX, PAPSS1, GPR107, HMGCR, MIOS, ACSF2, DLX5, CCDC113, GAN, TMEM176B, FCN3, ARHGAP24, CDS1, KIFC3, XPO4, LRCH1, PCYOX1, INTS7, AZIN1, SPG21, APLN, WNK2, G3BP2, FGF13, SLCO2B1, LIMCH1, AGTR1, FBN2, SARAF, CNOT6L, TRIP13, YTHDC2, ZNF512B, TMEM141, TAPT1, SACM1L, INSIG1, RNF20, BRCA1, ERLIN2, CADM3, AMD1, GLA, PUS7, CKS2, ROBO4, AK2, PRKAB2, BTD, HMMR, PLBD1, LEPROTL1, WDFY2, FLVCR2, PHKA2, PDP2, DCXR, SLC38A6, SLC39A8, NPL, SLC11A1, CTSA, PTPRJ, C1orf21, MME, DUSP4, MXD1, KLHL3, RCAN3, APH1B, FOLR1, DUS2, SLC44A3, TMEM43, PIK3CB, ABLIM3, GRHL1, DAB2, LDLR, SIPA1L1, SLC24A3, MAT2A, MKNK1, SLC16A3, GRHL3, EPS8L1, HGF, TP53BP2, LSM1, MOB3C, NHLRC2, CDYL2, KIF2A, MAN1A2, PAPPA, NPEPPS, INPP5F, NEK7, TLCD1, RASSF8, TMEM268, CSNK2A2, CCSAP, KIF17, ZNF75A, KLHL42, PKD2, NIN, PPP2R1B, HSPA9, GGCX, CYP19A1, GPT2, SH3RF1, EZH2, TKFC, LURAP1L, CHEK1, SPON1, SCMH1, PLAG1, STX6, EPB41L5, DONSON, IDE, PDZD2, GEMIN5, PRKCH, RAD51, MAFF, GPD1L, TOP2B, PREP, ARHGAP26, FNDC3A, STAT5B, CLTC, GADD45G, ODF2, VCP, RHOBTB3, TMEM154, DOCK8, MELK, SMARCA2, TTI1, MTMR4, ETNK2, ARHGAP28, ESRP1, HJURP, UBE2H) | 44.4% (42.2, 46.6) / 100.0% (100.0, 100.0) | 0.9% (0.7, 1.2) / — | — / 0.0% (0.0, 0.0) | — / 0.0% (0.0, 0.0) | \| | \| |  |  |  |
| CliXO:418 (CX3CR1, SLIT3, CEBPD, MYO5A, SLC2A1, GNG11, DNAJC1, AKAP13, SCARB1, ZBTB20, PTPN12, IGF1, KIAA0232, CBX5, DYRK2, ETS2, MEF2A, GPER1, ZNF462, IFI44L, CYTL1, GSN, PARM1, NFKB1, RAB3B, SEMA3G, ST6GAL1, MCTP2, SYK, NFIA, TXNDC11, SMIM3, LRRC8A, JAK2, CDK14, MS4A4A, LAPTM5, JUNB, F8, RIN2, CLIC5, CARD16, PRKCE, KIF5B, CCDC59, TBXAS1, DDIT4, GNB1, EXOC6, CCND1, TMTC4, SAV1, NECTIN4, PPP2R5A, KMT2E, FZD1, CYBB, DPYD, ELMO1, SNAPC1, C3AR1, TSPAN33, HMGN3, MIDN, LTF, RIMKLA, TRIP10, CLOCK, TACC1, FLI1, MBTPS2, MXI1, RNASE1, MAML2, SYPL1, AMOTL2, DDR2, ACTN1, TDP1, MIIP, TANK, ARRDC3, YPEL2, GALNT18, TYROBP, DUSP1, ZNF281, LAMA4, EFHD1, EDEM1, SBF2, TEX2, THOP1, PPP3CA, TLR7, THRA, LRRC32, HSPA2, PIK3AP1, TSPAN14, ZFYVE1, MRI1, CHIC2, MLLT1, COL6A1, SLC9A9, MINPP1, DIO2, SULF2, ASTE1, PLCB1, RNF11, CMKLR1, ZNF331, PODXL, SDC4, WDSUB1, HMOX1, PDGFC, SMPD2, SLC26A6, PGRMC1, SLC6A8, GUCA2A, TLR4, BCL2L11, MXRA5, MAP3K9, BCORL1, HK1, RCBTB2, ASAP2, RALGPS1, ADCY7, TMCC3, ANGPT1, ARL6IP5, RASAL2, ARTN, RASEF, ANKDD1A, AVL9, SYDE1, MATN2, ILF2, GCLM, CTBS, GOLM1, PROM1, PM20D2, EBF3, SH2B3, ATP10D, IL33, ERRFI1, HNMT, DKK3, ELL2, GRINA, PDE5A, MAP4K4, SESN3, NMNAT2, BRAF, INSL4, NIPAL3, TRIB2, ARID3A, KDM1A, TMEM106B, VEGFA, CNNM4, WDR11, MT1F, CREBL2, PKM, CDO1, NUDT5, HEY1, PEBP1, SLC38A1, LHFPL2, NCEH1, SEMA4C, ZMYND11, PLOD2, TRPC6, FANCE, VEGFC, PMAIP1, SHANK2, PFKFB4, ACVRL1, SLC35B3, GALK1, GBP4, EDN1, NUCB2, DHX29, ADARB1, OLFML2B, CPOX, PAPSS1, GPR107, HMGCR, MIOS, ACSF2, DLX5, CCDC113, GAN, TMEM176B, FCN3, ARHGAP24, CDS1, KIFC3, XPO4, LRCH1, PCYOX1, INTS7, AZIN1, SPG21, APLN, WNK2, G3BP2, FGF13, SLCO2B1, LIMCH1, AGTR1, FBN2, SARAF, CNOT6L, TRIP13, YTHDC2, ZNF512B, TMEM141, TAPT1, SACM1L, INSIG1, RNF20, BRCA1, ERLIN2, CADM3, AMD1, GLA, PUS7, CKS2, ROBO4, AK2, PRKAB2, BTD, HMMR, PLBD1, LEPROTL1, WDFY2, FLVCR2, PHKA2, PDP2, DCXR, SLC38A6, SLC39A8, NPL, SLC11A1, CTSA, PTPRJ, C1orf21, MME, DUSP4, MXD1, KLHL3, RCAN3, APH1B, FOLR1, DUS2, SLC44A3, TMEM43, PIK3CB, ABLIM3, GRHL1, DAB2, LDLR, SIPA1L1, SLC24A3, MAT2A, MKNK1, SLC16A3, GRHL3, EPS8L1, HGF, TP53BP2, LSM1, MOB3C, NHLRC2, CDYL2, KIF2A, MAN1A2, PAPPA, NPEPPS, INPP5F, NEK7, TLCD1, RASSF8, TMEM268, CSNK2A2, CCSAP, KIF17, ZNF75A, KLHL42, PKD2, NIN, PPP2R1B, HSPA9, GGCX, CYP19A1, GPT2, SH3RF1, EZH2, TKFC, LURAP1L, CHEK1, SPON1, SCMH1, PLAG1, STX6, EPB41L5, DONSON, IDE, PDZD2, GEMIN5, PRKCH, RAD51, MAFF, GPD1L, TOP2B, PREP, ARHGAP26, FNDC3A, STAT5B, CLTC, GADD45G, ODF2, VCP, RHOBTB3, TMEM154, DOCK8, MELK, SMARCA2, TTI1, MTMR4, ETNK2, ARHGAP28, ESRP1, HJURP, UBE2H) | 44.4% (42.2, 46.6) / 100.0% (100.0, 100.0) | 0.9% (0.7, 1.2) / — | — / 0.0% (0.0, 0.0) | — / 0.0% (0.0, 0.0) | \| | \| |  |  |  |
| ├── CliXO:410 (SLC2A1, SMIM3, TMTC4, NECTIN4, HMGN3, ACTN1, MIIP, ARRDC3, GALNT18, EFHD1, PPP3CA, COL6A1, DIO2, RNF11, SDC4, WDSUB1, HMOX1, SMPD2, PGRMC1, SLC6A8, BCL2L11, MAP3K9, HK1, RALGPS1, ADCY7, RASEF, AVL9, ILF2, GCLM, GOLM1, PM20D2, ATP10D, ERRFI1, HNMT, ELL2, GRINA, SESN3, NMNAT2, BRAF, INSL4, NIPAL3†, ARID3A, KDM1A, TMEM106B, VEGFA, CNNM4, MT1F, PKM, CDO1, NUDT5, PEBP1, LHFPL2, NCEH1, SEMA4C, PLOD2, FANCE, PMAIP1, SHANK2, PFKFB4, GALK1, NUCB2, DHX29, CPOX, PAPSS1, GPR107, HMGCR, MIOS, ACSF2, DLX5, CCDC113, GAN, CDS1, PCYOX1, AZIN1, SPG21, G3BP2, FBN2, TRIP13, ZNF512B, TMEM141, SACM1L, BRCA1, ERLIN2, GLA, CKS2, AK2, PRKAB2, HMMR, PLBD1, FLVCR2, PHKA2, DCXR, SLC38A6, SLC39A8, NPL, SLC11A1, CTSA, C1orf21, MME, KLHL3, RCAN3, APH1B, FOLR1, DUS2, SLC44A3, PIK3CB, GRHL1, LDLR, SIPA1L1, SLC24A3, MAT2A, MKNK1, SLC16A3, GRHL3, HGF, TP53BP2, CDYL2, NPEPPS, INPP5F, TLCD1, RASSF8, TMEM268, PPP2R1B, HSPA9, GGCX, GPT2†, SH3RF1, TKFC, CHEK1, SPON1, SCMH1, PLAG1, STX6, EPB41L5, DONSON, IDE, GEMIN5, RAD51, GPD1L, TOP2B, PREP, CLTC, ODF2, VCP, TMEM154, DOCK8, MELK, TTI1, ETNK2, ARHGAP28, ESRP1, HJURP, UBE2H†) | 67.3% (64.4, 70.2) / 74.8% (71.5, 78.1) | 0.0% (0.0, 0.0) / — | 78.1% (75.3, 80.8) / 70.9% (68.0, 73.7) | 98.9% (98.6, 99.2) / 87.0% (86.2, 87.7) | \|✓ | \|✓ | ✓ |  | CliXO:410 |
| │ ├── CliXO:354 (DIO2, PGRMC1) | 44.4% (42.2, 46.6) / 100.0% (100.0, 100.0) | 0.9% (0.7, 1.2) / — | — / 0.0% (0.0, 0.0) | — / 0.0% (0.0, 0.0) | \| | \| |  |  |  |
| │ ├── CliXO:360 (PEBP1, LDLR) | 44.4% (42.2, 46.6) / 100.0% (100.0, 100.0) | 0.9% (0.7, 1.2) / — | — / 0.0% (0.0, 0.0) | — / 0.0% (0.0, 0.0) | \| | \| |  |  |  |
| │ ├── CliXO:362 (SLC2A1, NECTIN4) | 44.4% (42.2, 46.6) / 100.0% (100.0, 100.0) | 0.9% (0.7, 1.2) / — | — / 0.0% (0.0, 0.0) | — / 0.0% (0.0, 0.0) | \| | \| |  |  |  |
| │ ├── CliXO:364 (MIIP, BCL2L11, PMAIP1) | 44.4% (42.2, 46.6) / 100.0% (100.0, 100.0) | 0.9% (0.7, 1.2) / — | — / 0.0% (0.0, 0.0) | — / 0.0% (0.0, 0.0) | \| | \| |  |  |  |
| │ ├── CliXO:367 (NIPAL3, MT1F, PAPSS1) | 44.4% (42.2, 46.6) / 100.0% (100.0, 100.0) | 0.9% (0.7, 1.2) / — | — / 0.0% (0.0, 0.0) | — / 0.0% (0.0, 0.0) | \| | \| |  |  | CliXO:410 |
| │ ├── CliXO:368 (FANCE, SLC16A3, INPP5F, RASSF8) | 44.4% (42.2, 46.6) / 100.0% (100.0, 100.0) | 0.9% (0.7, 1.2) / — | — / 0.0% (0.0, 0.0) | — / 0.0% (0.0, 0.0) | \| | \| |  |  |  |
| │ ├── CliXO:374 (HK1, NIPAL3, VEGFA, PKM, GALK1) | 44.4% (42.2, 46.6) / 100.0% (100.0, 100.0) | 0.9% (0.7, 1.2) / — | — / 0.0% (0.0, 0.0) | — / 0.0% (0.0, 0.0) | \| | \| |  |  |  |
| │ │ └── CliXO:358 (VEGFA, GALK1) | 44.4% (42.2, 46.6) / 100.0% (100.0, 100.0) | 0.9% (0.7, 1.2) / — | — / 0.0% (0.0, 0.0) | — / 0.0% (0.0, 0.0) | \| | \| |  |  |  |
| │ ├── CliXO:378 (GCLM, NMNAT2, TMEM106B, LHFPL2, PMAIP1, CDS1, RCAN3) | 44.4% (42.2, 46.6) / 100.0% (100.0, 100.0) | 0.9% (0.7, 1.2) / — | — / 0.0% (0.0, 0.0) | — / 0.0% (0.0, 0.0) | \| | \| |  |  |  |
| │ │ └── CliXO:359 (CDS1, RCAN3) | 44.4% (42.2, 46.6) / 100.0% (100.0, 100.0) | 0.9% (0.7, 1.2) / — | — / 0.0% (0.0, 0.0) | — / 0.0% (0.0, 0.0) | \| | \| |  |  |  |
| │ ├── CliXO:379 (HMGN3, PPP3CA, COL6A1, ZNF512B, CKS2, SPON1, EPB41L5) | 44.4% (42.2, 46.6) / 100.0% (100.0, 100.0) | 0.9% (0.7, 1.2) / — | — / 0.0% (0.0, 0.0) | — / 0.0% (0.0, 0.0) | \| | \| |  |  |  |
| │ │ └── CliXO:357 (COL6A1, CKS2) | 44.4% (42.2, 46.6) / 100.0% (100.0, 100.0) | 0.9% (0.7, 1.2) / — | — / 0.0% (0.0, 0.0) | — / 0.0% (0.0, 0.0) | \| | \| |  |  |  |
| │ ├── CliXO:380 (PEBP1, HMGCR, TRIP13, ERLIN2, C1orf21, SLC24A3, PPP2R1B, GPD1L) | 44.4% (42.2, 46.6) / 100.0% (100.0, 100.0) | 0.9% (0.7, 1.2) / — | — / 0.0% (0.0, 0.0) | — / 0.0% (0.0, 0.0) | \| | \| |  |  |  |
| │ ├── CliXO:382 (ACSF2, TRIP13, ZNF512B, DCXR, MME, HGF, NPEPPS, STX6, EPB41L5) | 44.4% (42.2, 46.6) / 100.0% (100.0, 100.0) | 0.9% (0.7, 1.2) / — | — / 0.0% (0.0, 0.0) | — / 0.0% (0.0, 0.0) | \| | \| |  |  |  |
| │ ├── CliXO:390 (FANCE, SHANK2, TRIP13, HMMR, PLBD1, PHKA2, DCXR, PIK3CB, GRHL1, SIPA1L1, SLC16A3, TP53BP2, INPP5F, TLCD1, PPP2R1B, GPT2, CHEK1, PLAG1, RAD51, TOP2B, MELK, ESRP1, HJURP) | 44.4% (42.2, 46.6) / 100.0% (100.0, 100.0) | 0.9% (0.7, 1.2) / — | — / 0.0% (0.0, 0.0) | — / 0.0% (0.0, 0.0) | \| | \| |  |  |  |
| │ │ └── CliXO:366 (TRIP13, HMMR, MELK) | 44.4% (42.2, 46.6) / 100.0% (100.0, 100.0) | 0.9% (0.7, 1.2) / — | — / 0.0% (0.0, 0.0) | — / 0.0% (0.0, 0.0) | \| | \| |  |  |  |
| │ ├── CliXO:391 (TMTC4, HMGN3, ACTN1, MIIP, PPP3CA, SDC4, SLC6A8, HK1, RASEF, GOLM1, HNMT, ARID3A, MT1F, PLOD2, PFKFB4, HMGCR, ACSF2, DLX5, PCYOX1, ERLIN2, CKS2, C1orf21, MME, SLC44A3, SLC24A3, IDE) | 44.4% (42.2, 46.6) / 100.0% (100.0, 100.0) | 0.9% (0.7, 1.2) / — | — / 0.0% (0.0, 0.0) | — / 0.0% (0.0, 0.0) | \| | \| |  |  |  |
| │ │ ├── CliXO:363 (C1orf21, SLC44A3, SLC24A3) | 44.4% (42.2, 46.6) / 100.0% (100.0, 100.0) | 0.9% (0.7, 1.2) / — | — / 0.0% (0.0, 0.0) | — / 0.0% (0.0, 0.0) | \| | \| |  |  |  |
| │ │ ├── CliXO:370 (TMTC4, RASEF, GOLM1, MT1F) | 44.4% (42.2, 46.6) / 100.0% (100.0, 100.0) | 0.9% (0.7, 1.2) / — | — / 0.0% (0.0, 0.0) | — / 0.0% (0.0, 0.0) | \| | \| |  |  |  |
| │ │ ├── CliXO:373 (TMTC4, GOLM1, HNMT, MT1F, PCYOX1) | 44.4% (42.2, 46.6) / 100.0% (100.0, 100.0) | 0.9% (0.7, 1.2) / — | — / 0.0% (0.0, 0.0) | — / 0.0% (0.0, 0.0) | \| | \| |  |  |  |
| │ │ └── CliXO:385 (HMGN3, ACTN1, MIIP, PPP3CA, SDC4, SLC6A8, HK1, ARID3A, PLOD2, PFKFB4, ACSF2, CKS2, MME) | 44.4% (42.2, 46.6) / 100.0% (100.0, 100.0) | 0.9% (0.7, 1.2) / — | — / 0.0% (0.0, 0.0) | — / 0.0% (0.0, 0.0) | \| | \| |  |  |  |
| │ └── CliXO:393 (TMTC4, WDSUB1, SMPD2, RASEF, GOLM1, HNMT, GRINA, SESN3, NIPAL3, TMEM106B, VEGFA, MT1F, NCEH1, NUCB2, DHX29, HMGCR, DLX5, CCDC113, PCYOX1, G3BP2, TMEM141, SACM1L, ERLIN2, CTSA, C1orf21, SLC44A3, LDLR, SLC24A3, TLCD1, HSPA9, GGCX, GPT2, IDE, PREP, VCP, TMEM154, DOCK8) | 44.4% (42.2, 46.6) / 100.0% (100.0, 100.0) | 0.9% (0.7, 1.2) / — | — / 0.0% (0.0, 0.0) | — / 0.0% (0.0, 0.0) | \| | \| |  |  |  |
| │ └── CliXO:383 (RASEF, GOLM1, HNMT, PCYOX1, G3BP2, CTSA, C1orf21, SLC44A3, SLC24A3, IDE, DOCK8) | 44.4% (42.2, 46.6) / 100.0% (100.0, 100.0) | 0.9% (0.7, 1.2) / — | — / 0.0% (0.0, 0.0) | — / 0.0% (0.0, 0.0) | \| | \| |  |  |  |
| │ └── CliXO:381 (RASEF, GOLM1, HNMT, G3BP2, CTSA, C1orf21, SLC44A3, SLC24A3, IDE) | 44.4% (42.2, 46.6) / 100.0% (100.0, 100.0) | 0.9% (0.7, 1.2) / — | — / 0.0% (0.0, 0.0) | — / 0.0% (0.0, 0.0) | \| | \| |  |  |  |
| │ └── CliXO:365 (CTSA, SLC44A3, SLC24A3) | 44.4% (42.2, 46.6) / 100.0% (100.0, 100.0) | 0.9% (0.7, 1.2) / — | — / 0.0% (0.0, 0.0) | — / 0.0% (0.0, 0.0) | \| | \| |  |  |  |
| └── CliXO:417 (CX3CR1, SLIT3, CEBPD, MYO5A, SLC2A1, GNG11, DNAJC1, AKAP13, SCARB1, ZBTB20†, PTPN12, IGF1, KIAA0232†, CBX5, DYRK2, ETS2, MEF2A, GPER1, ZNF462, IFI44L, CYTL1, GSN, PARM1, NFKB1, RAB3B, SEMA3G, ST6GAL1, MCTP2, SYK, NFIA, TXNDC11, SMIM3, LRRC8A, JAK2, CDK14, MS4A4A, LAPTM5, JUNB, F8, RIN2, CLIC5, CARD16, PRKCE, KIF5B, CCDC59, TBXAS1, DDIT4, GNB1, EXOC6, CCND1, TMTC4, SAV1, NECTIN4, PPP2R5A, KMT2E, FZD1, CYBB, DPYD, ELMO1, SNAPC1, C3AR1, TSPAN33, HMGN3, MIDN, LTF, RIMKLA, TRIP10, CLOCK, TACC1, FLI1, MBTPS2, MXI1, RNASE1, MAML2, SYPL1, AMOTL2, DDR2, ACTN1, TDP1, MIIP, TANK, ARRDC3, YPEL2, GALNT18, TYROBP, DUSP1, ZNF281, LAMA4, EFHD1, EDEM1, SBF2, TEX2, THOP1, PPP3CA, TLR7, THRA, LRRC32, HSPA2, PIK3AP1, TSPAN14, ZFYVE1, MRI1, CHIC2, MLLT1, COL6A1, SLC9A9, MINPP1, DIO2, SULF2, ASTE1, PLCB1, RNF11, CMKLR1, ZNF331, PODXL, SDC4, WDSUB1, PDGFC, SMPD2, SLC26A6, PGRMC1, SLC6A8, GUCA2A, TLR4, MXRA5, MAP3K9, BCORL1, HK1, RCBTB2, ASAP2, RALGPS1, ADCY7, TMCC3, ANGPT1, ARL6IP5, RASAL2, ARTN, RASEF, ANKDD1A, AVL9, SYDE1, MATN2, ILF2, GCLM, CTBS, GOLM1, PROM1, PM20D2, EBF3, SH2B3, ATP10D, IL33, ERRFI1, HNMT, DKK3†, ELL2, GRINA, PDE5A†, MAP4K4, SESN3, NMNAT2, BRAF, INSL4, NIPAL3†, TRIB2, ARID3A, KDM1A, TMEM106B, VEGFA, CNNM4, WDR11, MT1F†, CREBL2, PKM, CDO1, NUDT5, HEY1, PEBP1, SLC38A1, LHFPL2, NCEH1, SEMA4C, ZMYND11, PLOD2, TRPC6, FANCE, VEGFC, PMAIP1, SHANK2, PFKFB4, ACVRL1, SLC35B3, GALK1, GBP4, EDN1, NUCB2, DHX29, ADARB1, OLFML2B, CPOX, PAPSS1, GPR107, HMGCR, MIOS, ACSF2, DLX5, CCDC113, GAN, TMEM176B, FCN3, ARHGAP24, CDS1, KIFC3, XPO4, LRCH1, PCYOX1, INTS7, AZIN1, SPG21, APLN, WNK2, G3BP2, FGF13†, SLCO2B1, LIMCH1, AGTR1, FBN2, SARAF, CNOT6L, TRIP13, YTHDC2, ZNF512B, TMEM141, TAPT1, SACM1L, INSIG1, RNF20, BRCA1, ERLIN2, CADM3, AMD1, GLA, PUS7, CKS2, ROBO4, AK2, PRKAB2, BTD, HMMR, PLBD1, LEPROTL1, WDFY2, FLVCR2, PHKA2, PDP2, DCXR, SLC38A6, SLC39A8, NPL, SLC11A1, PTPRJ, C1orf21, MME, DUSP4, MXD1, KLHL3, RCAN3, APH1B, FOLR1, DUS2, SLC44A3, TMEM43, PIK3CB, ABLIM3, GRHL1, DAB2, LDLR, SIPA1L1, SLC24A3, MAT2A, MKNK1, SLC16A3, GRHL3, EPS8L1, HGF, TP53BP2, LSM1, MOB3C, NHLRC2, CDYL2, KIF2A, MAN1A2, PAPPA, NPEPPS, INPP5F, NEK7, TLCD1, RASSF8, TMEM268, CSNK2A2, CCSAP, KIF17, ZNF75A, KLHL42, PKD2, NIN†, PPP2R1B, HSPA9, GGCX, CYP19A1, GPT2†, SH3RF1, EZH2, TKFC, LURAP1L, CHEK1, SPON1, SCMH1, PLAG1, STX6, EPB41L5, DONSON, IDE, PDZD2, GEMIN5, PRKCH, RAD51, MAFF, GPD1L, TOP2B, PREP, ARHGAP26, FNDC3A, STAT5B, CLTC, GADD45G, ODF2, VCP, RHOBTB3, TMEM154, DOCK8, MELK, SMARCA2, TTI1, MTMR4, ETNK2, ARHGAP28, ESRP1, HJURP, UBE2H) | 67.3% (64.4, 70.2) / 74.8% (71.5, 78.1) | 0.0% (0.0, 0.0) / — | 78.1% (75.3, 80.8) / 70.9% (68.0, 73.7) | 98.9% (98.6, 99.2) / 87.0% (86.2, 87.7) | \|✓ | \|✓ | ✓ |  | CliXO:417 |
| ├── CliXO:412 (MYO5A, DNAJC1, CBX5, ZNF462, GSN, TXNDC11, JUNB, CCDC59, GNB1, TMTC4, KMT2E, SNAPC1, HMGN3, MXI1, AMOTL2, ACTN1, TDP1, MIIP, TYROBP, EDEM1, THOP1, PPP3CA, COL6A1, SLC9A9, DIO2, SDC4, WDSUB1, SMPD2, PGRMC1, SLC6A8, MAP3K9, HK1, RALGPS1, ADCY7, RASEF, ANKDD1A, AVL9, ILF2, GCLM, CTBS, GOLM1, PM20D2, ATP10D, ERRFI1, HNMT, GRINA, SESN3, NMNAT2, BRAF, NIPAL3, ARID3A, KDM1A, TMEM106B, VEGFA, CNNM4, WDR11, MT1F, PKM, CDO1, NUDT5, PEBP1, SLC38A1, LHFPL2, NCEH1, SEMA4C, PLOD2, FANCE, PMAIP1, SHANK2, PFKFB4, GALK1, CPOX, PAPSS1, GPR107, HMGCR, ACSF2, DLX5, CDS1, XPO4, PCYOX1, AZIN1, WNK2, G3BP2, FBN2, TRIP13, YTHDC2, ZNF512B, TMEM141, SACM1L, BRCA1, ERLIN2, GLA, PUS7, CKS2, AK2, PRKAB2, HMMR, PLBD1, WDFY2, FLVCR2, PHKA2, PDP2, DCXR, SLC38A6, SLC39A8, NPL, SLC11A1, C1orf21, MME, KLHL3, RCAN3, APH1B, FOLR1, DUS2, SLC44A3, PIK3CB, GRHL1, DAB2, LDLR, SIPA1L1, SLC24A3, MAT2A, MKNK1, SLC16A3, GRHL3, HGF, TP53BP2, LSM1, NHLRC2, NPEPPS, INPP5F, TLCD1, RASSF8, TMEM268, CSNK2A2, ZNF75A, KLHL42, PPP2R1B, HSPA9, GGCX, GPT2, SH3RF1, EZH2, TKFC, CHEK1, SPON1, SCMH1, PLAG1, STX6, EPB41L5, IDE, GEMIN5, RAD51, GPD1L, TOP2B, PREP, CLTC, ODF2, VCP, TMEM154, DOCK8, MELK, SMARCA2, TTI1, ETNK2, ARHGAP28, ESRP1, HJURP, UBE2H) | 44.4% (42.2, 46.6) / 100.0% (100.0, 100.0) | 0.9% (0.7, 1.2) / — | — / 0.0% (0.0, 0.0) | — / 0.0% (0.0, 0.0) | \| | \| |  |  |  |
| │ ├── CliXO:396 (PEBP1, SEMA4C, SHANK2, PFKFB4, HMGCR, ACSF2, XPO4, TRIP13, YTHDC2, BRCA1, ERLIN2, HMMR, PLBD1, WDFY2, PHKA2, DCXR, C1orf21, MME, APH1B, PIK3CB, SIPA1L1, SLC24A3, MAT2A, SLC16A3, HGF, TP53BP2, LSM1, NPEPPS, INPP5F, TLCD1, ZNF75A, KLHL42, PPP2R1B, GPT2, EZH2, CHEK1, SPON1, STX6, EPB41L5, IDE, RAD51, GPD1L, TOP2B, CLTC, ODF2, VCP, MELK, SMARCA2, TTI1, ETNK2, ARHGAP28, ESRP1, HJURP, UBE2H) | 44.4% (42.2, 46.6) / 100.0% (100.0, 100.0) | 0.9% (0.7, 1.2) / — | — / 0.0% (0.0, 0.0) | — / 0.0% (0.0, 0.0) | \| | \| |  |  |  |
| │ │ └── CliXO:389 (PHKA2, HGF, LSM1, ZNF75A, KLHL42, PPP2R1B, EZH2, CHEK1, SPON1, EPB41L5, RAD51, GPD1L, TOP2B, CLTC, ODF2, VCP, MELK, SMARCA2, ETNK2, ARHGAP28, ESRP1, UBE2H) | 44.4% (42.2, 46.6) / 100.0% (100.0, 100.0) | 0.9% (0.7, 1.2) / — | — / 0.0% (0.0, 0.0) | — / 0.0% (0.0, 0.0) | \| | \| |  |  |  |
| │ └── CliXO:405 (GSN, TMTC4, HMGN3, MXI1, ACTN1, MIIP, PPP3CA, COL6A1, DIO2, SDC4, SMPD2, PGRMC1, SLC6A8, HK1, RALGPS1, RASEF, ANKDD1A, GCLM, GOLM1, PM20D2, ATP10D, HNMT, GRINA, SESN3, NMNAT2, BRAF, NIPAL3, ARID3A, KDM1A, TMEM106B, VEGFA, CNNM4, MT1F, PKM, NUDT5, PEBP1, LHFPL2, NCEH1, SEMA4C, PLOD2, FANCE, PMAIP1, SHANK2, PFKFB4, GALK1, PAPSS1, GPR107, HMGCR, ACSF2, DLX5, CDS1, PCYOX1, AZIN1, G3BP2, FBN2, TRIP13, ZNF512B, TMEM141, ERLIN2, PUS7, CKS2, PRKAB2, HMMR, PLBD1, PHKA2, DCXR, SLC38A6, SLC39A8, NPL, C1orf21, MME, KLHL3, RCAN3, FOLR1, DUS2, SLC44A3, PIK3CB, LDLR, SIPA1L1, SLC24A3, MAT2A, MKNK1, SLC16A3, GRHL3, HGF, TP53BP2, NHLRC2, NPEPPS, INPP5F, RASSF8, TMEM268, PPP2R1B, GGCX, GPT2, SH3RF1, TKFC, SPON1, SCMH1, PLAG1, STX6, EPB41L5, IDE, GEMIN5, RAD51, GPD1L, TOP2B, PREP, CLTC, ODF2, VCP, TMEM154, DOCK8, TTI1, ETNK2, ARHGAP28, ESRP1, HJURP) | 44.4% (42.2, 46.6) / 100.0% (100.0, 100.0) | 0.9% (0.7, 1.2) / — | — / 0.0% (0.0, 0.0) | — / 0.0% (0.0, 0.0) | \| | \| |  |  | CliXO:417 |
| │ └── CliXO:395 (TMTC4, HMGN3, ACTN1, MIIP, PPP3CA, COL6A1, SDC4, PGRMC1, SLC6A8, HK1, RASEF, GOLM1, HNMT, NIPAL3, ARID3A, VEGFA, MT1F, PKM, NUDT5, SEMA4C, PLOD2, PFKFB4, GALK1, PAPSS1, HMGCR, ACSF2, DLX5, PCYOX1, G3BP2, ZNF512B, ERLIN2, PUS7, CKS2, DCXR, C1orf21, MME, FOLR1, SLC44A3, SLC24A3, GRHL3, NPEPPS, SPON1, SCMH1, EPB41L5, IDE, DOCK8) | 67.3% (64.4, 70.2) / 74.8% (71.5, 78.1) | 0.0% (0.0, 0.0) / — | 78.1% (75.3, 80.8) / 70.9% (68.0, 73.7) | 98.9% (98.6, 99.2) / 87.0% (86.2, 87.7) | \|✓ | \|✓ |  |  | CliXO:410 |
| │ ├── CliXO:376 (TMTC4, HMGN3, ACTN1, PPP3CA, ARID3A, PUS7) | 44.4% (42.2, 46.6) / 100.0% (100.0, 100.0) | 0.9% (0.7, 1.2) / — | — / 0.0% (0.0, 0.0) | — / 0.0% (0.0, 0.0) | \| | \| |  |  |  |
| └── CliXO:416 (CX3CR1, SLIT3, CEBPD, MYO5A, SLC2A1, GNG11, DNAJC1, AKAP13, SCARB1, ZBTB20, PTPN12, IGF1, KIAA0232, CBX5, DYRK2, ETS2, MEF2A, GPER1, ZNF462, IFI44L, CYTL1, GSN, PARM1, NFKB1, RAB3B, SEMA3G, ST6GAL1, MCTP2, SYK, NFIA, TXNDC11, SMIM3, LRRC8A, JAK2, CDK14, MS4A4A, LAPTM5, JUNB, F8, RIN2, CLIC5, CARD16, PRKCE, KIF5B, CCDC59, TBXAS1, DDIT4, GNB1, EXOC6, CCND1, TMTC4, SAV1, NECTIN4, PPP2R5A, KMT2E, FZD1, CYBB, DPYD, ELMO1, SNAPC1, C3AR1, TSPAN33, HMGN3, MIDN, LTF, RIMKLA, TRIP10, CLOCK, TACC1, FLI1, MBTPS2, MXI1, RNASE1, MAML2, SYPL1, AMOTL2, DDR2, ACTN1, TDP1, TANK, ARRDC3, YPEL2, GALNT18, TYROBP, DUSP1, ZNF281, LAMA4, EFHD1, EDEM1, SBF2, TEX2, THOP1, PPP3CA, TLR7, THRA, LRRC32, HSPA2, PIK3AP1, TSPAN14, ZFYVE1, MRI1, CHIC2, MLLT1, SLC9A9, MINPP1, DIO2, SULF2, ASTE1, PLCB1, RNF11, CMKLR1, ZNF331, PODXL, SDC4, WDSUB1, PDGFC, SMPD2, SLC26A6, PGRMC1, SLC6A8, GUCA2A, TLR4, MXRA5, MAP3K9, BCORL1, HK1, RCBTB2, ASAP2, RALGPS1, ADCY7, TMCC3, ANGPT1, ARL6IP5, RASAL2, ARTN, ANKDD1A, AVL9, SYDE1, MATN2, ILF2, GCLM, CTBS, GOLM1, PROM1, PM20D2, EBF3, SH2B3, ATP10D, IL33, ERRFI1, HNMT, DKK3, ELL2, GRINA, PDE5A, MAP4K4, SESN3, NMNAT2, BRAF, INSL4, NIPAL3, TRIB2, ARID3A, KDM1A, TMEM106B, VEGFA, CNNM4, WDR11, MT1F, CREBL2, PKM, CDO1, NUDT5, HEY1, PEBP1, SLC38A1, LHFPL2, NCEH1, SEMA4C, ZMYND11, PLOD2, TRPC6, FANCE, VEGFC, SHANK2, PFKFB4, ACVRL1, SLC35B3, GBP4, EDN1, NUCB2, DHX29, ADARB1, OLFML2B, CPOX, PAPSS1, GPR107, HMGCR, MIOS, ACSF2, DLX5, CCDC113, GAN, TMEM176B, FCN3, ARHGAP24, CDS1, KIFC3, XPO4, LRCH1, PCYOX1, INTS7, AZIN1, SPG21, APLN, WNK2, G3BP2, FGF13, SLCO2B1, LIMCH1, AGTR1, FBN2, SARAF, CNOT6L, TRIP13, YTHDC2, ZNF512B, TMEM141, TAPT1, SACM1L, INSIG1, RNF20, BRCA1, ERLIN2, CADM3, AMD1, GLA, PUS7, CKS2, ROBO4, AK2, PRKAB2, BTD, HMMR, PLBD1, LEPROTL1, WDFY2, FLVCR2, PHKA2, PDP2, DCXR, SLC38A6, SLC39A8, NPL, SLC11A1, PTPRJ, C1orf21, MME, DUSP4, MXD1, KLHL3, RCAN3, APH1B, DUS2, TMEM43, PIK3CB, ABLIM3, GRHL1, DAB2, LDLR, SIPA1L1, SLC24A3, MAT2A, MKNK1, SLC16A3, GRHL3, EPS8L1, HGF, TP53BP2, LSM1, MOB3C, NHLRC2, CDYL2, KIF2A, MAN1A2, PAPPA, NPEPPS, INPP5F, NEK7, TLCD1, TMEM268, CSNK2A2, CCSAP, KIF17, ZNF75A, KLHL42, PKD2, NIN, PPP2R1B, HSPA9, GGCX, CYP19A1, GPT2, SH3RF1, EZH2, TKFC, LURAP1L, CHEK1, SPON1, SCMH1, PLAG1, STX6, EPB41L5, DONSON, IDE, PDZD2, GEMIN5, PRKCH, RAD51, MAFF, GPD1L, TOP2B, PREP, ARHGAP26, FNDC3A, STAT5B, CLTC, GADD45G, ODF2, VCP, RHOBTB3, TMEM154, DOCK8, MELK, SMARCA2, TTI1, MTMR4, ETNK2, ARHGAP28, ESRP1, HJURP, UBE2H) | 44.4% (42.2, 46.6) / 100.0% (100.0, 100.0) | 0.9% (0.7, 1.2) / — | — / 0.0% (0.0, 0.0) | — / 0.0% (0.0, 0.0) | \| | \| |  |  |  |
| ├── CliXO:407 (CX3CR1, SLIT3, CEBPD, MYO5A, SLC2A1, GNG11, DNAJC1, AKAP13, SCARB1, IGF1, CBX5, DYRK2, MEF2A, GPER1, ZNF462, IFI44L, CYTL1, GSN, NFKB1, RAB3B, SEMA3G, MCTP2, NFIA, TXNDC11, SMIM3, CDK14, JUNB, RIN2, CLIC5, KIF5B, CCDC59, TBXAS1, DDIT4, GNB1, EXOC6, CCND1, SAV1, NECTIN4, KMT2E, FZD1, SNAPC1, TSPAN33, MIDN, LTF, RIMKLA, TRIP10, TACC1, FLI1, MXI1, RNASE1, MAML2, SYPL1, AMOTL2, TDP1, TANK, YPEL2, GALNT18, TYROBP, DUSP1, ZNF281, LAMA4, EFHD1, SBF2, TEX2, TLR7, THRA, LRRC32, HSPA2, PIK3AP1, TSPAN14, ZFYVE1, MRI1, CHIC2, MLLT1, SLC9A9, ASTE1, PLCB1, CMKLR1, ZNF331, PODXL, GUCA2A, MXRA5, BCORL1, RCBTB2, ASAP2, TMCC3, ANGPT1, ARL6IP5, RASAL2, ARTN, SYDE1, MATN2, PROM1, EBF3, SH2B3, IL33, ERRFI1, DKK3, PDE5A, MAP4K4, TRIB2, HEY1, ZMYND11, TRPC6, ACVRL1, SLC35B3, GBP4, EDN1, ADARB1, OLFML2B, TMEM176B, FCN3, ARHGAP24, KIFC3, LRCH1, APLN, FGF13, LIMCH1, AGTR1, SARAF, CNOT6L, TAPT1, INSIG1, RNF20, AMD1, ROBO4, LEPROTL1, MXD1, TMEM43, ABLIM3, EPS8L1, MOB3C, KIF2A, CCSAP, KIF17, LURAP1L, ARHGAP26, MTMR4) | 44.4% (42.2, 46.6) / 100.0% (100.0, 100.0) | 0.9% (0.7, 1.2) / — | — / 0.0% (0.0, 0.0) | — / 0.0% (0.0, 0.0) | \| | \| |  |  |  |
| │ ├── CliXO:356 (TMCC3, ADARB1) | 44.4% (42.2, 46.6) / 100.0% (100.0, 100.0) | 0.9% (0.7, 1.2) / — | — / 0.0% (0.0, 0.0) | — / 0.0% (0.0, 0.0) | \| | \| |  |  |  |
| │ ├── CliXO:372 (SLC2A1, ZNF331, ANGPT1, SYDE1, ARHGAP24) | 44.4% (42.2, 46.6) / 100.0% (100.0, 100.0) | 0.9% (0.7, 1.2) / — | — / 0.0% (0.0, 0.0) | — / 0.0% (0.0, 0.0) | \| | \| |  |  |  |
| │ ├── CliXO:384 (SLC2A1, SCARB1, SMIM3, NECTIN4, SYPL1, EFHD1, PIK3AP1, ARTN, ERRFI1, KIF2A, CCSAP) | 44.4% (42.2, 46.6) / 100.0% (100.0, 100.0) | 0.9% (0.7, 1.2) / — | — / 0.0% (0.0, 0.0) | — / 0.0% (0.0, 0.0) | \| | \| |  |  |  |
| │ │ └── CliXO:369 (SMIM3, SYPL1, EFHD1, ERRFI1) | 44.4% (42.2, 46.6) / 100.0% (100.0, 100.0) | 0.9% (0.7, 1.2) / — | — / 0.0% (0.0, 0.0) | — / 0.0% (0.0, 0.0) | \| | \| |  |  |  |
| │ ├── CliXO:387 (CYTL1, SEMA3G, GNB1, YPEL2, SBF2, LRRC32, HSPA2, MXRA5, ANGPT1, EBF3, PDE5A, ACVRL1, ARHGAP24, KIFC3) | 44.4% (42.2, 46.6) / 100.0% (100.0, 100.0) | 0.9% (0.7, 1.2) / — | — / 0.0% (0.0, 0.0) | — / 0.0% (0.0, 0.0) | \| | \| |  |  |  |
| │ └── CliXO:401 (CX3CR1, SLIT3, CEBPD, MYO5A, GNG11, DNAJC1, CBX5, DYRK2, GPER1, ZNF462, CYTL1, NFKB1, SEMA3G, NFIA, TXNDC11, CDK14, JUNB, RIN2, KIF5B, CCDC59, TBXAS1, GNB1, CCND1, KMT2E, FZD1, SNAPC1, TSPAN33, MIDN, LTF, RIMKLA, TACC1, FLI1, MXI1, AMOTL2, TDP1, YPEL2, GALNT18, TYROBP, DUSP1, ZNF281, TEX2, THRA, LRRC32, HSPA2, ZFYVE1, MRI1, CHIC2, MLLT1, PLCB1, GUCA2A, MXRA5, RCBTB2, ASAP2, ANGPT1, ARL6IP5, RASAL2, MATN2, PROM1, EBF3, SH2B3, IL33, DKK3, PDE5A, MAP4K4, TRIB2, HEY1, ZMYND11, TRPC6, GBP4, EDN1, OLFML2B, TMEM176B, ARHGAP24, KIFC3, LRCH1, FGF13, AGTR1, CNOT6L, TAPT1, ROBO4, ABLIM3, LURAP1L) | 44.4% (42.2, 46.6) / 100.0% (100.0, 100.0) | 0.9% (0.7, 1.2) / — | — / 0.0% (0.0, 0.0) | — / 0.0% (0.0, 0.0) | \| | \| |  |  |  |
| │ └── CliXO:392 (GNG11, CYTL1, SEMA3G, TXNDC11, JUNB, TBXAS1, CCND1, FZD1, LTF, TACC1, FLI1, MXI1, YPEL2, DUSP1, TEX2, THRA, ZFYVE1, CHIC2, PLCB1, GUCA2A, RCBTB2, ARL6IP5, PROM1, SH2B3, IL33, DKK3, MAP4K4, ZMYND11, GBP4, EDN1, OLFML2B, TMEM176B, ROBO4, LURAP1L) | 44.4% (42.2, 46.6) / 100.0% (100.0, 100.0) | 0.9% (0.7, 1.2) / — | — / 0.0% (0.0, 0.0) | — / 0.0% (0.0, 0.0) | \| | \| |  |  |  |
| │ └── CliXO:371 (CYTL1, FZD1, TACC1, THRA) | 44.4% (42.2, 46.6) / 100.0% (100.0, 100.0) | 0.9% (0.7, 1.2) / — | — / 0.0% (0.0, 0.0) | — / 0.0% (0.0, 0.0) | \| | \| |  |  |  |
| ├── CliXO:409 (TMTC4, ARRDC3, EDEM1, THOP1, WDSUB1, SMPD2, SLC26A6, MAP3K9, RALGPS1, ADCY7, ANKDD1A, AVL9, ILF2, GCLM, CTBS, GOLM1, PM20D2, ATP10D, ELL2, GRINA, SESN3, NMNAT2, BRAF, INSL4, TMEM106B, CNNM4, WDR11, CDO1, NUDT5, PEBP1, SLC38A1, LHFPL2, NCEH1, FANCE, SHANK2, NUCB2, DHX29, CPOX, GPR107, HMGCR, MIOS, CCDC113, GAN, CDS1, XPO4, PCYOX1, INTS7, AZIN1, SPG21, WNK2, G3BP2, FBN2, TRIP13, YTHDC2, ZNF512B, TMEM141, SACM1L, BRCA1, ERLIN2, GLA, AK2, PRKAB2, HMMR, PLBD1, WDFY2, FLVCR2, PHKA2, PDP2, DCXR, SLC38A6, SLC39A8, NPL, SLC11A1, PTPRJ, C1orf21, MME, DUSP4, KLHL3, RCAN3, APH1B, DUS2, PIK3CB, GRHL1, DAB2, LDLR, SIPA1L1, SLC24A3, MAT2A, MKNK1, SLC16A3, GRHL3, HGF, TP53BP2, LSM1, NHLRC2, MAN1A2, PAPPA, NPEPPS, INPP5F, NEK7, TLCD1, TMEM268, CSNK2A2, KIF17, ZNF75A, KLHL42, PKD2, NIN, PPP2R1B, HSPA9, GGCX, CYP19A1, GPT2, SH3RF1, EZH2, TKFC, CHEK1, SPON1, SCMH1, PLAG1, STX6, EPB41L5, DONSON, IDE, PDZD2, GEMIN5, RAD51, MAFF, GPD1L, TOP2B, PREP, ARHGAP26, FNDC3A, STAT5B, CLTC, GADD45G, ODF2, VCP, RHOBTB3, TMEM154, DOCK8, MELK, SMARCA2, ETNK2, ARHGAP28, ESRP1, HJURP, UBE2H) | 44.4% (42.2, 46.6) / 100.0% (100.0, 100.0) | 0.9% (0.7, 1.2) / — | — / 0.0% (0.0, 0.0) | — / 0.0% (0.0, 0.0) | \| | \| |  |  |  |
| │ ├── CliXO:394 (ARRDC3, WDSUB1, SMPD2, ANKDD1A, AVL9, GCLM, CTBS, ELL2, GRINA, SESN3, NMNAT2, INSL4, TMEM106B, CNNM4, WDR11, CDO1, LHFPL2, NCEH1, NUCB2, DHX29, GPR107, CCDC113, CDS1, AZIN1, SPG21, G3BP2, AK2, PLBD1, SLC38A6, SLC39A8, NPL, APH1B, SIPA1L1, LSM1, TLCD1, TMEM268, CSNK2A2, HSPA9, GPT2, IDE, GPD1L, CLTC, VCP, DOCK8) | 44.4% (42.2, 46.6) / 100.0% (100.0, 100.0) | 0.9% (0.7, 1.2) / — | — / 0.0% (0.0, 0.0) | — / 0.0% (0.0, 0.0) | \| | \| |  |  |  |
| │ ├── CliXO:403 (ARRDC3, EDEM1, THOP1, WDSUB1, SMPD2, SLC26A6, MAP3K9, RALGPS1, ADCY7, ANKDD1A, AVL9, ILF2, GCLM, CTBS, PM20D2, ATP10D, ELL2, GRINA, SESN3, BRAF, INSL4, TMEM106B, CNNM4, WDR11, CDO1, SLC38A1, LHFPL2, NCEH1, SHANK2, NUCB2, DHX29, CPOX, GPR107, MIOS, CCDC113, GAN, CDS1, XPO4, INTS7, AZIN1, SPG21, WNK2, G3BP2, FBN2, TRIP13, YTHDC2, TMEM141, BRCA1, AK2, PRKAB2, HMMR, PLBD1, WDFY2, FLVCR2, PHKA2, PDP2, SLC38A6, SLC39A8, NPL, SLC11A1, APH1B, DUS2, PIK3CB, GRHL1, SIPA1L1, MAT2A, MKNK1, TP53BP2, LSM1, NHLRC2, INPP5F, TLCD1, TMEM268, CSNK2A2, ZNF75A, KLHL42, PKD2, PPP2R1B, HSPA9, GGCX, GPT2, EZH2, CHEK1, PLAG1, DONSON, IDE, RAD51, GPD1L, TOP2B, CLTC, ODF2, VCP, DOCK8, MELK, SMARCA2, ETNK2, ARHGAP28, ESRP1, HJURP) | 44.4% (42.2, 46.6) / 100.0% (100.0, 100.0) | 0.9% (0.7, 1.2) / — | — / 0.0% (0.0, 0.0) | — / 0.0% (0.0, 0.0) | \| | \| |  |  |  |
| │ └── CliXO:406 (EDEM1, THOP1, SMPD2, SLC26A6, MAP3K9, RALGPS1, ADCY7, ANKDD1A, AVL9, ILF2, GCLM, CTBS, PM20D2, ATP10D, ELL2, GRINA, SESN3, NMNAT2, BRAF, TMEM106B, CNNM4, WDR11, CDO1, PEBP1, SLC38A1, LHFPL2, NCEH1, FANCE, SHANK2, NUCB2, DHX29, CPOX, GPR107, HMGCR, MIOS, CCDC113, GAN, CDS1, XPO4, PCYOX1, INTS7, AZIN1, SPG21, G3BP2, FBN2, YTHDC2, ZNF512B, TMEM141, SACM1L, BRCA1, ERLIN2, AK2, PRKAB2, HMMR, PLBD1, FLVCR2, PHKA2, PDP2, SLC38A6, SLC39A8, NPL, SLC11A1, PTPRJ, C1orf21, MME, DUSP4, KLHL3, RCAN3, APH1B, DUS2, PIK3CB, LDLR, SIPA1L1, SLC24A3, MAT2A, MKNK1, SLC16A3, HGF, TP53BP2, LSM1, NHLRC2, MAN1A2, NPEPPS, INPP5F, TLCD1, TMEM268, CSNK2A2, KLHL42, PKD2, PPP2R1B, HSPA9, GGCX, GPT2, EZH2, TKFC, CHEK1, SPON1, SCMH1, PLAG1, STX6, EPB41L5, DONSON, IDE, GEMIN5, RAD51, MAFF, GPD1L, TOP2B, FNDC3A, STAT5B, CLTC, GADD45G, ODF2, VCP, RHOBTB3, TMEM154, DOCK8, MELK, SMARCA2, ETNK2, ARHGAP28, ESRP1, HJURP, UBE2H) | 44.4% (42.2, 46.6) / 100.0% (100.0, 100.0) | 0.9% (0.7, 1.2) / — | — / 0.0% (0.0, 0.0) | — / 0.0% (0.0, 0.0) | \| | \| |  |  |  |
| │ └── CliXO:400 (EDEM1, THOP1, SMPD2, MAP3K9, ANKDD1A, AVL9, ILF2, GCLM, CTBS, PM20D2, GRINA, SESN3, NMNAT2, TMEM106B, CNNM4, WDR11, PEBP1, SLC38A1, LHFPL2, NCEH1, SHANK2, CPOX, GPR107, HMGCR, CDS1, XPO4, INTS7, SPG21, G3BP2, FBN2, ZNF512B, TMEM141, SACM1L, BRCA1, ERLIN2, AK2, PRKAB2, PLBD1, PDP2, SLC38A6, SLC39A8, NPL, C1orf21, MME, RCAN3, DUS2, LDLR, SIPA1L1, SLC24A3, MAT2A, MKNK1, SLC16A3, HGF, TP53BP2, LSM1, NHLRC2, INPP5F, TMEM268, KLHL42, PKD2, PPP2R1B, HSPA9, GGCX, TKFC, CHEK1, SPON1, PLAG1, EPB41L5, DONSON, IDE, GEMIN5, RAD51, GPD1L, TOP2B, CLTC, ODF2, VCP, DOCK8, SMARCA2, ARHGAP28, UBE2H) | 44.4% (42.2, 46.6) / 100.0% (100.0, 100.0) | 0.9% (0.7, 1.2) / — | — / 0.0% (0.0, 0.0) | — / 0.0% (0.0, 0.0) | \| | \| |  |  |  |
| │ └── CliXO:375 (HMGCR, ZNF512B, SACM1L, PKD2, DOCK8, SMARCA2) | 44.4% (42.2, 46.6) / 100.0% (100.0, 100.0) | 0.9% (0.7, 1.2) / — | — / 0.0% (0.0, 0.0) | — / 0.0% (0.0, 0.0) | \| | \| |  |  |  |
| └── CliXO:415 (CX3CR1, SLIT3, CEBPD, MYO5A, SLC2A1, GNG11, DNAJC1, AKAP13, SCARB1, ZBTB20, PTPN12, IGF1, KIAA0232, CBX5, DYRK2, ETS2, MEF2A, GPER1, ZNF462, IFI44L, CYTL1, GSN, PARM1, NFKB1, RAB3B, SEMA3G, ST6GAL1, MCTP2, SYK, NFIA, TXNDC11, SMIM3, LRRC8A, JAK2, CDK14, MS4A4A, LAPTM5, JUNB, F8, RIN2, CLIC5, CARD16, PRKCE, KIF5B, CCDC59, TBXAS1, DDIT4, GNB1, EXOC6, TMTC4, SAV1, NECTIN4, PPP2R5A, KMT2E, FZD1, CYBB, DPYD, ELMO1, SNAPC1, C3AR1, TSPAN33, HMGN3, MIDN, LTF, RIMKLA, TRIP10, CLOCK, TACC1, FLI1, MBTPS2, MXI1, RNASE1, MAML2, SYPL1, AMOTL2, DDR2, ACTN1, TDP1, TANK, ARRDC3, YPEL2, GALNT18, TYROBP, DUSP1, ZNF281, LAMA4, EFHD1, EDEM1, SBF2, TEX2, THOP1, PPP3CA, TLR7, THRA, LRRC32, HSPA2, PIK3AP1, TSPAN14, ZFYVE1, CHIC2, MLLT1, SLC9A9, MINPP1, DIO2, SULF2, ASTE1, PLCB1, RNF11, CMKLR1, PODXL, SDC4, WDSUB1, PDGFC, SMPD2, SLC26A6, PGRMC1, SLC6A8, GUCA2A, TLR4, MXRA5, MAP3K9, BCORL1, HK1, RCBTB2, ASAP2, RALGPS1, ADCY7, ANGPT1, ARL6IP5, RASAL2, ARTN, ANKDD1A, AVL9, MATN2, ILF2, GCLM, CTBS, GOLM1, PROM1, PM20D2, EBF3, SH2B3, ATP10D, IL33, HNMT, DKK3, ELL2, GRINA, PDE5A, MAP4K4, SESN3, NMNAT2, BRAF, INSL4, NIPAL3, TRIB2, ARID3A, KDM1A, TMEM106B, VEGFA, CNNM4, WDR11, MT1F, CREBL2, PKM, CDO1, NUDT5, HEY1, PEBP1, SLC38A1, LHFPL2, NCEH1, SEMA4C, ZMYND11, PLOD2, TRPC6, FANCE, VEGFC, SHANK2, PFKFB4, ACVRL1, SLC35B3, GBP4, EDN1, NUCB2, DHX29, ADARB1, OLFML2B, CPOX, PAPSS1, GPR107, HMGCR, MIOS, ACSF2, DLX5, CCDC113, GAN, TMEM176B, FCN3, ARHGAP24, CDS1, KIFC3, XPO4, LRCH1, PCYOX1, INTS7, AZIN1, SPG21, APLN, WNK2, G3BP2, FGF13, SLCO2B1, LIMCH1, AGTR1, FBN2, SARAF, CNOT6L, TRIP13, YTHDC2, ZNF512B, TMEM141, TAPT1, INSIG1, RNF20, BRCA1, ERLIN2, CADM3, AMD1, GLA, PUS7, CKS2, ROBO4, AK2, PRKAB2, BTD, HMMR, PLBD1, LEPROTL1, WDFY2, FLVCR2, PHKA2, PDP2, DCXR, SLC38A6, SLC39A8, NPL, SLC11A1, PTPRJ, C1orf21, MME, DUSP4, MXD1, APH1B, DUS2, TMEM43, PIK3CB, ABLIM3, GRHL1, DAB2, LDLR, SIPA1L1, SLC24A3, MAT2A, MKNK1, SLC16A3, GRHL3, EPS8L1, HGF, TP53BP2, LSM1, MOB3C, NHLRC2, CDYL2, KIF2A, MAN1A2, PAPPA, NPEPPS, INPP5F, NEK7, TLCD1, TMEM268, CSNK2A2, CCSAP, KIF17, ZNF75A, KLHL42, PKD2, NIN, PPP2R1B, HSPA9, GGCX, CYP19A1, GPT2, SH3RF1, EZH2, LURAP1L, CHEK1, SPON1, SCMH1, PLAG1, STX6, EPB41L5, DONSON, IDE, PDZD2, PRKCH, RAD51, MAFF, GPD1L, TOP2B, PREP, ARHGAP26, FNDC3A, STAT5B, CLTC, GADD45G, ODF2, VCP, RHOBTB3, TMEM154, DOCK8, MELK, SMARCA2, TTI1, MTMR4, ETNK2, ARHGAP28, ESRP1, HJURP, UBE2H) | 64.6% (61.9, 67.3) / 77.2% (74.8, 79.5) | 1.1% (0.8, 1.4) / — | 78.2% (75.6, 80.7) / 66.0% (63.1, 69.0) | 100.0% (100.0, 100.0) / 14.2% (13.4, 15.0) | \|✓ | \| |  |  |  |
| ├── CliXO:411 (CX3CR1, SLIT3, CEBPD, MYO5A, SLC2A1, GNG11, DNAJC1, SCARB1, ZBTB20, PTPN12, IGF1, KIAA0232, CBX5, DYRK2, ETS2, GPER1, ZNF462, IFI44L, CYTL1, PARM1, NFKB1, RAB3B, SEMA3G, ST6GAL1, MCTP2, SYK, NFIA, TXNDC11, SMIM3, LRRC8A, JAK2, CDK14, MS4A4A, JUNB, RIN2, CLIC5, CARD16, PRKCE, KIF5B, CCDC59, TBXAS1, DDIT4, GNB1, EXOC6, SAV1, NECTIN4, PPP2R5A, KMT2E, FZD1, CYBB, DPYD, ELMO1, SNAPC1, C3AR1, TSPAN33, MIDN, LTF, RIMKLA, TRIP10, CLOCK, TACC1, FLI1, MBTPS2, MXI1, RNASE1, MAML2, SYPL1, AMOTL2, TDP1, TANK, YPEL2, GALNT18, TYROBP, DUSP1, ZNF281, EFHD1, SBF2, TEX2, TLR7, THRA, LRRC32, HSPA2, PIK3AP1, TSPAN14, ZFYVE1, CHIC2, MLLT1, SLC9A9, MINPP1, ASTE1, PLCB1, RNF11, CMKLR1, PODXL, PDGFC, GUCA2A, TLR4, MXRA5, BCORL1, RCBTB2, ASAP2, ANGPT1, ARL6IP5, RASAL2, ARTN, MATN2, PROM1, EBF3, SH2B3, IL33, DKK3, PDE5A, MAP4K4, TRIB2, CREBL2, HEY1, ZMYND11, TRPC6, VEGFC, ACVRL1, SLC35B3, GBP4, EDN1, ADARB1, OLFML2B, TMEM176B, FCN3, ARHGAP24, KIFC3, LRCH1, APLN, FGF13, SLCO2B1, LIMCH1, AGTR1, SARAF, CNOT6L, TAPT1, INSIG1, RNF20, CADM3, ROBO4, LEPROTL1, PTPRJ, DUSP4, TMEM43, ABLIM3, DAB2, MOB3C, CDYL2, KIF2A, PAPPA, KIF17, NIN, LURAP1L, PDZD2, PRKCH, MAFF, ARHGAP26, FNDC3A, STAT5B, RHOBTB3, MTMR4) | 64.6% (61.9, 67.3) / 77.2% (74.8, 79.5) | 1.1% (0.8, 1.4) / — | 78.2% (75.6, 80.7) / 66.0% (63.1, 69.0) | 100.0% (100.0, 100.0) / 14.2% (13.4, 15.0) | \|✓ | \| |  |  |  |
| │ ├── CliXO:353 (CYBB, C3AR1) | 44.4% (42.2, 46.6) / 100.0% (100.0, 100.0) | 0.9% (0.7, 1.2) / — | — / 0.0% (0.0, 0.0) | — / 0.0% (0.0, 0.0) | \| | \| |  |  |  |
| │ ├── CliXO:361 (PIK3AP1, TLR4) | 44.4% (42.2, 46.6) / 100.0% (100.0, 100.0) | 0.9% (0.7, 1.2) / — | — / 0.0% (0.0, 0.0) | — / 0.0% (0.0, 0.0) | \| | \| |  |  |  |
| │ ├── CliXO:386 (GNG11, SCARB1, DYRK2, CYTL1, RAB3B, MCTP2, JAK2, CLIC5, EXOC6, FZD1, TACC1, EFHD1, RCBTB2) | 44.4% (42.2, 46.6) / 100.0% (100.0, 100.0) | 0.9% (0.7, 1.2) / — | — / 0.0% (0.0, 0.0) | — / 0.0% (0.0, 0.0) | \| | \| |  |  |  |
| │ └── CliXO:402 (CEBPD, MYO5A, GNG11, DNAJC1, PTPN12, KIAA0232, CBX5, DYRK2, GPER1, ZNF462, IFI44L, CYTL1, NFKB1, SEMA3G, SYK, TXNDC11, CDK14, PRKCE, CCDC59, TBXAS1, GNB1, SAV1, KMT2E, FZD1, DPYD, SNAPC1, TSPAN33, MIDN, RIMKLA, TRIP10, TACC1, FLI1, MXI1, RNASE1, MAML2, AMOTL2, TDP1, YPEL2, GALNT18, DUSP1, ZNF281, SBF2, TLR7, LRRC32, HSPA2, ZFYVE1, SLC9A9, MINPP1, ASTE1, MXRA5, RCBTB2, ANGPT1, ARL6IP5, MATN2, EBF3, SH2B3, IL33, DKK3, PDE5A, TRIB2, CREBL2, HEY1, TRPC6, ACVRL1, SLC35B3, GBP4, EDN1, ADARB1, TMEM176B, FCN3, ARHGAP24, KIFC3, LRCH1, APLN, FGF13, SLCO2B1, SARAF, CNOT6L, TAPT1, RNF20, ROBO4, LEPROTL1, ABLIM3, DAB2, MOB3C) | 44.4% (42.2, 46.6) / 100.0% (100.0, 100.0) | 0.9% (0.7, 1.2) / — | — / 0.0% (0.0, 0.0) | — / 0.0% (0.0, 0.0) | \| | \| |  |  |  |
| │ ├── CliXO:355 (KIAA0232, PRKCE) | 44.4% (42.2, 46.6) / 100.0% (100.0, 100.0) | 0.9% (0.7, 1.2) / — | — / 0.0% (0.0, 0.0) | — / 0.0% (0.0, 0.0) | \| | \| |  |  | CliXO:417 |
| └── CliXO:414 (CX3CR1, SLIT3, CEBPD, MYO5A, GNG11, DNAJC1, AKAP13, SCARB1, ZBTB20, PTPN12, IGF1, KIAA0232, CBX5, DYRK2, ETS2, MEF2A, GPER1, ZNF462, IFI44L, CYTL1, GSN, PARM1, NFKB1, RAB3B, ST6GAL1, MCTP2, SYK, NFIA, TXNDC11, LRRC8A, JAK2, CDK14, MS4A4A, LAPTM5, JUNB, F8, RIN2, CLIC5, CARD16, PRKCE, KIF5B, CCDC59, TBXAS1, DDIT4, GNB1, EXOC6, TMTC4, PPP2R5A, KMT2E, FZD1, CYBB, DPYD, SNAPC1, C3AR1, TSPAN33, HMGN3, MIDN, LTF, RIMKLA, TRIP10, CLOCK, TACC1, FLI1, MXI1, RNASE1, MAML2, SYPL1, AMOTL2, DDR2, ACTN1, TDP1, TANK, ARRDC3, YPEL2, TYROBP, DUSP1, ZNF281, LAMA4, EFHD1, EDEM1, THOP1, PPP3CA, TLR7, HSPA2, PIK3AP1, TSPAN14, ZFYVE1, CHIC2, MLLT1, SLC9A9, MINPP1, DIO2, SULF2, PLCB1, RNF11, CMKLR1, PODXL, SDC4, WDSUB1, SMPD2, SLC26A6, PGRMC1, SLC6A8, TLR4, MXRA5, MAP3K9, BCORL1, HK1, RCBTB2, ASAP2, RALGPS1, ADCY7, ANGPT1, RASAL2, ANKDD1A, AVL9, MATN2, ILF2, GCLM, CTBS, GOLM1, PM20D2, EBF3, SH2B3, ATP10D, IL33, HNMT, DKK3, ELL2, GRINA, PDE5A, SESN3, NMNAT2, BRAF, INSL4, NIPAL3, TRIB2, ARID3A, KDM1A, TMEM106B, VEGFA, CNNM4, WDR11, MT1F, CREBL2, PKM, CDO1, NUDT5, HEY1, PEBP1, SLC38A1, LHFPL2, NCEH1, SEMA4C, ZMYND11, PLOD2, TRPC6, FANCE, VEGFC, SHANK2, PFKFB4, ACVRL1, SLC35B3, GBP4, EDN1, NUCB2, DHX29, ADARB1, OLFML2B, CPOX, PAPSS1, GPR107, HMGCR, MIOS, ACSF2, DLX5, CCDC113, GAN, TMEM176B, FCN3, ARHGAP24, CDS1, KIFC3, XPO4, PCYOX1, INTS7, AZIN1, SPG21, APLN, WNK2, G3BP2, FGF13, SLCO2B1, LIMCH1, AGTR1, FBN2, SARAF, CNOT6L, TRIP13, YTHDC2, ZNF512B, TMEM141, TAPT1, INSIG1, RNF20, BRCA1, ERLIN2, AMD1, GLA, PUS7, CKS2, ROBO4, AK2, PRKAB2, BTD, HMMR, PLBD1, LEPROTL1, WDFY2, FLVCR2, PHKA2, PDP2, DCXR, SLC38A6, SLC39A8, NPL, SLC11A1, PTPRJ, C1orf21, MME, DUSP4, MXD1, APH1B, DUS2, TMEM43, PIK3CB, ABLIM3, GRHL1, DAB2, LDLR, SIPA1L1, SLC24A3, MAT2A, MKNK1, SLC16A3, GRHL3, EPS8L1, HGF, TP53BP2, LSM1, MOB3C, NHLRC2, CDYL2, KIF2A, MAN1A2, PAPPA, NPEPPS, INPP5F, NEK7, TLCD1, TMEM268, CSNK2A2, CCSAP, KIF17, ZNF75A, KLHL42, PKD2, NIN, PPP2R1B, HSPA9, GGCX, CYP19A1, GPT2, SH3RF1, EZH2, LURAP1L, CHEK1, SPON1, SCMH1, PLAG1, STX6, EPB41L5, DONSON, IDE, PDZD2, PRKCH, RAD51, MAFF, GPD1L, TOP2B, PREP, ARHGAP26, FNDC3A, STAT5B, CLTC, GADD45G, ODF2, VCP, RHOBTB3, TMEM154, DOCK8, MELK, SMARCA2, TTI1, MTMR4, ETNK2, ARHGAP28, ESRP1, HJURP, UBE2H) | 44.4% (42.2, 46.6) / 100.0% (100.0, 100.0) | 0.9% (0.7, 1.2) / — | — / 0.0% (0.0, 0.0) | — / 0.0% (0.0, 0.0) | \| | \| |  |  |  |
| ├── CliXO:388 (AKAP13, ETS2, MEF2A, GSN, PARM1, ST6GAL1, LRRC8A, F8, KIF5B, DDIT4, LTF, DDR2, LAMA4, SULF2, PODXL, EDN1) | 44.4% (42.2, 46.6) / 100.0% (100.0, 100.0) | 0.9% (0.7, 1.2) / — | — / 0.0% (0.0, 0.0) | — / 0.0% (0.0, 0.0) | \| | \| |  |  | CliXO:399 |
| ├── CliXO:404 (CX3CR1, SLIT3, CEBPD, MYO5A, GNG11, AKAP13, ZBTB20, PTPN12, IGF1, KIAA0232, DYRK2, ETS2, MEF2A, GPER1, ZNF462, CYTL1, GSN, PARM1, RAB3B, ST6GAL1, MCTP2, NFIA, TXNDC11, LRRC8A, JAK2, MS4A4A, JUNB, F8, RIN2, CARD16, PRKCE, KIF5B, CCDC59, EXOC6, KMT2E, FZD1, CYBB, DPYD, C3AR1, TSPAN33, MIDN, LTF, RIMKLA, TRIP10, CLOCK, TACC1, FLI1, MAML2, SYPL1, AMOTL2, DDR2, TANK, YPEL2, ZNF281, LAMA4, EFHD1, TLR7, PIK3AP1, TSPAN14, ZFYVE1, CHIC2, MLLT1, MINPP1, PLCB1, RNF11, CMKLR1, PODXL, TLR4, MXRA5, BCORL1, RCBTB2, ASAP2, ANGPT1, RASAL2, MATN2, IL33, DKK3, PDE5A, TRIB2, CREBL2, HEY1, ZMYND11, TRPC6, VEGFC, EDN1, ADARB1, OLFML2B, TMEM176B, FCN3, ARHGAP24, KIFC3, APLN, SLCO2B1, LIMCH1, AGTR1, SARAF, INSIG1, RNF20, AMD1, ROBO4, LEPROTL1, ABLIM3, MOB3C, LURAP1L, MTMR4) | 44.4% (42.2, 46.6) / 100.0% (100.0, 100.0) | 0.9% (0.7, 1.2) / — | — / 0.0% (0.0, 0.0) | — / 0.0% (0.0, 0.0) | \| | \| |  |  |  |
| │ ├── CliXO:377 (AKAP13, ZBTB20, ST6GAL1, NFIA, F8, KIF5B, CLOCK) | 44.4% (42.2, 46.6) / 100.0% (100.0, 100.0) | 0.9% (0.7, 1.2) / — | — / 0.0% (0.0, 0.0) | — / 0.0% (0.0, 0.0) | \| | \| |  |  |  |
| │ └── CliXO:397 (GNG11, PTPN12, KIAA0232, DYRK2, CYTL1, RAB3B, MCTP2, JAK2, MS4A4A, RIN2, CARD16, PRKCE, EXOC6, FZD1, CYBB, C3AR1, TACC1, FLI1, SYPL1, TANK, YPEL2, EFHD1, TLR7, PIK3AP1, CHIC2, MINPP1, PLCB1, RNF11, CMKLR1, TLR4, RCBTB2, ASAP2, ANGPT1, MATN2, IL33, DKK3, PDE5A, CREBL2, TRPC6, VEGFC, ADARB1, OLFML2B, TMEM176B, ARHGAP24, SLCO2B1, LIMCH1, AGTR1, SARAF, INSIG1, RNF20, AMD1, LEPROTL1, ABLIM3, MOB3C, LURAP1L, MTMR4) | 44.4% (42.2, 46.6) / 100.0% (100.0, 100.0) | 0.9% (0.7, 1.2) / — | — / 0.0% (0.0, 0.0) | — / 0.0% (0.0, 0.0) | \| | \| |  |  | CliXO:417 |
| └── CliXO:413 (CX3CR1, SLIT3, CEBPD, MYO5A, GNG11, DNAJC1, AKAP13, SCARB1, ZBTB20, PTPN12, IGF1, KIAA0232, CBX5, DYRK2, ETS2, MEF2A, GPER1, ZNF462, IFI44L, CYTL1, GSN, PARM1, NFKB1, RAB3B, ST6GAL1, MCTP2, SYK, NFIA, TXNDC11, LRRC8A, JAK2, CDK14, MS4A4A, LAPTM5, JUNB, F8, RIN2, CLIC5, CARD16, PRKCE, KIF5B, CCDC59, TBXAS1, DDIT4, GNB1, EXOC6, PPP2R5A, KMT2E, FZD1, CYBB, DPYD, SNAPC1, C3AR1, TSPAN33, MIDN, LTF, RIMKLA, TRIP10, TACC1, FLI1, MXI1, RNASE1, MAML2, SYPL1, AMOTL2, DDR2, TDP1, TANK, ARRDC3, YPEL2, TYROBP, DUSP1, ZNF281, LAMA4, EFHD1, EDEM1, THOP1, TLR7, HSPA2, PIK3AP1, TSPAN14, ZFYVE1, CHIC2, MLLT1, SLC9A9, MINPP1, PLCB1, RNF11, CMKLR1, PODXL, WDSUB1, SMPD2, SLC26A6, TLR4, MXRA5, MAP3K9, BCORL1, RCBTB2, ASAP2, RALGPS1, ADCY7, ANGPT1, RASAL2, ANKDD1A, AVL9, MATN2, ILF2, GCLM, CTBS, PM20D2, EBF3, SH2B3, ATP10D, DKK3, ELL2, GRINA, PDE5A, SESN3, NMNAT2, BRAF, INSL4, TRIB2, TMEM106B, CNNM4, WDR11, CDO1, HEY1, SLC38A1, LHFPL2, NCEH1, ZMYND11, TRPC6, VEGFC, SHANK2, ACVRL1, SLC35B3, GBP4, EDN1, NUCB2, DHX29, ADARB1, OLFML2B, CPOX, GPR107, MIOS, CCDC113, GAN, TMEM176B, FCN3, ARHGAP24, CDS1, KIFC3, XPO4, INTS7, AZIN1, SPG21, APLN, WNK2, G3BP2, FGF13, SLCO2B1, LIMCH1, AGTR1, FBN2, SARAF, CNOT6L, TRIP13, YTHDC2, TMEM141, TAPT1, INSIG1, RNF20, BRCA1, AMD1, ROBO4, AK2, PRKAB2, BTD, HMMR, PLBD1, LEPROTL1, WDFY2, FLVCR2, PHKA2, PDP2, SLC38A6, SLC39A8, NPL, SLC11A1, PTPRJ, DUSP4, MXD1, APH1B, DUS2, TMEM43, PIK3CB, ABLIM3, GRHL1, DAB2, SIPA1L1, MAT2A, MKNK1, EPS8L1, TP53BP2, LSM1, MOB3C, NHLRC2, CDYL2, KIF2A, MAN1A2, PAPPA, INPP5F, NEK7, TLCD1, TMEM268, CSNK2A2, CCSAP, KIF17, ZNF75A, KLHL42, PKD2, NIN, PPP2R1B, HSPA9, GGCX, CYP19A1, GPT2, EZH2, LURAP1L, CHEK1, PLAG1, DONSON, IDE, PDZD2, PRKCH, RAD51, MAFF, GPD1L, TOP2B, ARHGAP26, FNDC3A, STAT5B, CLTC, GADD45G, ODF2, VCP, RHOBTB3, DOCK8, MELK, SMARCA2, MTMR4, ETNK2, ARHGAP28, ESRP1, HJURP) | 44.4% (42.2, 46.6) / 100.0% (100.0, 100.0) | 0.9% (0.7, 1.2) / — | — / 0.0% (0.0, 0.0) | — / 0.0% (0.0, 0.0) | \| | \| |  |  |  |
| ├── CliXO:399 (CX3CR1†, SLIT3, CEBPD, MYO5A, DNAJC1, AKAP13, ZBTB20, IGF1, CBX5, ETS2, MEF2A†, GPER1, ZNF462, IFI44L, GSN, PARM1, ST6GAL1, SYK, NFIA, TXNDC11, LRRC8A, CDK14, LAPTM5, JUNB, F8, KIF5B, CCDC59, TBXAS1, GNB1, PPP2R5A, KMT2E, DPYD, SNAPC1, TSPAN33, MIDN, RIMKLA, FLI1, MXI1, RNASE1, AMOTL2, DDR2, TDP1, TYROBP, DUSP1, ZNF281, TLR7, TSPAN14, ZFYVE1, SLC9A9, PODXL, TLR4, ANGPT1, RASAL2, SH2B3, PDE5A, TRIB2, HEY1, ZMYND11, TRPC6, VEGFC, ACVRL1, GBP4, EDN1, ADARB1, OLFML2B, TMEM176B, FCN3, ARHGAP24, KIFC3, APLN, SLCO2B1, AGTR1, SARAF, CNOT6L, ROBO4, LEPROTL1, ABLIM3, LURAP1L) | 61.4% (58.9, 64.0) / 84.6% (82.5, 86.7) | 0.0% (0.0, 0.0) / — | 82.4% (80.0, 84.8) / 57.6% (54.9, 60.4) | 98.8% (98.5, 99.2) / 79.6% (78.7, 80.4) | \|✓ | \|✓ | ✓ |  | CliXO:399 |
| │ └── CliXO:398 (CX3CR1, SLIT3, CEBPD, MYO5A, DNAJC1, AKAP13, ZBTB20, IGF1, CBX5, ETS2, MEF2A, GPER1, ZNF462, IFI44L, GSN, PARM1, ST6GAL1, NFIA, TXNDC11, LRRC8A, CDK14, JUNB, F8, KIF5B, CCDC59, TBXAS1, GNB1, KMT2E, SNAPC1, TSPAN33, MIDN, RIMKLA, FLI1, MXI1, AMOTL2, DDR2, TDP1, TYROBP, DUSP1, TSPAN14, ZFYVE1, PODXL, ANGPT1, RASAL2, SH2B3, PDE5A, TRIB2, HEY1, ZMYND11, TRPC6, ACVRL1, GBP4, EDN1, TMEM176B, FCN3, KIFC3, APLN, AGTR1, CNOT6L, ROBO4, LEPROTL1, ABLIM3) | 44.4% (42.2, 46.6) / 100.0% (100.0, 100.0) | 0.9% (0.7, 1.2) / — | — / 0.0% (0.0, 0.0) | — / 0.0% (0.0, 0.0) | \| | \| |  |  | CliXO:399 |
| └── CliXO:408 (GNG11, SCARB1, DYRK2, CYTL1, RAB3B, MCTP2, JAK2, CLIC5, EXOC6, FZD1, TACC1, TANK, ARRDC3, EFHD1, EDEM1, THOP1, RNF11, WDSUB1, SMPD2, SLC26A6, MAP3K9, RCBTB2, RALGPS1, ADCY7, ANKDD1A, AVL9, ILF2, GCLM, CTBS, PM20D2, ATP10D, ELL2, GRINA, SESN3, NMNAT2, BRAF, INSL4, TMEM106B, CNNM4, WDR11, CDO1, SLC38A1, LHFPL2, NCEH1, SHANK2, NUCB2, DHX29, CPOX, GPR107, MIOS, CCDC113, GAN, CDS1, XPO4, INTS7, AZIN1, SPG21, WNK2, G3BP2, FBN2, TRIP13, YTHDC2, TMEM141, TAPT1, BRCA1, AK2, PRKAB2, BTD, HMMR, PLBD1, WDFY2, FLVCR2, PHKA2, PDP2, SLC38A6, SLC39A8, NPL, SLC11A1, PTPRJ, DUSP4, MXD1, APH1B, DUS2, TMEM43, PIK3CB, GRHL1, DAB2, SIPA1L1, MAT2A, MKNK1, EPS8L1, TP53BP2, LSM1, NHLRC2, CDYL2, KIF2A, MAN1A2, PAPPA, INPP5F, NEK7, TLCD1, TMEM268, CSNK2A2, CCSAP, KIF17, ZNF75A, KLHL42, PKD2, NIN, PPP2R1B, HSPA9, GGCX, CYP19A1, GPT2, EZH2, CHEK1, PLAG1, DONSON, IDE, PDZD2, PRKCH, RAD51, MAFF, GPD1L, TOP2B, ARHGAP26, FNDC3A, STAT5B, CLTC, GADD45G, ODF2, VCP, RHOBTB3, DOCK8, MELK, SMARCA2, MTMR4, ETNK2, ARHGAP28, ESRP1, HJURP) | 44.4% (42.2, 46.6) / 100.0% (100.0, 100.0) | 0.9% (0.7, 1.2) / — | — / 0.0% (0.0, 0.0) | — / 0.0% (0.0, 0.0) | \| | \| |  |  |  |

The Reported column indicates the ontology from Tables 1–2 to which this row’s eligible genes belong. An ontology is reported if it passes all criteria (C1–C4) and its eligible gene set is not a strict subset of another passing ontology’s gene set. The column is empty when the ontology has no eligible genes or does not pass all criteria. ✓, the ontology passed the criterion; †, the gene had a grad-CAM value >97.5th percentile in the same ontology; |, separator between PPV/TPR and NPV/TNR results in C1 and C2 columns; AUC-ROC, area under the receiver operating characteristic curve; C1, lower bounds finite and >0; C2, absolute difference ≤25% between development and test sets; C3, at least 1 eligible gene; C4, lower bound of AUC-ROC >0.5 in both sets; CI, confidence interval; Dev, development set; grad-CAM, gradient-weighted class activation mapping; HELLP, hemolysis, elevated liver enzyme, and low platelet; NPV, negative predictive value; PE, preeclampsia; PPV, positive predictive value; TNR, true negative rate; TPR, true positive rate.

Table S14. AUC-ROC, AUC-PRC, and RMSE for the early-onset, severe PE pathogenesis model connected with first-trimester placenta.

| Ontology | AUC-ROC (Dev) | AUC-ROC (Test) | AUC-PRC (Dev) | AUC-PRC (Test) | RMSE (Dev) | RMSE (Test) | C1 | C2 | C3 | C4 | Reported |
| --- | --- | --- | --- | --- | --- | --- | --- | --- | --- | --- | --- |
| root (CX3CR1, SLIT3, CEBPD, MYO5A, SLC2A1, GNG11, DNAJC1, AKAP13, SCARB1, ZBTB20, PTPN12, IGF1, KIAA0232, CBX5, DYRK2, ETS2, MEF2A, GPER1, ZNF462, IFI44L, CYTL1, GSN, PARM1, NFKB1, RAB3B, SEMA3G, ST6GAL1, MCTP2, SYK, NFIA, TXNDC11, SMIM3, LRRC8A, JAK2, CDK14, MS4A4A, LAPTM5, JUNB, F8, RIN2, CLIC5, CARD16, PRKCE, KIF5B, CCDC59, TBXAS1, DDIT4, GNB1, EXOC6, CCND1, TMTC4, SAV1, NECTIN4, PPP2R5A, KMT2E, FZD1, CYBB, DPYD, ELMO1, SNAPC1, C3AR1, TSPAN33, HMGN3, MIDN, LTF, RIMKLA, TRIP10, CLOCK, TACC1, FLI1, MBTPS2, MXI1, RNASE1, MAML2, SYPL1, AMOTL2, DDR2, ACTN1, TDP1, MIIP, TANK, ARRDC3, YPEL2, GALNT18, TYROBP, DUSP1, ZNF281, LAMA4, EFHD1, EDEM1, SBF2, TEX2, THOP1, PPP3CA, TLR7, THRA, LRRC32, HSPA2, PIK3AP1, TSPAN14, ZFYVE1, MRI1, CHIC2, MLLT1, COL6A1, SLC9A9, MINPP1, DIO2, SULF2, ASTE1, PLCB1, RNF11, CMKLR1, ZNF331, PODXL, SDC4, WDSUB1, HMOX1, PDGFC, SMPD2, SLC26A6, PGRMC1, SLC6A8, GUCA2A, TLR4, BCL2L11, MXRA5, MAP3K9, BCORL1, HK1, RCBTB2, ASAP2, RALGPS1, ADCY7, TMCC3, ANGPT1, ARL6IP5, RASAL2, ARTN, RASEF, ANKDD1A, AVL9, SYDE1, MATN2, ILF2, GCLM, CTBS, GOLM1, PROM1, PM20D2, EBF3, SH2B3, ATP10D, IL33, ERRFI1, HNMT, DKK3, ELL2, GRINA, PDE5A, MAP4K4, SESN3, NMNAT2, BRAF, INSL4, NIPAL3, TRIB2, ARID3A, KDM1A, TMEM106B, VEGFA, CNNM4, WDR11, MT1F, CREBL2, PKM, CDO1, NUDT5, HEY1, PEBP1, SLC38A1, LHFPL2, NCEH1, SEMA4C, ZMYND11, PLOD2, TRPC6, FANCE, VEGFC, PMAIP1, SHANK2, PFKFB4, ACVRL1, SLC35B3, GALK1, GBP4, EDN1, NUCB2, DHX29, ADARB1, OLFML2B, CPOX, PAPSS1, GPR107, HMGCR, MIOS, ACSF2, DLX5, CCDC113, GAN, TMEM176B, FCN3, ARHGAP24, CDS1, KIFC3, XPO4, LRCH1, PCYOX1, INTS7, AZIN1, SPG21, APLN, WNK2, G3BP2, FGF13, SLCO2B1, LIMCH1, AGTR1, FBN2, SARAF, CNOT6L, TRIP13, YTHDC2, ZNF512B, TMEM141, TAPT1, SACM1L, INSIG1, RNF20, BRCA1, ERLIN2, CADM3, AMD1, GLA, PUS7, CKS2, ROBO4, AK2, PRKAB2, BTD, HMMR, PLBD1, LEPROTL1, WDFY2, FLVCR2, PHKA2, PDP2, DCXR, SLC38A6, SLC39A8, NPL, SLC11A1, CTSA, PTPRJ, C1orf21, MME, DUSP4, MXD1, KLHL3, RCAN3, APH1B, FOLR1, DUS2, SLC44A3, TMEM43, PIK3CB, ABLIM3, GRHL1, DAB2, LDLR, SIPA1L1, SLC24A3, MAT2A, MKNK1, SLC16A3, GRHL3, EPS8L1, HGF, TP53BP2, LSM1, MOB3C, NHLRC2, CDYL2, KIF2A, MAN1A2, PAPPA, NPEPPS, INPP5F, NEK7, TLCD1, RASSF8, TMEM268, CSNK2A2, CCSAP, KIF17, ZNF75A, KLHL42, PKD2, NIN, PPP2R1B, HSPA9, GGCX, CYP19A1, GPT2, SH3RF1, EZH2, TKFC, LURAP1L, CHEK1, SPON1, SCMH1, PLAG1, STX6, EPB41L5, DONSON, IDE, PDZD2, GEMIN5, PRKCH, RAD51, MAFF, GPD1L, TOP2B, PREP, ARHGAP26, FNDC3A, STAT5B, CLTC, GADD45G, ODF2, VCP, RHOBTB3, TMEM154, DOCK8, MELK, SMARCA2, TTI1, MTMR4, ETNK2, ARHGAP28, ESRP1, HJURP, UBE2H) | 0.500 (0.500, 0.500) | 0.433 (0.371, 0.495) | 0.719 | 0.504 | 0.642 (±0.0121) | 0.847 (±0.0011) | \| | \| |  |  |  |
| CliXO:418 (CX3CR1, SLIT3, CEBPD, MYO5A, SLC2A1, GNG11, DNAJC1, AKAP13, SCARB1, ZBTB20, PTPN12, IGF1, KIAA0232, CBX5, DYRK2, ETS2, MEF2A, GPER1, ZNF462, IFI44L, CYTL1, GSN, PARM1, NFKB1, RAB3B, SEMA3G, ST6GAL1, MCTP2, SYK, NFIA, TXNDC11, SMIM3, LRRC8A, JAK2, CDK14, MS4A4A, LAPTM5, JUNB, F8, RIN2, CLIC5, CARD16, PRKCE, KIF5B, CCDC59, TBXAS1, DDIT4, GNB1, EXOC6, CCND1, TMTC4, SAV1, NECTIN4, PPP2R5A, KMT2E, FZD1, CYBB, DPYD, ELMO1, SNAPC1, C3AR1, TSPAN33, HMGN3, MIDN, LTF, RIMKLA, TRIP10, CLOCK, TACC1, FLI1, MBTPS2, MXI1, RNASE1, MAML2, SYPL1, AMOTL2, DDR2, ACTN1, TDP1, MIIP, TANK, ARRDC3, YPEL2, GALNT18, TYROBP, DUSP1, ZNF281, LAMA4, EFHD1, EDEM1, SBF2, TEX2, THOP1, PPP3CA, TLR7, THRA, LRRC32, HSPA2, PIK3AP1, TSPAN14, ZFYVE1, MRI1, CHIC2, MLLT1, COL6A1, SLC9A9, MINPP1, DIO2, SULF2, ASTE1, PLCB1, RNF11, CMKLR1, ZNF331, PODXL, SDC4, WDSUB1, HMOX1, PDGFC, SMPD2, SLC26A6, PGRMC1, SLC6A8, GUCA2A, TLR4, BCL2L11, MXRA5, MAP3K9, BCORL1, HK1, RCBTB2, ASAP2, RALGPS1, ADCY7, TMCC3, ANGPT1, ARL6IP5, RASAL2, ARTN, RASEF, ANKDD1A, AVL9, SYDE1, MATN2, ILF2, GCLM, CTBS, GOLM1, PROM1, PM20D2, EBF3, SH2B3, ATP10D, IL33, ERRFI1, HNMT, DKK3, ELL2, GRINA, PDE5A, MAP4K4, SESN3, NMNAT2, BRAF, INSL4, NIPAL3, TRIB2, ARID3A, KDM1A, TMEM106B, VEGFA, CNNM4, WDR11, MT1F, CREBL2, PKM, CDO1, NUDT5, HEY1, PEBP1, SLC38A1, LHFPL2, NCEH1, SEMA4C, ZMYND11, PLOD2, TRPC6, FANCE, VEGFC, PMAIP1, SHANK2, PFKFB4, ACVRL1, SLC35B3, GALK1, GBP4, EDN1, NUCB2, DHX29, ADARB1, OLFML2B, CPOX, PAPSS1, GPR107, HMGCR, MIOS, ACSF2, DLX5, CCDC113, GAN, TMEM176B, FCN3, ARHGAP24, CDS1, KIFC3, XPO4, LRCH1, PCYOX1, INTS7, AZIN1, SPG21, APLN, WNK2, G3BP2, FGF13, SLCO2B1, LIMCH1, AGTR1, FBN2, SARAF, CNOT6L, TRIP13, YTHDC2, ZNF512B, TMEM141, TAPT1, SACM1L, INSIG1, RNF20, BRCA1, ERLIN2, CADM3, AMD1, GLA, PUS7, CKS2, ROBO4, AK2, PRKAB2, BTD, HMMR, PLBD1, LEPROTL1, WDFY2, FLVCR2, PHKA2, PDP2, DCXR, SLC38A6, SLC39A8, NPL, SLC11A1, CTSA, PTPRJ, C1orf21, MME, DUSP4, MXD1, KLHL3, RCAN3, APH1B, FOLR1, DUS2, SLC44A3, TMEM43, PIK3CB, ABLIM3, GRHL1, DAB2, LDLR, SIPA1L1, SLC24A3, MAT2A, MKNK1, SLC16A3, GRHL3, EPS8L1, HGF, TP53BP2, LSM1, MOB3C, NHLRC2, CDYL2, KIF2A, MAN1A2, PAPPA, NPEPPS, INPP5F, NEK7, TLCD1, RASSF8, TMEM268, CSNK2A2, CCSAP, KIF17, ZNF75A, KLHL42, PKD2, NIN, PPP2R1B, HSPA9, GGCX, CYP19A1, GPT2, SH3RF1, EZH2, TKFC, LURAP1L, CHEK1, SPON1, SCMH1, PLAG1, STX6, EPB41L5, DONSON, IDE, PDZD2, GEMIN5, PRKCH, RAD51, MAFF, GPD1L, TOP2B, PREP, ARHGAP26, FNDC3A, STAT5B, CLTC, GADD45G, ODF2, VCP, RHOBTB3, TMEM154, DOCK8, MELK, SMARCA2, TTI1, MTMR4, ETNK2, ARHGAP28, ESRP1, HJURP, UBE2H) | 0.272 (0.251, 0.292) | 0.491 (0.421, 0.561) | 0.395 | 0.504 | 0.513 (±0.0029) | 0.569 (±0.0003) | \| | \| |  |  |  |
| ├── CliXO:410 (SLC2A1, SMIM3, TMTC4, NECTIN4, HMGN3, ACTN1, MIIP, ARRDC3, GALNT18, EFHD1, PPP3CA, COL6A1, DIO2, RNF11, SDC4, WDSUB1, HMOX1, SMPD2, PGRMC1, SLC6A8, BCL2L11, MAP3K9, HK1, RALGPS1, ADCY7, RASEF, AVL9, ILF2, GCLM, GOLM1, PM20D2, ATP10D, ERRFI1, HNMT, ELL2, GRINA, SESN3, NMNAT2, BRAF, INSL4, NIPAL3†, ARID3A, KDM1A, TMEM106B, VEGFA, CNNM4, MT1F, PKM, CDO1, NUDT5, PEBP1, LHFPL2, NCEH1, SEMA4C, PLOD2, FANCE, PMAIP1, SHANK2, PFKFB4, GALK1, NUCB2, DHX29, CPOX, PAPSS1, GPR107, HMGCR, MIOS, ACSF2, DLX5, CCDC113, GAN, CDS1, PCYOX1, AZIN1, SPG21, G3BP2, FBN2, TRIP13, ZNF512B, TMEM141, SACM1L, BRCA1, ERLIN2, GLA, CKS2, AK2, PRKAB2, HMMR, PLBD1, FLVCR2, PHKA2, DCXR, SLC38A6, SLC39A8, NPL, SLC11A1, CTSA, C1orf21, MME, KLHL3, RCAN3, APH1B, FOLR1, DUS2, SLC44A3, PIK3CB, GRHL1, LDLR, SIPA1L1, SLC24A3, MAT2A, MKNK1, SLC16A3, GRHL3, HGF, TP53BP2, CDYL2, NPEPPS, INPP5F, TLCD1, RASSF8, TMEM268, PPP2R1B, HSPA9, GGCX, GPT2†, SH3RF1, TKFC, CHEK1, SPON1, SCMH1, PLAG1, STX6, EPB41L5, DONSON, IDE, GEMIN5, RAD51, GPD1L, TOP2B, PREP, CLTC, ODF2, VCP, TMEM154, DOCK8, MELK, TTI1, ETNK2, ARHGAP28, ESRP1, HJURP, UBE2H†) | 0.728 (0.708, 0.749) | 0.376 (0.322, 0.430) | 0.768 | 0.004 | 0.498 (±0.0005) | 0.486 (±0.0001) | \|✓ | \|✓ | ✓ |  | CliXO:410 |
| │ ├── CliXO:354 (DIO2, PGRMC1) | 0.500 (0.500, 0.500) | 0.433 (0.371, 0.495) | 0.719 | 0.504 | 0.502 (±0.0055) | 0.375 (±0.0009) | \| | \| |  |  |  |
| │ ├── CliXO:360 (PEBP1, LDLR) | 0.500 (0.500, 0.500) | 0.433 (0.371, 0.495) | 0.719 | 0.504 | 0.502 (±0.0055) | 0.375 (±0.0009) | \| | \| |  |  |  |
| │ ├── CliXO:362 (SLC2A1, NECTIN4) | 0.500 (0.500, 0.500) | 0.433 (0.371, 0.495) | 0.719 | 0.504 | 0.502 (±0.0055) | 0.375 (±0.0009) | \| | \| |  |  |  |
| │ ├── CliXO:364 (MIIP, BCL2L11, PMAIP1) | 0.500 (0.500, 0.500) | 0.433 (0.371, 0.495) | 0.719 | 0.504 | 0.502 (±0.0055) | 0.375 (±0.0009) | \| | \| |  |  |  |
| │ ├── CliXO:367 (NIPAL3, MT1F, PAPSS1) | 0.500 (0.500, 0.500) | 0.433 (0.371, 0.495) | 0.719 | 0.504 | 0.500 (±0.0049) | 0.390 (±0.0008) | \| | \| |  |  | CliXO:410 |
| │ ├── CliXO:368 (FANCE, SLC16A3, INPP5F, RASSF8) | 0.500 (0.500, 0.500) | 0.433 (0.371, 0.495) | 0.719 | 0.504 | 0.502 (±0.0055) | 0.375 (±0.0009) | \| | \| |  |  |  |
| │ ├── CliXO:374 (HK1, NIPAL3, VEGFA, PKM, GALK1) | 0.500 (0.500, 0.500) | 0.433 (0.371, 0.495) | 0.719 | 0.504 | 0.504 (±0.0013) | 0.530 (±0.0002) | \| | \| |  |  |  |
| │ │ └── CliXO:358 (VEGFA, GALK1) | 0.500 (0.500, 0.500) | 0.433 (0.371, 0.495) | 0.719 | 0.504 | 0.502 (±0.0055) | 0.375 (±0.0009) | \| | \| |  |  |  |
| │ ├── CliXO:378 (GCLM, NMNAT2, TMEM106B, LHFPL2, PMAIP1, CDS1, RCAN3) | 0.500 (0.500, 0.500) | 0.433 (0.371, 0.495) | 0.719 | 0.504 | 0.502 (±0.0055) | 0.375 (±0.0009) | \| | \| |  |  |  |
| │ │ └── CliXO:359 (CDS1, RCAN3) | 0.500 (0.500, 0.500) | 0.433 (0.371, 0.495) | 0.719 | 0.504 | 0.502 (±0.0055) | 0.375 (±0.0009) | \| | \| |  |  |  |
| │ ├── CliXO:379 (HMGN3, PPP3CA, COL6A1, ZNF512B, CKS2, SPON1, EPB41L5) | 0.500 (0.500, 0.500) | 0.433 (0.371, 0.495) | 0.719 | 0.504 | 0.502 (±0.0055) | 0.375 (±0.0009) | \| | \| |  |  |  |
| │ │ └── CliXO:357 (COL6A1, CKS2) | 0.500 (0.500, 0.500) | 0.433 (0.371, 0.495) | 0.719 | 0.504 | 0.502 (±0.0055) | 0.375 (±0.0009) | \| | \| |  |  |  |
| │ ├── CliXO:380 (PEBP1, HMGCR, TRIP13, ERLIN2, C1orf21, SLC24A3, PPP2R1B, GPD1L) | 0.500 (0.500, 0.500) | 0.433 (0.371, 0.495) | 0.719 | 0.504 | 0.502 (±0.0055) | 0.375 (±0.0009) | \| | \| |  |  |  |
| │ ├── CliXO:382 (ACSF2, TRIP13, ZNF512B, DCXR, MME, HGF, NPEPPS, STX6, EPB41L5) | 0.500 (0.500, 0.500) | 0.433 (0.371, 0.495) | 0.719 | 0.504 | 0.502 (±0.0055) | 0.375 (±0.0009) | \| | \| |  |  |  |
| │ ├── CliXO:390 (FANCE, SHANK2, TRIP13, HMMR, PLBD1, PHKA2, DCXR, PIK3CB, GRHL1, SIPA1L1, SLC16A3, TP53BP2, INPP5F, TLCD1, PPP2R1B, GPT2, CHEK1, PLAG1, RAD51, TOP2B, MELK, ESRP1, HJURP) | 0.500 (0.500, 0.500) | 0.433 (0.371, 0.495) | 0.719 | 0.504 | 0.500 (±0.0000) | 0.499 (±0.0000) | \| | \| |  |  |  |
| │ │ └── CliXO:366 (TRIP13, HMMR, MELK) | 0.500 (0.500, 0.500) | 0.433 (0.371, 0.495) | 0.719 | 0.504 | 0.502 (±0.0055) | 0.375 (±0.0009) | \| | \| |  |  |  |
| │ ├── CliXO:391 (TMTC4, HMGN3, ACTN1, MIIP, PPP3CA, SDC4, SLC6A8, HK1, RASEF, GOLM1, HNMT, ARID3A, MT1F, PLOD2, PFKFB4, HMGCR, ACSF2, DLX5, PCYOX1, ERLIN2, CKS2, C1orf21, MME, SLC44A3, SLC24A3, IDE) | 0.500 (0.500, 0.500) | 0.433 (0.371, 0.495) | 0.719 | 0.504 | 0.502 (±0.0055) | 0.375 (±0.0009) | \| | \| |  |  |  |
| │ │ ├── CliXO:363 (C1orf21, SLC44A3, SLC24A3) | 0.500 (0.500, 0.500) | 0.433 (0.371, 0.495) | 0.719 | 0.504 | 0.502 (±0.0055) | 0.375 (±0.0009) | \| | \| |  |  |  |
| │ │ ├── CliXO:370 (TMTC4, RASEF, GOLM1, MT1F) | 0.500 (0.500, 0.500) | 0.433 (0.371, 0.495) | 0.719 | 0.504 | 0.502 (±0.0055) | 0.375 (±0.0009) | \| | \| |  |  |  |
| │ │ ├── CliXO:373 (TMTC4, GOLM1, HNMT, MT1F, PCYOX1) | 0.500 (0.500, 0.500) | 0.433 (0.371, 0.495) | 0.719 | 0.504 | 0.502 (±0.0055) | 0.375 (±0.0009) | \| | \| |  |  |  |
| │ │ └── CliXO:385 (HMGN3, ACTN1, MIIP, PPP3CA, SDC4, SLC6A8, HK1, ARID3A, PLOD2, PFKFB4, ACSF2, CKS2, MME) | 0.500 (0.500, 0.500) | 0.433 (0.371, 0.495) | 0.719 | 0.504 | 0.502 (±0.0055) | 0.375 (±0.0009) | \| | \| |  |  |  |
| │ └── CliXO:393 (TMTC4, WDSUB1, SMPD2, RASEF, GOLM1, HNMT, GRINA, SESN3, NIPAL3, TMEM106B, VEGFA, MT1F, NCEH1, NUCB2, DHX29, HMGCR, DLX5, CCDC113, PCYOX1, G3BP2, TMEM141, SACM1L, ERLIN2, CTSA, C1orf21, SLC44A3, LDLR, SLC24A3, TLCD1, HSPA9, GGCX, GPT2, IDE, PREP, VCP, TMEM154, DOCK8) | 0.500 (0.500, 0.500) | 0.433 (0.371, 0.495) | 0.719 | 0.504 | 0.505 (±0.0015) | 0.533 (±0.0002) | \| | \| |  |  |  |
| │ │ ├── CliXO:370 (TMTC4, RASEF, GOLM1, MT1F) | 0.500 (0.500, 0.500) | 0.433 (0.371, 0.495) | 0.719 | 0.504 | 0.502 (±0.0055) | 0.375 (±0.0009) | \| | \| |  |  |  |
| │ │ ├── CliXO:373 (TMTC4, GOLM1, HNMT, MT1F, PCYOX1) | 0.500 (0.500, 0.500) | 0.433 (0.371, 0.495) | 0.719 | 0.504 | 0.502 (±0.0055) | 0.375 (±0.0009) | \| | \| |  |  |  |
| │ └── CliXO:383 (RASEF, GOLM1, HNMT, PCYOX1, G3BP2, CTSA, C1orf21, SLC44A3, SLC24A3, IDE, DOCK8) | 0.500 (0.500, 0.500) | 0.433 (0.371, 0.495) | 0.719 | 0.504 | 0.502 (±0.0055) | 0.375 (±0.0009) | \| | \| |  |  |  |
| │ └── CliXO:381 (RASEF, GOLM1, HNMT, G3BP2, CTSA, C1orf21, SLC44A3, SLC24A3, IDE) | 0.500 (0.500, 0.500) | 0.433 (0.371, 0.495) | 0.719 | 0.504 | 0.502 (±0.0055) | 0.375 (±0.0009) | \| | \| |  |  |  |
| │ │ ├── CliXO:363 (C1orf21, SLC44A3, SLC24A3) | 0.500 (0.500, 0.500) | 0.433 (0.371, 0.495) | 0.719 | 0.504 | 0.502 (±0.0055) | 0.375 (±0.0009) | \| | \| |  |  |  |
| │ └── CliXO:365 (CTSA, SLC44A3, SLC24A3) | 0.500 (0.500, 0.500) | 0.433 (0.371, 0.495) | 0.719 | 0.504 | 0.502 (±0.0055) | 0.375 (±0.0009) | \| | \| |  |  |  |
| └── CliXO:417 (CX3CR1, SLIT3, CEBPD, MYO5A, SLC2A1, GNG11, DNAJC1, AKAP13, SCARB1, ZBTB20†, PTPN12, IGF1, KIAA0232†, CBX5, DYRK2, ETS2, MEF2A, GPER1, ZNF462, IFI44L, CYTL1, GSN, PARM1, NFKB1, RAB3B, SEMA3G, ST6GAL1, MCTP2, SYK, NFIA, TXNDC11, SMIM3, LRRC8A, JAK2, CDK14, MS4A4A, LAPTM5, JUNB, F8, RIN2, CLIC5, CARD16, PRKCE, KIF5B, CCDC59, TBXAS1, DDIT4, GNB1, EXOC6, CCND1, TMTC4, SAV1, NECTIN4, PPP2R5A, KMT2E, FZD1, CYBB, DPYD, ELMO1, SNAPC1, C3AR1, TSPAN33, HMGN3, MIDN, LTF, RIMKLA, TRIP10, CLOCK, TACC1, FLI1, MBTPS2, MXI1, RNASE1, MAML2, SYPL1, AMOTL2, DDR2, ACTN1, TDP1, MIIP, TANK, ARRDC3, YPEL2, GALNT18, TYROBP, DUSP1, ZNF281, LAMA4, EFHD1, EDEM1, SBF2, TEX2, THOP1, PPP3CA, TLR7, THRA, LRRC32, HSPA2, PIK3AP1, TSPAN14, ZFYVE1, MRI1, CHIC2, MLLT1, COL6A1, SLC9A9, MINPP1, DIO2, SULF2, ASTE1, PLCB1, RNF11, CMKLR1, ZNF331, PODXL, SDC4, WDSUB1, PDGFC, SMPD2, SLC26A6, PGRMC1, SLC6A8, GUCA2A, TLR4, MXRA5, MAP3K9, BCORL1, HK1, RCBTB2, ASAP2, RALGPS1, ADCY7, TMCC3, ANGPT1, ARL6IP5, RASAL2, ARTN, RASEF, ANKDD1A, AVL9, SYDE1, MATN2, ILF2, GCLM, CTBS, GOLM1, PROM1, PM20D2, EBF3, SH2B3, ATP10D, IL33, ERRFI1, HNMT, DKK3†, ELL2, GRINA, PDE5A†, MAP4K4, SESN3, NMNAT2, BRAF, INSL4, NIPAL3†, TRIB2, ARID3A, KDM1A, TMEM106B, VEGFA, CNNM4, WDR11, MT1F†, CREBL2, PKM, CDO1, NUDT5, HEY1, PEBP1, SLC38A1, LHFPL2, NCEH1, SEMA4C, ZMYND11, PLOD2, TRPC6, FANCE, VEGFC, PMAIP1, SHANK2, PFKFB4, ACVRL1, SLC35B3, GALK1, GBP4, EDN1, NUCB2, DHX29, ADARB1, OLFML2B, CPOX, PAPSS1, GPR107, HMGCR, MIOS, ACSF2, DLX5, CCDC113, GAN, TMEM176B, FCN3, ARHGAP24, CDS1, KIFC3, XPO4, LRCH1, PCYOX1, INTS7, AZIN1, SPG21, APLN, WNK2, G3BP2, FGF13†, SLCO2B1, LIMCH1, AGTR1, FBN2, SARAF, CNOT6L, TRIP13, YTHDC2, ZNF512B, TMEM141, TAPT1, SACM1L, INSIG1, RNF20, BRCA1, ERLIN2, CADM3, AMD1, GLA, PUS7, CKS2, ROBO4, AK2, PRKAB2, BTD, HMMR, PLBD1, LEPROTL1, WDFY2, FLVCR2, PHKA2, PDP2, DCXR, SLC38A6, SLC39A8, NPL, SLC11A1, PTPRJ, C1orf21, MME, DUSP4, MXD1, KLHL3, RCAN3, APH1B, FOLR1, DUS2, SLC44A3, TMEM43, PIK3CB, ABLIM3, GRHL1, DAB2, LDLR, SIPA1L1, SLC24A3, MAT2A, MKNK1, SLC16A3, GRHL3, EPS8L1, HGF, TP53BP2, LSM1, MOB3C, NHLRC2, CDYL2, KIF2A, MAN1A2, PAPPA, NPEPPS, INPP5F, NEK7, TLCD1, RASSF8, TMEM268, CSNK2A2, CCSAP, KIF17, ZNF75A, KLHL42, PKD2, NIN†, PPP2R1B, HSPA9, GGCX, CYP19A1, GPT2†, SH3RF1, EZH2, TKFC, LURAP1L, CHEK1, SPON1, SCMH1, PLAG1, STX6, EPB41L5, DONSON, IDE, PDZD2, GEMIN5, PRKCH, RAD51, MAFF, GPD1L, TOP2B, PREP, ARHGAP26, FNDC3A, STAT5B, CLTC, GADD45G, ODF2, VCP, RHOBTB3, TMEM154, DOCK8, MELK, SMARCA2, TTI1, MTMR4, ETNK2, ARHGAP28, ESRP1, HJURP, UBE2H) | 0.724 (0.704, 0.743) | 0.376 (0.322, 0.430) | 0.746 | 0.004 | 0.490 (±0.0044) | 0.390 (±0.0009) | \|✓ | \|✓ | ✓ |  | CliXO:417 |
| ├── CliXO:412 (MYO5A, DNAJC1, CBX5, ZNF462, GSN, TXNDC11, JUNB, CCDC59, GNB1, TMTC4, KMT2E, SNAPC1, HMGN3, MXI1, AMOTL2, ACTN1, TDP1, MIIP, TYROBP, EDEM1, THOP1, PPP3CA, COL6A1, SLC9A9, DIO2, SDC4, WDSUB1, SMPD2, PGRMC1, SLC6A8, MAP3K9, HK1, RALGPS1, ADCY7, RASEF, ANKDD1A, AVL9, ILF2, GCLM, CTBS, GOLM1, PM20D2, ATP10D, ERRFI1, HNMT, GRINA, SESN3, NMNAT2, BRAF, NIPAL3, ARID3A, KDM1A, TMEM106B, VEGFA, CNNM4, WDR11, MT1F, PKM, CDO1, NUDT5, PEBP1, SLC38A1, LHFPL2, NCEH1, SEMA4C, PLOD2, FANCE, PMAIP1, SHANK2, PFKFB4, GALK1, CPOX, PAPSS1, GPR107, HMGCR, ACSF2, DLX5, CDS1, XPO4, PCYOX1, AZIN1, WNK2, G3BP2, FBN2, TRIP13, YTHDC2, ZNF512B, TMEM141, SACM1L, BRCA1, ERLIN2, GLA, PUS7, CKS2, AK2, PRKAB2, HMMR, PLBD1, WDFY2, FLVCR2, PHKA2, PDP2, DCXR, SLC38A6, SLC39A8, NPL, SLC11A1, C1orf21, MME, KLHL3, RCAN3, APH1B, FOLR1, DUS2, SLC44A3, PIK3CB, GRHL1, DAB2, LDLR, SIPA1L1, SLC24A3, MAT2A, MKNK1, SLC16A3, GRHL3, HGF, TP53BP2, LSM1, NHLRC2, NPEPPS, INPP5F, TLCD1, RASSF8, TMEM268, CSNK2A2, ZNF75A, KLHL42, PPP2R1B, HSPA9, GGCX, GPT2, SH3RF1, EZH2, TKFC, CHEK1, SPON1, SCMH1, PLAG1, STX6, EPB41L5, IDE, GEMIN5, RAD51, GPD1L, TOP2B, PREP, CLTC, ODF2, VCP, TMEM154, DOCK8, MELK, SMARCA2, TTI1, ETNK2, ARHGAP28, ESRP1, HJURP, UBE2H) | 0.728 (0.708, 0.749) | 0.376 (0.322, 0.430) | 0.768 | 0.004 | 0.568 (±0.0086) | 0.717 (±0.0008) | \| | \| |  |  |  |
| │ ├── CliXO:390 (FANCE, SHANK2, TRIP13, HMMR, PLBD1, PHKA2, DCXR, PIK3CB, GRHL1, SIPA1L1, SLC16A3, TP53BP2, INPP5F, TLCD1, PPP2R1B, GPT2, CHEK1, PLAG1, RAD51, TOP2B, MELK, ESRP1, HJURP) | 0.500 (0.500, 0.500) | 0.433 (0.371, 0.495) | 0.719 | 0.504 | 0.500 (±0.0000) | 0.499 (±0.0000) | \| | \| |  |  |  |
| │ │ └── CliXO:366 (TRIP13, HMMR, MELK) | 0.500 (0.500, 0.500) | 0.433 (0.371, 0.495) | 0.719 | 0.504 | 0.502 (±0.0055) | 0.375 (±0.0009) | \| | \| |  |  |  |
| │ ├── CliXO:396 (PEBP1, SEMA4C, SHANK2, PFKFB4, HMGCR, ACSF2, XPO4, TRIP13, YTHDC2, BRCA1, ERLIN2, HMMR, PLBD1, WDFY2, PHKA2, DCXR, C1orf21, MME, APH1B, PIK3CB, SIPA1L1, SLC24A3, MAT2A, SLC16A3, HGF, TP53BP2, LSM1, NPEPPS, INPP5F, TLCD1, ZNF75A, KLHL42, PPP2R1B, GPT2, EZH2, CHEK1, SPON1, STX6, EPB41L5, IDE, RAD51, GPD1L, TOP2B, CLTC, ODF2, VCP, MELK, SMARCA2, TTI1, ETNK2, ARHGAP28, ESRP1, HJURP, UBE2H) | 0.500 (0.500, 0.500) | 0.433 (0.371, 0.495) | 0.719 | 0.504 | 0.500 (±0.0000) | 0.499 (±0.0000) | \| | \| |  |  |  |
| │ │ └── CliXO:366 (TRIP13, HMMR, MELK) | 0.500 (0.500, 0.500) | 0.433 (0.371, 0.495) | 0.719 | 0.504 | 0.502 (±0.0055) | 0.375 (±0.0009) | \| | \| |  |  |  |
| │ ├── CliXO:380 (PEBP1, HMGCR, TRIP13, ERLIN2, C1orf21, SLC24A3, PPP2R1B, GPD1L) | 0.500 (0.500, 0.500) | 0.433 (0.371, 0.495) | 0.719 | 0.504 | 0.502 (±0.0055) | 0.375 (±0.0009) | \| | \| |  |  |  |
| │ │ └── CliXO:389 (PHKA2, HGF, LSM1, ZNF75A, KLHL42, PPP2R1B, EZH2, CHEK1, SPON1, EPB41L5, RAD51, GPD1L, TOP2B, CLTC, ODF2, VCP, MELK, SMARCA2, ETNK2, ARHGAP28, ESRP1, UBE2H) | 0.500 (0.500, 0.500) | 0.433 (0.371, 0.495) | 0.719 | 0.504 | 0.502 (±0.0055) | 0.375 (±0.0009) | \| | \| |  |  |  |
| │ └── CliXO:405 (GSN, TMTC4, HMGN3, MXI1, ACTN1, MIIP, PPP3CA, COL6A1, DIO2, SDC4, SMPD2, PGRMC1, SLC6A8, HK1, RALGPS1, RASEF, ANKDD1A, GCLM, GOLM1, PM20D2, ATP10D, HNMT, GRINA, SESN3, NMNAT2, BRAF, NIPAL3, ARID3A, KDM1A, TMEM106B, VEGFA, CNNM4, MT1F, PKM, NUDT5, PEBP1, LHFPL2, NCEH1, SEMA4C, PLOD2, FANCE, PMAIP1, SHANK2, PFKFB4, GALK1, PAPSS1, GPR107, HMGCR, ACSF2, DLX5, CDS1, PCYOX1, AZIN1, G3BP2, FBN2, TRIP13, ZNF512B, TMEM141, ERLIN2, PUS7, CKS2, PRKAB2, HMMR, PLBD1, PHKA2, DCXR, SLC38A6, SLC39A8, NPL, C1orf21, MME, KLHL3, RCAN3, FOLR1, DUS2, SLC44A3, PIK3CB, LDLR, SIPA1L1, SLC24A3, MAT2A, MKNK1, SLC16A3, GRHL3, HGF, TP53BP2, NHLRC2, NPEPPS, INPP5F, RASSF8, TMEM268, PPP2R1B, GGCX, GPT2, SH3RF1, TKFC, SPON1, SCMH1, PLAG1, STX6, EPB41L5, IDE, GEMIN5, RAD51, GPD1L, TOP2B, PREP, CLTC, ODF2, VCP, TMEM154, DOCK8, TTI1, ETNK2, ARHGAP28, ESRP1, HJURP) | 0.272 (0.251, 0.292) | 0.491 (0.421, 0.561) | 0.395 | 0.504 | 0.503 (±0.0051) | 0.390 (±0.0008) | \| | \| |  |  | CliXO:417 |
| │ ├── CliXO:354 (DIO2, PGRMC1) | 0.500 (0.500, 0.500) | 0.433 (0.371, 0.495) | 0.719 | 0.504 | 0.502 (±0.0055) | 0.375 (±0.0009) | \| | \| |  |  |  |
| │ ├── CliXO:360 (PEBP1, LDLR) | 0.500 (0.500, 0.500) | 0.433 (0.371, 0.495) | 0.719 | 0.504 | 0.502 (±0.0055) | 0.375 (±0.0009) | \| | \| |  |  |  |
| │ ├── CliXO:368 (FANCE, SLC16A3, INPP5F, RASSF8) | 0.500 (0.500, 0.500) | 0.433 (0.371, 0.495) | 0.719 | 0.504 | 0.502 (±0.0055) | 0.375 (±0.0009) | \| | \| |  |  |  |
| │ ├── CliXO:378 (GCLM, NMNAT2, TMEM106B, LHFPL2, PMAIP1, CDS1, RCAN3) | 0.500 (0.500, 0.500) | 0.433 (0.371, 0.495) | 0.719 | 0.504 | 0.502 (±0.0055) | 0.375 (±0.0009) | \| | \| |  |  |  |
| │ │ └── CliXO:359 (CDS1, RCAN3) | 0.500 (0.500, 0.500) | 0.433 (0.371, 0.495) | 0.719 | 0.504 | 0.502 (±0.0055) | 0.375 (±0.0009) | \| | \| |  |  |  |
| │ ├── CliXO:380 (PEBP1, HMGCR, TRIP13, ERLIN2, C1orf21, SLC24A3, PPP2R1B, GPD1L) | 0.500 (0.500, 0.500) | 0.433 (0.371, 0.495) | 0.719 | 0.504 | 0.502 (±0.0055) | 0.375 (±0.0009) | \| | \| |  |  |  |
| │ ├── CliXO:382 (ACSF2, TRIP13, ZNF512B, DCXR, MME, HGF, NPEPPS, STX6, EPB41L5) | 0.500 (0.500, 0.500) | 0.433 (0.371, 0.495) | 0.719 | 0.504 | 0.502 (±0.0055) | 0.375 (±0.0009) | \| | \| |  |  |  |
| │ └── CliXO:395 (TMTC4, HMGN3, ACTN1, MIIP, PPP3CA, COL6A1, SDC4, PGRMC1, SLC6A8, HK1, RASEF, GOLM1, HNMT, NIPAL3, ARID3A, VEGFA, MT1F, PKM, NUDT5, SEMA4C, PLOD2, PFKFB4, GALK1, PAPSS1, HMGCR, ACSF2, DLX5, PCYOX1, G3BP2, ZNF512B, ERLIN2, PUS7, CKS2, DCXR, C1orf21, MME, FOLR1, SLC44A3, SLC24A3, GRHL3, NPEPPS, SPON1, SCMH1, EPB41L5, IDE, DOCK8) | 0.728 (0.708, 0.749) | 0.376 (0.322, 0.430) | 0.768 | 0.004 | 0.497 (±0.0045) | 0.397 (±0.0007) | \|✓ | \|✓ |  |  | CliXO:410 |
| │ ├── CliXO:367 (NIPAL3, MT1F, PAPSS1) | 0.500 (0.500, 0.500) | 0.433 (0.371, 0.495) | 0.719 | 0.504 | 0.500 (±0.0049) | 0.390 (±0.0008) | \| | \| |  |  | CliXO:410 |
| │ ├── CliXO:374 (HK1, NIPAL3, VEGFA, PKM, GALK1) | 0.500 (0.500, 0.500) | 0.433 (0.371, 0.495) | 0.719 | 0.504 | 0.504 (±0.0013) | 0.530 (±0.0002) | \| | \| |  |  |  |
| │ │ └── CliXO:358 (VEGFA, GALK1) | 0.500 (0.500, 0.500) | 0.433 (0.371, 0.495) | 0.719 | 0.504 | 0.502 (±0.0055) | 0.375 (±0.0009) | \| | \| |  |  |  |
| │ ├── CliXO:376 (TMTC4, HMGN3, ACTN1, PPP3CA, ARID3A, PUS7) | 0.500 (0.500, 0.500) | 0.433 (0.371, 0.495) | 0.719 | 0.504 | 0.502 (±0.0055) | 0.375 (±0.0009) | \| | \| |  |  |  |
| │ ├── CliXO:379 (HMGN3, PPP3CA, COL6A1, ZNF512B, CKS2, SPON1, EPB41L5) | 0.500 (0.500, 0.500) | 0.433 (0.371, 0.495) | 0.719 | 0.504 | 0.502 (±0.0055) | 0.375 (±0.0009) | \| | \| |  |  |  |
| │ │ └── CliXO:357 (COL6A1, CKS2) | 0.500 (0.500, 0.500) | 0.433 (0.371, 0.495) | 0.719 | 0.504 | 0.502 (±0.0055) | 0.375 (±0.0009) | \| | \| |  |  |  |
| │ ├── CliXO:391 (TMTC4, HMGN3, ACTN1, MIIP, PPP3CA, SDC4, SLC6A8, HK1, RASEF, GOLM1, HNMT, ARID3A, MT1F, PLOD2, PFKFB4, HMGCR, ACSF2, DLX5, PCYOX1, ERLIN2, CKS2, C1orf21, MME, SLC44A3, SLC24A3, IDE) | 0.500 (0.500, 0.500) | 0.433 (0.371, 0.495) | 0.719 | 0.504 | 0.502 (±0.0055) | 0.375 (±0.0009) | \| | \| |  |  |  |
| │ │ ├── CliXO:363 (C1orf21, SLC44A3, SLC24A3) | 0.500 (0.500, 0.500) | 0.433 (0.371, 0.495) | 0.719 | 0.504 | 0.502 (±0.0055) | 0.375 (±0.0009) | \| | \| |  |  |  |
| │ │ ├── CliXO:370 (TMTC4, RASEF, GOLM1, MT1F) | 0.500 (0.500, 0.500) | 0.433 (0.371, 0.495) | 0.719 | 0.504 | 0.502 (±0.0055) | 0.375 (±0.0009) | \| | \| |  |  |  |
| │ │ ├── CliXO:373 (TMTC4, GOLM1, HNMT, MT1F, PCYOX1) | 0.500 (0.500, 0.500) | 0.433 (0.371, 0.495) | 0.719 | 0.504 | 0.502 (±0.0055) | 0.375 (±0.0009) | \| | \| |  |  |  |
| │ │ └── CliXO:385 (HMGN3, ACTN1, MIIP, PPP3CA, SDC4, SLC6A8, HK1, ARID3A, PLOD2, PFKFB4, ACSF2, CKS2, MME) | 0.500 (0.500, 0.500) | 0.433 (0.371, 0.495) | 0.719 | 0.504 | 0.502 (±0.0055) | 0.375 (±0.0009) | \| | \| |  |  |  |
| └── CliXO:416 (CX3CR1, SLIT3, CEBPD, MYO5A, SLC2A1, GNG11, DNAJC1, AKAP13, SCARB1, ZBTB20, PTPN12, IGF1, KIAA0232, CBX5, DYRK2, ETS2, MEF2A, GPER1, ZNF462, IFI44L, CYTL1, GSN, PARM1, NFKB1, RAB3B, SEMA3G, ST6GAL1, MCTP2, SYK, NFIA, TXNDC11, SMIM3, LRRC8A, JAK2, CDK14, MS4A4A, LAPTM5, JUNB, F8, RIN2, CLIC5, CARD16, PRKCE, KIF5B, CCDC59, TBXAS1, DDIT4, GNB1, EXOC6, CCND1, TMTC4, SAV1, NECTIN4, PPP2R5A, KMT2E, FZD1, CYBB, DPYD, ELMO1, SNAPC1, C3AR1, TSPAN33, HMGN3, MIDN, LTF, RIMKLA, TRIP10, CLOCK, TACC1, FLI1, MBTPS2, MXI1, RNASE1, MAML2, SYPL1, AMOTL2, DDR2, ACTN1, TDP1, TANK, ARRDC3, YPEL2, GALNT18, TYROBP, DUSP1, ZNF281, LAMA4, EFHD1, EDEM1, SBF2, TEX2, THOP1, PPP3CA, TLR7, THRA, LRRC32, HSPA2, PIK3AP1, TSPAN14, ZFYVE1, MRI1, CHIC2, MLLT1, SLC9A9, MINPP1, DIO2, SULF2, ASTE1, PLCB1, RNF11, CMKLR1, ZNF331, PODXL, SDC4, WDSUB1, PDGFC, SMPD2, SLC26A6, PGRMC1, SLC6A8, GUCA2A, TLR4, MXRA5, MAP3K9, BCORL1, HK1, RCBTB2, ASAP2, RALGPS1, ADCY7, TMCC3, ANGPT1, ARL6IP5, RASAL2, ARTN, ANKDD1A, AVL9, SYDE1, MATN2, ILF2, GCLM, CTBS, GOLM1, PROM1, PM20D2, EBF3, SH2B3, ATP10D, IL33, ERRFI1, HNMT, DKK3, ELL2, GRINA, PDE5A, MAP4K4, SESN3, NMNAT2, BRAF, INSL4, NIPAL3, TRIB2, ARID3A, KDM1A, TMEM106B, VEGFA, CNNM4, WDR11, MT1F, CREBL2, PKM, CDO1, NUDT5, HEY1, PEBP1, SLC38A1, LHFPL2, NCEH1, SEMA4C, ZMYND11, PLOD2, TRPC6, FANCE, VEGFC, SHANK2, PFKFB4, ACVRL1, SLC35B3, GBP4, EDN1, NUCB2, DHX29, ADARB1, OLFML2B, CPOX, PAPSS1, GPR107, HMGCR, MIOS, ACSF2, DLX5, CCDC113, GAN, TMEM176B, FCN3, ARHGAP24, CDS1, KIFC3, XPO4, LRCH1, PCYOX1, INTS7, AZIN1, SPG21, APLN, WNK2, G3BP2, FGF13, SLCO2B1, LIMCH1, AGTR1, FBN2, SARAF, CNOT6L, TRIP13, YTHDC2, ZNF512B, TMEM141, TAPT1, SACM1L, INSIG1, RNF20, BRCA1, ERLIN2, CADM3, AMD1, GLA, PUS7, CKS2, ROBO4, AK2, PRKAB2, BTD, HMMR, PLBD1, LEPROTL1, WDFY2, FLVCR2, PHKA2, PDP2, DCXR, SLC38A6, SLC39A8, NPL, SLC11A1, PTPRJ, C1orf21, MME, DUSP4, MXD1, KLHL3, RCAN3, APH1B, DUS2, TMEM43, PIK3CB, ABLIM3, GRHL1, DAB2, LDLR, SIPA1L1, SLC24A3, MAT2A, MKNK1, SLC16A3, GRHL3, EPS8L1, HGF, TP53BP2, LSM1, MOB3C, NHLRC2, CDYL2, KIF2A, MAN1A2, PAPPA, NPEPPS, INPP5F, NEK7, TLCD1, TMEM268, CSNK2A2, CCSAP, KIF17, ZNF75A, KLHL42, PKD2, NIN, PPP2R1B, HSPA9, GGCX, CYP19A1, GPT2, SH3RF1, EZH2, TKFC, LURAP1L, CHEK1, SPON1, SCMH1, PLAG1, STX6, EPB41L5, DONSON, IDE, PDZD2, GEMIN5, PRKCH, RAD51, MAFF, GPD1L, TOP2B, PREP, ARHGAP26, FNDC3A, STAT5B, CLTC, GADD45G, ODF2, VCP, RHOBTB3, TMEM154, DOCK8, MELK, SMARCA2, TTI1, MTMR4, ETNK2, ARHGAP28, ESRP1, HJURP, UBE2H) | 0.716 (0.697, 0.735) | 0.494 (0.424, 0.565) | 0.763 | 0.504 | 0.569 (±0.0089) | 0.738 (±0.0009) | \| | \| |  |  |  |
| ├── CliXO:407 (CX3CR1, SLIT3, CEBPD, MYO5A, SLC2A1, GNG11, DNAJC1, AKAP13, SCARB1, IGF1, CBX5, DYRK2, MEF2A, GPER1, ZNF462, IFI44L, CYTL1, GSN, NFKB1, RAB3B, SEMA3G, MCTP2, NFIA, TXNDC11, SMIM3, CDK14, JUNB, RIN2, CLIC5, KIF5B, CCDC59, TBXAS1, DDIT4, GNB1, EXOC6, CCND1, SAV1, NECTIN4, KMT2E, FZD1, SNAPC1, TSPAN33, MIDN, LTF, RIMKLA, TRIP10, TACC1, FLI1, MXI1, RNASE1, MAML2, SYPL1, AMOTL2, TDP1, TANK, YPEL2, GALNT18, TYROBP, DUSP1, ZNF281, LAMA4, EFHD1, SBF2, TEX2, TLR7, THRA, LRRC32, HSPA2, PIK3AP1, TSPAN14, ZFYVE1, MRI1, CHIC2, MLLT1, SLC9A9, ASTE1, PLCB1, CMKLR1, ZNF331, PODXL, GUCA2A, MXRA5, BCORL1, RCBTB2, ASAP2, TMCC3, ANGPT1, ARL6IP5, RASAL2, ARTN, SYDE1, MATN2, PROM1, EBF3, SH2B3, IL33, ERRFI1, DKK3, PDE5A, MAP4K4, TRIB2, HEY1, ZMYND11, TRPC6, ACVRL1, SLC35B3, GBP4, EDN1, ADARB1, OLFML2B, TMEM176B, FCN3, ARHGAP24, KIFC3, LRCH1, APLN, FGF13, LIMCH1, AGTR1, SARAF, CNOT6L, TAPT1, INSIG1, RNF20, AMD1, ROBO4, LEPROTL1, MXD1, TMEM43, ABLIM3, EPS8L1, MOB3C, KIF2A, CCSAP, KIF17, LURAP1L, ARHGAP26, MTMR4) | 0.500 (0.500, 0.500) | 0.433 (0.371, 0.495) | 0.719 | 0.504 | 0.502 (±0.0058) | 0.370 (±0.0010) | \| | \| |  |  |  |
| │ ├── CliXO:356 (TMCC3, ADARB1) | 0.500 (0.500, 0.500) | 0.433 (0.371, 0.495) | 0.719 | 0.504 | 0.502 (±0.0055) | 0.375 (±0.0009) | \| | \| |  |  |  |
| │ ├── CliXO:372 (SLC2A1, ZNF331, ANGPT1, SYDE1, ARHGAP24) | 0.500 (0.500, 0.500) | 0.433 (0.371, 0.495) | 0.719 | 0.504 | 0.502 (±0.0055) | 0.375 (±0.0009) | \| | \| |  |  |  |
| │ ├── CliXO:384 (SLC2A1, SCARB1, SMIM3, NECTIN4, SYPL1, EFHD1, PIK3AP1, ARTN, ERRFI1, KIF2A, CCSAP) | 0.500 (0.500, 0.500) | 0.433 (0.371, 0.495) | 0.719 | 0.504 | 0.502 (±0.0055) | 0.375 (±0.0009) | \| | \| |  |  |  |
| │ ├── CliXO:362 (SLC2A1, NECTIN4) | 0.500 (0.500, 0.500) | 0.433 (0.371, 0.495) | 0.719 | 0.504 | 0.502 (±0.0055) | 0.375 (±0.0009) | \| | \| |  |  |  |
| │ │ └── CliXO:369 (SMIM3, SYPL1, EFHD1, ERRFI1) | 0.500 (0.500, 0.500) | 0.433 (0.371, 0.495) | 0.719 | 0.504 | 0.502 (±0.0055) | 0.375 (±0.0009) | \| | \| |  |  |  |
| │ ├── CliXO:387 (CYTL1, SEMA3G, GNB1, YPEL2, SBF2, LRRC32, HSPA2, MXRA5, ANGPT1, EBF3, PDE5A, ACVRL1, ARHGAP24, KIFC3) | 0.500 (0.500, 0.500) | 0.433 (0.371, 0.495) | 0.719 | 0.504 | 0.502 (±0.0055) | 0.375 (±0.0009) | \| | \| |  |  |  |
| │ └── CliXO:401 (CX3CR1, SLIT3, CEBPD, MYO5A, GNG11, DNAJC1, CBX5, DYRK2, GPER1, ZNF462, CYTL1, NFKB1, SEMA3G, NFIA, TXNDC11, CDK14, JUNB, RIN2, KIF5B, CCDC59, TBXAS1, GNB1, CCND1, KMT2E, FZD1, SNAPC1, TSPAN33, MIDN, LTF, RIMKLA, TACC1, FLI1, MXI1, AMOTL2, TDP1, YPEL2, GALNT18, TYROBP, DUSP1, ZNF281, TEX2, THRA, LRRC32, HSPA2, ZFYVE1, MRI1, CHIC2, MLLT1, PLCB1, GUCA2A, MXRA5, RCBTB2, ASAP2, ANGPT1, ARL6IP5, RASAL2, MATN2, PROM1, EBF3, SH2B3, IL33, DKK3, PDE5A, MAP4K4, TRIB2, HEY1, ZMYND11, TRPC6, GBP4, EDN1, OLFML2B, TMEM176B, ARHGAP24, KIFC3, LRCH1, FGF13, AGTR1, CNOT6L, TAPT1, ROBO4, ABLIM3, LURAP1L) | 0.500 (0.500, 0.500) | 0.433 (0.371, 0.495) | 0.719 | 0.504 | 0.497 (±0.0025) | 0.444 (±0.0004) | \| | \| |  |  |  |
| │ └── CliXO:392 (GNG11, CYTL1, SEMA3G, TXNDC11, JUNB, TBXAS1, CCND1, FZD1, LTF, TACC1, FLI1, MXI1, YPEL2, DUSP1, TEX2, THRA, ZFYVE1, CHIC2, PLCB1, GUCA2A, RCBTB2, ARL6IP5, PROM1, SH2B3, IL33, DKK3, MAP4K4, ZMYND11, GBP4, EDN1, OLFML2B, TMEM176B, ROBO4, LURAP1L) | 0.500 (0.500, 0.500) | 0.433 (0.371, 0.495) | 0.719 | 0.504 | 0.502 (±0.0055) | 0.375 (±0.0009) | \| | \| |  |  |  |
| │ └── CliXO:371 (CYTL1, FZD1, TACC1, THRA) | 0.500 (0.500, 0.500) | 0.433 (0.371, 0.495) | 0.719 | 0.504 | 0.502 (±0.0055) | 0.375 (±0.0009) | \| | \| |  |  |  |
| ├── CliXO:409 (TMTC4, ARRDC3, EDEM1, THOP1, WDSUB1, SMPD2, SLC26A6, MAP3K9, RALGPS1, ADCY7, ANKDD1A, AVL9, ILF2, GCLM, CTBS, GOLM1, PM20D2, ATP10D, ELL2, GRINA, SESN3, NMNAT2, BRAF, INSL4, TMEM106B, CNNM4, WDR11, CDO1, NUDT5, PEBP1, SLC38A1, LHFPL2, NCEH1, FANCE, SHANK2, NUCB2, DHX29, CPOX, GPR107, HMGCR, MIOS, CCDC113, GAN, CDS1, XPO4, PCYOX1, INTS7, AZIN1, SPG21, WNK2, G3BP2, FBN2, TRIP13, YTHDC2, ZNF512B, TMEM141, SACM1L, BRCA1, ERLIN2, GLA, AK2, PRKAB2, HMMR, PLBD1, WDFY2, FLVCR2, PHKA2, PDP2, DCXR, SLC38A6, SLC39A8, NPL, SLC11A1, PTPRJ, C1orf21, MME, DUSP4, KLHL3, RCAN3, APH1B, DUS2, PIK3CB, GRHL1, DAB2, LDLR, SIPA1L1, SLC24A3, MAT2A, MKNK1, SLC16A3, GRHL3, HGF, TP53BP2, LSM1, NHLRC2, MAN1A2, PAPPA, NPEPPS, INPP5F, NEK7, TLCD1, TMEM268, CSNK2A2, KIF17, ZNF75A, KLHL42, PKD2, NIN, PPP2R1B, HSPA9, GGCX, CYP19A1, GPT2, SH3RF1, EZH2, TKFC, CHEK1, SPON1, SCMH1, PLAG1, STX6, EPB41L5, DONSON, IDE, PDZD2, GEMIN5, RAD51, MAFF, GPD1L, TOP2B, PREP, ARHGAP26, FNDC3A, STAT5B, CLTC, GADD45G, ODF2, VCP, RHOBTB3, TMEM154, DOCK8, MELK, SMARCA2, ETNK2, ARHGAP28, ESRP1, HJURP, UBE2H) | 0.084 (0.070, 0.097) | 0.433 (0.371, 0.495) | 0.219 | 0.504 | 0.501 (±0.0024) | 0.443 (±0.0004) | \| | \| |  |  |  |
| │ ├── CliXO:380 (PEBP1, HMGCR, TRIP13, ERLIN2, C1orf21, SLC24A3, PPP2R1B, GPD1L) | 0.500 (0.500, 0.500) | 0.433 (0.371, 0.495) | 0.719 | 0.504 | 0.502 (±0.0055) | 0.375 (±0.0009) | \| | \| |  |  |  |
| │ │ └── CliXO:389 (PHKA2, HGF, LSM1, ZNF75A, KLHL42, PPP2R1B, EZH2, CHEK1, SPON1, EPB41L5, RAD51, GPD1L, TOP2B, CLTC, ODF2, VCP, MELK, SMARCA2, ETNK2, ARHGAP28, ESRP1, UBE2H) | 0.500 (0.500, 0.500) | 0.433 (0.371, 0.495) | 0.719 | 0.504 | 0.502 (±0.0055) | 0.375 (±0.0009) | \| | \| |  |  |  |
| │ ├── CliXO:390 (FANCE, SHANK2, TRIP13, HMMR, PLBD1, PHKA2, DCXR, PIK3CB, GRHL1, SIPA1L1, SLC16A3, TP53BP2, INPP5F, TLCD1, PPP2R1B, GPT2, CHEK1, PLAG1, RAD51, TOP2B, MELK, ESRP1, HJURP) | 0.500 (0.500, 0.500) | 0.433 (0.371, 0.495) | 0.719 | 0.504 | 0.500 (±0.0000) | 0.499 (±0.0000) | \| | \| |  |  |  |
| │ │ └── CliXO:366 (TRIP13, HMMR, MELK) | 0.500 (0.500, 0.500) | 0.433 (0.371, 0.495) | 0.719 | 0.504 | 0.502 (±0.0055) | 0.375 (±0.0009) | \| | \| |  |  |  |
| │ ├── CliXO:394 (ARRDC3, WDSUB1, SMPD2, ANKDD1A, AVL9, GCLM, CTBS, ELL2, GRINA, SESN3, NMNAT2, INSL4, TMEM106B, CNNM4, WDR11, CDO1, LHFPL2, NCEH1, NUCB2, DHX29, GPR107, CCDC113, CDS1, AZIN1, SPG21, G3BP2, AK2, PLBD1, SLC38A6, SLC39A8, NPL, APH1B, SIPA1L1, LSM1, TLCD1, TMEM268, CSNK2A2, HSPA9, GPT2, IDE, GPD1L, CLTC, VCP, DOCK8) | 0.500 (0.500, 0.500) | 0.433 (0.371, 0.495) | 0.719 | 0.504 | 0.500 (±0.0044) | 0.400 (±0.0007) | \| | \| |  |  |  |
| │ ├── CliXO:403 (ARRDC3, EDEM1, THOP1, WDSUB1, SMPD2, SLC26A6, MAP3K9, RALGPS1, ADCY7, ANKDD1A, AVL9, ILF2, GCLM, CTBS, PM20D2, ATP10D, ELL2, GRINA, SESN3, BRAF, INSL4, TMEM106B, CNNM4, WDR11, CDO1, SLC38A1, LHFPL2, NCEH1, SHANK2, NUCB2, DHX29, CPOX, GPR107, MIOS, CCDC113, GAN, CDS1, XPO4, INTS7, AZIN1, SPG21, WNK2, G3BP2, FBN2, TRIP13, YTHDC2, TMEM141, BRCA1, AK2, PRKAB2, HMMR, PLBD1, WDFY2, FLVCR2, PHKA2, PDP2, SLC38A6, SLC39A8, NPL, SLC11A1, APH1B, DUS2, PIK3CB, GRHL1, SIPA1L1, MAT2A, MKNK1, TP53BP2, LSM1, NHLRC2, INPP5F, TLCD1, TMEM268, CSNK2A2, ZNF75A, KLHL42, PKD2, PPP2R1B, HSPA9, GGCX, GPT2, EZH2, CHEK1, PLAG1, DONSON, IDE, RAD51, GPD1L, TOP2B, CLTC, ODF2, VCP, DOCK8, MELK, SMARCA2, ETNK2, ARHGAP28, ESRP1, HJURP) | 0.500 (0.500, 0.500) | 0.433 (0.371, 0.495) | 0.719 | 0.504 | 0.497 (±0.0015) | 0.466 (±0.0002) | \| | \| |  |  |  |
| │ │ └── CliXO:366 (TRIP13, HMMR, MELK) | 0.500 (0.500, 0.500) | 0.433 (0.371, 0.495) | 0.719 | 0.504 | 0.502 (±0.0055) | 0.375 (±0.0009) | \| | \| |  |  |  |
| │ └── CliXO:406 (EDEM1, THOP1, SMPD2, SLC26A6, MAP3K9, RALGPS1, ADCY7, ANKDD1A, AVL9, ILF2, GCLM, CTBS, PM20D2, ATP10D, ELL2, GRINA, SESN3, NMNAT2, BRAF, TMEM106B, CNNM4, WDR11, CDO1, PEBP1, SLC38A1, LHFPL2, NCEH1, FANCE, SHANK2, NUCB2, DHX29, CPOX, GPR107, HMGCR, MIOS, CCDC113, GAN, CDS1, XPO4, PCYOX1, INTS7, AZIN1, SPG21, G3BP2, FBN2, YTHDC2, ZNF512B, TMEM141, SACM1L, BRCA1, ERLIN2, AK2, PRKAB2, HMMR, PLBD1, FLVCR2, PHKA2, PDP2, SLC38A6, SLC39A8, NPL, SLC11A1, PTPRJ, C1orf21, MME, DUSP4, KLHL3, RCAN3, APH1B, DUS2, PIK3CB, LDLR, SIPA1L1, SLC24A3, MAT2A, MKNK1, SLC16A3, HGF, TP53BP2, LSM1, NHLRC2, MAN1A2, NPEPPS, INPP5F, TLCD1, TMEM268, CSNK2A2, KLHL42, PKD2, PPP2R1B, HSPA9, GGCX, GPT2, EZH2, TKFC, CHEK1, SPON1, SCMH1, PLAG1, STX6, EPB41L5, DONSON, IDE, GEMIN5, RAD51, MAFF, GPD1L, TOP2B, FNDC3A, STAT5B, CLTC, GADD45G, ODF2, VCP, RHOBTB3, TMEM154, DOCK8, MELK, SMARCA2, ETNK2, ARHGAP28, ESRP1, HJURP, UBE2H) | 0.500 (0.500, 0.500) | 0.433 (0.371, 0.495) | 0.719 | 0.504 | 0.508 (±0.0022) | 0.550 (±0.0003) | \| | \| |  |  |  |
| │ └── CliXO:400 (EDEM1, THOP1, SMPD2, MAP3K9, ANKDD1A, AVL9, ILF2, GCLM, CTBS, PM20D2, GRINA, SESN3, NMNAT2, TMEM106B, CNNM4, WDR11, PEBP1, SLC38A1, LHFPL2, NCEH1, SHANK2, CPOX, GPR107, HMGCR, CDS1, XPO4, INTS7, SPG21, G3BP2, FBN2, ZNF512B, TMEM141, SACM1L, BRCA1, ERLIN2, AK2, PRKAB2, PLBD1, PDP2, SLC38A6, SLC39A8, NPL, C1orf21, MME, RCAN3, DUS2, LDLR, SIPA1L1, SLC24A3, MAT2A, MKNK1, SLC16A3, HGF, TP53BP2, LSM1, NHLRC2, INPP5F, TMEM268, KLHL42, PKD2, PPP2R1B, HSPA9, GGCX, TKFC, CHEK1, SPON1, PLAG1, EPB41L5, DONSON, IDE, GEMIN5, RAD51, GPD1L, TOP2B, CLTC, ODF2, VCP, DOCK8, SMARCA2, ARHGAP28, UBE2H) | 0.500 (0.500, 0.500) | 0.433 (0.371, 0.495) | 0.719 | 0.504 | 0.551 (±0.0073) | 0.681 (±0.0007) | \| | \| |  |  |  |
| │ │ └── CliXO:359 (CDS1, RCAN3) | 0.500 (0.500, 0.500) | 0.433 (0.371, 0.495) | 0.719 | 0.504 | 0.502 (±0.0055) | 0.375 (±0.0009) | \| | \| |  |  |  |
| │ ├── CliXO:360 (PEBP1, LDLR) | 0.500 (0.500, 0.500) | 0.433 (0.371, 0.495) | 0.719 | 0.504 | 0.502 (±0.0055) | 0.375 (±0.0009) | \| | \| |  |  |  |
| │ └── CliXO:375 (HMGCR, ZNF512B, SACM1L, PKD2, DOCK8, SMARCA2) | 0.500 (0.500, 0.500) | 0.433 (0.371, 0.495) | 0.719 | 0.504 | 0.502 (±0.0055) | 0.375 (±0.0009) | \| | \| |  |  |  |
| └── CliXO:415 (CX3CR1, SLIT3, CEBPD, MYO5A, SLC2A1, GNG11, DNAJC1, AKAP13, SCARB1, ZBTB20, PTPN12, IGF1, KIAA0232, CBX5, DYRK2, ETS2, MEF2A, GPER1, ZNF462, IFI44L, CYTL1, GSN, PARM1, NFKB1, RAB3B, SEMA3G, ST6GAL1, MCTP2, SYK, NFIA, TXNDC11, SMIM3, LRRC8A, JAK2, CDK14, MS4A4A, LAPTM5, JUNB, F8, RIN2, CLIC5, CARD16, PRKCE, KIF5B, CCDC59, TBXAS1, DDIT4, GNB1, EXOC6, TMTC4, SAV1, NECTIN4, PPP2R5A, KMT2E, FZD1, CYBB, DPYD, ELMO1, SNAPC1, C3AR1, TSPAN33, HMGN3, MIDN, LTF, RIMKLA, TRIP10, CLOCK, TACC1, FLI1, MBTPS2, MXI1, RNASE1, MAML2, SYPL1, AMOTL2, DDR2, ACTN1, TDP1, TANK, ARRDC3, YPEL2, GALNT18, TYROBP, DUSP1, ZNF281, LAMA4, EFHD1, EDEM1, SBF2, TEX2, THOP1, PPP3CA, TLR7, THRA, LRRC32, HSPA2, PIK3AP1, TSPAN14, ZFYVE1, CHIC2, MLLT1, SLC9A9, MINPP1, DIO2, SULF2, ASTE1, PLCB1, RNF11, CMKLR1, PODXL, SDC4, WDSUB1, PDGFC, SMPD2, SLC26A6, PGRMC1, SLC6A8, GUCA2A, TLR4, MXRA5, MAP3K9, BCORL1, HK1, RCBTB2, ASAP2, RALGPS1, ADCY7, ANGPT1, ARL6IP5, RASAL2, ARTN, ANKDD1A, AVL9, MATN2, ILF2, GCLM, CTBS, GOLM1, PROM1, PM20D2, EBF3, SH2B3, ATP10D, IL33, HNMT, DKK3, ELL2, GRINA, PDE5A, MAP4K4, SESN3, NMNAT2, BRAF, INSL4, NIPAL3, TRIB2, ARID3A, KDM1A, TMEM106B, VEGFA, CNNM4, WDR11, MT1F, CREBL2, PKM, CDO1, NUDT5, HEY1, PEBP1, SLC38A1, LHFPL2, NCEH1, SEMA4C, ZMYND11, PLOD2, TRPC6, FANCE, VEGFC, SHANK2, PFKFB4, ACVRL1, SLC35B3, GBP4, EDN1, NUCB2, DHX29, ADARB1, OLFML2B, CPOX, PAPSS1, GPR107, HMGCR, MIOS, ACSF2, DLX5, CCDC113, GAN, TMEM176B, FCN3, ARHGAP24, CDS1, KIFC3, XPO4, LRCH1, PCYOX1, INTS7, AZIN1, SPG21, APLN, WNK2, G3BP2, FGF13, SLCO2B1, LIMCH1, AGTR1, FBN2, SARAF, CNOT6L, TRIP13, YTHDC2, ZNF512B, TMEM141, TAPT1, INSIG1, RNF20, BRCA1, ERLIN2, CADM3, AMD1, GLA, PUS7, CKS2, ROBO4, AK2, PRKAB2, BTD, HMMR, PLBD1, LEPROTL1, WDFY2, FLVCR2, PHKA2, PDP2, DCXR, SLC38A6, SLC39A8, NPL, SLC11A1, PTPRJ, C1orf21, MME, DUSP4, MXD1, APH1B, DUS2, TMEM43, PIK3CB, ABLIM3, GRHL1, DAB2, LDLR, SIPA1L1, SLC24A3, MAT2A, MKNK1, SLC16A3, GRHL3, EPS8L1, HGF, TP53BP2, LSM1, MOB3C, NHLRC2, CDYL2, KIF2A, MAN1A2, PAPPA, NPEPPS, INPP5F, NEK7, TLCD1, TMEM268, CSNK2A2, CCSAP, KIF17, ZNF75A, KLHL42, PKD2, NIN, PPP2R1B, HSPA9, GGCX, CYP19A1, GPT2, SH3RF1, EZH2, LURAP1L, CHEK1, SPON1, SCMH1, PLAG1, STX6, EPB41L5, DONSON, IDE, PDZD2, PRKCH, RAD51, MAFF, GPD1L, TOP2B, PREP, ARHGAP26, FNDC3A, STAT5B, CLTC, GADD45G, ODF2, VCP, RHOBTB3, TMEM154, DOCK8, MELK, SMARCA2, TTI1, MTMR4, ETNK2, ARHGAP28, ESRP1, HJURP, UBE2H) | 0.716 (0.697, 0.735) | 0.494 (0.424, 0.565) | 0.763 | 0.504 | 0.497 (±0.0043) | 0.406 (±0.0006) | \|✓ | \| |  |  |  |
| ├── CliXO:411 (CX3CR1, SLIT3, CEBPD, MYO5A, SLC2A1, GNG11, DNAJC1, SCARB1, ZBTB20, PTPN12, IGF1, KIAA0232, CBX5, DYRK2, ETS2, GPER1, ZNF462, IFI44L, CYTL1, PARM1, NFKB1, RAB3B, SEMA3G, ST6GAL1, MCTP2, SYK, NFIA, TXNDC11, SMIM3, LRRC8A, JAK2, CDK14, MS4A4A, JUNB, RIN2, CLIC5, CARD16, PRKCE, KIF5B, CCDC59, TBXAS1, DDIT4, GNB1, EXOC6, SAV1, NECTIN4, PPP2R5A, KMT2E, FZD1, CYBB, DPYD, ELMO1, SNAPC1, C3AR1, TSPAN33, MIDN, LTF, RIMKLA, TRIP10, CLOCK, TACC1, FLI1, MBTPS2, MXI1, RNASE1, MAML2, SYPL1, AMOTL2, TDP1, TANK, YPEL2, GALNT18, TYROBP, DUSP1, ZNF281, EFHD1, SBF2, TEX2, TLR7, THRA, LRRC32, HSPA2, PIK3AP1, TSPAN14, ZFYVE1, CHIC2, MLLT1, SLC9A9, MINPP1, ASTE1, PLCB1, RNF11, CMKLR1, PODXL, PDGFC, GUCA2A, TLR4, MXRA5, BCORL1, RCBTB2, ASAP2, ANGPT1, ARL6IP5, RASAL2, ARTN, MATN2, PROM1, EBF3, SH2B3, IL33, DKK3, PDE5A, MAP4K4, TRIB2, CREBL2, HEY1, ZMYND11, TRPC6, VEGFC, ACVRL1, SLC35B3, GBP4, EDN1, ADARB1, OLFML2B, TMEM176B, FCN3, ARHGAP24, KIFC3, LRCH1, APLN, FGF13, SLCO2B1, LIMCH1, AGTR1, SARAF, CNOT6L, TAPT1, INSIG1, RNF20, CADM3, ROBO4, LEPROTL1, PTPRJ, DUSP4, TMEM43, ABLIM3, DAB2, MOB3C, CDYL2, KIF2A, PAPPA, KIF17, NIN, LURAP1L, PDZD2, PRKCH, MAFF, ARHGAP26, FNDC3A, STAT5B, RHOBTB3, MTMR4) | 0.716 (0.697, 0.735) | 0.494 (0.424, 0.565) | 0.763 | 0.504 | 0.509 (±0.0031) | 0.577 (±0.0004) | \|✓ | \| |  |  |  |
| │ ├── CliXO:353 (CYBB, C3AR1) | 0.500 (0.500, 0.500) | 0.433 (0.371, 0.495) | 0.719 | 0.504 | 0.502 (±0.0055) | 0.375 (±0.0009) | \| | \| |  |  |  |
| │ ├── CliXO:361 (PIK3AP1, TLR4) | 0.500 (0.500, 0.500) | 0.433 (0.371, 0.495) | 0.719 | 0.504 | 0.502 (±0.0055) | 0.375 (±0.0009) | \| | \| |  |  |  |
| │ ├── CliXO:362 (SLC2A1, NECTIN4) | 0.500 (0.500, 0.500) | 0.433 (0.371, 0.495) | 0.719 | 0.504 | 0.502 (±0.0055) | 0.375 (±0.0009) | \| | \| |  |  |  |
| │ └── CliXO:371 (CYTL1, FZD1, TACC1, THRA) | 0.500 (0.500, 0.500) | 0.433 (0.371, 0.495) | 0.719 | 0.504 | 0.502 (±0.0055) | 0.375 (±0.0009) | \| | \| |  |  |  |
| │ ├── CliXO:386 (GNG11, SCARB1, DYRK2, CYTL1, RAB3B, MCTP2, JAK2, CLIC5, EXOC6, FZD1, TACC1, EFHD1, RCBTB2) | 0.500 (0.500, 0.500) | 0.433 (0.371, 0.495) | 0.719 | 0.504 | 0.502 (±0.0055) | 0.375 (±0.0009) | \| | \| |  |  |  |
| │ └── CliXO:402 (CEBPD, MYO5A, GNG11, DNAJC1, PTPN12, KIAA0232, CBX5, DYRK2, GPER1, ZNF462, IFI44L, CYTL1, NFKB1, SEMA3G, SYK, TXNDC11, CDK14, PRKCE, CCDC59, TBXAS1, GNB1, SAV1, KMT2E, FZD1, DPYD, SNAPC1, TSPAN33, MIDN, RIMKLA, TRIP10, TACC1, FLI1, MXI1, RNASE1, MAML2, AMOTL2, TDP1, YPEL2, GALNT18, DUSP1, ZNF281, SBF2, TLR7, LRRC32, HSPA2, ZFYVE1, SLC9A9, MINPP1, ASTE1, MXRA5, RCBTB2, ANGPT1, ARL6IP5, MATN2, EBF3, SH2B3, IL33, DKK3, PDE5A, TRIB2, CREBL2, HEY1, TRPC6, ACVRL1, SLC35B3, GBP4, EDN1, ADARB1, TMEM176B, FCN3, ARHGAP24, KIFC3, LRCH1, APLN, FGF13, SLCO2B1, SARAF, CNOT6L, TAPT1, RNF20, ROBO4, LEPROTL1, ABLIM3, DAB2, MOB3C) | 0.716 (0.697, 0.735) | 0.494 (0.424, 0.565) | 0.763 | 0.504 | 0.520 (±0.0044) | 0.607 (±0.0005) | \| | \| |  |  |  |
| │ ├── CliXO:355 (KIAA0232, PRKCE) | 0.500 (0.500, 0.500) | 0.433 (0.371, 0.495) | 0.719 | 0.504 | 0.501 (±0.0054) | 0.379 (±0.0009) | \| | \| |  |  | CliXO:417 |
| │ ├── CliXO:387 (CYTL1, SEMA3G, GNB1, YPEL2, SBF2, LRRC32, HSPA2, MXRA5, ANGPT1, EBF3, PDE5A, ACVRL1, ARHGAP24, KIFC3) | 0.500 (0.500, 0.500) | 0.433 (0.371, 0.495) | 0.719 | 0.504 | 0.502 (±0.0055) | 0.375 (±0.0009) | \| | \| |  |  |  |
| └── CliXO:414 (CX3CR1, SLIT3, CEBPD, MYO5A, GNG11, DNAJC1, AKAP13, SCARB1, ZBTB20, PTPN12, IGF1, KIAA0232, CBX5, DYRK2, ETS2, MEF2A, GPER1, ZNF462, IFI44L, CYTL1, GSN, PARM1, NFKB1, RAB3B, ST6GAL1, MCTP2, SYK, NFIA, TXNDC11, LRRC8A, JAK2, CDK14, MS4A4A, LAPTM5, JUNB, F8, RIN2, CLIC5, CARD16, PRKCE, KIF5B, CCDC59, TBXAS1, DDIT4, GNB1, EXOC6, TMTC4, PPP2R5A, KMT2E, FZD1, CYBB, DPYD, SNAPC1, C3AR1, TSPAN33, HMGN3, MIDN, LTF, RIMKLA, TRIP10, CLOCK, TACC1, FLI1, MXI1, RNASE1, MAML2, SYPL1, AMOTL2, DDR2, ACTN1, TDP1, TANK, ARRDC3, YPEL2, TYROBP, DUSP1, ZNF281, LAMA4, EFHD1, EDEM1, THOP1, PPP3CA, TLR7, HSPA2, PIK3AP1, TSPAN14, ZFYVE1, CHIC2, MLLT1, SLC9A9, MINPP1, DIO2, SULF2, PLCB1, RNF11, CMKLR1, PODXL, SDC4, WDSUB1, SMPD2, SLC26A6, PGRMC1, SLC6A8, TLR4, MXRA5, MAP3K9, BCORL1, HK1, RCBTB2, ASAP2, RALGPS1, ADCY7, ANGPT1, RASAL2, ANKDD1A, AVL9, MATN2, ILF2, GCLM, CTBS, GOLM1, PM20D2, EBF3, SH2B3, ATP10D, IL33, HNMT, DKK3, ELL2, GRINA, PDE5A, SESN3, NMNAT2, BRAF, INSL4, NIPAL3, TRIB2, ARID3A, KDM1A, TMEM106B, VEGFA, CNNM4, WDR11, MT1F, CREBL2, PKM, CDO1, NUDT5, HEY1, PEBP1, SLC38A1, LHFPL2, NCEH1, SEMA4C, ZMYND11, PLOD2, TRPC6, FANCE, VEGFC, SHANK2, PFKFB4, ACVRL1, SLC35B3, GBP4, EDN1, NUCB2, DHX29, ADARB1, OLFML2B, CPOX, PAPSS1, GPR107, HMGCR, MIOS, ACSF2, DLX5, CCDC113, GAN, TMEM176B, FCN3, ARHGAP24, CDS1, KIFC3, XPO4, PCYOX1, INTS7, AZIN1, SPG21, APLN, WNK2, G3BP2, FGF13, SLCO2B1, LIMCH1, AGTR1, FBN2, SARAF, CNOT6L, TRIP13, YTHDC2, ZNF512B, TMEM141, TAPT1, INSIG1, RNF20, BRCA1, ERLIN2, AMD1, GLA, PUS7, CKS2, ROBO4, AK2, PRKAB2, BTD, HMMR, PLBD1, LEPROTL1, WDFY2, FLVCR2, PHKA2, PDP2, DCXR, SLC38A6, SLC39A8, NPL, SLC11A1, PTPRJ, C1orf21, MME, DUSP4, MXD1, APH1B, DUS2, TMEM43, PIK3CB, ABLIM3, GRHL1, DAB2, LDLR, SIPA1L1, SLC24A3, MAT2A, MKNK1, SLC16A3, GRHL3, EPS8L1, HGF, TP53BP2, LSM1, MOB3C, NHLRC2, CDYL2, KIF2A, MAN1A2, PAPPA, NPEPPS, INPP5F, NEK7, TLCD1, TMEM268, CSNK2A2, CCSAP, KIF17, ZNF75A, KLHL42, PKD2, NIN, PPP2R1B, HSPA9, GGCX, CYP19A1, GPT2, SH3RF1, EZH2, LURAP1L, CHEK1, SPON1, SCMH1, PLAG1, STX6, EPB41L5, DONSON, IDE, PDZD2, PRKCH, RAD51, MAFF, GPD1L, TOP2B, PREP, ARHGAP26, FNDC3A, STAT5B, CLTC, GADD45G, ODF2, VCP, RHOBTB3, TMEM154, DOCK8, MELK, SMARCA2, TTI1, MTMR4, ETNK2, ARHGAP28, ESRP1, HJURP, UBE2H) | 0.485 (0.464, 0.507) | 0.337 (0.288, 0.385) | 0.360 | 0.004 | 0.543 (±0.0067) | 0.655 (±0.0007) | \| | \| |  |  |  |
| │ ├── CliXO:354 (DIO2, PGRMC1) | 0.500 (0.500, 0.500) | 0.433 (0.371, 0.495) | 0.719 | 0.504 | 0.502 (±0.0055) | 0.375 (±0.0009) | \| | \| |  |  |  |
| │ ├── CliXO:360 (PEBP1, LDLR) | 0.500 (0.500, 0.500) | 0.433 (0.371, 0.495) | 0.719 | 0.504 | 0.502 (±0.0055) | 0.375 (±0.0009) | \| | \| |  |  |  |
| │ ├── CliXO:367 (NIPAL3, MT1F, PAPSS1) | 0.500 (0.500, 0.500) | 0.433 (0.371, 0.495) | 0.719 | 0.504 | 0.500 (±0.0049) | 0.390 (±0.0008) | \| | \| |  |  | CliXO:410 |
| │ │ ├── CliXO:373 (TMTC4, GOLM1, HNMT, MT1F, PCYOX1) | 0.500 (0.500, 0.500) | 0.433 (0.371, 0.495) | 0.719 | 0.504 | 0.502 (±0.0055) | 0.375 (±0.0009) | \| | \| |  |  |  |
| │ ├── CliXO:376 (TMTC4, HMGN3, ACTN1, PPP3CA, ARID3A, PUS7) | 0.500 (0.500, 0.500) | 0.433 (0.371, 0.495) | 0.719 | 0.504 | 0.502 (±0.0055) | 0.375 (±0.0009) | \| | \| |  |  |  |
| │ ├── CliXO:382 (ACSF2, TRIP13, ZNF512B, DCXR, MME, HGF, NPEPPS, STX6, EPB41L5) | 0.500 (0.500, 0.500) | 0.433 (0.371, 0.495) | 0.719 | 0.504 | 0.502 (±0.0055) | 0.375 (±0.0009) | \| | \| |  |  |  |
| ├── CliXO:388 (AKAP13, ETS2, MEF2A, GSN, PARM1, ST6GAL1, LRRC8A, F8, KIF5B, DDIT4, LTF, DDR2, LAMA4, SULF2, PODXL, EDN1) | 0.500 (0.500, 0.500) | 0.433 (0.371, 0.495) | 0.719 | 0.504 | 0.503 (±0.0060) | 0.364 (±0.0010) | \| | \| |  |  | CliXO:399 |
| │ ├── CliXO:390 (FANCE, SHANK2, TRIP13, HMMR, PLBD1, PHKA2, DCXR, PIK3CB, GRHL1, SIPA1L1, SLC16A3, TP53BP2, INPP5F, TLCD1, PPP2R1B, GPT2, CHEK1, PLAG1, RAD51, TOP2B, MELK, ESRP1, HJURP) | 0.500 (0.500, 0.500) | 0.433 (0.371, 0.495) | 0.719 | 0.504 | 0.500 (±0.0000) | 0.499 (±0.0000) | \| | \| |  |  |  |
| │ │ └── CliXO:366 (TRIP13, HMMR, MELK) | 0.500 (0.500, 0.500) | 0.433 (0.371, 0.495) | 0.719 | 0.504 | 0.502 (±0.0055) | 0.375 (±0.0009) | \| | \| |  |  |  |
| │ ├── CliXO:396 (PEBP1, SEMA4C, SHANK2, PFKFB4, HMGCR, ACSF2, XPO4, TRIP13, YTHDC2, BRCA1, ERLIN2, HMMR, PLBD1, WDFY2, PHKA2, DCXR, C1orf21, MME, APH1B, PIK3CB, SIPA1L1, SLC24A3, MAT2A, SLC16A3, HGF, TP53BP2, LSM1, NPEPPS, INPP5F, TLCD1, ZNF75A, KLHL42, PPP2R1B, GPT2, EZH2, CHEK1, SPON1, STX6, EPB41L5, IDE, RAD51, GPD1L, TOP2B, CLTC, ODF2, VCP, MELK, SMARCA2, TTI1, ETNK2, ARHGAP28, ESRP1, HJURP, UBE2H) | 0.500 (0.500, 0.500) | 0.433 (0.371, 0.495) | 0.719 | 0.504 | 0.500 (±0.0000) | 0.499 (±0.0000) | \| | \| |  |  |  |
| │ │ └── CliXO:366 (TRIP13, HMMR, MELK) | 0.500 (0.500, 0.500) | 0.433 (0.371, 0.495) | 0.719 | 0.504 | 0.502 (±0.0055) | 0.375 (±0.0009) | \| | \| |  |  |  |
| │ ├── CliXO:380 (PEBP1, HMGCR, TRIP13, ERLIN2, C1orf21, SLC24A3, PPP2R1B, GPD1L) | 0.500 (0.500, 0.500) | 0.433 (0.371, 0.495) | 0.719 | 0.504 | 0.502 (±0.0055) | 0.375 (±0.0009) | \| | \| |  |  |  |
| │ │ └── CliXO:389 (PHKA2, HGF, LSM1, ZNF75A, KLHL42, PPP2R1B, EZH2, CHEK1, SPON1, EPB41L5, RAD51, GPD1L, TOP2B, CLTC, ODF2, VCP, MELK, SMARCA2, ETNK2, ARHGAP28, ESRP1, UBE2H) | 0.500 (0.500, 0.500) | 0.433 (0.371, 0.495) | 0.719 | 0.504 | 0.502 (±0.0055) | 0.375 (±0.0009) | \| | \| |  |  |  |
| ├── CliXO:404 (CX3CR1, SLIT3, CEBPD, MYO5A, GNG11, AKAP13, ZBTB20, PTPN12, IGF1, KIAA0232, DYRK2, ETS2, MEF2A, GPER1, ZNF462, CYTL1, GSN, PARM1, RAB3B, ST6GAL1, MCTP2, NFIA, TXNDC11, LRRC8A, JAK2, MS4A4A, JUNB, F8, RIN2, CARD16, PRKCE, KIF5B, CCDC59, EXOC6, KMT2E, FZD1, CYBB, DPYD, C3AR1, TSPAN33, MIDN, LTF, RIMKLA, TRIP10, CLOCK, TACC1, FLI1, MAML2, SYPL1, AMOTL2, DDR2, TANK, YPEL2, ZNF281, LAMA4, EFHD1, TLR7, PIK3AP1, TSPAN14, ZFYVE1, CHIC2, MLLT1, MINPP1, PLCB1, RNF11, CMKLR1, PODXL, TLR4, MXRA5, BCORL1, RCBTB2, ASAP2, ANGPT1, RASAL2, MATN2, IL33, DKK3, PDE5A, TRIB2, CREBL2, HEY1, ZMYND11, TRPC6, VEGFC, EDN1, ADARB1, OLFML2B, TMEM176B, FCN3, ARHGAP24, KIFC3, APLN, SLCO2B1, LIMCH1, AGTR1, SARAF, INSIG1, RNF20, AMD1, ROBO4, LEPROTL1, ABLIM3, MOB3C, LURAP1L, MTMR4) | 0.500 (0.500, 0.500) | 0.433 (0.371, 0.495) | 0.719 | 0.504 | 0.545 (±0.0112) | 0.229 (±0.0033) | \| | \| |  |  |  |
| │ ├── CliXO:377 (AKAP13, ZBTB20, ST6GAL1, NFIA, F8, KIF5B, CLOCK) | 0.500 (0.500, 0.500) | 0.433 (0.371, 0.495) | 0.719 | 0.504 | 0.502 (±0.0055) | 0.375 (±0.0009) | \| | \| |  |  |  |
| │ └── CliXO:397 (GNG11, PTPN12, KIAA0232, DYRK2, CYTL1, RAB3B, MCTP2, JAK2, MS4A4A, RIN2, CARD16, PRKCE, EXOC6, FZD1, CYBB, C3AR1, TACC1, FLI1, SYPL1, TANK, YPEL2, EFHD1, TLR7, PIK3AP1, CHIC2, MINPP1, PLCB1, RNF11, CMKLR1, TLR4, RCBTB2, ASAP2, ANGPT1, MATN2, IL33, DKK3, PDE5A, CREBL2, TRPC6, VEGFC, ADARB1, OLFML2B, TMEM176B, ARHGAP24, SLCO2B1, LIMCH1, AGTR1, SARAF, INSIG1, RNF20, AMD1, LEPROTL1, ABLIM3, MOB3C, LURAP1L, MTMR4) | 0.500 (0.500, 0.500) | 0.433 (0.371, 0.495) | 0.719 | 0.504 | 0.497 (±0.0033) | 0.426 (±0.0005) | \| | \| |  |  | CliXO:417 |
| │ ├── CliXO:353 (CYBB, C3AR1) | 0.500 (0.500, 0.500) | 0.433 (0.371, 0.495) | 0.719 | 0.504 | 0.502 (±0.0055) | 0.375 (±0.0009) | \| | \| |  |  |  |
| │ ├── CliXO:355 (KIAA0232, PRKCE) | 0.500 (0.500, 0.500) | 0.433 (0.371, 0.495) | 0.719 | 0.504 | 0.501 (±0.0054) | 0.379 (±0.0009) | \| | \| |  |  | CliXO:417 |
| │ ├── CliXO:361 (PIK3AP1, TLR4) | 0.500 (0.500, 0.500) | 0.433 (0.371, 0.495) | 0.719 | 0.504 | 0.502 (±0.0055) | 0.375 (±0.0009) | \| | \| |  |  |  |
| └── CliXO:413 (CX3CR1, SLIT3, CEBPD, MYO5A, GNG11, DNAJC1, AKAP13, SCARB1, ZBTB20, PTPN12, IGF1, KIAA0232, CBX5, DYRK2, ETS2, MEF2A, GPER1, ZNF462, IFI44L, CYTL1, GSN, PARM1, NFKB1, RAB3B, ST6GAL1, MCTP2, SYK, NFIA, TXNDC11, LRRC8A, JAK2, CDK14, MS4A4A, LAPTM5, JUNB, F8, RIN2, CLIC5, CARD16, PRKCE, KIF5B, CCDC59, TBXAS1, DDIT4, GNB1, EXOC6, PPP2R5A, KMT2E, FZD1, CYBB, DPYD, SNAPC1, C3AR1, TSPAN33, MIDN, LTF, RIMKLA, TRIP10, TACC1, FLI1, MXI1, RNASE1, MAML2, SYPL1, AMOTL2, DDR2, TDP1, TANK, ARRDC3, YPEL2, TYROBP, DUSP1, ZNF281, LAMA4, EFHD1, EDEM1, THOP1, TLR7, HSPA2, PIK3AP1, TSPAN14, ZFYVE1, CHIC2, MLLT1, SLC9A9, MINPP1, PLCB1, RNF11, CMKLR1, PODXL, WDSUB1, SMPD2, SLC26A6, TLR4, MXRA5, MAP3K9, BCORL1, RCBTB2, ASAP2, RALGPS1, ADCY7, ANGPT1, RASAL2, ANKDD1A, AVL9, MATN2, ILF2, GCLM, CTBS, PM20D2, EBF3, SH2B3, ATP10D, DKK3, ELL2, GRINA, PDE5A, SESN3, NMNAT2, BRAF, INSL4, TRIB2, TMEM106B, CNNM4, WDR11, CDO1, HEY1, SLC38A1, LHFPL2, NCEH1, ZMYND11, TRPC6, VEGFC, SHANK2, ACVRL1, SLC35B3, GBP4, EDN1, NUCB2, DHX29, ADARB1, OLFML2B, CPOX, GPR107, MIOS, CCDC113, GAN, TMEM176B, FCN3, ARHGAP24, CDS1, KIFC3, XPO4, INTS7, AZIN1, SPG21, APLN, WNK2, G3BP2, FGF13, SLCO2B1, LIMCH1, AGTR1, FBN2, SARAF, CNOT6L, TRIP13, YTHDC2, TMEM141, TAPT1, INSIG1, RNF20, BRCA1, AMD1, ROBO4, AK2, PRKAB2, BTD, HMMR, PLBD1, LEPROTL1, WDFY2, FLVCR2, PHKA2, PDP2, SLC38A6, SLC39A8, NPL, SLC11A1, PTPRJ, DUSP4, MXD1, APH1B, DUS2, TMEM43, PIK3CB, ABLIM3, GRHL1, DAB2, SIPA1L1, MAT2A, MKNK1, EPS8L1, TP53BP2, LSM1, MOB3C, NHLRC2, CDYL2, KIF2A, MAN1A2, PAPPA, INPP5F, NEK7, TLCD1, TMEM268, CSNK2A2, CCSAP, KIF17, ZNF75A, KLHL42, PKD2, NIN, PPP2R1B, HSPA9, GGCX, CYP19A1, GPT2, EZH2, LURAP1L, CHEK1, PLAG1, DONSON, IDE, PDZD2, PRKCH, RAD51, MAFF, GPD1L, TOP2B, ARHGAP26, FNDC3A, STAT5B, CLTC, GADD45G, ODF2, VCP, RHOBTB3, DOCK8, MELK, SMARCA2, MTMR4, ETNK2, ARHGAP28, ESRP1, HJURP) | 0.500 (0.500, 0.500) | 0.433 (0.371, 0.495) | 0.719 | 0.504 | 0.569 (±0.0086) | 0.719 (±0.0008) | \| | \| |  |  |  |
| │ ├── CliXO:353 (CYBB, C3AR1) | 0.500 (0.500, 0.500) | 0.433 (0.371, 0.495) | 0.719 | 0.504 | 0.502 (±0.0055) | 0.375 (±0.0009) | \| | \| |  |  |  |
| │ ├── CliXO:355 (KIAA0232, PRKCE) | 0.500 (0.500, 0.500) | 0.433 (0.371, 0.495) | 0.719 | 0.504 | 0.501 (±0.0054) | 0.379 (±0.0009) | \| | \| |  |  | CliXO:417 |
| │ ├── CliXO:361 (PIK3AP1, TLR4) | 0.500 (0.500, 0.500) | 0.433 (0.371, 0.495) | 0.719 | 0.504 | 0.502 (±0.0055) | 0.375 (±0.0009) | \| | \| |  |  |  |
| ├── CliXO:399 (CX3CR1†, SLIT3, CEBPD, MYO5A, DNAJC1, AKAP13, ZBTB20, IGF1, CBX5, ETS2, MEF2A†, GPER1, ZNF462, IFI44L, GSN, PARM1, ST6GAL1, SYK, NFIA, TXNDC11, LRRC8A, CDK14, LAPTM5, JUNB, F8, KIF5B, CCDC59, TBXAS1, GNB1, PPP2R5A, KMT2E, DPYD, SNAPC1, TSPAN33, MIDN, RIMKLA, FLI1, MXI1, RNASE1, AMOTL2, DDR2, TDP1, TYROBP, DUSP1, ZNF281, TLR7, TSPAN14, ZFYVE1, SLC9A9, PODXL, TLR4, ANGPT1, RASAL2, SH2B3, PDE5A, TRIB2, HEY1, ZMYND11, TRPC6, VEGFC, ACVRL1, GBP4, EDN1, ADARB1, OLFML2B, TMEM176B, FCN3, ARHGAP24, KIFC3, APLN, SLCO2B1, AGTR1, SARAF, CNOT6L, ROBO4, LEPROTL1, ABLIM3, LURAP1L) | 0.711 (0.694, 0.728) | 0.345 (0.296, 0.394) | 0.760 | 0.004 | 0.496 (±0.0042) | 0.401 (±0.0007) | \|✓ | \|✓ | ✓ |  | CliXO:399 |
| │ └── CliXO:398 (CX3CR1, SLIT3, CEBPD, MYO5A, DNAJC1, AKAP13, ZBTB20, IGF1, CBX5, ETS2, MEF2A, GPER1, ZNF462, IFI44L, GSN, PARM1, ST6GAL1, NFIA, TXNDC11, LRRC8A, CDK14, JUNB, F8, KIF5B, CCDC59, TBXAS1, GNB1, KMT2E, SNAPC1, TSPAN33, MIDN, RIMKLA, FLI1, MXI1, AMOTL2, DDR2, TDP1, TYROBP, DUSP1, TSPAN14, ZFYVE1, PODXL, ANGPT1, RASAL2, SH2B3, PDE5A, TRIB2, HEY1, ZMYND11, TRPC6, ACVRL1, GBP4, EDN1, TMEM176B, FCN3, KIFC3, APLN, AGTR1, CNOT6L, ROBO4, LEPROTL1, ABLIM3) | 0.500 (0.500, 0.500) | 0.433 (0.371, 0.495) | 0.719 | 0.504 | 0.503 (±0.0060) | 0.364 (±0.0010) | \| | \| |  |  | CliXO:399 |
| └── CliXO:408 (GNG11, SCARB1, DYRK2, CYTL1, RAB3B, MCTP2, JAK2, CLIC5, EXOC6, FZD1, TACC1, TANK, ARRDC3, EFHD1, EDEM1, THOP1, RNF11, WDSUB1, SMPD2, SLC26A6, MAP3K9, RCBTB2, RALGPS1, ADCY7, ANKDD1A, AVL9, ILF2, GCLM, CTBS, PM20D2, ATP10D, ELL2, GRINA, SESN3, NMNAT2, BRAF, INSL4, TMEM106B, CNNM4, WDR11, CDO1, SLC38A1, LHFPL2, NCEH1, SHANK2, NUCB2, DHX29, CPOX, GPR107, MIOS, CCDC113, GAN, CDS1, XPO4, INTS7, AZIN1, SPG21, WNK2, G3BP2, FBN2, TRIP13, YTHDC2, TMEM141, TAPT1, BRCA1, AK2, PRKAB2, BTD, HMMR, PLBD1, WDFY2, FLVCR2, PHKA2, PDP2, SLC38A6, SLC39A8, NPL, SLC11A1, PTPRJ, DUSP4, MXD1, APH1B, DUS2, TMEM43, PIK3CB, GRHL1, DAB2, SIPA1L1, MAT2A, MKNK1, EPS8L1, TP53BP2, LSM1, NHLRC2, CDYL2, KIF2A, MAN1A2, PAPPA, INPP5F, NEK7, TLCD1, TMEM268, CSNK2A2, CCSAP, KIF17, ZNF75A, KLHL42, PKD2, NIN, PPP2R1B, HSPA9, GGCX, CYP19A1, GPT2, EZH2, CHEK1, PLAG1, DONSON, IDE, PDZD2, PRKCH, RAD51, MAFF, GPD1L, TOP2B, ARHGAP26, FNDC3A, STAT5B, CLTC, GADD45G, ODF2, VCP, RHOBTB3, DOCK8, MELK, SMARCA2, MTMR4, ETNK2, ARHGAP28, ESRP1, HJURP) | 0.500 (0.500, 0.500) | 0.433 (0.371, 0.495) | 0.719 | 0.504 | 0.515 (±0.0031) | 0.571 (±0.0003) | \| | \| |  |  |  |
| │ ├── CliXO:386 (GNG11, SCARB1, DYRK2, CYTL1, RAB3B, MCTP2, JAK2, CLIC5, EXOC6, FZD1, TACC1, EFHD1, RCBTB2) | 0.500 (0.500, 0.500) | 0.433 (0.371, 0.495) | 0.719 | 0.504 | 0.502 (±0.0055) | 0.375 (±0.0009) | \| | \| |  |  |  |
| │ ├── CliXO:394 (ARRDC3, WDSUB1, SMPD2, ANKDD1A, AVL9, GCLM, CTBS, ELL2, GRINA, SESN3, NMNAT2, INSL4, TMEM106B, CNNM4, WDR11, CDO1, LHFPL2, NCEH1, NUCB2, DHX29, GPR107, CCDC113, CDS1, AZIN1, SPG21, G3BP2, AK2, PLBD1, SLC38A6, SLC39A8, NPL, APH1B, SIPA1L1, LSM1, TLCD1, TMEM268, CSNK2A2, HSPA9, GPT2, IDE, GPD1L, CLTC, VCP, DOCK8) | 0.500 (0.500, 0.500) | 0.433 (0.371, 0.495) | 0.719 | 0.504 | 0.500 (±0.0044) | 0.400 (±0.0007) | \| | \| |  |  |  |
| │ ├── CliXO:403 (ARRDC3, EDEM1, THOP1, WDSUB1, SMPD2, SLC26A6, MAP3K9, RALGPS1, ADCY7, ANKDD1A, AVL9, ILF2, GCLM, CTBS, PM20D2, ATP10D, ELL2, GRINA, SESN3, BRAF, INSL4, TMEM106B, CNNM4, WDR11, CDO1, SLC38A1, LHFPL2, NCEH1, SHANK2, NUCB2, DHX29, CPOX, GPR107, MIOS, CCDC113, GAN, CDS1, XPO4, INTS7, AZIN1, SPG21, WNK2, G3BP2, FBN2, TRIP13, YTHDC2, TMEM141, BRCA1, AK2, PRKAB2, HMMR, PLBD1, WDFY2, FLVCR2, PHKA2, PDP2, SLC38A6, SLC39A8, NPL, SLC11A1, APH1B, DUS2, PIK3CB, GRHL1, SIPA1L1, MAT2A, MKNK1, TP53BP2, LSM1, NHLRC2, INPP5F, TLCD1, TMEM268, CSNK2A2, ZNF75A, KLHL42, PKD2, PPP2R1B, HSPA9, GGCX, GPT2, EZH2, CHEK1, PLAG1, DONSON, IDE, RAD51, GPD1L, TOP2B, CLTC, ODF2, VCP, DOCK8, MELK, SMARCA2, ETNK2, ARHGAP28, ESRP1, HJURP) | 0.500 (0.500, 0.500) | 0.433 (0.371, 0.495) | 0.719 | 0.504 | 0.497 (±0.0015) | 0.466 (±0.0002) | \| | \| |  |  |  |
| │ │ └── CliXO:366 (TRIP13, HMMR, MELK) | 0.500 (0.500, 0.500) | 0.433 (0.371, 0.495) | 0.719 | 0.504 | 0.502 (±0.0055) | 0.375 (±0.0009) | \| | \| |  |  |  |

The Reported column indicates the ontology from Tables 1–2 to which this row’s eligible genes belong. An ontology is reported if it passes all criteria (C1–C4) and its eligible gene set is not a strict subset of another passing ontology’s gene set. The column is empty when the ontology has no eligible genes or does not pass all criteria. ✓, the ontology passed the criterion; †, the gene had a grad-CAM value >97.5th percentile in the same ontology; |, separator between PPV/TPR and NPV/TNR results in C1 and C2 columns; AUC-PRC, area under the precision-recall curve; AUC-ROC, area under the receiver operating characteristic curve; C1, lower bounds finite and >0; C2, absolute difference ≤25% between development and test sets; C3, at least 1 eligible gene; C4, lower bound of AUC-ROC >0.5 in both sets; CI, confidence interval; Dev, development set; grad-CAM, gradient-weighted class activation mapping; HELLP, hemolysis, elevated liver enzyme, and low platelet; PE, preeclampsia; RMSE, root mean square error.

Table S15. Threshold-specific confusion matrices for the early-onset, severe PE pathogenesis model connected with second-trimester placenta.

| Ontology | PPV/TPR (Dev) | PPV/TPR (Test) | NPV/TNR (Dev) | NPV/TNR (Test) | C1 | C2 | C3 | C4 | Reported |
| --- | --- | --- | --- | --- | --- | --- | --- | --- | --- |
| root (GADD45G, KIF2A, STAT5B, HEY1, TMTC4, SLC35F2, ENG, C1orf21, LIMCH1, CLIC5, CLMN, TAPT1, APLN, SCARB1, HMGN3, GPER1, PAM, EPS8L1, MAFF, VSNL1, PAPPA2†, PRKCH, NECTIN4, SLC7A2, SLC24A3†, RASAL2, CCSAP, NEK7, PLOD2, PAPPA, CDK19, ABLIM3, MTMR4, HJURP, ARHGAP26, SYDE1, DUSP4, ACSF2, MAN1A2, EFHD1, PLCXD2, HMGCR, GOLM1, RAB3B) | 78.2% (73.8, 82.6) / 56.4% (52.6, 60.2) | — / — | 74.6% (72.5, 76.7) / 89.4% (87.5, 91.4) | 99.3% (99.1, 99.5) / 100.0% (100.0, 100.0) | \|✓ | \|✓ | ✓ |  | root |
| ONT:0 (GADD45G, KIF2A, STAT5B, HEY1, TMTC4, ENG, CLIC5, CLMN, TAPT1, APLN, SCARB1, GPER1, PAM, MAFF, PAPPA2, PRKCH, SLC7A2, SLC24A3, RASAL2, CCSAP, PAPPA, ARHGAP26, SYDE1, DUSP4, MAN1A2, EFHD1, HMGCR) | 41.4% (39.0, 43.7) / 100.0% (100.0, 100.0) | 0.7% (0.5, 0.9) / — | — / 0.0% (0.0, 0.0) | — / 0.0% (0.0, 0.0) | \| | \| |  |  | root |
| └── GO:0032501 (GADD45G, KIF2A, STAT5B, HEY1, TMTC4, ENG, CLIC5, CLMN, TAPT1, APLN, SCARB1, GPER1, PAM, MAFF, PAPPA2, PRKCH, SLC7A2, SLC24A3, RASAL2, CCSAP, PAPPA, ARHGAP26, SYDE1, DUSP4, MAN1A2, EFHD1, HMGCR) | 41.4% (39.0, 43.7) / 100.0% (100.0, 100.0) | 0.7% (0.5, 0.9) / — | — / 0.0% (0.0, 0.0) | — / 0.0% (0.0, 0.0) | \| | \| |  |  | root |

The Reported column indicates the ontology from Tables 1–2 to which this row’s eligible genes belong. An ontology is reported if it passes all criteria (C1–C4) and its eligible gene set is not a strict subset of another passing ontology’s gene set. The column is empty when the ontology has no eligible genes or does not pass all criteria. ✓, the ontology passed the criterion; †, the gene had a grad-CAM value >97.5th percentile in the same ontology; |, separator between PPV/TPR and NPV/TNR results in C1 and C2 columns; AUC-ROC, area under the receiver operating characteristic curve; C1, lower bounds finite and >0; C2, absolute difference ≤25% between development and test sets; C3, at least 1 eligible gene; C4, lower bound of AUC-ROC >0.5 in both sets; CI, confidence interval; Dev, development set; grad-CAM, gradient-weighted class activation mapping; HELLP, hemolysis, elevated liver enzyme, and low platelet; NPV, negative predictive value; PE, preeclampsia; PPV, positive predictive value; TNR, true negative rate; TPR, true positive rate.

Table S16. AUC-ROC, AUC-PRC, and RMSE for the early-onset, severe PE pathogenesis model connected with second-trimester placenta.

| Ontology | AUC-ROC (Dev) | AUC-ROC (Test) | AUC-PRC (Dev) | AUC-PRC (Test) | RMSE (Dev) | RMSE (Test) | C1 | C2 | C3 | C4 | Reported |
| --- | --- | --- | --- | --- | --- | --- | --- | --- | --- | --- | --- |
| root (GADD45G, KIF2A, STAT5B, HEY1, TMTC4, SLC35F2, ENG, C1orf21, LIMCH1, CLIC5, CLMN, TAPT1, APLN, SCARB1, HMGN3, GPER1, PAM, EPS8L1, MAFF, VSNL1, PAPPA2†, PRKCH, NECTIN4, SLC7A2, SLC24A3†, RASAL2, CCSAP, NEK7, PLOD2, PAPPA, CDK19, ABLIM3, MTMR4, HJURP, ARHGAP26, SYDE1, DUSP4, ACSF2, MAN1A2, EFHD1, PLCXD2, HMGCR, GOLM1, RAB3B) | 0.845 (0.829, 0.862) | 0.407 (0.333, 0.481) | 0.597 | 0.504 | 0.430 (±0.0124) | 0.190 (±0.0036) | \|✓ | \|✓ | ✓ |  | root |
| ONT:0 (GADD45G, KIF2A, STAT5B, HEY1, TMTC4, ENG, CLIC5, CLMN, TAPT1, APLN, SCARB1, GPER1, PAM, MAFF, PAPPA2, PRKCH, SLC7A2, SLC24A3, RASAL2, CCSAP, PAPPA, ARHGAP26, SYDE1, DUSP4, MAN1A2, EFHD1, HMGCR) | 0.155 (0.138, 0.171) | 0.393 (0.322, 0.465) | 0.104 | 0.004 | 0.710 (±0.0115) | 0.280 (±0.0029) | \| | \| |  |  | root |
| └── GO:0032501 (GADD45G, KIF2A, STAT5B, HEY1, TMTC4, ENG, CLIC5, CLMN, TAPT1, APLN, SCARB1, GPER1, PAM, MAFF, PAPPA2, PRKCH, SLC7A2, SLC24A3, RASAL2, CCSAP, PAPPA, ARHGAP26, SYDE1, DUSP4, MAN1A2, EFHD1, HMGCR) | 0.155 (0.138, 0.171) | 0.393 (0.322, 0.465) | 0.104 | 0.004 | 0.702 (±0.0117) | 0.322 (±0.0022) | \| | \| |  |  | root |

The Reported column indicates the ontology from Tables 1–2 to which this row’s eligible genes belong. An ontology is reported if it passes all criteria (C1–C4) and its eligible gene set is not a strict subset of another passing ontology’s gene set. The column is empty when the ontology has no eligible genes or does not pass all criteria. ✓, the ontology passed the criterion; †, the gene had a grad-CAM value >97.5th percentile in the same ontology; |, separator between PPV/TPR and NPV/TNR results in C1 and C2 columns; AUC-PRC, area under the precision-recall curve; AUC-ROC, area under the receiver operating characteristic curve; C1, lower bounds finite and >0; C2, absolute difference ≤25% between development and test sets; C3, at least 1 eligible gene; C4, lower bound of AUC-ROC >0.5 in both sets; CI, confidence interval; Dev, development set; grad-CAM, gradient-weighted class activation mapping; HELLP, hemolysis, elevated liver enzyme, and low platelet; PE, preeclampsia; RMSE, root mean square error.

Table S17. Threshold-specific confusion matrices for the early-onset, severe PE pathogenesis model connected with third-trimester placenta under HELLP syndrome.

| Ontology | PPV/TPR (Dev) | PPV/TPR (Test) | NPV/TNR (Dev) | NPV/TNR (Test) | C1 | C2 | C3 | C4 | Reported |
| --- | --- | --- | --- | --- | --- | --- | --- | --- | --- |
| root (GPR146†, SUV39H1, PIK3CB, CORO2A, BASP1, DLX5, SFXN3, SLC6A8, ZMYND8, SEMA4C, LRRC8A, CNNM4, HEY1, SEC23A, MIDN, EAF1, ERRFI1, PFKFB4, SLC2A1, VWCE, ANKRD37, ITGA5, GALK1, FANCE, MME, PPL, SWAP70†, CYP2J2, MOB3A, SEMA4B, FBLIM1, ALKBH5†, EFNB1, TSC22D1, KRT19, GUCA2A, C4orf3, SPAG4, SLC16A3, NECTIN4, TPP1, BCAT2, EPS8L2, GREM2†, KLF4†, DUSP1†, DNAJC1, PIK3R1, SMIM3, GLA, NPEPPS, EPHB3, CSNK2A2, SIPA1L1, TOR1B, CLIC3, WFS1, CEBPA, PGRMC1, PLIN2, MYOF, TMEM45A, PLOD2, KLF3, EPS8L1, IGSF8, GRHL1, CXXC1, ENG, ARID3A, FLNB, TIMP3, PPP1R12C, AFMID, PIP5KL1, ELF4, FSTL3, INPP5B, BCORL1, APH1B, INHA, MAP3K9, SYDE1, PMEL, SHANK2, TREM1, IL36RN, HK1, FCN3, AMOT, QPCT, GSN, CCDC127, IPPK, ZNF331, NIM1K, GRHL3, HSPA9, GDPD3, P2RY2, ZDHHC9, MAML2, PIGX, ACKR2, WNK2, CDO1, TM9SF3, P4HA1, PREP, CYB5R1, SLC26A6, PRKAB2, MAP3K8, VEGFA, OVGP1, SLC31A2, TSPAN12, GCLM, PLCXD2, EFHD1, OCEL1, ZFAT, SCARB1, BHLHE40, FOSL2, SLC25A4, JUNB, LRRFIP2, HMOX1, KIF5B, SDC4, BAD, DPP7, WDFY2, FPGS, PRKCH, CLTC, CDK9, TMEM107, DONSON, ERO1A, NEK11, KIT, SPG21, GPT2, LDLR, HPCAL1, RPL15, TRIP10, ZC2HC1A, ABCA1, KMT2E, PHTF2, DIO2, PODXL, MBNL2, GORASP2, MXD1, STK24, CRISPLD2, NUFIP2, ZNF143, VEGFC, RSU1, IQGAP2, SUMO3, CREB1, FRZB, BCAR3, EBI3, NOD1, ASTE1, HMGB3, TXNRD1, SH3BP5, IGSF3, RASEF, MOB3C, TAF9B, PFKM, SH3PXD2A, PAWR, BTD, FKBP8, MTMR6, PIK3AP1, IGF1, PGBD2, MUC15, IDE, CCND1, MIIP, MAP4K4, PCCB, SH3RF1, FRMD4A, TLE6, CLDN16, PKD2, GGCX, MAT2A, NFIL3, ANXA1, NMNAT2, RNF20, ASAH1, CEBPD, C9orf72, GPX3, HSPA2, PRKAR1A, DYRK2, LYN, ARRDC3, MRS2, FEM1B, ERCC6, PDE5A, IL33, FBXO8, PJA1, SPRED1, PIP5K1B†, APLN, GBE1, BTBD10, LEP, TSPAN3, ILVBL, CYB561A3, EXOC6, PAM, GALE, ZNF462, DDR2, STXBP6, PKM, HTRA1, LIMCH1, ARHGAP5, FAM43A, ADK, ATXN10, HPGDS, CALM1, ZFR, FAM20B, TRA2B, PAPPA2, CASD1, WDR11, RANBP6, SACM1L, CYP19A1, SLC23A2, UBE2Q2, HNMT, ZEB2, TRIM24, MXRA5, AZIN1, RBBP4, CCSAP, CD28, SPON1, PROCR, LRRC1, TLR7, UBB, KLHL42, ZNF555, CARD16, ZNF75A, AK2, NHLRC2, ZMYM4, VWA5A, PLPP3, MRPS30, FBN2, ZMYND11, CYBB, EZR†) | 70.7% (68.1, 73.2) / 81.8% (79.5, 84.0) | 2.5% (1.8, 3.1) / — | 83.3% (80.9, 85.7) / 72.8% (70.1, 75.5) | 100.0% (100.0, 100.0) / 61.1% (60.0, 62.2) | \|✓ | \|✓ | ✓ | ✓ | root |
| CliXO:350 (GPR146, SUV39H1, PIK3CB, CORO2A, BASP1, DLX5, SFXN3, SLC6A8, ZMYND8, SEMA4C, LRRC8A, CNNM4, HEY1, SEC23A, MIDN, EAF1, ERRFI1, PFKFB4, SLC2A1, VWCE, ANKRD37, ITGA5, GALK1, FANCE, MME, PPL, SWAP70, CYP2J2, MOB3A, SEMA4B, FBLIM1, ALKBH5, EFNB1, TSC22D1, KRT19, GUCA2A, C4orf3, SPAG4, SLC16A3, NECTIN4, TPP1, BCAT2, EPS8L2, GREM2, KLF4, DUSP1, DNAJC1, PIK3R1, SMIM3, GLA, NPEPPS, EPHB3, CSNK2A2, SIPA1L1, TOR1B, CLIC3, WFS1, CEBPA, PGRMC1, PLIN2, MYOF, TMEM45A, PLOD2, KLF3, EPS8L1, IGSF8, GRHL1, CXXC1, ENG, ARID3A, FLNB, TIMP3, PPP1R12C, AFMID, PIP5KL1, ELF4, FSTL3, INPP5B, BCORL1, APH1B, INHA, MAP3K9, SYDE1, PMEL, SHANK2, TREM1, IL36RN, HK1, FCN3, AMOT, QPCT, GSN, CCDC127, IPPK, ZNF331, NIM1K, GRHL3, HSPA9, GDPD3, P2RY2, ZDHHC9, MAML2, PIGX, ACKR2, WNK2, CDO1, TM9SF3, P4HA1, PREP, CYB5R1, SLC26A6, PRKAB2, MAP3K8, VEGFA, OVGP1, SLC31A2, TSPAN12, GCLM, PLCXD2, EFHD1, OCEL1, ZFAT, SCARB1, BHLHE40, FOSL2, SLC25A4, JUNB, LRRFIP2, HMOX1, KIF5B, SDC4, BAD, DPP7, WDFY2, FPGS, PRKCH, CLTC, CDK9, TMEM107, DONSON, ERO1A, NEK11, KIT, SPG21, GPT2, LDLR, HPCAL1, RPL15, TRIP10, ZC2HC1A, ABCA1, KMT2E, PHTF2, DIO2, PODXL, MBNL2, GORASP2, MXD1, STK24, CRISPLD2, NUFIP2, ZNF143, VEGFC, RSU1, IQGAP2, SUMO3, CREB1, FRZB, BCAR3, EBI3, NOD1, ASTE1, HMGB3, TXNRD1, SH3BP5, IGSF3, RASEF, MOB3C, TAF9B, PFKM, SH3PXD2A, PAWR, BTD, FKBP8, MTMR6, PIK3AP1, IGF1, PGBD2, MUC15, IDE, CCND1, MIIP, MAP4K4, PCCB, SH3RF1, FRMD4A, TLE6, CLDN16, PKD2, GGCX, MAT2A, NFIL3, ANXA1, NMNAT2, RNF20, ASAH1, CEBPD, C9orf72, GPX3, HSPA2, PRKAR1A, DYRK2, LYN, ARRDC3, MRS2, FEM1B, ERCC6, PDE5A, IL33, FBXO8, PJA1, SPRED1, PIP5K1B, APLN, GBE1, BTBD10, LEP, TSPAN3, ILVBL, CYB561A3, EXOC6, PAM, GALE, ZNF462, DDR2, STXBP6, PKM, HTRA1, LIMCH1, ARHGAP5, FAM43A, ADK, ATXN10, HPGDS, CALM1, ZFR, FAM20B, TRA2B, PAPPA2, CASD1, WDR11, RANBP6, SACM1L, CYP19A1, SLC23A2, UBE2Q2, HNMT, ZEB2, TRIM24, MXRA5, AZIN1, RBBP4, CCSAP, CD28, SPON1, PROCR, LRRC1, TLR7, UBB, KLHL42, ZNF555, CARD16, ZNF75A, AK2, NHLRC2, ZMYM4, VWA5A, PLPP3, MRPS30, FBN2, ZMYND11, CYBB, EZR) | 44.2% (42.0, 46.3) / 100.0% (100.0, 100.0) | 1.0% (0.7, 1.2) / — | — / 0.0% (0.0, 0.0) | — / 0.0% (0.0, 0.0) | \| | \| |  | ✓ |  |
| ├── CliXO:323 (AMOT, ZFAT, IDE, MAT2A, ASAH1, TSPAN3, ATXN10) | 44.2% (42.0, 46.3) / 100.0% (100.0, 100.0) | 1.0% (0.7, 1.2) / — | — / 0.0% (0.0, 0.0) | — / 0.0% (0.0, 0.0) | \| | \| |  |  |  |
| │ └── CliXO:310 (AMOT, ZFAT, MAT2A) | 44.2% (42.0, 46.3) / 100.0% (100.0, 100.0) | 1.0% (0.7, 1.2) / — | — / 0.0% (0.0, 0.0) | — / 0.0% (0.0, 0.0) | \| | \| |  |  |  |
| ├── CliXO:326 (CORO2A, TSC22D1, MBNL2, ZNF143, HMGB3, LYN, FEM1B, BTBD10, LIMCH1, LRRC1) | 44.2% (42.0, 46.3) / 100.0% (100.0, 100.0) | 1.0% (0.7, 1.2) / — | — / 0.0% (0.0, 0.0) | — / 0.0% (0.0, 0.0) | \| | \| |  |  |  |
| ├── CliXO:340 (PIK3CB, BASP1, DLX5, SLC6A8, ZMYND8, SEMA4C, CNNM4, ERRFI1, PFKFB4, GALK1, FANCE, MME, SWAP70, CYP2J2, MOB3A, SEMA4B, FBLIM1, SLC16A3, TPP1, GREM2, PIK3R1, SMIM3, GLA, EPHB3, SIPA1L1, CEBPA, MYOF, KLF3, EPS8L1, IGSF8, GRHL1, ENG, FLNB, TIMP3, AFMID, PIP5KL1, ELF4, FSTL3, INPP5B, BCORL1, APH1B, SHANK2, TREM1, HK1, FCN3, AMOT, QPCT, IPPK, ZNF331, NIM1K, ZDHHC9, WNK2, CDO1, P4HA1, PREP, CYB5R1, SLC26A6, PRKAB2, VEGFA, FOSL2, SLC25A4, LRRFIP2, HMOX1, DPP7, WDFY2, FPGS, CDK9, DONSON, KIT, GPT2, LDLR, HPCAL1, ABCA1, PODXL, GORASP2, CRISPLD2, VEGFC, NOD1, MIIP, SH3RF1, FRMD4A, TLE6, GGCX, CEBPD, GPX3, HSPA2, GBE1, FAM43A, TRIM24) | 44.2% (42.0, 46.3) / 100.0% (100.0, 100.0) | 1.0% (0.7, 1.2) / — | — / 0.0% (0.0, 0.0) | — / 0.0% (0.0, 0.0) | \| | \| |  |  |  |
| │ ├── CliXO:284 (PODXL, GPX3) | 44.2% (42.0, 46.3) / 100.0% (100.0, 100.0) | 1.0% (0.7, 1.2) / — | — / 0.0% (0.0, 0.0) | — / 0.0% (0.0, 0.0) | \| | \| |  |  |  |
| │ ├── CliXO:286 (BCORL1, WNK2) | 44.2% (42.0, 46.3) / 100.0% (100.0, 100.0) | 1.0% (0.7, 1.2) / — | — / 0.0% (0.0, 0.0) | — / 0.0% (0.0, 0.0) | \| | \| |  |  |  |
| │ ├── CliXO:291 (EPS8L1, SHANK2) | 44.2% (42.0, 46.3) / 100.0% (100.0, 100.0) | 1.0% (0.7, 1.2) / — | — / 0.0% (0.0, 0.0) | — / 0.0% (0.0, 0.0) | \| | \| |  |  |  |
| │ ├── CliXO:293 (DPP7, GPX3) | 44.2% (42.0, 46.3) / 100.0% (100.0, 100.0) | 1.0% (0.7, 1.2) / — | — / 0.0% (0.0, 0.0) | — / 0.0% (0.0, 0.0) | \| | \| |  |  |  |
| │ ├── CliXO:303 (VEGFA, CRISPLD2) | 44.2% (42.0, 46.3) / 100.0% (100.0, 100.0) | 1.0% (0.7, 1.2) / — | — / 0.0% (0.0, 0.0) | — / 0.0% (0.0, 0.0) | \| | \| |  |  |  |
| │ ├── CliXO:308 (TPP1, GORASP2, GGCX) | 44.2% (42.0, 46.3) / 100.0% (100.0, 100.0) | 1.0% (0.7, 1.2) / — | — / 0.0% (0.0, 0.0) | — / 0.0% (0.0, 0.0) | \| | \| |  |  |  |
| │ ├── CliXO:321 (IGSF8, TIMP3, ZDHHC9, PREP, SLC26A6, LDLR) | 44.2% (42.0, 46.3) / 100.0% (100.0, 100.0) | 1.0% (0.7, 1.2) / — | — / 0.0% (0.0, 0.0) | — / 0.0% (0.0, 0.0) | \| | \| |  |  |  |
| │ └── CliXO:325 (GLA, AMOT, PREP, SLC26A6, SLC25A4, WDFY2, LDLR, CEBPD, FAM43A) | 44.2% (42.0, 46.3) / 100.0% (100.0, 100.0) | 1.0% (0.7, 1.2) / — | — / 0.0% (0.0, 0.0) | — / 0.0% (0.0, 0.0) | \| | \| |  |  |  |
| │ └── CliXO:301 (AMOT, LDLR) | 44.2% (42.0, 46.3) / 100.0% (100.0, 100.0) | 1.0% (0.7, 1.2) / — | — / 0.0% (0.0, 0.0) | — / 0.0% (0.0, 0.0) | \| | \| |  |  |  |
| ├── CliXO:341 (IL36RN, HSPA9, PIGX, TM9SF3, TSPAN12, GCLM, PLCXD2, ZFAT, KIF5B, CLTC, SPG21, RPL15, ZC2HC1A, PHTF2, MBNL2, NUFIP2, RSU1, IQGAP2, SUMO3, CREB1, FRZB, TXNRD1, MOB3C, TAF9B, PFKM, PAWR, MTMR6, IGF1, MUC15, IDE, CCND1, MAP4K4, PCCB, CLDN16, PKD2, MAT2A, ANXA1, NMNAT2, RNF20, ASAH1, C9orf72, PRKAR1A, DYRK2, MRS2, FEM1B, ERCC6, PDE5A, IL33, FBXO8, SPRED1, PIP5K1B, APLN, BTBD10, TSPAN3, CYB561A3, EXOC6, ZNF462, DDR2, STXBP6, ARHGAP5, ADK, ATXN10, HPGDS, ZFR, FAM20B, TRA2B, CASD1, WDR11, RANBP6, SACM1L, CYP19A1, SLC23A2, HNMT, ZEB2, MXRA5, AZIN1, RBBP4, CD28, SPON1, TLR7, UBB, KLHL42, ZNF555, CARD16, ZNF75A, AK2, NHLRC2, ZMYM4, VWA5A, PLPP3, MRPS30, FBN2, ZMYND11, CYBB) | 44.2% (42.0, 46.3) / 100.0% (100.0, 100.0) | 1.0% (0.7, 1.2) / — | — / 0.0% (0.0, 0.0) | — / 0.0% (0.0, 0.0) | \| | \| |  |  |  |
| │ ├── CliXO:283 (SPG21, RSU1) | 44.2% (42.0, 46.3) / 100.0% (100.0, 100.0) | 1.0% (0.7, 1.2) / — | — / 0.0% (0.0, 0.0) | — / 0.0% (0.0, 0.0) | \| | \| |  |  |  |
| │ ├── CliXO:285 (PFKM, SPON1) | 44.2% (42.0, 46.3) / 100.0% (100.0, 100.0) | 1.0% (0.7, 1.2) / — | — / 0.0% (0.0, 0.0) | — / 0.0% (0.0, 0.0) | \| | \| |  |  |  |
| │ ├── CliXO:298 (SUMO3, SLC23A2) | 44.2% (42.0, 46.3) / 100.0% (100.0, 100.0) | 1.0% (0.7, 1.2) / — | — / 0.0% (0.0, 0.0) | — / 0.0% (0.0, 0.0) | \| | \| |  |  |  |
| │ ├── CliXO:314 (IL36RN, PLCXD2, PDE5A, PIP5K1B) | 44.2% (42.0, 46.3) / 100.0% (100.0, 100.0) | 1.0% (0.7, 1.2) / — | — / 0.0% (0.0, 0.0) | — / 0.0% (0.0, 0.0) | \| | \| |  |  |  |
| │ ├── CliXO:319 (FEM1B, BTBD10, ARHGAP5, ADK, ZFR, CASD1) | 44.2% (42.0, 46.3) / 100.0% (100.0, 100.0) | 1.0% (0.7, 1.2) / — | — / 0.0% (0.0, 0.0) | — / 0.0% (0.0, 0.0) | \| | \| |  |  |  |
| │ │ └── CliXO:287 (BTBD10, ADK) | 44.2% (42.0, 46.3) / 100.0% (100.0, 100.0) | 1.0% (0.7, 1.2) / — | — / 0.0% (0.0, 0.0) | — / 0.0% (0.0, 0.0) | \| | \| |  |  |  |
| │ ├── CliXO:320 (MBNL2, PIP5K1B, ZNF462, ZFR, RANBP6, ZMYM4) | 44.2% (42.0, 46.3) / 100.0% (100.0, 100.0) | 1.0% (0.7, 1.2) / — | — / 0.0% (0.0, 0.0) | — / 0.0% (0.0, 0.0) | \| | \| |  |  |  |
| │ │ └── CliXO:302 (MBNL2, ZNF462) | 44.2% (42.0, 46.3) / 100.0% (100.0, 100.0) | 1.0% (0.7, 1.2) / — | — / 0.0% (0.0, 0.0) | — / 0.0% (0.0, 0.0) | \| | \| |  |  |  |
| │ ├── CliXO:330 (TM9SF3, SPG21, ZC2HC1A, PHTF2, ANXA1, RNF20, C9orf72, BTBD10, ZFR, RANBP6, SACM1L, CYP19A1, AZIN1, ZNF75A, AK2, NHLRC2, ZMYND11) | 44.2% (42.0, 46.3) / 100.0% (100.0, 100.0) | 1.0% (0.7, 1.2) / — | — / 0.0% (0.0, 0.0) | — / 0.0% (0.0, 0.0) | \| | \| |  |  |  |
| │ │ └── CliXO:288 (ZNF75A, ZMYND11) | 44.2% (42.0, 46.3) / 100.0% (100.0, 100.0) | 1.0% (0.7, 1.2) / — | — / 0.0% (0.0, 0.0) | — / 0.0% (0.0, 0.0) | \| | \| |  |  |  |
| │ └── CliXO:339 (HSPA9, PIGX, TM9SF3, GCLM, PLCXD2, ZFAT, KIF5B, CLTC, SPG21, RPL15, ZC2HC1A, PHTF2, NUFIP2, IQGAP2, CREB1, FRZB, TXNRD1, TAF9B, PAWR, MTMR6, IGF1, MUC15, IDE, CCND1, MAP4K4, PCCB, CLDN16, PKD2, MAT2A, ANXA1, NMNAT2, RNF20, ASAH1, C9orf72, PRKAR1A, DYRK2, MRS2, ERCC6, PDE5A, IL33, FBXO8, SPRED1, PIP5K1B, BTBD10, TSPAN3, CYB561A3, EXOC6, ZNF462, DDR2, STXBP6, ARHGAP5, ADK, ATXN10, HPGDS, ZFR, FAM20B, TRA2B, CASD1, WDR11, RANBP6, SACM1L, SLC23A2, HNMT, ZEB2, MXRA5, AZIN1, RBBP4, CD28, SPON1, TLR7, UBB, KLHL42, ZNF555, CARD16, ZNF75A, AK2, NHLRC2, ZMYM4, VWA5A, PLPP3, MRPS30, FBN2, ZMYND11, CYBB) | 44.2% (42.0, 46.3) / 100.0% (100.0, 100.0) | 1.0% (0.7, 1.2) / — | — / 0.0% (0.0, 0.0) | — / 0.0% (0.0, 0.0) | \| | \| |  |  |  |
| │ ├── CliXO:289 (NMNAT2, VWA5A) | 44.2% (42.0, 46.3) / 100.0% (100.0, 100.0) | 1.0% (0.7, 1.2) / — | — / 0.0% (0.0, 0.0) | — / 0.0% (0.0, 0.0) | \| | \| |  |  |  |
| │ ├── CliXO:306 (SPG21, TAF9B, FBXO8) | 44.2% (42.0, 46.3) / 100.0% (100.0, 100.0) | 1.0% (0.7, 1.2) / — | — / 0.0% (0.0, 0.0) | — / 0.0% (0.0, 0.0) | \| | \| |  |  |  |
| │ └── CliXO:337 (HSPA9, PIGX, TM9SF3, GCLM, PLCXD2, ZFAT, KIF5B, CLTC, SPG21, RPL15, ZC2HC1A, PHTF2, NUFIP2, IQGAP2, CREB1, FRZB, TAF9B, PAWR, MTMR6, IGF1, MUC15, IDE, CCND1, MAP4K4, PCCB, CLDN16, PKD2, MAT2A, ASAH1, C9orf72, PRKAR1A, DYRK2, MRS2, ERCC6, PDE5A, IL33, SPRED1, PIP5K1B, BTBD10, TSPAN3, CYB561A3, EXOC6, ZNF462, DDR2, STXBP6, ARHGAP5, ADK, ATXN10, HPGDS, ZFR, FAM20B, TRA2B, CASD1, WDR11, RANBP6, SACM1L, SLC23A2, HNMT, ZEB2, MXRA5, AZIN1, RBBP4, CD28, SPON1, TLR7, UBB, KLHL42, ZNF555, CARD16, ZNF75A, NHLRC2, ZMYM4, VWA5A, PLPP3, MRPS30, FBN2, ZMYND11, CYBB) | 44.2% (42.0, 46.3) / 100.0% (100.0, 100.0) | 1.0% (0.7, 1.2) / — | — / 0.0% (0.0, 0.0) | — / 0.0% (0.0, 0.0) | \| | \| |  |  |  |
| │ ├── CliXO:305 (HSPA9, ATXN10, RBBP4) | 44.2% (42.0, 46.3) / 100.0% (100.0, 100.0) | 1.0% (0.7, 1.2) / — | — / 0.0% (0.0, 0.0) | — / 0.0% (0.0, 0.0) | \| | \| |  |  |  |
| │ ├── CliXO:328 (GCLM, PLCXD2, ZC2HC1A, PHTF2, PAWR, CCND1, PCCB, MAT2A, PRKAR1A, PDE5A, STXBP6, CD28, KLHL42, FBN2, CYBB) | 44.2% (42.0, 46.3) / 100.0% (100.0, 100.0) | 1.0% (0.7, 1.2) / — | — / 0.0% (0.0, 0.0) | — / 0.0% (0.0, 0.0) | \| | \| |  |  |  |
| │ │ └── CliXO:309 (PLCXD2, PAWR, PDE5A) | 44.2% (42.0, 46.3) / 100.0% (100.0, 100.0) | 1.0% (0.7, 1.2) / — | — / 0.0% (0.0, 0.0) | — / 0.0% (0.0, 0.0) | \| | \| |  |  |  |
| │ └── CliXO:335 (PIGX, TM9SF3, GCLM, PLCXD2, KIF5B, CLTC, SPG21, RPL15, ZC2HC1A, PHTF2, NUFIP2, IQGAP2, CREB1, TAF9B, PAWR, MTMR6, IGF1, IDE, CCND1, CLDN16, PKD2, ASAH1, C9orf72, PRKAR1A, MRS2, PDE5A, IL33, SPRED1, PIP5K1B, CYB561A3, EXOC6, ZNF462, DDR2, STXBP6, ATXN10, HPGDS, FAM20B, WDR11, SACM1L, SLC23A2, HNMT, ZEB2, MXRA5, AZIN1, RBBP4, CD28, SPON1, TLR7, UBB, KLHL42, CARD16, ZNF75A, NHLRC2, ZMYM4, VWA5A, PLPP3, MRPS30, FBN2, ZMYND11, CYBB) | 44.2% (42.0, 46.3) / 100.0% (100.0, 100.0) | 1.0% (0.7, 1.2) / — | — / 0.0% (0.0, 0.0) | — / 0.0% (0.0, 0.0) | \| | \| |  |  |  |
| │ ├── CliXO:292 (KIF5B, MTMR6) | 44.2% (42.0, 46.3) / 100.0% (100.0, 100.0) | 1.0% (0.7, 1.2) / — | — / 0.0% (0.0, 0.0) | — / 0.0% (0.0, 0.0) | \| | \| |  |  |  |
| │ ├── CliXO:317 (CCND1, IL33, EXOC6, STXBP6, MXRA5) | 44.2% (42.0, 46.3) / 100.0% (100.0, 100.0) | 1.0% (0.7, 1.2) / — | — / 0.0% (0.0, 0.0) | — / 0.0% (0.0, 0.0) | \| | \| |  |  |  |
| │ └── CliXO:332 (TM9SF3, GCLM, CLTC, RPL15, ZC2HC1A, NUFIP2, IGF1, IDE, PKD2, ASAH1, MRS2, SPRED1, PIP5K1B, CYB561A3, ATXN10, HPGDS, FAM20B, WDR11, SLC23A2, HNMT, ZEB2, AZIN1, CD28, SPON1, TLR7, KLHL42, PLPP3, FBN2, ZMYND11, CYBB) | 44.2% (42.0, 46.3) / 100.0% (100.0, 100.0) | 1.0% (0.7, 1.2) / — | — / 0.0% (0.0, 0.0) | — / 0.0% (0.0, 0.0) | \| | \| |  |  |  |
| │ ├── CliXO:304 (IGF1, ZEB2) | 44.2% (42.0, 46.3) / 100.0% (100.0, 100.0) | 1.0% (0.7, 1.2) / — | — / 0.0% (0.0, 0.0) | — / 0.0% (0.0, 0.0) | \| | \| |  |  |  |
| │ ├── CliXO:322 (SPRED1, HPGDS, HNMT, ZEB2, CD28, TLR7, CYBB) | 44.2% (42.0, 46.3) / 100.0% (100.0, 100.0) | 1.0% (0.7, 1.2) / — | — / 0.0% (0.0, 0.0) | — / 0.0% (0.0, 0.0) | \| | \| |  |  |  |
| │ │ └── CliXO:297 (SPRED1, CD28) | 44.2% (42.0, 46.3) / 100.0% (100.0, 100.0) | 1.0% (0.7, 1.2) / — | — / 0.0% (0.0, 0.0) | — / 0.0% (0.0, 0.0) | \| | \| |  |  |  |
| │ └── CliXO:324 (TM9SF3, PKD2, ASAH1, PIP5K1B, FAM20B, KLHL42, PLPP3, FBN2) | 44.2% (42.0, 46.3) / 100.0% (100.0, 100.0) | 1.0% (0.7, 1.2) / — | — / 0.0% (0.0, 0.0) | — / 0.0% (0.0, 0.0) | \| | \| |  |  |  |
| └── CliXO:349 (GPR146, SUV39H1, PIK3CB, CORO2A, BASP1, DLX5, SFXN3, SLC6A8, ZMYND8, SEMA4C, LRRC8A, CNNM4, HEY1, SEC23A, MIDN, EAF1, ERRFI1, PFKFB4, SLC2A1, VWCE, ANKRD37, ITGA5, GALK1, FANCE, MME, PPL, SWAP70, CYP2J2, MOB3A, SEMA4B, FBLIM1, ALKBH5, EFNB1, TSC22D1, KRT19, GUCA2A, C4orf3, SPAG4, SLC16A3, NECTIN4, TPP1, BCAT2, EPS8L2, GREM2, KLF4, DUSP1, DNAJC1, PIK3R1, SMIM3, GLA, NPEPPS, EPHB3, CSNK2A2, SIPA1L1, TOR1B, CLIC3, WFS1, CEBPA, PGRMC1, PLIN2, MYOF, TMEM45A, PLOD2, KLF3, EPS8L1, IGSF8, GRHL1, CXXC1, ENG, ARID3A, FLNB, TIMP3, PPP1R12C, AFMID, PIP5KL1, ELF4, FSTL3, INPP5B, BCORL1, APH1B, INHA, MAP3K9, SYDE1, PMEL, SHANK2, TREM1, HK1, FCN3, QPCT, GSN, CCDC127, IPPK, ZNF331, NIM1K, GRHL3, GDPD3, P2RY2, ZDHHC9, MAML2, ACKR2, WNK2, CDO1, P4HA1, PREP, CYB5R1, SLC26A6, PRKAB2, MAP3K8, VEGFA, OVGP1, SLC31A2, EFHD1, OCEL1, SCARB1, BHLHE40, FOSL2, SLC25A4, JUNB, LRRFIP2, HMOX1, SDC4, BAD, DPP7, FPGS, PRKCH, CDK9, TMEM107, DONSON, ERO1A, NEK11, KIT, GPT2, LDLR, HPCAL1, TRIP10, ABCA1, KMT2E, DIO2, PODXL, GORASP2, MXD1, STK24, CRISPLD2, ZNF143, VEGFC, BCAR3, EBI3, NOD1, ASTE1, HMGB3, SH3BP5, IGSF3, RASEF, SH3PXD2A, BTD, FKBP8, PIK3AP1, PGBD2, MIIP, SH3RF1, FRMD4A, TLE6, GGCX, NFIL3, CEBPD, GPX3, HSPA2, LYN, ARRDC3, PJA1, GBE1, LEP, ILVBL, PAM, GALE, PKM, HTRA1, LIMCH1, FAM43A, CALM1, PAPPA2, UBE2Q2, TRIM24, CCSAP, PROCR, LRRC1, EZR) | 70.7% (68.1, 73.2) / 81.8% (79.5, 84.0) | 2.5% (1.8, 3.1) / — | 83.3% (80.9, 85.7) / 72.8% (70.1, 75.5) | 100.0% (100.0, 100.0) / 61.1% (60.0, 62.2) | \|✓ | \|✓ |  | ✓ | root |
| ├── CliXO:313 (FBLIM1, EPS8L2, MAP3K9, LRRFIP2) | 44.2% (42.0, 46.3) / 100.0% (100.0, 100.0) | 1.0% (0.7, 1.2) / — | — / 0.0% (0.0, 0.0) | — / 0.0% (0.0, 0.0) | \| | \| |  |  |  |
| ├── CliXO:329 (ZMYND8, HEY1, SLC2A1, PPL, CYP2J2, SMIM3, AFMID, PIP5KL1, INPP5B, FCN3, SCARB1, PRKCH, PODXL, VEGFC, NOD1, GPX3) | 44.2% (42.0, 46.3) / 100.0% (100.0, 100.0) | 1.0% (0.7, 1.2) / — | — / 0.0% (0.0, 0.0) | — / 0.0% (0.0, 0.0) | \| | \| |  |  |  |
| └── CliXO:348 (GPR146, SUV39H1, PIK3CB, CORO2A, BASP1, DLX5, SFXN3, SLC6A8, ZMYND8, SEMA4C, LRRC8A, CNNM4, HEY1, SEC23A, MIDN, EAF1, ERRFI1, PFKFB4, SLC2A1, VWCE, ANKRD37, ITGA5, GALK1, FANCE, MME, PPL, SWAP70, CYP2J2, MOB3A, SEMA4B, FBLIM1, ALKBH5, EFNB1, TSC22D1, KRT19, GUCA2A, C4orf3, SPAG4, SLC16A3, NECTIN4, TPP1, BCAT2, EPS8L2, GREM2, KLF4, DUSP1, DNAJC1, PIK3R1, SMIM3, GLA, NPEPPS, EPHB3, CSNK2A2, SIPA1L1, TOR1B, CLIC3, WFS1, CEBPA, PGRMC1, PLIN2, MYOF, TMEM45A, PLOD2, KLF3, EPS8L1, IGSF8, GRHL1, CXXC1, ENG, ARID3A, FLNB, TIMP3, PPP1R12C, AFMID, PIP5KL1, ELF4, FSTL3, INPP5B, BCORL1, APH1B, INHA, MAP3K9, SYDE1, PMEL, SHANK2, TREM1, HK1, QPCT, GSN, CCDC127, IPPK, ZNF331, NIM1K, GRHL3, GDPD3, P2RY2, ZDHHC9, MAML2, ACKR2, WNK2, CDO1, P4HA1, PREP, CYB5R1, SLC26A6, PRKAB2, MAP3K8, VEGFA, OVGP1, SLC31A2, EFHD1, OCEL1, SCARB1, BHLHE40, FOSL2, JUNB, HMOX1, SDC4, BAD, DPP7, FPGS, PRKCH, CDK9, TMEM107, DONSON, ERO1A, NEK11, KIT, GPT2, LDLR, HPCAL1, TRIP10, ABCA1, KMT2E, DIO2, PODXL, GORASP2, MXD1, STK24, CRISPLD2, ZNF143, VEGFC, BCAR3, EBI3, ASTE1, HMGB3, SH3BP5, IGSF3, RASEF, SH3PXD2A, BTD, FKBP8, PIK3AP1, PGBD2, MIIP, SH3RF1, FRMD4A, TLE6, GGCX, NFIL3, CEBPD, GPX3, HSPA2, LYN, ARRDC3, PJA1, GBE1, LEP, ILVBL, PAM, GALE, PKM, HTRA1, LIMCH1, FAM43A, CALM1, PAPPA2, UBE2Q2, TRIM24, CCSAP, PROCR, LRRC1, EZR) | 44.2% (42.0, 46.3) / 100.0% (100.0, 100.0) | 1.0% (0.7, 1.2) / — | — / 0.0% (0.0, 0.0) | — / 0.0% (0.0, 0.0) | \| | \| |  |  | root |
| └── CliXO:347 (GPR146, SUV39H1, PIK3CB, CORO2A, BASP1, DLX5, SFXN3, SLC6A8, ZMYND8, SEMA4C, LRRC8A, CNNM4, HEY1, SEC23A, MIDN, EAF1, ERRFI1, PFKFB4, SLC2A1, VWCE, ANKRD37, ITGA5, GALK1, FANCE, MME, PPL, SWAP70, CYP2J2, MOB3A, SEMA4B, FBLIM1, ALKBH5, EFNB1, TSC22D1, KRT19, GUCA2A, C4orf3, SPAG4, SLC16A3, NECTIN4, TPP1, BCAT2, EPS8L2, GREM2, KLF4, DUSP1, DNAJC1, PIK3R1, SMIM3, GLA, NPEPPS, EPHB3, CSNK2A2, SIPA1L1, TOR1B, CLIC3, WFS1, CEBPA, PGRMC1, PLIN2, MYOF, TMEM45A, PLOD2, KLF3, EPS8L1, IGSF8, GRHL1, CXXC1, ENG, ARID3A, FLNB, TIMP3, PPP1R12C, AFMID, PIP5KL1, ELF4, FSTL3, INPP5B, BCORL1, APH1B, INHA, MAP3K9, SYDE1, PMEL, SHANK2, TREM1, HK1, QPCT, GSN, CCDC127, IPPK, ZNF331, GRHL3, GDPD3, P2RY2, MAML2, ACKR2, WNK2, CDO1, P4HA1, PREP, CYB5R1, PRKAB2, MAP3K8, VEGFA, EFHD1, OCEL1, SCARB1, BHLHE40, FOSL2, JUNB, HMOX1, SDC4, BAD, DPP7, FPGS, PRKCH, CDK9, TMEM107, DONSON, ERO1A, NEK11, KIT, GPT2, HPCAL1, TRIP10, ABCA1, KMT2E, DIO2, PODXL, MXD1, STK24, CRISPLD2, ZNF143, VEGFC, BCAR3, EBI3, ASTE1, HMGB3, SH3BP5, IGSF3, RASEF, SH3PXD2A, BTD, FKBP8, PIK3AP1, PGBD2, MIIP, SH3RF1, FRMD4A, TLE6, NFIL3, CEBPD, GPX3, HSPA2, LYN, ARRDC3, PJA1, GBE1, LEP, ILVBL, PAM, GALE, PKM, HTRA1, LIMCH1, FAM43A, CALM1, PAPPA2, UBE2Q2, TRIM24, CCSAP, PROCR, LRRC1, EZR) | 70.7% (68.1, 73.2) / 81.8% (79.5, 84.0) | 2.5% (1.8, 3.1) / — | 83.3% (80.9, 85.7) / 72.8% (70.1, 75.5) | 100.0% (100.0, 100.0) / 61.1% (60.0, 62.2) | \|✓ | \|✓ |  | ✓ | root |
| ├── CliXO:316 (EPS8L2, ARID3A, BCORL1, WNK2, CDK9) | 44.2% (42.0, 46.3) / 100.0% (100.0, 100.0) | 1.0% (0.7, 1.2) / — | — / 0.0% (0.0, 0.0) | — / 0.0% (0.0, 0.0) | \| | \| |  |  |  |
| └── CliXO:346 (GPR146, SUV39H1, PIK3CB, CORO2A, BASP1, DLX5, SFXN3, SLC6A8, ZMYND8, SEMA4C, LRRC8A, CNNM4, HEY1, SEC23A, MIDN, EAF1, ERRFI1, PFKFB4, SLC2A1, VWCE, ANKRD37, ITGA5, GALK1, FANCE, MME, PPL, SWAP70, CYP2J2, MOB3A, SEMA4B, FBLIM1, ALKBH5, EFNB1, TSC22D1, KRT19, GUCA2A, C4orf3, SPAG4, SLC16A3, NECTIN4, TPP1, BCAT2, EPS8L2, GREM2, KLF4, DUSP1, DNAJC1, PIK3R1, GLA, NPEPPS, EPHB3, CSNK2A2, SIPA1L1, TOR1B, CLIC3, WFS1, CEBPA, PGRMC1, PLIN2, MYOF, TMEM45A, PLOD2, KLF3, EPS8L1, IGSF8, GRHL1, CXXC1, ENG, ARID3A, FLNB, TIMP3, PPP1R12C, AFMID, PIP5KL1, ELF4, FSTL3, INPP5B, BCORL1, APH1B, INHA, MAP3K9, SYDE1, PMEL, SHANK2, TREM1, HK1, QPCT, GSN, CCDC127, ZNF331, GRHL3, GDPD3, P2RY2, MAML2, ACKR2, CDO1, P4HA1, PREP, CYB5R1, PRKAB2, MAP3K8, VEGFA, EFHD1, SCARB1, BHLHE40, FOSL2, JUNB, HMOX1, SDC4, BAD, DPP7, FPGS, PRKCH, CDK9, TMEM107, DONSON, ERO1A, NEK11, KIT, GPT2, HPCAL1, TRIP10, KMT2E, DIO2, PODXL, MXD1, STK24, CRISPLD2, ZNF143, VEGFC, BCAR3, EBI3, ASTE1, HMGB3, SH3BP5, IGSF3, RASEF, SH3PXD2A, BTD, FKBP8, PIK3AP1, PGBD2, MIIP, SH3RF1, FRMD4A, TLE6, NFIL3, CEBPD, GPX3, HSPA2, LYN, ARRDC3, PJA1, GBE1, LEP, ILVBL, PAM, GALE, PKM, HTRA1, LIMCH1, FAM43A, CALM1, PAPPA2, UBE2Q2, TRIM24, CCSAP, PROCR, LRRC1, EZR) | 44.2% (42.0, 46.3) / 100.0% (100.0, 100.0) | 1.0% (0.7, 1.2) / — | — / 0.0% (0.0, 0.0) | — / 0.0% (0.0, 0.0) | \| | \| |  |  | root |
| ├── CliXO:312 (TMEM45A, APH1B, VEGFA, CRISPLD2) | 44.2% (42.0, 46.3) / 100.0% (100.0, 100.0) | 1.0% (0.7, 1.2) / — | — / 0.0% (0.0, 0.0) | — / 0.0% (0.0, 0.0) | \| | \| |  |  |  |
| └── CliXO:345 (GPR146, SUV39H1, PIK3CB, CORO2A, BASP1, DLX5, SFXN3, SLC6A8, ZMYND8, SEMA4C, LRRC8A, CNNM4, HEY1, SEC23A, MIDN, EAF1, ERRFI1, PFKFB4, SLC2A1, VWCE, ANKRD37, ITGA5, GALK1, FANCE, MME, PPL, SWAP70, CYP2J2, MOB3A, SEMA4B, FBLIM1, ALKBH5, EFNB1, TSC22D1, KRT19, GUCA2A, C4orf3, SPAG4, SLC16A3, NECTIN4, TPP1, BCAT2, EPS8L2, GREM2, KLF4, DUSP1, DNAJC1, PIK3R1, GLA, NPEPPS, EPHB3, CSNK2A2, SIPA1L1, TOR1B, CLIC3, WFS1, CEBPA, PLIN2, MYOF, TMEM45A, PLOD2, KLF3, EPS8L1, IGSF8, GRHL1, CXXC1, ENG, ARID3A, FLNB, TIMP3, PPP1R12C, PIP5KL1, ELF4, FSTL3, BCORL1, INHA, MAP3K9, SYDE1, PMEL, SHANK2, TREM1, HK1, QPCT, GSN, CCDC127, ZNF331, GRHL3, GDPD3, P2RY2, MAML2, ACKR2, CDO1, P4HA1, PREP, CYB5R1, PRKAB2, MAP3K8, VEGFA, EFHD1, SCARB1, BHLHE40, FOSL2, JUNB, HMOX1, SDC4, BAD, DPP7, FPGS, PRKCH, CDK9, DONSON, ERO1A, NEK11, KIT, GPT2, HPCAL1, TRIP10, KMT2E, DIO2, MXD1, STK24, CRISPLD2, BCAR3, EBI3, ASTE1, HMGB3, SH3BP5, IGSF3, RASEF, SH3PXD2A, BTD, FKBP8, PIK3AP1, PGBD2, MIIP, SH3RF1, FRMD4A, TLE6, NFIL3, CEBPD, GPX3, HSPA2, LYN, ARRDC3, PJA1, GBE1, LEP, ILVBL, PAM, GALE, PKM, HTRA1, LIMCH1, FAM43A, CALM1, PAPPA2, UBE2Q2, TRIM24, CCSAP, PROCR, LRRC1, EZR) | 44.2% (42.0, 46.3) / 100.0% (100.0, 100.0) | 1.0% (0.7, 1.2) / — | — / 0.0% (0.0, 0.0) | — / 0.0% (0.0, 0.0) | \| | \| |  | ✓ | root |
| ├── CliXO:338 (GPR146, PIK3CB, DLX5, SFXN3, SEMA4C, LRRC8A, EAF1, PFKFB4, VWCE, ITGA5, GALK1, MME, CYP2J2, MOB3A, SEMA4B, ALKBH5, C4orf3, SPAG4, SLC16A3, NECTIN4, EPS8L2, GREM2, KLF4, DNAJC1, GLA, NPEPPS, CSNK2A2, SIPA1L1, TOR1B, CLIC3, TMEM45A, PLOD2, KLF3, EPS8L1, IGSF8, CXXC1, ENG, ARID3A, FLNB, PPP1R12C, ELF4, FSTL3, BCORL1, INHA, MAP3K9, SYDE1, PMEL, SHANK2, HK1, QPCT, GSN, ZNF331, GDPD3, ACKR2, P4HA1, PREP, PRKAB2, SCARB1, FOSL2, SDC4, CDK9, DONSON, ERO1A, GPT2, DIO2, CRISPLD2, SH3BP5, IGSF3, SH3PXD2A, BTD, FKBP8, PIK3AP1, SH3RF1, FRMD4A, TLE6, HSPA2, GBE1, LEP, GALE, CCSAP, PROCR) | 44.2% (42.0, 46.3) / 100.0% (100.0, 100.0) | 1.0% (0.7, 1.2) / — | — / 0.0% (0.0, 0.0) | — / 0.0% (0.0, 0.0) | \| | \| |  | ✓ |  |
| │ ├── CliXO:290 (GLA, BTD) | 44.2% (42.0, 46.3) / 100.0% (100.0, 100.0) | 1.0% (0.7, 1.2) / — | — / 0.0% (0.0, 0.0) | — / 0.0% (0.0, 0.0) | \| | \| |  |  |  |
| │ ├── CliXO:299 (PLOD2, P4HA1) | 44.2% (42.0, 46.3) / 100.0% (100.0, 100.0) | 1.0% (0.7, 1.2) / — | — / 0.0% (0.0, 0.0) | — / 0.0% (0.0, 0.0) | \| | \| |  |  |  |
| │ └── CliXO:307 (INHA, FKBP8, GALE) | 44.2% (42.0, 46.3) / 100.0% (100.0, 100.0) | 1.0% (0.7, 1.2) / — | — / 0.0% (0.0, 0.0) | — / 0.0% (0.0, 0.0) | \| | \| |  |  |  |
| └── CliXO:344 (GPR146, SUV39H1, PIK3CB, CORO2A, BASP1, DLX5, SFXN3, SLC6A8, ZMYND8, SEMA4C, LRRC8A, CNNM4, HEY1, SEC23A, MIDN, EAF1, ERRFI1, PFKFB4, SLC2A1, VWCE, ANKRD37, ITGA5, GALK1, FANCE, MME, PPL, SWAP70, CYP2J2, MOB3A, SEMA4B, FBLIM1, ALKBH5, EFNB1, TSC22D1, KRT19, GUCA2A, C4orf3, SPAG4, SLC16A3, NECTIN4, TPP1, BCAT2, EPS8L2, GREM2, KLF4, DUSP1, DNAJC1, PIK3R1, NPEPPS, EPHB3, CSNK2A2, SIPA1L1, CLIC3, WFS1, CEBPA, PLIN2, MYOF, TMEM45A, PLOD2, KLF3, EPS8L1, IGSF8, GRHL1, ENG, ARID3A, FLNB, TIMP3, PPP1R12C, PIP5KL1, ELF4, FSTL3, BCORL1, INHA, MAP3K9, SYDE1, PMEL, TREM1, HK1, QPCT, GSN, CCDC127, ZNF331, GRHL3, GDPD3, P2RY2, MAML2, ACKR2, CDO1, P4HA1, PREP, CYB5R1, PRKAB2, MAP3K8, VEGFA, EFHD1, SCARB1, BHLHE40, FOSL2, JUNB, HMOX1, SDC4, BAD, DPP7, FPGS, PRKCH, CDK9, ERO1A, NEK11, KIT, GPT2, HPCAL1, TRIP10, KMT2E, DIO2, MXD1, STK24, CRISPLD2, BCAR3, EBI3, ASTE1, HMGB3, SH3BP5, IGSF3, RASEF, SH3PXD2A, BTD, FKBP8, PIK3AP1, PGBD2, MIIP, FRMD4A, NFIL3, CEBPD, GPX3, LYN, ARRDC3, PJA1, GBE1, LEP, ILVBL, PAM, GALE, PKM, HTRA1, LIMCH1, FAM43A, CALM1, PAPPA2, UBE2Q2, TRIM24, CCSAP, PROCR, LRRC1, EZR) | 86.6% (84.2, 89.0) / 52.2% (49.1, 55.3) | — / — | 71.2% (69.0, 73.3) / 93.4% (92.0, 94.9) | 99.0% (98.8, 99.3) / 100.0% (100.0, 100.0) | \|✓ | \| |  | ✓ | root |
| ├── CliXO:296 (NEK11, ASTE1) | 44.2% (42.0, 46.3) / 100.0% (100.0, 100.0) | 1.0% (0.7, 1.2) / — | — / 0.0% (0.0, 0.0) | — / 0.0% (0.0, 0.0) | \| | \| |  |  |  |
| ├── CliXO:318 (ANKRD37, PLIN2, MAP3K8, BHLHE40, NFIL3, ARRDC3) | 44.2% (42.0, 46.3) / 100.0% (100.0, 100.0) | 1.0% (0.7, 1.2) / — | — / 0.0% (0.0, 0.0) | — / 0.0% (0.0, 0.0) | \| | \| |  |  |  |
| ├── CliXO:333 (PIK3CB, SLC6A8, SEMA4C, LRRC8A, VWCE, ITGA5, GALK1, FANCE, MOB3A, FBLIM1, ALKBH5, EFNB1, KRT19, GUCA2A, SPAG4, TPP1, TMEM45A, IGSF8, ENG, FLNB, FSTL3, INHA, GDPD3, P4HA1, CYB5R1, DPP7, HPCAL1, EBI3, BTD, GPX3, LEP, GALE, PROCR) | 44.2% (42.0, 46.3) / 100.0% (100.0, 100.0) | 1.0% (0.7, 1.2) / — | — / 0.0% (0.0, 0.0) | — / 0.0% (0.0, 0.0) | \| | \| |  |  |  |
| │ └── CliXO:315 (SLC6A8, VWCE, GALK1, SPAG4) | 44.2% (42.0, 46.3) / 100.0% (100.0, 100.0) | 1.0% (0.7, 1.2) / — | — / 0.0% (0.0, 0.0) | — / 0.0% (0.0, 0.0) | \| | \| |  |  |  |
| └── CliXO:343 (GPR146, SUV39H1, PIK3CB, CORO2A, BASP1, DLX5, SFXN3, SLC6A8, ZMYND8, SEMA4C, LRRC8A, CNNM4, HEY1, SEC23A, MIDN, EAF1, ERRFI1, PFKFB4, SLC2A1, VWCE, ANKRD37, ITGA5, GALK1, FANCE, MME, PPL, SWAP70, CYP2J2, MOB3A, FBLIM1, ALKBH5, EFNB1, TSC22D1, KRT19, GUCA2A, C4orf3, SPAG4, SLC16A3, NECTIN4, TPP1, BCAT2, EPS8L2, GREM2, KLF4, DUSP1, DNAJC1, PIK3R1, NPEPPS, EPHB3, CSNK2A2, CLIC3, WFS1, CEBPA, PLIN2, MYOF, TMEM45A, PLOD2, KLF3, EPS8L1, IGSF8, GRHL1, ENG, ARID3A, FLNB, TIMP3, PPP1R12C, PIP5KL1, ELF4, FSTL3, INHA, MAP3K9, SYDE1, TREM1, QPCT, ZNF331, GRHL3, P2RY2, MAML2, ACKR2, CDO1, P4HA1, PREP, CYB5R1, PRKAB2, MAP3K8, VEGFA, EFHD1, SCARB1, BHLHE40, FOSL2, HMOX1, SDC4, BAD, DPP7, FPGS, PRKCH, CDK9, ERO1A, NEK11, KIT, GPT2, HPCAL1, TRIP10, KMT2E, DIO2, MXD1, STK24, CRISPLD2, BCAR3, EBI3, HMGB3, SH3BP5, IGSF3, RASEF, SH3PXD2A, BTD, FKBP8, PIK3AP1, FRMD4A, CEBPD, GPX3, LYN, ARRDC3, PJA1, GBE1, LEP, ILVBL, PAM, GALE, PKM, HTRA1, LIMCH1, FAM43A, CALM1, PAPPA2, UBE2Q2, TRIM24, CCSAP, PROCR, LRRC1, EZR) | 44.2% (42.0, 46.3) / 100.0% (100.0, 100.0) | 1.0% (0.7, 1.2) / — | — / 0.0% (0.0, 0.0) | — / 0.0% (0.0, 0.0) | \| | \| |  | ✓ | root |
| ├── CliXO:294 (ZNF331, KMT2E) | 44.2% (42.0, 46.3) / 100.0% (100.0, 100.0) | 1.0% (0.7, 1.2) / — | — / 0.0% (0.0, 0.0) | — / 0.0% (0.0, 0.0) | \| | \| |  |  |  |
| ├── CliXO:334 (PIK3CB, BASP1, SFXN3, SLC6A8, LRRC8A, CNNM4, SLC2A1, ITGA5, GALK1, MOB3A, ALKBH5, GUCA2A, SPAG4, SLC16A3, NECTIN4, KLF4, EPHB3, CLIC3, WFS1, TMEM45A, ENG, ARID3A, PPP1R12C, INHA, TREM1, P2RY2, SCARB1, BAD, FPGS, PRKCH, CDK9, MXD1, EBI3, RASEF, LEP) | 44.2% (42.0, 46.3) / 100.0% (100.0, 100.0) | 1.0% (0.7, 1.2) / — | — / 0.0% (0.0, 0.0) | — / 0.0% (0.0, 0.0) | \| | \| |  |  | root |
| │ └── CliXO:327 (CNNM4, SLC2A1, ALKBH5, NECTIN4, EPHB3, WFS1, ENG, INHA, TREM1, P2RY2, SCARB1, PRKCH, MXD1, EBI3, LEP) | 44.2% (42.0, 46.3) / 100.0% (100.0, 100.0) | 1.0% (0.7, 1.2) / — | — / 0.0% (0.0, 0.0) | — / 0.0% (0.0, 0.0) | \| | \| |  |  | root |
| └── CliXO:342 (GPR146, SUV39H1, PIK3CB, CORO2A, BASP1, DLX5, SFXN3, SLC6A8, ZMYND8, SEMA4C, LRRC8A, HEY1, MIDN, EAF1, ERRFI1, PFKFB4, SLC2A1, VWCE, ANKRD37, ITGA5, GALK1, MME, PPL, CYP2J2, MOB3A, ALKBH5, EFNB1, KRT19, GUCA2A, C4orf3, SPAG4, SLC16A3, NECTIN4, EPS8L2, GREM2, KLF4, DUSP1, PIK3R1, NPEPPS, EPHB3, CSNK2A2, CLIC3, WFS1, PLIN2, MYOF, TMEM45A, PLOD2, KLF3, EPS8L1, GRHL1, ENG, ARID3A, FLNB, PPP1R12C, ELF4, FSTL3, INHA, MAP3K9, SYDE1, QPCT, GRHL3, P2RY2, MAML2, ACKR2, CDO1, P4HA1, PRKAB2, EFHD1, SCARB1, BHLHE40, FOSL2, BAD, CDK9, ERO1A, NEK11, GPT2, TRIP10, KMT2E, DIO2, MXD1, STK24, BCAR3, EBI3, HMGB3, SH3BP5, IGSF3, RASEF, SH3PXD2A, BTD, FKBP8, PIK3AP1, FRMD4A, LYN, ARRDC3, LEP, ILVBL, PAM, GALE, PKM, HTRA1, LIMCH1, CALM1, PAPPA2, UBE2Q2, TRIM24, CCSAP, PROCR, LRRC1, EZR) | 44.2% (42.0, 46.3) / 100.0% (100.0, 100.0) | 1.0% (0.7, 1.2) / — | — / 0.0% (0.0, 0.0) | — / 0.0% (0.0, 0.0) | \| | \| |  |  | root |
| ├── CliXO:295 (WFS1, LYN) | 44.2% (42.0, 46.3) / 100.0% (100.0, 100.0) | 1.0% (0.7, 1.2) / — | — / 0.0% (0.0, 0.0) | — / 0.0% (0.0, 0.0) | \| | \| |  |  |  |
| ├── CliXO:300 (MAML2, KMT2E) | 44.2% (42.0, 46.3) / 100.0% (100.0, 100.0) | 1.0% (0.7, 1.2) / — | — / 0.0% (0.0, 0.0) | — / 0.0% (0.0, 0.0) | \| | \| |  |  |  |
| └── CliXO:336 (GPR146, SUV39H1, PIK3CB, CORO2A, SFXN3, SLC6A8, SEMA4C, LRRC8A, HEY1, MIDN, VWCE, ANKRD37, ITGA5, GALK1, MME, PPL, CYP2J2, MOB3A, ALKBH5, EFNB1, KRT19, SPAG4, SLC16A3, NECTIN4, DUSP1, PLIN2, MYOF, TMEM45A, PLOD2, KLF3, EPS8L1, GRHL1, ENG, ARID3A, FLNB, PPP1R12C, ELF4, FSTL3, INHA, MAP3K9, SYDE1, QPCT, P2RY2, ACKR2, P4HA1, PRKAB2, EFHD1, SCARB1, BHLHE40, FOSL2, ERO1A, GPT2, KMT2E, STK24, SH3BP5, RASEF, SH3PXD2A, PIK3AP1, LYN, ARRDC3, LEP, PAM, PKM, LIMCH1, CALM1, PAPPA2, UBE2Q2, TRIM24, CCSAP, PROCR, LRRC1) | 44.2% (42.0, 46.3) / 100.0% (100.0, 100.0) | 1.0% (0.7, 1.2) / — | — / 0.0% (0.0, 0.0) | — / 0.0% (0.0, 0.0) | \| | \| |  |  | root |
| ├── CliXO:311 (ANKRD37, DUSP1, BHLHE40, ARRDC3) | 44.2% (42.0, 46.3) / 100.0% (100.0, 100.0) | 1.0% (0.7, 1.2) / — | — / 0.0% (0.0, 0.0) | — / 0.0% (0.0, 0.0) | \| | \| |  |  | root |
| └── CliXO:331 (GPR146, PIK3CB, SFXN3, KRT19, SPAG4, NECTIN4, TMEM45A, ENG, FLNB, FSTL3, INHA, SYDE1, QPCT, P4HA1, BHLHE40, ERO1A, GPT2, KMT2E, SH3BP5, RASEF, SH3PXD2A, PIK3AP1, LEP, LIMCH1, CALM1, UBE2Q2, CCSAP, LRRC1) | 44.2% (42.0, 46.3) / 100.0% (100.0, 100.0) | 1.0% (0.7, 1.2) / — | — / 0.0% (0.0, 0.0) | — / 0.0% (0.0, 0.0) | \| | \| |  |  |  |

The Reported column indicates the ontology from Tables 1–2 to which this row’s eligible genes belong. An ontology is reported if it passes all criteria (C1–C4) and its eligible gene set is not a strict subset of another passing ontology’s gene set. The column is empty when the ontology has no eligible genes or does not pass all criteria. ✓, the ontology passed the criterion; †, the gene had a grad-CAM value >97.5th percentile in the same ontology; |, separator between PPV/TPR and NPV/TNR results in C1 and C2 columns; AUC-ROC, area under the receiver operating characteristic curve; C1, lower bounds finite and >0; C2, absolute difference ≤25% between development and test sets; C3, at least 1 eligible gene; C4, lower bound of AUC-ROC >0.5 in both sets; CI, confidence interval; Dev, development set; grad-CAM, gradient-weighted class activation mapping; HELLP, hemolysis, elevated liver enzyme, and low platelet; NPV, negative predictive value; PE, preeclampsia; PPV, positive predictive value; TNR, true negative rate; TPR, true positive rate.

Table S18. AUC-ROC, AUC-PRC, and RMSE for the early-onset, severe PE pathogenesis model connected with third-trimester placenta under HELLP syndrome.

| Ontology | AUC-ROC (Dev) | AUC-ROC (Test) | AUC-PRC (Dev) | AUC-PRC (Test) | RMSE (Dev) | RMSE (Test) | C1 | C2 | C3 | C4 | Reported |
| --- | --- | --- | --- | --- | --- | --- | --- | --- | --- | --- | --- |
| root (GPR146†, SUV39H1, PIK3CB, CORO2A, BASP1, DLX5, SFXN3, SLC6A8, ZMYND8, SEMA4C, LRRC8A, CNNM4, HEY1, SEC23A, MIDN, EAF1, ERRFI1, PFKFB4, SLC2A1, VWCE, ANKRD37, ITGA5, GALK1, FANCE, MME, PPL, SWAP70†, CYP2J2, MOB3A, SEMA4B, FBLIM1, ALKBH5†, EFNB1, TSC22D1, KRT19, GUCA2A, C4orf3, SPAG4, SLC16A3, NECTIN4, TPP1, BCAT2, EPS8L2, GREM2†, KLF4†, DUSP1†, DNAJC1, PIK3R1, SMIM3, GLA, NPEPPS, EPHB3, CSNK2A2, SIPA1L1, TOR1B, CLIC3, WFS1, CEBPA, PGRMC1, PLIN2, MYOF, TMEM45A, PLOD2, KLF3, EPS8L1, IGSF8, GRHL1, CXXC1, ENG, ARID3A, FLNB, TIMP3, PPP1R12C, AFMID, PIP5KL1, ELF4, FSTL3, INPP5B, BCORL1, APH1B, INHA, MAP3K9, SYDE1, PMEL, SHANK2, TREM1, IL36RN, HK1, FCN3, AMOT, QPCT, GSN, CCDC127, IPPK, ZNF331, NIM1K, GRHL3, HSPA9, GDPD3, P2RY2, ZDHHC9, MAML2, PIGX, ACKR2, WNK2, CDO1, TM9SF3, P4HA1, PREP, CYB5R1, SLC26A6, PRKAB2, MAP3K8, VEGFA, OVGP1, SLC31A2, TSPAN12, GCLM, PLCXD2, EFHD1, OCEL1, ZFAT, SCARB1, BHLHE40, FOSL2, SLC25A4, JUNB, LRRFIP2, HMOX1, KIF5B, SDC4, BAD, DPP7, WDFY2, FPGS, PRKCH, CLTC, CDK9, TMEM107, DONSON, ERO1A, NEK11, KIT, SPG21, GPT2, LDLR, HPCAL1, RPL15, TRIP10, ZC2HC1A, ABCA1, KMT2E, PHTF2, DIO2, PODXL, MBNL2, GORASP2, MXD1, STK24, CRISPLD2, NUFIP2, ZNF143, VEGFC, RSU1, IQGAP2, SUMO3, CREB1, FRZB, BCAR3, EBI3, NOD1, ASTE1, HMGB3, TXNRD1, SH3BP5, IGSF3, RASEF, MOB3C, TAF9B, PFKM, SH3PXD2A, PAWR, BTD, FKBP8, MTMR6, PIK3AP1, IGF1, PGBD2, MUC15, IDE, CCND1, MIIP, MAP4K4, PCCB, SH3RF1, FRMD4A, TLE6, CLDN16, PKD2, GGCX, MAT2A, NFIL3, ANXA1, NMNAT2, RNF20, ASAH1, CEBPD, C9orf72, GPX3, HSPA2, PRKAR1A, DYRK2, LYN, ARRDC3, MRS2, FEM1B, ERCC6, PDE5A, IL33, FBXO8, PJA1, SPRED1, PIP5K1B†, APLN, GBE1, BTBD10, LEP, TSPAN3, ILVBL, CYB561A3, EXOC6, PAM, GALE, ZNF462, DDR2, STXBP6, PKM, HTRA1, LIMCH1, ARHGAP5, FAM43A, ADK, ATXN10, HPGDS, CALM1, ZFR, FAM20B, TRA2B, PAPPA2, CASD1, WDR11, RANBP6, SACM1L, CYP19A1, SLC23A2, UBE2Q2, HNMT, ZEB2, TRIM24, MXRA5, AZIN1, RBBP4, CCSAP, CD28, SPON1, PROCR, LRRC1, TLR7, UBB, KLHL42, ZNF555, CARD16, ZNF75A, AK2, NHLRC2, ZMYM4, VWA5A, PLPP3, MRPS30, FBN2, ZMYND11, CYBB, EZR†) | 0.773 (0.754, 0.792) | 0.645 (0.527, 0.762) | 0.798 | 0.504 | 0.564 (±0.0125) | 0.185 (±0.0043) | \|✓ | \|✓ | ✓ | ✓ | root |
| CliXO:350 (GPR146, SUV39H1, PIK3CB, CORO2A, BASP1, DLX5, SFXN3, SLC6A8, ZMYND8, SEMA4C, LRRC8A, CNNM4, HEY1, SEC23A, MIDN, EAF1, ERRFI1, PFKFB4, SLC2A1, VWCE, ANKRD37, ITGA5, GALK1, FANCE, MME, PPL, SWAP70, CYP2J2, MOB3A, SEMA4B, FBLIM1, ALKBH5, EFNB1, TSC22D1, KRT19, GUCA2A, C4orf3, SPAG4, SLC16A3, NECTIN4, TPP1, BCAT2, EPS8L2, GREM2, KLF4, DUSP1, DNAJC1, PIK3R1, SMIM3, GLA, NPEPPS, EPHB3, CSNK2A2, SIPA1L1, TOR1B, CLIC3, WFS1, CEBPA, PGRMC1, PLIN2, MYOF, TMEM45A, PLOD2, KLF3, EPS8L1, IGSF8, GRHL1, CXXC1, ENG, ARID3A, FLNB, TIMP3, PPP1R12C, AFMID, PIP5KL1, ELF4, FSTL3, INPP5B, BCORL1, APH1B, INHA, MAP3K9, SYDE1, PMEL, SHANK2, TREM1, IL36RN, HK1, FCN3, AMOT, QPCT, GSN, CCDC127, IPPK, ZNF331, NIM1K, GRHL3, HSPA9, GDPD3, P2RY2, ZDHHC9, MAML2, PIGX, ACKR2, WNK2, CDO1, TM9SF3, P4HA1, PREP, CYB5R1, SLC26A6, PRKAB2, MAP3K8, VEGFA, OVGP1, SLC31A2, TSPAN12, GCLM, PLCXD2, EFHD1, OCEL1, ZFAT, SCARB1, BHLHE40, FOSL2, SLC25A4, JUNB, LRRFIP2, HMOX1, KIF5B, SDC4, BAD, DPP7, WDFY2, FPGS, PRKCH, CLTC, CDK9, TMEM107, DONSON, ERO1A, NEK11, KIT, SPG21, GPT2, LDLR, HPCAL1, RPL15, TRIP10, ZC2HC1A, ABCA1, KMT2E, PHTF2, DIO2, PODXL, MBNL2, GORASP2, MXD1, STK24, CRISPLD2, NUFIP2, ZNF143, VEGFC, RSU1, IQGAP2, SUMO3, CREB1, FRZB, BCAR3, EBI3, NOD1, ASTE1, HMGB3, TXNRD1, SH3BP5, IGSF3, RASEF, MOB3C, TAF9B, PFKM, SH3PXD2A, PAWR, BTD, FKBP8, MTMR6, PIK3AP1, IGF1, PGBD2, MUC15, IDE, CCND1, MIIP, MAP4K4, PCCB, SH3RF1, FRMD4A, TLE6, CLDN16, PKD2, GGCX, MAT2A, NFIL3, ANXA1, NMNAT2, RNF20, ASAH1, CEBPD, C9orf72, GPX3, HSPA2, PRKAR1A, DYRK2, LYN, ARRDC3, MRS2, FEM1B, ERCC6, PDE5A, IL33, FBXO8, PJA1, SPRED1, PIP5K1B, APLN, GBE1, BTBD10, LEP, TSPAN3, ILVBL, CYB561A3, EXOC6, PAM, GALE, ZNF462, DDR2, STXBP6, PKM, HTRA1, LIMCH1, ARHGAP5, FAM43A, ADK, ATXN10, HPGDS, CALM1, ZFR, FAM20B, TRA2B, PAPPA2, CASD1, WDR11, RANBP6, SACM1L, CYP19A1, SLC23A2, UBE2Q2, HNMT, ZEB2, TRIM24, MXRA5, AZIN1, RBBP4, CCSAP, CD28, SPON1, PROCR, LRRC1, TLR7, UBB, KLHL42, ZNF555, CARD16, ZNF75A, AK2, NHLRC2, ZMYM4, VWA5A, PLPP3, MRPS30, FBN2, ZMYND11, CYBB, EZR) | 0.773 (0.754, 0.792) | 0.645 (0.527, 0.762) | 0.798 | 0.504 | 0.674 (±0.0128) | 0.898 (±0.0011) | \| | \| |  | ✓ |  |
| ├── CliXO:323 (AMOT, ZFAT, IDE, MAT2A, ASAH1, TSPAN3, ATXN10) | 0.500 (0.500, 0.500) | 0.400 (0.327, 0.473) | 0.719 | 0.504 | 0.558 (±0.0121) | 0.201 (±0.0038) | \| | \| |  |  |  |
| │ └── CliXO:310 (AMOT, ZFAT, MAT2A) | 0.500 (0.500, 0.500) | 0.400 (0.327, 0.473) | 0.719 | 0.504 | 0.558 (±0.0121) | 0.201 (±0.0038) | \| | \| |  |  |  |
| ├── CliXO:326 (CORO2A, TSC22D1, MBNL2, ZNF143, HMGB3, LYN, FEM1B, BTBD10, LIMCH1, LRRC1) | 0.500 (0.500, 0.500) | 0.400 (0.327, 0.473) | 0.719 | 0.504 | 0.558 (±0.0121) | 0.201 (±0.0038) | \| | \| |  |  |  |
| ├── CliXO:340 (PIK3CB, BASP1, DLX5, SLC6A8, ZMYND8, SEMA4C, CNNM4, ERRFI1, PFKFB4, GALK1, FANCE, MME, SWAP70, CYP2J2, MOB3A, SEMA4B, FBLIM1, SLC16A3, TPP1, GREM2, PIK3R1, SMIM3, GLA, EPHB3, SIPA1L1, CEBPA, MYOF, KLF3, EPS8L1, IGSF8, GRHL1, ENG, FLNB, TIMP3, AFMID, PIP5KL1, ELF4, FSTL3, INPP5B, BCORL1, APH1B, SHANK2, TREM1, HK1, FCN3, AMOT, QPCT, IPPK, ZNF331, NIM1K, ZDHHC9, WNK2, CDO1, P4HA1, PREP, CYB5R1, SLC26A6, PRKAB2, VEGFA, FOSL2, SLC25A4, LRRFIP2, HMOX1, DPP7, WDFY2, FPGS, CDK9, DONSON, KIT, GPT2, LDLR, HPCAL1, ABCA1, PODXL, GORASP2, CRISPLD2, VEGFC, NOD1, MIIP, SH3RF1, FRMD4A, TLE6, GGCX, CEBPD, GPX3, HSPA2, GBE1, FAM43A, TRIM24) | 0.500 (0.500, 0.500) | 0.400 (0.327, 0.473) | 0.719 | 0.504 | 0.498 (±0.0040) | 0.410 (±0.0005) | \| | \| |  |  |  |
| │ ├── CliXO:284 (PODXL, GPX3) | 0.500 (0.500, 0.500) | 0.400 (0.327, 0.473) | 0.719 | 0.504 | 0.558 (±0.0121) | 0.201 (±0.0038) | \| | \| |  |  |  |
| │ ├── CliXO:286 (BCORL1, WNK2) | 0.500 (0.500, 0.500) | 0.400 (0.327, 0.473) | 0.719 | 0.504 | 0.558 (±0.0121) | 0.201 (±0.0038) | \| | \| |  |  |  |
| │ ├── CliXO:291 (EPS8L1, SHANK2) | 0.500 (0.500, 0.500) | 0.400 (0.327, 0.473) | 0.719 | 0.504 | 0.558 (±0.0121) | 0.201 (±0.0038) | \| | \| |  |  |  |
| │ ├── CliXO:293 (DPP7, GPX3) | 0.500 (0.500, 0.500) | 0.400 (0.327, 0.473) | 0.719 | 0.504 | 0.558 (±0.0121) | 0.201 (±0.0038) | \| | \| |  |  |  |
| │ ├── CliXO:303 (VEGFA, CRISPLD2) | 0.500 (0.500, 0.500) | 0.400 (0.327, 0.473) | 0.719 | 0.504 | 0.558 (±0.0121) | 0.201 (±0.0038) | \| | \| |  |  |  |
| │ ├── CliXO:308 (TPP1, GORASP2, GGCX) | 0.500 (0.500, 0.500) | 0.400 (0.327, 0.473) | 0.719 | 0.504 | 0.558 (±0.0121) | 0.201 (±0.0038) | \| | \| |  |  |  |
| │ ├── CliXO:321 (IGSF8, TIMP3, ZDHHC9, PREP, SLC26A6, LDLR) | 0.500 (0.500, 0.500) | 0.400 (0.327, 0.473) | 0.719 | 0.504 | 0.558 (±0.0121) | 0.201 (±0.0038) | \| | \| |  |  |  |
| │ └── CliXO:325 (GLA, AMOT, PREP, SLC26A6, SLC25A4, WDFY2, LDLR, CEBPD, FAM43A) | 0.500 (0.500, 0.500) | 0.400 (0.327, 0.473) | 0.719 | 0.504 | 0.558 (±0.0121) | 0.201 (±0.0038) | \| | \| |  |  |  |
| │ └── CliXO:301 (AMOT, LDLR) | 0.500 (0.500, 0.500) | 0.400 (0.327, 0.473) | 0.719 | 0.504 | 0.558 (±0.0121) | 0.201 (±0.0038) | \| | \| |  |  |  |
| ├── CliXO:341 (IL36RN, HSPA9, PIGX, TM9SF3, TSPAN12, GCLM, PLCXD2, ZFAT, KIF5B, CLTC, SPG21, RPL15, ZC2HC1A, PHTF2, MBNL2, NUFIP2, RSU1, IQGAP2, SUMO3, CREB1, FRZB, TXNRD1, MOB3C, TAF9B, PFKM, PAWR, MTMR6, IGF1, MUC15, IDE, CCND1, MAP4K4, PCCB, CLDN16, PKD2, MAT2A, ANXA1, NMNAT2, RNF20, ASAH1, C9orf72, PRKAR1A, DYRK2, MRS2, FEM1B, ERCC6, PDE5A, IL33, FBXO8, SPRED1, PIP5K1B, APLN, BTBD10, TSPAN3, CYB561A3, EXOC6, ZNF462, DDR2, STXBP6, ARHGAP5, ADK, ATXN10, HPGDS, ZFR, FAM20B, TRA2B, CASD1, WDR11, RANBP6, SACM1L, CYP19A1, SLC23A2, HNMT, ZEB2, MXRA5, AZIN1, RBBP4, CD28, SPON1, TLR7, UBB, KLHL42, ZNF555, CARD16, ZNF75A, AK2, NHLRC2, ZMYM4, VWA5A, PLPP3, MRPS30, FBN2, ZMYND11, CYBB) | 0.500 (0.500, 0.500) | 0.400 (0.327, 0.473) | 0.719 | 0.504 | 0.558 (±0.0121) | 0.201 (±0.0038) | \| | \| |  |  |  |
| │ ├── CliXO:283 (SPG21, RSU1) | 0.500 (0.500, 0.500) | 0.400 (0.327, 0.473) | 0.719 | 0.504 | 0.558 (±0.0121) | 0.201 (±0.0038) | \| | \| |  |  |  |
| │ ├── CliXO:285 (PFKM, SPON1) | 0.500 (0.500, 0.500) | 0.400 (0.327, 0.473) | 0.719 | 0.504 | 0.558 (±0.0121) | 0.201 (±0.0038) | \| | \| |  |  |  |
| │ ├── CliXO:298 (SUMO3, SLC23A2) | 0.500 (0.500, 0.500) | 0.400 (0.327, 0.473) | 0.719 | 0.504 | 0.558 (±0.0121) | 0.201 (±0.0038) | \| | \| |  |  |  |
| │ ├── CliXO:314 (IL36RN, PLCXD2, PDE5A, PIP5K1B) | 0.500 (0.500, 0.500) | 0.400 (0.327, 0.473) | 0.719 | 0.504 | 0.558 (±0.0121) | 0.201 (±0.0038) | \| | \| |  |  |  |
| │ ├── CliXO:319 (FEM1B, BTBD10, ARHGAP5, ADK, ZFR, CASD1) | 0.500 (0.500, 0.500) | 0.400 (0.327, 0.473) | 0.719 | 0.504 | 0.558 (±0.0121) | 0.201 (±0.0038) | \| | \| |  |  |  |
| │ │ └── CliXO:287 (BTBD10, ADK) | 0.500 (0.500, 0.500) | 0.400 (0.327, 0.473) | 0.719 | 0.504 | 0.558 (±0.0121) | 0.201 (±0.0038) | \| | \| |  |  |  |
| │ ├── CliXO:320 (MBNL2, PIP5K1B, ZNF462, ZFR, RANBP6, ZMYM4) | 0.500 (0.500, 0.500) | 0.400 (0.327, 0.473) | 0.719 | 0.504 | 0.558 (±0.0121) | 0.201 (±0.0038) | \| | \| |  |  |  |
| │ │ └── CliXO:302 (MBNL2, ZNF462) | 0.500 (0.500, 0.500) | 0.400 (0.327, 0.473) | 0.719 | 0.504 | 0.558 (±0.0121) | 0.201 (±0.0038) | \| | \| |  |  |  |
| │ ├── CliXO:330 (TM9SF3, SPG21, ZC2HC1A, PHTF2, ANXA1, RNF20, C9orf72, BTBD10, ZFR, RANBP6, SACM1L, CYP19A1, AZIN1, ZNF75A, AK2, NHLRC2, ZMYND11) | 0.500 (0.500, 0.500) | 0.400 (0.327, 0.473) | 0.719 | 0.504 | 0.558 (±0.0121) | 0.201 (±0.0038) | \| | \| |  |  |  |
| │ │ └── CliXO:288 (ZNF75A, ZMYND11) | 0.500 (0.500, 0.500) | 0.400 (0.327, 0.473) | 0.719 | 0.504 | 0.558 (±0.0121) | 0.201 (±0.0038) | \| | \| |  |  |  |
| │ └── CliXO:339 (HSPA9, PIGX, TM9SF3, GCLM, PLCXD2, ZFAT, KIF5B, CLTC, SPG21, RPL15, ZC2HC1A, PHTF2, NUFIP2, IQGAP2, CREB1, FRZB, TXNRD1, TAF9B, PAWR, MTMR6, IGF1, MUC15, IDE, CCND1, MAP4K4, PCCB, CLDN16, PKD2, MAT2A, ANXA1, NMNAT2, RNF20, ASAH1, C9orf72, PRKAR1A, DYRK2, MRS2, ERCC6, PDE5A, IL33, FBXO8, SPRED1, PIP5K1B, BTBD10, TSPAN3, CYB561A3, EXOC6, ZNF462, DDR2, STXBP6, ARHGAP5, ADK, ATXN10, HPGDS, ZFR, FAM20B, TRA2B, CASD1, WDR11, RANBP6, SACM1L, SLC23A2, HNMT, ZEB2, MXRA5, AZIN1, RBBP4, CD28, SPON1, TLR7, UBB, KLHL42, ZNF555, CARD16, ZNF75A, AK2, NHLRC2, ZMYM4, VWA5A, PLPP3, MRPS30, FBN2, ZMYND11, CYBB) | 0.500 (0.500, 0.500) | 0.400 (0.327, 0.473) | 0.719 | 0.504 | 0.558 (±0.0121) | 0.201 (±0.0038) | \| | \| |  |  |  |
| │ ├── CliXO:289 (NMNAT2, VWA5A) | 0.500 (0.500, 0.500) | 0.400 (0.327, 0.473) | 0.719 | 0.504 | 0.558 (±0.0121) | 0.201 (±0.0038) | \| | \| |  |  |  |
| │ ├── CliXO:306 (SPG21, TAF9B, FBXO8) | 0.500 (0.500, 0.500) | 0.400 (0.327, 0.473) | 0.719 | 0.504 | 0.558 (±0.0121) | 0.201 (±0.0038) | \| | \| |  |  |  |
| │ └── CliXO:337 (HSPA9, PIGX, TM9SF3, GCLM, PLCXD2, ZFAT, KIF5B, CLTC, SPG21, RPL15, ZC2HC1A, PHTF2, NUFIP2, IQGAP2, CREB1, FRZB, TAF9B, PAWR, MTMR6, IGF1, MUC15, IDE, CCND1, MAP4K4, PCCB, CLDN16, PKD2, MAT2A, ASAH1, C9orf72, PRKAR1A, DYRK2, MRS2, ERCC6, PDE5A, IL33, SPRED1, PIP5K1B, BTBD10, TSPAN3, CYB561A3, EXOC6, ZNF462, DDR2, STXBP6, ARHGAP5, ADK, ATXN10, HPGDS, ZFR, FAM20B, TRA2B, CASD1, WDR11, RANBP6, SACM1L, SLC23A2, HNMT, ZEB2, MXRA5, AZIN1, RBBP4, CD28, SPON1, TLR7, UBB, KLHL42, ZNF555, CARD16, ZNF75A, NHLRC2, ZMYM4, VWA5A, PLPP3, MRPS30, FBN2, ZMYND11, CYBB) | 0.500 (0.500, 0.500) | 0.400 (0.327, 0.473) | 0.719 | 0.504 | 0.558 (±0.0121) | 0.201 (±0.0038) | \| | \| |  |  |  |
| │ │ └── CliXO:287 (BTBD10, ADK) | 0.500 (0.500, 0.500) | 0.400 (0.327, 0.473) | 0.719 | 0.504 | 0.558 (±0.0121) | 0.201 (±0.0038) | \| | \| |  |  |  |
| │ ├── CliXO:305 (HSPA9, ATXN10, RBBP4) | 0.500 (0.500, 0.500) | 0.400 (0.327, 0.473) | 0.719 | 0.504 | 0.558 (±0.0121) | 0.201 (±0.0038) | \| | \| |  |  |  |
| │ ├── CliXO:328 (GCLM, PLCXD2, ZC2HC1A, PHTF2, PAWR, CCND1, PCCB, MAT2A, PRKAR1A, PDE5A, STXBP6, CD28, KLHL42, FBN2, CYBB) | 0.500 (0.500, 0.500) | 0.400 (0.327, 0.473) | 0.719 | 0.504 | 0.558 (±0.0121) | 0.201 (±0.0038) | \| | \| |  |  |  |
| │ │ └── CliXO:309 (PLCXD2, PAWR, PDE5A) | 0.500 (0.500, 0.500) | 0.400 (0.327, 0.473) | 0.719 | 0.504 | 0.558 (±0.0121) | 0.201 (±0.0038) | \| | \| |  |  |  |
| │ └── CliXO:335 (PIGX, TM9SF3, GCLM, PLCXD2, KIF5B, CLTC, SPG21, RPL15, ZC2HC1A, PHTF2, NUFIP2, IQGAP2, CREB1, TAF9B, PAWR, MTMR6, IGF1, IDE, CCND1, CLDN16, PKD2, ASAH1, C9orf72, PRKAR1A, MRS2, PDE5A, IL33, SPRED1, PIP5K1B, CYB561A3, EXOC6, ZNF462, DDR2, STXBP6, ATXN10, HPGDS, FAM20B, WDR11, SACM1L, SLC23A2, HNMT, ZEB2, MXRA5, AZIN1, RBBP4, CD28, SPON1, TLR7, UBB, KLHL42, CARD16, ZNF75A, NHLRC2, ZMYM4, VWA5A, PLPP3, MRPS30, FBN2, ZMYND11, CYBB) | 0.500 (0.500, 0.500) | 0.400 (0.327, 0.473) | 0.719 | 0.504 | 0.558 (±0.0121) | 0.201 (±0.0038) | \| | \| |  |  |  |
| │ │ └── CliXO:288 (ZNF75A, ZMYND11) | 0.500 (0.500, 0.500) | 0.400 (0.327, 0.473) | 0.719 | 0.504 | 0.558 (±0.0121) | 0.201 (±0.0038) | \| | \| |  |  |  |
| │ ├── CliXO:292 (KIF5B, MTMR6) | 0.500 (0.500, 0.500) | 0.400 (0.327, 0.473) | 0.719 | 0.504 | 0.558 (±0.0121) | 0.201 (±0.0038) | \| | \| |  |  |  |
| │ │ └── CliXO:309 (PLCXD2, PAWR, PDE5A) | 0.500 (0.500, 0.500) | 0.400 (0.327, 0.473) | 0.719 | 0.504 | 0.558 (±0.0121) | 0.201 (±0.0038) | \| | \| |  |  |  |
| │ ├── CliXO:317 (CCND1, IL33, EXOC6, STXBP6, MXRA5) | 0.500 (0.500, 0.500) | 0.400 (0.327, 0.473) | 0.719 | 0.504 | 0.558 (±0.0121) | 0.201 (±0.0038) | \| | \| |  |  |  |
| │ └── CliXO:332 (TM9SF3, GCLM, CLTC, RPL15, ZC2HC1A, NUFIP2, IGF1, IDE, PKD2, ASAH1, MRS2, SPRED1, PIP5K1B, CYB561A3, ATXN10, HPGDS, FAM20B, WDR11, SLC23A2, HNMT, ZEB2, AZIN1, CD28, SPON1, TLR7, KLHL42, PLPP3, FBN2, ZMYND11, CYBB) | 0.500 (0.500, 0.500) | 0.400 (0.327, 0.473) | 0.719 | 0.504 | 0.558 (±0.0121) | 0.201 (±0.0038) | \| | \| |  |  |  |
| │ ├── CliXO:304 (IGF1, ZEB2) | 0.500 (0.500, 0.500) | 0.400 (0.327, 0.473) | 0.719 | 0.504 | 0.558 (±0.0121) | 0.201 (±0.0038) | \| | \| |  |  |  |
| │ ├── CliXO:322 (SPRED1, HPGDS, HNMT, ZEB2, CD28, TLR7, CYBB) | 0.500 (0.500, 0.500) | 0.400 (0.327, 0.473) | 0.719 | 0.504 | 0.558 (±0.0121) | 0.201 (±0.0038) | \| | \| |  |  |  |
| │ │ └── CliXO:297 (SPRED1, CD28) | 0.500 (0.500, 0.500) | 0.400 (0.327, 0.473) | 0.719 | 0.504 | 0.558 (±0.0121) | 0.201 (±0.0038) | \| | \| |  |  |  |
| │ └── CliXO:324 (TM9SF3, PKD2, ASAH1, PIP5K1B, FAM20B, KLHL42, PLPP3, FBN2) | 0.500 (0.500, 0.500) | 0.400 (0.327, 0.473) | 0.719 | 0.504 | 0.558 (±0.0121) | 0.201 (±0.0038) | \| | \| |  |  |  |
| └── CliXO:349 (GPR146, SUV39H1, PIK3CB, CORO2A, BASP1, DLX5, SFXN3, SLC6A8, ZMYND8, SEMA4C, LRRC8A, CNNM4, HEY1, SEC23A, MIDN, EAF1, ERRFI1, PFKFB4, SLC2A1, VWCE, ANKRD37, ITGA5, GALK1, FANCE, MME, PPL, SWAP70, CYP2J2, MOB3A, SEMA4B, FBLIM1, ALKBH5, EFNB1, TSC22D1, KRT19, GUCA2A, C4orf3, SPAG4, SLC16A3, NECTIN4, TPP1, BCAT2, EPS8L2, GREM2, KLF4, DUSP1, DNAJC1, PIK3R1, SMIM3, GLA, NPEPPS, EPHB3, CSNK2A2, SIPA1L1, TOR1B, CLIC3, WFS1, CEBPA, PGRMC1, PLIN2, MYOF, TMEM45A, PLOD2, KLF3, EPS8L1, IGSF8, GRHL1, CXXC1, ENG, ARID3A, FLNB, TIMP3, PPP1R12C, AFMID, PIP5KL1, ELF4, FSTL3, INPP5B, BCORL1, APH1B, INHA, MAP3K9, SYDE1, PMEL, SHANK2, TREM1, HK1, FCN3, QPCT, GSN, CCDC127, IPPK, ZNF331, NIM1K, GRHL3, GDPD3, P2RY2, ZDHHC9, MAML2, ACKR2, WNK2, CDO1, P4HA1, PREP, CYB5R1, SLC26A6, PRKAB2, MAP3K8, VEGFA, OVGP1, SLC31A2, EFHD1, OCEL1, SCARB1, BHLHE40, FOSL2, SLC25A4, JUNB, LRRFIP2, HMOX1, SDC4, BAD, DPP7, FPGS, PRKCH, CDK9, TMEM107, DONSON, ERO1A, NEK11, KIT, GPT2, LDLR, HPCAL1, TRIP10, ABCA1, KMT2E, DIO2, PODXL, GORASP2, MXD1, STK24, CRISPLD2, ZNF143, VEGFC, BCAR3, EBI3, NOD1, ASTE1, HMGB3, SH3BP5, IGSF3, RASEF, SH3PXD2A, BTD, FKBP8, PIK3AP1, PGBD2, MIIP, SH3RF1, FRMD4A, TLE6, GGCX, NFIL3, CEBPD, GPX3, HSPA2, LYN, ARRDC3, PJA1, GBE1, LEP, ILVBL, PAM, GALE, PKM, HTRA1, LIMCH1, FAM43A, CALM1, PAPPA2, UBE2Q2, TRIM24, CCSAP, PROCR, LRRC1, EZR) | 0.773 (0.754, 0.792) | 0.645 (0.527, 0.762) | 0.798 | 0.504 | 0.506 (±0.0068) | 0.344 (±0.0011) | \|✓ | \|✓ |  | ✓ | root |
| ├── CliXO:313 (FBLIM1, EPS8L2, MAP3K9, LRRFIP2) | 0.500 (0.500, 0.500) | 0.400 (0.327, 0.473) | 0.719 | 0.504 | 0.558 (±0.0121) | 0.201 (±0.0038) | \| | \| |  |  |  |
| ├── CliXO:329 (ZMYND8, HEY1, SLC2A1, PPL, CYP2J2, SMIM3, AFMID, PIP5KL1, INPP5B, FCN3, SCARB1, PRKCH, PODXL, VEGFC, NOD1, GPX3) | 0.500 (0.500, 0.500) | 0.400 (0.327, 0.473) | 0.719 | 0.504 | 0.558 (±0.0121) | 0.201 (±0.0038) | \| | \| |  |  |  |
| │ ├── CliXO:284 (PODXL, GPX3) | 0.500 (0.500, 0.500) | 0.400 (0.327, 0.473) | 0.719 | 0.504 | 0.558 (±0.0121) | 0.201 (±0.0038) | \| | \| |  |  |  |
| └── CliXO:348 (GPR146, SUV39H1, PIK3CB, CORO2A, BASP1, DLX5, SFXN3, SLC6A8, ZMYND8, SEMA4C, LRRC8A, CNNM4, HEY1, SEC23A, MIDN, EAF1, ERRFI1, PFKFB4, SLC2A1, VWCE, ANKRD37, ITGA5, GALK1, FANCE, MME, PPL, SWAP70, CYP2J2, MOB3A, SEMA4B, FBLIM1, ALKBH5, EFNB1, TSC22D1, KRT19, GUCA2A, C4orf3, SPAG4, SLC16A3, NECTIN4, TPP1, BCAT2, EPS8L2, GREM2, KLF4, DUSP1, DNAJC1, PIK3R1, SMIM3, GLA, NPEPPS, EPHB3, CSNK2A2, SIPA1L1, TOR1B, CLIC3, WFS1, CEBPA, PGRMC1, PLIN2, MYOF, TMEM45A, PLOD2, KLF3, EPS8L1, IGSF8, GRHL1, CXXC1, ENG, ARID3A, FLNB, TIMP3, PPP1R12C, AFMID, PIP5KL1, ELF4, FSTL3, INPP5B, BCORL1, APH1B, INHA, MAP3K9, SYDE1, PMEL, SHANK2, TREM1, HK1, QPCT, GSN, CCDC127, IPPK, ZNF331, NIM1K, GRHL3, GDPD3, P2RY2, ZDHHC9, MAML2, ACKR2, WNK2, CDO1, P4HA1, PREP, CYB5R1, SLC26A6, PRKAB2, MAP3K8, VEGFA, OVGP1, SLC31A2, EFHD1, OCEL1, SCARB1, BHLHE40, FOSL2, JUNB, HMOX1, SDC4, BAD, DPP7, FPGS, PRKCH, CDK9, TMEM107, DONSON, ERO1A, NEK11, KIT, GPT2, LDLR, HPCAL1, TRIP10, ABCA1, KMT2E, DIO2, PODXL, GORASP2, MXD1, STK24, CRISPLD2, ZNF143, VEGFC, BCAR3, EBI3, ASTE1, HMGB3, SH3BP5, IGSF3, RASEF, SH3PXD2A, BTD, FKBP8, PIK3AP1, PGBD2, MIIP, SH3RF1, FRMD4A, TLE6, GGCX, NFIL3, CEBPD, GPX3, HSPA2, LYN, ARRDC3, PJA1, GBE1, LEP, ILVBL, PAM, GALE, PKM, HTRA1, LIMCH1, FAM43A, CALM1, PAPPA2, UBE2Q2, TRIM24, CCSAP, PROCR, LRRC1, EZR) | 0.500 (0.500, 0.500) | 0.400 (0.327, 0.473) | 0.719 | 0.504 | 0.505 (±0.0067) | 0.347 (±0.0011) | \| | \| |  |  | root |
| │ ├── CliXO:308 (TPP1, GORASP2, GGCX) | 0.500 (0.500, 0.500) | 0.400 (0.327, 0.473) | 0.719 | 0.504 | 0.558 (±0.0121) | 0.201 (±0.0038) | \| | \| |  |  |  |
| │ ├── CliXO:321 (IGSF8, TIMP3, ZDHHC9, PREP, SLC26A6, LDLR) | 0.500 (0.500, 0.500) | 0.400 (0.327, 0.473) | 0.719 | 0.504 | 0.558 (±0.0121) | 0.201 (±0.0038) | \| | \| |  |  |  |
| └── CliXO:347 (GPR146, SUV39H1, PIK3CB, CORO2A, BASP1, DLX5, SFXN3, SLC6A8, ZMYND8, SEMA4C, LRRC8A, CNNM4, HEY1, SEC23A, MIDN, EAF1, ERRFI1, PFKFB4, SLC2A1, VWCE, ANKRD37, ITGA5, GALK1, FANCE, MME, PPL, SWAP70, CYP2J2, MOB3A, SEMA4B, FBLIM1, ALKBH5, EFNB1, TSC22D1, KRT19, GUCA2A, C4orf3, SPAG4, SLC16A3, NECTIN4, TPP1, BCAT2, EPS8L2, GREM2, KLF4, DUSP1, DNAJC1, PIK3R1, SMIM3, GLA, NPEPPS, EPHB3, CSNK2A2, SIPA1L1, TOR1B, CLIC3, WFS1, CEBPA, PGRMC1, PLIN2, MYOF, TMEM45A, PLOD2, KLF3, EPS8L1, IGSF8, GRHL1, CXXC1, ENG, ARID3A, FLNB, TIMP3, PPP1R12C, AFMID, PIP5KL1, ELF4, FSTL3, INPP5B, BCORL1, APH1B, INHA, MAP3K9, SYDE1, PMEL, SHANK2, TREM1, HK1, QPCT, GSN, CCDC127, IPPK, ZNF331, GRHL3, GDPD3, P2RY2, MAML2, ACKR2, WNK2, CDO1, P4HA1, PREP, CYB5R1, PRKAB2, MAP3K8, VEGFA, EFHD1, OCEL1, SCARB1, BHLHE40, FOSL2, JUNB, HMOX1, SDC4, BAD, DPP7, FPGS, PRKCH, CDK9, TMEM107, DONSON, ERO1A, NEK11, KIT, GPT2, HPCAL1, TRIP10, ABCA1, KMT2E, DIO2, PODXL, MXD1, STK24, CRISPLD2, ZNF143, VEGFC, BCAR3, EBI3, ASTE1, HMGB3, SH3BP5, IGSF3, RASEF, SH3PXD2A, BTD, FKBP8, PIK3AP1, PGBD2, MIIP, SH3RF1, FRMD4A, TLE6, NFIL3, CEBPD, GPX3, HSPA2, LYN, ARRDC3, PJA1, GBE1, LEP, ILVBL, PAM, GALE, PKM, HTRA1, LIMCH1, FAM43A, CALM1, PAPPA2, UBE2Q2, TRIM24, CCSAP, PROCR, LRRC1, EZR) | 0.773 (0.754, 0.792) | 0.645 (0.527, 0.762) | 0.730 | 0.504 | 0.503 (±0.0064) | 0.354 (±0.0010) | \|✓ | \|✓ |  | ✓ | root |
| ├── CliXO:316 (EPS8L2, ARID3A, BCORL1, WNK2, CDK9) | 0.500 (0.500, 0.500) | 0.400 (0.327, 0.473) | 0.719 | 0.504 | 0.558 (±0.0121) | 0.201 (±0.0038) | \| | \| |  |  |  |
| │ ├── CliXO:286 (BCORL1, WNK2) | 0.500 (0.500, 0.500) | 0.400 (0.327, 0.473) | 0.719 | 0.504 | 0.558 (±0.0121) | 0.201 (±0.0038) | \| | \| |  |  |  |
| └── CliXO:346 (GPR146, SUV39H1, PIK3CB, CORO2A, BASP1, DLX5, SFXN3, SLC6A8, ZMYND8, SEMA4C, LRRC8A, CNNM4, HEY1, SEC23A, MIDN, EAF1, ERRFI1, PFKFB4, SLC2A1, VWCE, ANKRD37, ITGA5, GALK1, FANCE, MME, PPL, SWAP70, CYP2J2, MOB3A, SEMA4B, FBLIM1, ALKBH5, EFNB1, TSC22D1, KRT19, GUCA2A, C4orf3, SPAG4, SLC16A3, NECTIN4, TPP1, BCAT2, EPS8L2, GREM2, KLF4, DUSP1, DNAJC1, PIK3R1, GLA, NPEPPS, EPHB3, CSNK2A2, SIPA1L1, TOR1B, CLIC3, WFS1, CEBPA, PGRMC1, PLIN2, MYOF, TMEM45A, PLOD2, KLF3, EPS8L1, IGSF8, GRHL1, CXXC1, ENG, ARID3A, FLNB, TIMP3, PPP1R12C, AFMID, PIP5KL1, ELF4, FSTL3, INPP5B, BCORL1, APH1B, INHA, MAP3K9, SYDE1, PMEL, SHANK2, TREM1, HK1, QPCT, GSN, CCDC127, ZNF331, GRHL3, GDPD3, P2RY2, MAML2, ACKR2, CDO1, P4HA1, PREP, CYB5R1, PRKAB2, MAP3K8, VEGFA, EFHD1, SCARB1, BHLHE40, FOSL2, JUNB, HMOX1, SDC4, BAD, DPP7, FPGS, PRKCH, CDK9, TMEM107, DONSON, ERO1A, NEK11, KIT, GPT2, HPCAL1, TRIP10, KMT2E, DIO2, PODXL, MXD1, STK24, CRISPLD2, ZNF143, VEGFC, BCAR3, EBI3, ASTE1, HMGB3, SH3BP5, IGSF3, RASEF, SH3PXD2A, BTD, FKBP8, PIK3AP1, PGBD2, MIIP, SH3RF1, FRMD4A, TLE6, NFIL3, CEBPD, GPX3, HSPA2, LYN, ARRDC3, PJA1, GBE1, LEP, ILVBL, PAM, GALE, PKM, HTRA1, LIMCH1, FAM43A, CALM1, PAPPA2, UBE2Q2, TRIM24, CCSAP, PROCR, LRRC1, EZR) | 0.500 (0.500, 0.500) | 0.400 (0.327, 0.473) | 0.719 | 0.504 | 0.714 (±0.0138) | 0.952 (±0.0012) | \| | \| |  |  | root |
| │ ├── CliXO:284 (PODXL, GPX3) | 0.500 (0.500, 0.500) | 0.400 (0.327, 0.473) | 0.719 | 0.504 | 0.558 (±0.0121) | 0.201 (±0.0038) | \| | \| |  |  |  |
| ├── CliXO:312 (TMEM45A, APH1B, VEGFA, CRISPLD2) | 0.500 (0.500, 0.500) | 0.400 (0.327, 0.473) | 0.719 | 0.504 | 0.558 (±0.0121) | 0.201 (±0.0038) | \| | \| |  |  |  |
| │ ├── CliXO:303 (VEGFA, CRISPLD2) | 0.500 (0.500, 0.500) | 0.400 (0.327, 0.473) | 0.719 | 0.504 | 0.558 (±0.0121) | 0.201 (±0.0038) | \| | \| |  |  |  |
| └── CliXO:345 (GPR146, SUV39H1, PIK3CB, CORO2A, BASP1, DLX5, SFXN3, SLC6A8, ZMYND8, SEMA4C, LRRC8A, CNNM4, HEY1, SEC23A, MIDN, EAF1, ERRFI1, PFKFB4, SLC2A1, VWCE, ANKRD37, ITGA5, GALK1, FANCE, MME, PPL, SWAP70, CYP2J2, MOB3A, SEMA4B, FBLIM1, ALKBH5, EFNB1, TSC22D1, KRT19, GUCA2A, C4orf3, SPAG4, SLC16A3, NECTIN4, TPP1, BCAT2, EPS8L2, GREM2, KLF4, DUSP1, DNAJC1, PIK3R1, GLA, NPEPPS, EPHB3, CSNK2A2, SIPA1L1, TOR1B, CLIC3, WFS1, CEBPA, PLIN2, MYOF, TMEM45A, PLOD2, KLF3, EPS8L1, IGSF8, GRHL1, CXXC1, ENG, ARID3A, FLNB, TIMP3, PPP1R12C, PIP5KL1, ELF4, FSTL3, BCORL1, INHA, MAP3K9, SYDE1, PMEL, SHANK2, TREM1, HK1, QPCT, GSN, CCDC127, ZNF331, GRHL3, GDPD3, P2RY2, MAML2, ACKR2, CDO1, P4HA1, PREP, CYB5R1, PRKAB2, MAP3K8, VEGFA, EFHD1, SCARB1, BHLHE40, FOSL2, JUNB, HMOX1, SDC4, BAD, DPP7, FPGS, PRKCH, CDK9, DONSON, ERO1A, NEK11, KIT, GPT2, HPCAL1, TRIP10, KMT2E, DIO2, MXD1, STK24, CRISPLD2, BCAR3, EBI3, ASTE1, HMGB3, SH3BP5, IGSF3, RASEF, SH3PXD2A, BTD, FKBP8, PIK3AP1, PGBD2, MIIP, SH3RF1, FRMD4A, TLE6, NFIL3, CEBPD, GPX3, HSPA2, LYN, ARRDC3, PJA1, GBE1, LEP, ILVBL, PAM, GALE, PKM, HTRA1, LIMCH1, FAM43A, CALM1, PAPPA2, UBE2Q2, TRIM24, CCSAP, PROCR, LRRC1, EZR) | 0.818 (0.802, 0.835) | 0.645 (0.527, 0.762) | 0.833 | 0.504 | 0.689 (±0.0132) | 0.919 (±0.0012) | \| | \| |  | ✓ | root |
| ├── CliXO:338 (GPR146, PIK3CB, DLX5, SFXN3, SEMA4C, LRRC8A, EAF1, PFKFB4, VWCE, ITGA5, GALK1, MME, CYP2J2, MOB3A, SEMA4B, ALKBH5, C4orf3, SPAG4, SLC16A3, NECTIN4, EPS8L2, GREM2, KLF4, DNAJC1, GLA, NPEPPS, CSNK2A2, SIPA1L1, TOR1B, CLIC3, TMEM45A, PLOD2, KLF3, EPS8L1, IGSF8, CXXC1, ENG, ARID3A, FLNB, PPP1R12C, ELF4, FSTL3, BCORL1, INHA, MAP3K9, SYDE1, PMEL, SHANK2, HK1, QPCT, GSN, ZNF331, GDPD3, ACKR2, P4HA1, PREP, PRKAB2, SCARB1, FOSL2, SDC4, CDK9, DONSON, ERO1A, GPT2, DIO2, CRISPLD2, SH3BP5, IGSF3, SH3PXD2A, BTD, FKBP8, PIK3AP1, SH3RF1, FRMD4A, TLE6, HSPA2, GBE1, LEP, GALE, CCSAP, PROCR) | 0.773 (0.754, 0.792) | 0.645 (0.527, 0.762) | 0.798 | 0.504 | 0.633 (±0.0115) | 0.833 (±0.0010) | \| | \| |  | ✓ |  |
| │ ├── CliXO:290 (GLA, BTD) | 0.500 (0.500, 0.500) | 0.400 (0.327, 0.473) | 0.719 | 0.504 | 0.558 (±0.0121) | 0.201 (±0.0038) | \| | \| |  |  |  |
| │ ├── CliXO:291 (EPS8L1, SHANK2) | 0.500 (0.500, 0.500) | 0.400 (0.327, 0.473) | 0.719 | 0.504 | 0.558 (±0.0121) | 0.201 (±0.0038) | \| | \| |  |  |  |
| │ ├── CliXO:299 (PLOD2, P4HA1) | 0.500 (0.500, 0.500) | 0.400 (0.327, 0.473) | 0.719 | 0.504 | 0.558 (±0.0121) | 0.201 (±0.0038) | \| | \| |  |  |  |
| │ └── CliXO:307 (INHA, FKBP8, GALE) | 0.500 (0.500, 0.500) | 0.400 (0.327, 0.473) | 0.719 | 0.504 | 0.558 (±0.0121) | 0.201 (±0.0038) | \| | \| |  |  |  |
| └── CliXO:344 (GPR146, SUV39H1, PIK3CB, CORO2A, BASP1, DLX5, SFXN3, SLC6A8, ZMYND8, SEMA4C, LRRC8A, CNNM4, HEY1, SEC23A, MIDN, EAF1, ERRFI1, PFKFB4, SLC2A1, VWCE, ANKRD37, ITGA5, GALK1, FANCE, MME, PPL, SWAP70, CYP2J2, MOB3A, SEMA4B, FBLIM1, ALKBH5, EFNB1, TSC22D1, KRT19, GUCA2A, C4orf3, SPAG4, SLC16A3, NECTIN4, TPP1, BCAT2, EPS8L2, GREM2, KLF4, DUSP1, DNAJC1, PIK3R1, NPEPPS, EPHB3, CSNK2A2, SIPA1L1, CLIC3, WFS1, CEBPA, PLIN2, MYOF, TMEM45A, PLOD2, KLF3, EPS8L1, IGSF8, GRHL1, ENG, ARID3A, FLNB, TIMP3, PPP1R12C, PIP5KL1, ELF4, FSTL3, BCORL1, INHA, MAP3K9, SYDE1, PMEL, TREM1, HK1, QPCT, GSN, CCDC127, ZNF331, GRHL3, GDPD3, P2RY2, MAML2, ACKR2, CDO1, P4HA1, PREP, CYB5R1, PRKAB2, MAP3K8, VEGFA, EFHD1, SCARB1, BHLHE40, FOSL2, JUNB, HMOX1, SDC4, BAD, DPP7, FPGS, PRKCH, CDK9, ERO1A, NEK11, KIT, GPT2, HPCAL1, TRIP10, KMT2E, DIO2, MXD1, STK24, CRISPLD2, BCAR3, EBI3, ASTE1, HMGB3, SH3BP5, IGSF3, RASEF, SH3PXD2A, BTD, FKBP8, PIK3AP1, PGBD2, MIIP, FRMD4A, NFIL3, CEBPD, GPX3, LYN, ARRDC3, PJA1, GBE1, LEP, ILVBL, PAM, GALE, PKM, HTRA1, LIMCH1, FAM43A, CALM1, PAPPA2, UBE2Q2, TRIM24, CCSAP, PROCR, LRRC1, EZR) | 0.818 (0.802, 0.835) | 0.645 (0.527, 0.762) | 0.574 | 0.504 | 0.564 (±0.0089) | 0.724 (±0.0010) | \|✓ | \| |  | ✓ | root |
| ├── CliXO:296 (NEK11, ASTE1) | 0.500 (0.500, 0.500) | 0.400 (0.327, 0.473) | 0.719 | 0.504 | 0.558 (±0.0121) | 0.201 (±0.0038) | \| | \| |  |  |  |
| ├── CliXO:318 (ANKRD37, PLIN2, MAP3K8, BHLHE40, NFIL3, ARRDC3) | 0.500 (0.500, 0.500) | 0.400 (0.327, 0.473) | 0.719 | 0.504 | 0.558 (±0.0121) | 0.201 (±0.0038) | \| | \| |  |  |  |
| ├── CliXO:333 (PIK3CB, SLC6A8, SEMA4C, LRRC8A, VWCE, ITGA5, GALK1, FANCE, MOB3A, FBLIM1, ALKBH5, EFNB1, KRT19, GUCA2A, SPAG4, TPP1, TMEM45A, IGSF8, ENG, FLNB, FSTL3, INHA, GDPD3, P4HA1, CYB5R1, DPP7, HPCAL1, EBI3, BTD, GPX3, LEP, GALE, PROCR) | 0.500 (0.500, 0.500) | 0.400 (0.327, 0.473) | 0.719 | 0.504 | 0.515 (±0.0082) | 0.308 (±0.0016) | \| | \| |  |  |  |
| │ ├── CliXO:293 (DPP7, GPX3) | 0.500 (0.500, 0.500) | 0.400 (0.327, 0.473) | 0.719 | 0.504 | 0.558 (±0.0121) | 0.201 (±0.0038) | \| | \| |  |  |  |
| │ └── CliXO:315 (SLC6A8, VWCE, GALK1, SPAG4) | 0.500 (0.500, 0.500) | 0.400 (0.327, 0.473) | 0.719 | 0.504 | 0.558 (±0.0121) | 0.201 (±0.0038) | \| | \| |  |  |  |
| └── CliXO:343 (GPR146, SUV39H1, PIK3CB, CORO2A, BASP1, DLX5, SFXN3, SLC6A8, ZMYND8, SEMA4C, LRRC8A, CNNM4, HEY1, SEC23A, MIDN, EAF1, ERRFI1, PFKFB4, SLC2A1, VWCE, ANKRD37, ITGA5, GALK1, FANCE, MME, PPL, SWAP70, CYP2J2, MOB3A, FBLIM1, ALKBH5, EFNB1, TSC22D1, KRT19, GUCA2A, C4orf3, SPAG4, SLC16A3, NECTIN4, TPP1, BCAT2, EPS8L2, GREM2, KLF4, DUSP1, DNAJC1, PIK3R1, NPEPPS, EPHB3, CSNK2A2, CLIC3, WFS1, CEBPA, PLIN2, MYOF, TMEM45A, PLOD2, KLF3, EPS8L1, IGSF8, GRHL1, ENG, ARID3A, FLNB, TIMP3, PPP1R12C, PIP5KL1, ELF4, FSTL3, INHA, MAP3K9, SYDE1, TREM1, QPCT, ZNF331, GRHL3, P2RY2, MAML2, ACKR2, CDO1, P4HA1, PREP, CYB5R1, PRKAB2, MAP3K8, VEGFA, EFHD1, SCARB1, BHLHE40, FOSL2, HMOX1, SDC4, BAD, DPP7, FPGS, PRKCH, CDK9, ERO1A, NEK11, KIT, GPT2, HPCAL1, TRIP10, KMT2E, DIO2, MXD1, STK24, CRISPLD2, BCAR3, EBI3, HMGB3, SH3BP5, IGSF3, RASEF, SH3PXD2A, BTD, FKBP8, PIK3AP1, FRMD4A, CEBPD, GPX3, LYN, ARRDC3, PJA1, GBE1, LEP, ILVBL, PAM, GALE, PKM, HTRA1, LIMCH1, FAM43A, CALM1, PAPPA2, UBE2Q2, TRIM24, CCSAP, PROCR, LRRC1, EZR) | 0.813 (0.798, 0.828) | 0.645 (0.527, 0.762) | 0.694 | 0.504 | 0.698 (±0.0134) | 0.929 (±0.0012) | \| | \| |  | ✓ | root |
| │ ├── CliXO:293 (DPP7, GPX3) | 0.500 (0.500, 0.500) | 0.400 (0.327, 0.473) | 0.719 | 0.504 | 0.558 (±0.0121) | 0.201 (±0.0038) | \| | \| |  |  |  |
| ├── CliXO:294 (ZNF331, KMT2E) | 0.500 (0.500, 0.500) | 0.400 (0.327, 0.473) | 0.719 | 0.504 | 0.558 (±0.0121) | 0.201 (±0.0038) | \| | \| |  |  |  |
| │ ├── CliXO:303 (VEGFA, CRISPLD2) | 0.500 (0.500, 0.500) | 0.400 (0.327, 0.473) | 0.719 | 0.504 | 0.558 (±0.0121) | 0.201 (±0.0038) | \| | \| |  |  |  |
| ├── CliXO:334 (PIK3CB, BASP1, SFXN3, SLC6A8, LRRC8A, CNNM4, SLC2A1, ITGA5, GALK1, MOB3A, ALKBH5, GUCA2A, SPAG4, SLC16A3, NECTIN4, KLF4, EPHB3, CLIC3, WFS1, TMEM45A, ENG, ARID3A, PPP1R12C, INHA, TREM1, P2RY2, SCARB1, BAD, FPGS, PRKCH, CDK9, MXD1, EBI3, RASEF, LEP) | 0.500 (0.500, 0.500) | 0.400 (0.327, 0.473) | 0.719 | 0.504 | 0.514 (±0.0081) | 0.311 (±0.0015) | \| | \| |  |  | root |
| │ └── CliXO:327 (CNNM4, SLC2A1, ALKBH5, NECTIN4, EPHB3, WFS1, ENG, INHA, TREM1, P2RY2, SCARB1, PRKCH, MXD1, EBI3, LEP) | 0.227 (0.208, 0.246) | 0.155 (0.126, 0.184) | 0.350 | 0.004 | 0.518 (±0.0082) | 0.310 (±0.0016) | \| | \| |  |  | root |
| └── CliXO:342 (GPR146, SUV39H1, PIK3CB, CORO2A, BASP1, DLX5, SFXN3, SLC6A8, ZMYND8, SEMA4C, LRRC8A, HEY1, MIDN, EAF1, ERRFI1, PFKFB4, SLC2A1, VWCE, ANKRD37, ITGA5, GALK1, MME, PPL, CYP2J2, MOB3A, ALKBH5, EFNB1, KRT19, GUCA2A, C4orf3, SPAG4, SLC16A3, NECTIN4, EPS8L2, GREM2, KLF4, DUSP1, PIK3R1, NPEPPS, EPHB3, CSNK2A2, CLIC3, WFS1, PLIN2, MYOF, TMEM45A, PLOD2, KLF3, EPS8L1, GRHL1, ENG, ARID3A, FLNB, PPP1R12C, ELF4, FSTL3, INHA, MAP3K9, SYDE1, QPCT, GRHL3, P2RY2, MAML2, ACKR2, CDO1, P4HA1, PRKAB2, EFHD1, SCARB1, BHLHE40, FOSL2, BAD, CDK9, ERO1A, NEK11, GPT2, TRIP10, KMT2E, DIO2, MXD1, STK24, BCAR3, EBI3, HMGB3, SH3BP5, IGSF3, RASEF, SH3PXD2A, BTD, FKBP8, PIK3AP1, FRMD4A, LYN, ARRDC3, LEP, ILVBL, PAM, GALE, PKM, HTRA1, LIMCH1, CALM1, PAPPA2, UBE2Q2, TRIM24, CCSAP, PROCR, LRRC1, EZR) | 0.293 (0.272, 0.314) | 0.400 (0.327, 0.473) | 0.318 | 0.504 | 0.585 (±0.0091) | 0.747 (±0.0008) | \| | \| |  |  | root |
| ├── CliXO:295 (WFS1, LYN) | 0.500 (0.500, 0.500) | 0.400 (0.327, 0.473) | 0.719 | 0.504 | 0.558 (±0.0121) | 0.201 (±0.0038) | \| | \| |  |  |  |
| ├── CliXO:300 (MAML2, KMT2E) | 0.500 (0.500, 0.500) | 0.400 (0.327, 0.473) | 0.719 | 0.504 | 0.558 (±0.0121) | 0.201 (±0.0038) | \| | \| |  |  |  |
| │ └── CliXO:307 (INHA, FKBP8, GALE) | 0.500 (0.500, 0.500) | 0.400 (0.327, 0.473) | 0.719 | 0.504 | 0.558 (±0.0121) | 0.201 (±0.0038) | \| | \| |  |  |  |
| └── CliXO:336 (GPR146, SUV39H1, PIK3CB, CORO2A, SFXN3, SLC6A8, SEMA4C, LRRC8A, HEY1, MIDN, VWCE, ANKRD37, ITGA5, GALK1, MME, PPL, CYP2J2, MOB3A, ALKBH5, EFNB1, KRT19, SPAG4, SLC16A3, NECTIN4, DUSP1, PLIN2, MYOF, TMEM45A, PLOD2, KLF3, EPS8L1, GRHL1, ENG, ARID3A, FLNB, PPP1R12C, ELF4, FSTL3, INHA, MAP3K9, SYDE1, QPCT, P2RY2, ACKR2, P4HA1, PRKAB2, EFHD1, SCARB1, BHLHE40, FOSL2, ERO1A, GPT2, KMT2E, STK24, SH3BP5, RASEF, SH3PXD2A, PIK3AP1, LYN, ARRDC3, LEP, PAM, PKM, LIMCH1, CALM1, PAPPA2, UBE2Q2, TRIM24, CCSAP, PROCR, LRRC1) | 0.707 (0.686, 0.728) | 0.400 (0.327, 0.473) | 0.779 | 0.504 | 0.606 (±0.0144) | 0.118 (±0.0087) | \| | \| |  |  | root |
| │ ├── CliXO:299 (PLOD2, P4HA1) | 0.500 (0.500, 0.500) | 0.400 (0.327, 0.473) | 0.719 | 0.504 | 0.558 (±0.0121) | 0.201 (±0.0038) | \| | \| |  |  |  |
| ├── CliXO:311 (ANKRD37, DUSP1, BHLHE40, ARRDC3) | 0.293 (0.272, 0.314) | 0.400 (0.327, 0.473) | 0.476 | 0.504 | 0.527 (±0.0092) | 0.292 (±0.0018) | \| | \| |  |  | root |
| │ └── CliXO:315 (SLC6A8, VWCE, GALK1, SPAG4) | 0.500 (0.500, 0.500) | 0.400 (0.327, 0.473) | 0.719 | 0.504 | 0.558 (±0.0121) | 0.201 (±0.0038) | \| | \| |  |  |  |
| └── CliXO:331 (GPR146, PIK3CB, SFXN3, KRT19, SPAG4, NECTIN4, TMEM45A, ENG, FLNB, FSTL3, INHA, SYDE1, QPCT, P4HA1, BHLHE40, ERO1A, GPT2, KMT2E, SH3BP5, RASEF, SH3PXD2A, PIK3AP1, LEP, LIMCH1, CALM1, UBE2Q2, CCSAP, LRRC1) | 0.500 (0.500, 0.500) | 0.400 (0.327, 0.473) | 0.719 | 0.504 | 0.558 (±0.0121) | 0.201 (±0.0038) | \| | \| |  |  |  |

The Reported column indicates the ontology from Tables 1–2 to which this row’s eligible genes belong. An ontology is reported if it passes all criteria (C1–C4) and its eligible gene set is not a strict subset of another passing ontology’s gene set. The column is empty when the ontology has no eligible genes or does not pass all criteria. ✓, the ontology passed the criterion; †, the gene had a grad-CAM value >97.5th percentile in the same ontology; |, separator between PPV/TPR and NPV/TNR results in C1 and C2 columns; AUC-PRC, area under the precision-recall curve; AUC-ROC, area under the receiver operating characteristic curve; C1, lower bounds finite and >0; C2, absolute difference ≤25% between development and test sets; C3, at least 1 eligible gene; C4, lower bound of AUC-ROC >0.5 in both sets; CI, confidence interval; Dev, development set; grad-CAM, gradient-weighted class activation mapping; HELLP, hemolysis, elevated liver enzyme, and low platelet; PE, preeclampsia; RMSE, root mean square error.

Table S19. Definitions of genes identified in the pathogenesis models.

| Gene | Full name | Definition |
| --- | --- | --- |
| *ANKRD13A* | Ankyrin repeat domain 13A | Endocytic trafficking; ubiquitinated receptor binding; receptor internalization & degradation |
| *SLA* | Src Like Adaptor | T-cell receptor signaling; negative regulation of immune activation; cytokine response modulation |
| *PDE5A* | Phosphodiesterase 5A | Hydrolyzes cGMP; smooth muscle contraction; vasodilation; platelet function; nitric oxide signaling |
| *ALKBH5* | AlkB Homolog 5 | m6A RNA demethylase; RNA stability; fertility; stress responses |
| *SWAP70* | SWAP Switching B Cell Complex 70 kDa Subunit | Actin cytoskeleton dynamics; B-cell activation; membrane trafficking |
| *DUSP1* | Dual Specificity Phosphatase 1 | MAPK phosphatase; stress response; inflammation control; apoptosis regulation |
| *KLF4* | Kruppel Like Factor 4 | Transcription factor; stem-cell reprogramming; epithelial homeostasis; inflammation |
| *GREM2* | Gremlin 2, DAN Family BMP Antagonist | BMP signaling inhibitor; development; tissue remodeling |
| *GPR146* | G Protein Coupled Receptor 146 | Orphan GPCR; lipid metabolism; cardiovascular regulation |
| *PIP5K1B* | Phosphatidylinositol-4-Phosphate 5-Kinase Type 1 Beta | Generates PIP2; cytoskeletal dynamics; membrane trafficking; signal transduction |
| *EZR* | Ezrin | Cytoskeleton–membrane linker; cell shape; migration; adhesion; signaling |

Table S20. Internal and external validation AUC-ROC of the ablation study comparing *t*-SNE, PCA, and UMAP for three-dimensional feature map construction in DI-VNN across the seven pathogenesis models.*t*-SNE, PCA, and UMAP for three-dimensional feature map construction in DI-VNN across the seven pathogenesis models.

| PE Subtype | Associated Factor | t-SNE (int.) | t-SNE (ext.) | PCA (int.) | PCA (ext.) | UMAP (int.) | UMAP (ext.) |
| --- | --- | --- | --- | --- | --- | --- | --- |
| LOPE | Mid-secretory endometrium | 0.500 | 0.467 | 0.875 | 0.045 | 0.500 | 0.045 |
| LOPE | Chorioamnionitis (T3) | 1.000 | 0.765 | 0.875 | 0.500 | 0.500 | 0.500 |
| LOPE | HELLP syndrome (T3) | 1.000 | 0.500 | 1.000 | 0.500 | 0.500 | 0.500 |
| EOPE | Placentation (T2) | 0.875 | 0.628 | 0.750 | 0.820 | 1.000 | 0.488 |
| EOPE | Late-secretory endometrium | 1.000 | 0.640 | 1.000 | 0.524 | 0.750 | 0.517 |
| EOPE | Placentation (T1) | 0.625 | 0.513 | 1.000 | 0.526 | 0.750 | 0.519 |
| EOPE | HELLP syndrome (T3) | 1.000 | 0.621 | 0.125 | 0.565 | 0.500 | 0.500 |
| **Mean** |  | **0.857** | **0.591** | **0.804** | **0.497** | **0.643** | **0.438** |

Each model was trained on the development set with four L2 regularization values (0.01, 0.1, 1, 10) for 30 tuning epochs. The best L2 was selected using a weighted criterion balancing validation AUC-ROC and training-validation gap. Final training used the best L2 for up to 500 epochs with patience of 250. Internal validation (int) used an 80/20 split of the development set for early stopping during model training. External validation (ext.) used an independent validation set with diverse microarray platforms, normalized by aligning each sample’s expression distribution to the average expression profile of controls in the development set using quantile normalization with a target distribution. AUC-ROC, area under the receiver operating characteristic curve; DI-VNN, deep-insight visible neural network; EOPE, early-onset, severe preeclampsia; ext., external validation; HELLP, hemolysis, elevated liver enzyme, and low platelet; int, internal validation; LOPE, late-onset, severe preeclampsia; PCA, principal component analysis; T1, first trimester; T2, second trimester; T3, third trimester; *t*-SNE, *t*-moderated stochastic neighbor embedding; UMAP, uniform manifold approximation and projection.

Figure S1. Net benefit bar plots for the late-onset, severe PE pathogenesis model connected with mid-secretory endometrium. Each bar represents the net benefit of the model (blue) or the reference (grey) at the selected threshold for each ontology. The y-axis labels show the ontology name with the threshold value in brackets (th). CliXO, clique-extracted ontology; GO, gene ontology; HELLP, hemolysis, elevated liver enzyme, and low platelet; PE, preeclampsia; th, threshold.

Figure S2. Net benefit bar plots for the late-onset, severe PE pathogenesis model connected with third-trimester placenta under chorioamnionitis. Each bar represents the net benefit of the model (blue) or the reference (grey) at the selected threshold for each ontology. The y-axis labels show the ontology name with the threshold value in brackets (th). CliXO, clique-extracted ontology; GO, gene ontology; HELLP, hemolysis, elevated liver enzyme, and low platelet; PE, preeclampsia; th, threshold.

Figure S3. ROC curves for the late-onset, severe PE pathogenesis model connected with third-trimester placenta under chorioamnionitis. Binormal-smoothed ROC curves are shown for ontologies passing all criteria (C1–C4). The dashed diagonal line represents chance performance. Each color represents a different ontology. AUC-ROC, area under the receiver operating characteristic curve; C1–C4, cascading validation criteria; CliXO, clique-extracted ontology; FPR, false positive rate; GO, gene ontology; HELLP, hemolysis, elevated liver enzyme, and low platelet; PE, preeclampsia; ROC, receiver operating characteristic; TPR, true positive rate.

Figure S4. Net benefit bar plots for the late-onset, severe PE pathogenesis model connected with third-trimester placenta under HELLP syndrome. Each bar represents the net benefit of the model (blue) or the reference (grey) at the selected threshold for each ontology. The y-axis labels show the ontology name with the threshold value in brackets (th). CliXO, clique-extracted ontology; GO, gene ontology; HELLP, hemolysis, elevated liver enzyme, and low platelet; PE, preeclampsia; th, threshold.

Figure S5. ROC curves for the late-onset, severe PE pathogenesis model connected with third-trimester placenta under HELLP syndrome. Binormal-smoothed ROC curves are shown for ontologies passing all criteria (C1–C4). The dashed diagonal line represents chance performance. Each color represents a different ontology. AUC-ROC, area under the receiver operating characteristic curve; C1–C4, cascading validation criteria; CliXO, clique-extracted ontology; FPR, false positive rate; GO, gene ontology; HELLP, hemolysis, elevated liver enzyme, and low platelet; PE, preeclampsia; ROC, receiver operating characteristic; TPR, true positive rate.

Figure S6. Net benefit bar plots for the early-onset, severe PE pathogenesis model connected with late-secretory endometrium. Each bar represents the net benefit of the model (blue) or the reference (grey) at the selected threshold for each ontology. The y-axis labels show the ontology name with the threshold value in brackets (th). CliXO, clique-extracted ontology; GO, gene ontology; HELLP, hemolysis, elevated liver enzyme, and low platelet; PE, preeclampsia; th, threshold.

Figure S7. Net benefit bar plots for the early-onset, severe PE pathogenesis model connected with first-trimester placenta. Each bar represents the net benefit of the model (blue) or the reference (grey) at the selected threshold for each ontology. The y-axis labels show the ontology name with the threshold value in brackets (th). CliXO, clique-extracted ontology; GO, gene ontology; HELLP, hemolysis, elevated liver enzyme, and low platelet; PE, preeclampsia; th, threshold.

Figure S8. Net benefit bar plots for the early-onset, severe PE pathogenesis model connected with second-trimester placenta. Each bar represents the net benefit of the model (blue) or the reference (grey) at the selected threshold for each ontology. The y-axis labels show the ontology name with the threshold value in brackets (th). CliXO, clique-extracted ontology; GO, gene ontology; HELLP, hemolysis, elevated liver enzyme, and low platelet; PE, preeclampsia; th, threshold.

Figure S9. Net benefit bar plots for the early-onset, severe PE pathogenesis model connected with third-trimester placenta under HELLP syndrome. Each bar represents the net benefit of the model (blue) or the reference (grey) at the selected threshold for each ontology. The y-axis labels show the ontology name with the threshold value in brackets (th). CliXO, clique-extracted ontology; GO, gene ontology; HELLP, hemolysis, elevated liver enzyme, and low platelet; PE, preeclampsia; th, threshold.

Figure S10. ROC curves for the early-onset, severe PE pathogenesis model connected with third-trimester placenta under HELLP syndrome. Binormal-smoothed ROC curves are shown for ontologies passing all criteria (C1–C4). The dashed diagonal line represents chance performance. Each color represents a different ontology. AUC-ROC, area under the receiver operating characteristic curve; C1–C4, cascading validation criteria; CliXO, clique-extracted ontology; FPR, false positive rate; GO, gene ontology; HELLP, hemolysis, elevated liver enzyme, and low platelet; PE, preeclampsia; ROC, receiver operating characteristic; TPR, true positive rate.

# References

1.	Reimand J, Isserlin R, Voisin V, et al. Pathway enrichment analysis and visualization of omics data using g:Profiler, GSEA, Cytoscape and EnrichmentMap. *Nature protocols*. Feb 2019;14(2):482-517. doi:10.1038/s41596-018-0103-9

2.	Ma J, Yu MK, Fong S, et al. Using deep learning to model the hierarchical structure and function of a cell. *Nature methods*. Apr 2018;15(4):290-298. doi:10.1038/nmeth.4627

3.	Kim CJ, Romero R, Chaemsaithong P, Chaiyasit N, Yoon BH, Kim YM. Acute chorioamnionitis and funisitis: definition, pathologic features, and clinical significance. *American journal of obstetrics and gynecology*. Oct 2015;213(4 Suppl):S29-52. doi:10.1016/j.ajog.2015.08.040

4.	Lisonkova S, Joseph KS. Incidence of preeclampsia: risk factors and outcomes associated with early- versus late-onset disease. *American journal of obstetrics and gynecology*. Dec 2013;209(6):544.e1-544.e12. doi:10.1016/j.ajog.2013.08.019

5.	Sufriyana H, Wu YW, Su EC. Low- and high-level information analyses of transcriptome connecting endometrial-decidua-placental origin of preeclampsia subtypes: A preliminary study. *Pacific Symposium on Biocomputing Pacific Symposium on Biocomputing*. 2024;29:549-563.

6.	Sisti G, Faraci A, Silva J, Upadhyay R. Neutrophil-to-Lymphocyte Ratio, Platelet-to-Lymphocyte Ratio, and Routine Complete Blood Count Components in HELLP Syndrome: A Matched Case Control Study. *Medicina (Kaunas)*. May 8 2019;55(5)doi:10.3390/medicina55050123

7.	Gardikioti A, Venou TM, Gavriilaki E, et al. Molecular Advances in Preeclampsia and HELLP Syndrome. *Int J Mol Sci*. Mar 31 2022;23(7)doi:10.3390/ijms23073851

8.	Sufriyana H, Wu YW, Su EC. Human-guided deep learning with ante-hoc explainability by convolutional network from non-image data for pregnancy prognostication. *Neural networks : the official journal of the International Neural Network Society*. May 2023;162:99-116. doi:10.1016/j.neunet.2023.02.020

9.	Hu M, Alkhairy S, Lee I, et al. Evaluation of large language models for discovery of gene set function. *Research square*. Sep 18 2023;doi:10.21203/rs.3.rs-3270331/v1
